# A blood atlas of COVID-19 defines hallmarks of disease severity and specificity

**DOI:** 10.1016/j.cell.2022.01.012

**Published:** 2022-03-03

**Authors:** COvid-19 Multi-omics Blood ATlas (COMBAT) Consortium, David J. Ahern, Zhichao Ai, Mark Ainsworth, Chris Allan, Alice Allcock, Brian Angus, M. Azim Ansari, Carolina V. Arancibia-Cárcamo, Dominik Aschenbrenner, Moustafa Attar, J. Kenneth Baillie, Eleanor Barnes, Rachael Bashford-Rogers, Archana Bashyal, Sally Beer, Georgina Berridge, Amy Beveridge, Sagida Bibi, Tihana Bicanic, Luke Blackwell, Paul Bowness, Andrew Brent, Andrew Brown, John Broxholme, David Buck, Katie L. Burnham, Helen Byrne, Susana Camara, Ivan Candido Ferreira, Philip Charles, Wentao Chen, Yi-Ling Chen, Amanda Chong, Elizabeth A. Clutterbuck, Mark Coles, Christopher P. Conlon, Richard Cornall, Adam P. Cribbs, Fabiola Curion, Emma E. Davenport, Neil Davidson, Simon Davis, Calliope A. Dendrou, Julie Dequaire, Lea Dib, James Docker, Christina Dold, Tao Dong, Damien Downes, Hal Drakesmith, Susanna J. Dunachie, David A. Duncan, Chris Eijsbouts, Robert Esnouf, Alexis Espinosa, Rachel Etherington, Benjamin Fairfax, Rory Fairhead, Hai Fang, Shayan Fassih, Sally Felle, Maria Fernandez Mendoza, Ricardo Ferreira, Roman Fischer, Thomas Foord, Aden Forrow, John Frater, Anastasia Fries, Veronica Gallardo Sanchez, Lucy C. Garner, Clementine Geeves, Dominique Georgiou, Leila Godfrey, Tanya Golubchik, Maria Gomez Vazquez, Angie Green, Hong Harper, Heather A. Harrington, Raphael Heilig, Svenja Hester, Jennifer Hill, Charles Hinds, Clare Hird, Ling-Pei Ho, Renee Hoekzema, Benjamin Hollis, Jim Hughes, Paula Hutton, Matthew A. Jackson-Wood, Ashwin Jainarayanan, Anna James-Bott, Kathrin Jansen, Katie Jeffery, Elizabeth Jones, Luke Jostins, Georgina Kerr, David Kim, Paul Klenerman, Julian C. Knight, Vinod Kumar, Piyush Kumar Sharma, Prathiba Kurupati, Andrew Kwok, Angela Lee, Aline Linder, Teresa Lockett, Lorne Lonie, Maria Lopopolo, Martyna Lukoseviciute, Jian Luo, Spyridoula Marinou, Brian Marsden, Jose Martinez, Philippa C. Matthews, Michalina Mazurczyk, Simon McGowan, Stuart McKechnie, Adam Mead, Alexander J. Mentzer, Yuxin Mi, Claudia Monaco, Ruddy Montadon, Giorgio Napolitani, Isar Nassiri, Alex Novak, Darragh P. O'Brien, Daniel O'Connor, Denise O'Donnell, Graham Ogg, Lauren Overend, Inhye Park, Ian Pavord, Yanchun Peng, Frank Penkava, Mariana Pereira Pinho, Elena Perez, Andrew J. Pollard, Fiona Powrie, Bethan Psaila, T. Phuong Quan, Emmanouela Repapi, Santiago Revale, Laura Silva-Reyes, Jean-Baptiste Richard, Charlotte Rich-Griffin, Thomas Ritter, Christine S. Rollier, Matthew Rowland, Fabian Ruehle, Mariolina Salio, Stephen Nicholas Sansom, Raphael Sanches Peres, Alberto Santos Delgado, Tatjana Sauka-Spengler, Ron Schwessinger, Giuseppe Scozzafava, Gavin Screaton, Anna Seigal, Malcolm G. Semple, Martin Sergeant, Christina Simoglou Karali, David Sims, Donal Skelly, Hubert Slawinski, Alberto Sobrinodiaz, Nikolaos Sousos, Lizzie Stafford, Lisa Stockdale, Marie Strickland, Otto Sumray, Bo Sun, Chelsea Taylor, Stephen Taylor, Adan Taylor, Supat Thongjuea, Hannah Thraves, John A. Todd, Adriana Tomic, Orion Tong, Amy Trebes, Dominik Trzupek, Felicia Anna Tucci, Lance Turtle, Irina Udalova, Holm Uhlig, Erinke van Grinsven, Iolanda Vendrell, Marije Verheul, Alexandru Voda, Guanlin Wang, Lihui Wang, Dapeng Wang, Peter Watkinson, Robert Watson, Michael Weinberger, Justin Whalley, Lorna Witty, Katherine Wray, Luzheng Xue, Hing Yuen Yeung, Zixi Yin, Rebecca K. Young, Jonathan Youngs, Ping Zhang, Yasemin-Xiomara Zurke

**Keywords:** coronavirus, SARS-CoV-2, COVID-19, blood, immune, transcriptomics, epigenetics, proteomics, multi-omics, personalized medicine

## Abstract

Treatment of severe COVID-19 is currently limited by clinical heterogeneity and incomplete description of specific immune biomarkers. We present here a comprehensive multi-omic blood atlas for patients with varying COVID-19 severity in an integrated comparison with influenza and sepsis patients versus healthy volunteers. We identify immune signatures and correlates of host response. Hallmarks of disease severity involved cells, their inflammatory mediators and networks, including progenitor cells and specific myeloid and lymphocyte subsets, features of the immune repertoire, acute phase response, metabolism, and coagulation. Persisting immune activation involving AP-1/p38MAPK was a specific feature of COVID-19. The plasma proteome enabled sub-phenotyping into patient clusters, predictive of severity and outcome. Systems-based integrative analyses including tensor and matrix decomposition of all modalities revealed feature groupings linked with severity and specificity compared to influenza and sepsis. Our approach and blood atlas will support future drug development, clinical trial design, and personalized medicine approaches for COVID-19.

## Highlights

• Blood atlas delineating innate and adaptive immune dysregulation in COVID-19
• Shared and specific immune signatures of COVID-19, influenza and all cause sepsis
• Multi-omic immune profiling differentiates hospitalized patient severity in COVID-19
• Immune activation and proliferation involving AP-1/p38MAPK associated with COVID-19

## Graphical abstract

**Figure:**
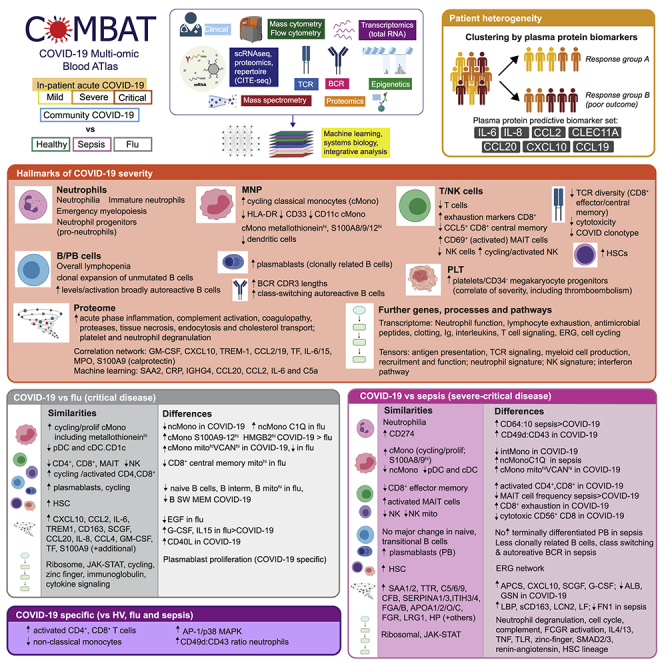

## Teaser

A multi-omic analysis of patient blood samples reveals both similarities and specific features of COVID-19 when compared with samples obtained from sepsis or influenza patients, which could yield better targeted therapies for severe COVID-19.

## Introduction

The pathophysiology associated with severe acute respiratory syndrome coronavirus 2 (SARS-CoV-2) reflects a complex interplay between virus-induced lung pathology and maladaptive host immune responses (Kuri-Cervantes et al., 2020; Mathew et al., 2020; Tay et al., 2020). Severe COVID-19 is characterized by hypoxia, with risk of rapid deterioration, progression to acute respiratory distress syndrome, multiorgan failure, and death. Predisposing factors include age, gender, ethnicity, obesity, and comorbidities. Currently, opportunities for biomarker-led timed and targeted precision medicine approaches are limited by an incomplete understanding of pathogenesis and heterogeneity among patients with severe disease (Wynants et al., 2020). A dysregulated hyperinflammatory state occurs in some individuals (Moore and June, 2020), consistent with reported benefits from glucocorticoids (dexamethasone), inhibitors of the IL-6 receptor (tocilizumab/sarilumab), and Janus kinases (baricitinib) (Gordon et al., 2021; Horby et al., 2021a, 2021b; Kalil et al., 2021). Nevertheless, blood-derived signatures of severity are diverse, including evidence of immune suppression, myeloid dysfunction, lymphopenia, interferon driven immunopathology, T cell activation/exhaustion, and immune senescence (Bost et al., 2021; Chen and John Wherry, 2020; Diao et al., 2020; Hadjadj et al., 2020; Mann et al., 2020; Schulte-Schrepping et al., 2020). Comparison with other severe respiratory viruses such as influenza show differences in target cells and control of viral replication but also shared mechanisms, notably a dysregulated host response (Flerlage et al., 2021; Lee et al., 2020; Zhu et al., 2020). Features of cytokine hyperactivation and lymphocyte exhaustion are proposed as shared mechanisms of severe COVID-19 with sepsis (Arunachalam et al., 2020; Boomer et al., 2012; Diao et al., 2020). Here, we demonstrate the informativeness of a multi-modal, integrative systems biology approach through the COvid-19 Multi-omics Blood ATlas (COMBAT) consortium. We identify cells, mediators, and pathways in peripheral blood that are hallmarks of increasing COVID-19 severity; resolve shared and specific features with influenza and sepsis; and define potential biomarkers of the variable individual response to SARS-CoV-2 infection to support a future personalized medicine approach.

## Results

### Clinical features, severity metrics, and disease stratification in COVID-19

**Figure 1:**
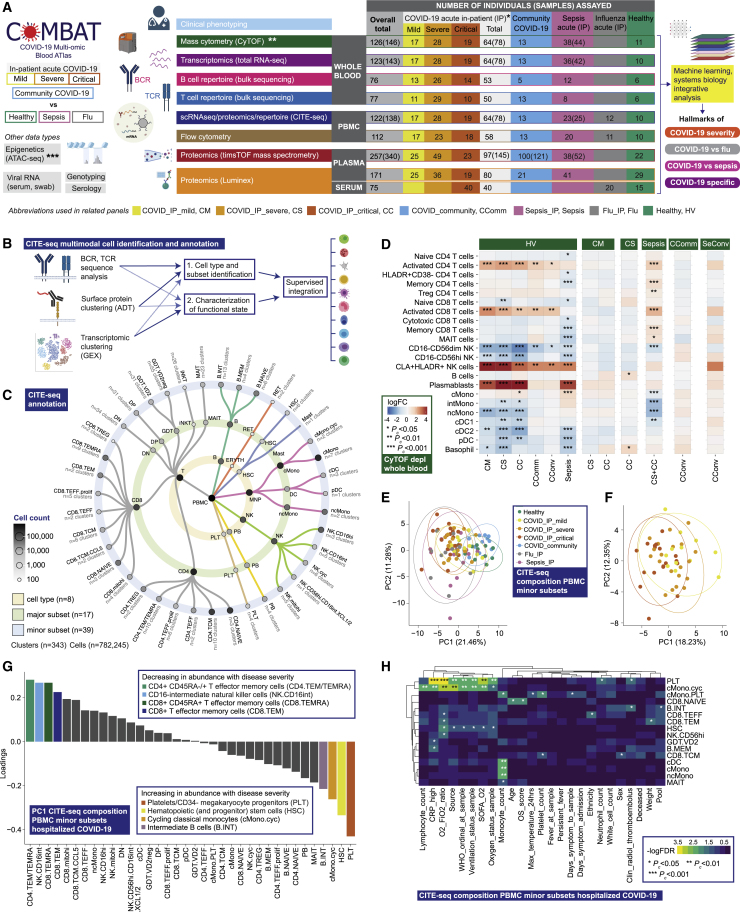
Single cell compositional analysis reveals variance in cell populations by clinical group and severity (A) Study design, assay modalities, and workflow. Table shows number of patients assayed, with number of samples in brackets where more than one sample assayed. ^∗^WHO severity categories show number of patients at time of sampling ^∗∗^single paired convalescent sample assayed for n = 16 COVID-19 and n = 3 sepsis patients; ^∗∗∗^10 samples assayed (8 samples for paired acute-convalescent COVID-19 and 2 healthy). (B) Summary of supervised multimodal annotation strategy for the CITE-seq data (described in STAR Methods; clustering of GEX modality shown in Figure S1E). (C) Summary of cell populations identified by CITE-seq (phenotypes shown in Data S4). (D) Differential abundance of major cell populations in granulocyte (CD66^+^) depleted whole blood where significant between comparator groups (7,118,158 cells assayed using single cell mass cytometry). (E–H) CITE-seq compositional analysis of minor cell subsets. (E and F) Principal components analysis (PCA) showing PC1 versus PC2 with 95% data ellipses (assuming a multivariate t-distribution) of (E) all comparator groups and (F) hospitalized COVID-19 cases. (G) Loadings of minor cell subsets on PC1 for hospitalized COVID-19 cases. (H) Covariate analysis for clinical, demographic, and experimental variables for hospitalized COVID-19 cases plotting significant minor cell subsets (BH adjusted ANOVA for significance). See Figures S1 and S2.

We aimed to characterize the peripheral blood response in COVID-19. To do this, we analyzed a prospective cohort of adult patients with confirmed SARS-CoV-2 presenting to clinical services at the start of the United Kingdom pandemic (February–March 2020). We recruited 116 hospitalized COVID-19 patients following informed consent at a single site (Oxford University Hospitals) through the Sepsis Immunomics study (ethics approval South Central - Oxford C Research Ethics Committee in England Ref 19/SC/0296) (STAR Methods, Table S1). The overall mortality rate was 23.3%. Samples were collected during the acute admission and in survivors from 28 days after discharge (convalescent samples). We compared these patients with community COVID-19 cases in the recovery phase (never admitted to hospital), age-matched healthy volunteers, influenza cases requiring mechanical ventilation (critically ill receiving intensive care), and all-cause sepsis patients (hospitalized encompassing severe and critical disease) recruited prior to the pandemic (Figure 1A; STAR Methods; Table S1).

**Figure S1:**
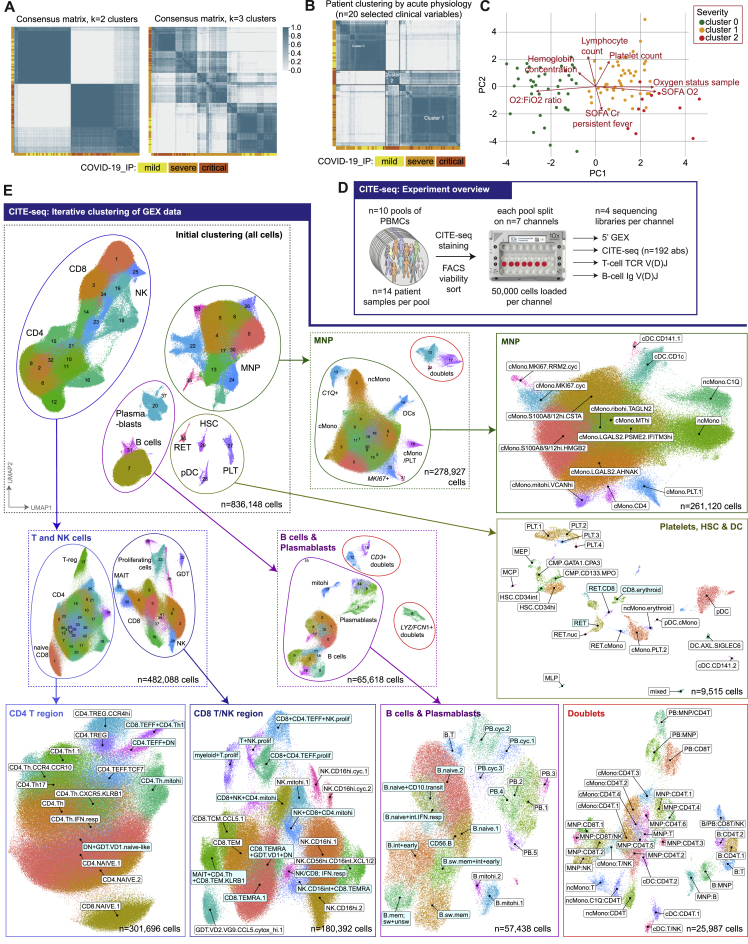
Study cohorts, clinical covariates and CITE-seq analysis, related to Figure 1 (A,B) Unsupervised clustering of samples from hospitalized COVID-19 patients by consensus k-means clustering followed by hierarchical clustering on the consensus matrix based on (A) 49 clinical features (excluding WHO severity classifiers) to determine patient groupings demonstrated the optimal cluster number was 2 or 3 (B) acute measures of physiology and clinical biomarkers of response without significant missingness (including measures of oxygenation requirements, blood cell counts, fever, ALT, CRP) (Data S2). (C) Biplot illustrating for PC1 and PC2 features driving clustering identified in Figure S1B. (D) Overview of the CITE-seq experiment. A total of n = 140 PBMC samples from COVID-19, sepsis, influenza and healthy volunteers were mixed into n = 10 pools (left). Each pool comprised of n = 14 samples from different individuals. After staining, viable cells were isolated by FACS and captured using n = 7 10X channels per pool (center). From each channel, four libraries were generated from gene expression (GEX), surface proteome (ADT), TCR repertoire and BCR repertoire modalities (right). (E) UMAP plots showing the iterative gene expression (GEX) clustering of the CITE-seq dataset. Initial GEX clustering of all cells identified four subgroups (as demarcated by the ellipses in the UMAP, top left). Re-clustering of the T and NK cells (middle left) identified two major subgroups which were extracted for final cluster analysis as the “CD4 T region” and “CD8 T/NK region” (bottom left and bottom center left). A similar process was followed for analysis of the B cells and plasmablasts (PB) (middle center and bottom center-right) and for the mononuclear phagocytes (MNP) (top center and top-right). A number of doublet clusters were identified during initial re-clustering of the B/PB and MNPs and were re-clustered separately (bottom-right). The final group of cells identified in the initial clustering consisted of platelets (PLT), hematopoietic stem (and progenitor) cells (HSC) and some dendritic cells (DC) which were extracted and clustered together (middle right). The initial and intermediate clustering steps are shown in dashed boxes, while the final set of GEX clusters that were annotated and used as an input for the multimodal annotation are shown in the solid boxes. As described in the STAR Methods, highly variable gene discovery, integration and clustering were performed separately for each of the clustering results shown. For this figure, the final six GEX clustering analyses (bottom and right) were labeled by mapping the multimodal annotations back onto the GEX manifolds: white labels indicate that > 80% of cells in the GEX cluster mapped to the given multimodal cell cluster, cyan labels indicate a mapping to multiple multimodal cell clusters (indicative names shown).

To optimize our chances of identifying specific cellular and molecular biomarkers that might in future have clinical utility, we analyzed patient classifiers of illness severity derived from clinical features in our hospitalized COVID-19 cohort (STAR Methods). This showed severity scores and surrogate markers of illness response were highly correlated (Data S2) and that patient clusters showed broad concordance to the first-released WHO categorical criteria, namely mild (no requirement for supplemental oxygen), severe (oxygen saturation SaO_2_ ≤ 93% on air but not requiring mechanical ventilation), and critical (requiring mechanical ventilation) (Figure S1A). This clustering persisted when we restricted the analysis to acute measures of physiology and clinical biomarkers, where the main correlates of consensus clustering related to ventilation status (Figures S1B and S1C). Accordingly, hereinafter we refer to WHO categorical criteria as our primary hospitalized COVID-19 severity comparator groups.

### COVID-19 severity is associated with differences in abundance of diverse immune cell populations

**Figure S2:**
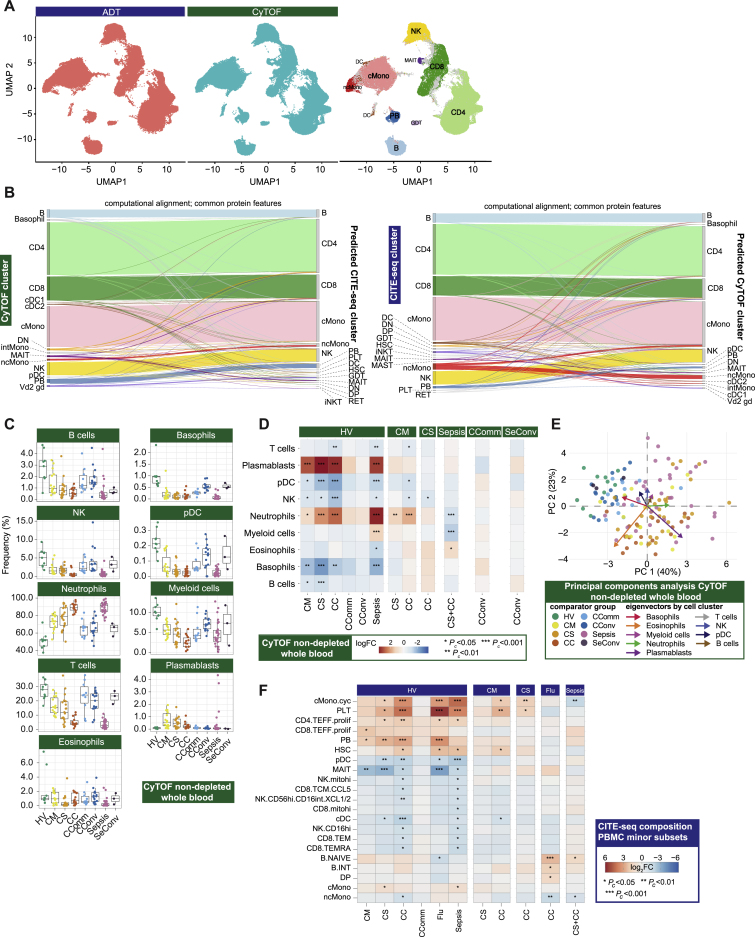
Single cell compositional approaches, related to Figure 1 (A-B) Concordance and cross validation of cell composition using single cell resolution mass cytometry (Helios CyTOF system) clustering (from granulocyte (CD66^+^) depleted whole blood with down sampling to a maximum of 75,000 cells and 7,118,158 cells assayed) and CITE-seq analysis of viability sorted peripheral blood mononuclear cells (PBMCs) from 140 samples profiled using the 10X Genomics platform. (A) Concordance in cell composition annotation between assay types is demonstrated with UMAP showing joint visualization of CITE-seq and CyTOF datasets including side by side plot of CITE-seq cell surface protein quantification (ADT) and mass cytometry together with a plot of cell annotations transferred between datasets and colored by cell type where concordant (94.5% of cells) or discordant (gray) (B) Plots demonstrating cross validation of mass cytometry and CITE-seq cell clusters. (C-E) Stabilized whole blood (Cytodelics) from COVID-19 patients (non-granulocyte depleted samples) analyzed by mass cytometry (including matched samples collected during convalescence from 16 COVID-19 hospitalized patients). A self-organizing map algorithm (FlowSOM) resolved 25 clusters by consensus clustering for 3,893,390 cells after down sampling to a maximum of 40,000 cells. Clusters merged to identify broad immune cell populations (Data S3). (C) Cell frequencies by clinical group. Boxplots show median, first and third quartiles; whiskers show 1.5x interquartile range. (D) Differential abundance analysis in patients compared to healthy volunteers, and different disease states clustering major cell populations using empirical Bayes analysis (statistical inference estimating priors from the data). (E) PCA with arrows indicating drivers of variation by cell population. (F) Differential abundance analysis in patients compared to healthy volunteers, and between disease categories for minor cell subsets using empirical Bayes analysis. Abbreviations for CITE-seq (panel F). B: B cell; cDC: classical dendritic cell; cMono: classical monocytes; cyc: cycling; DC: dendritic cell; DN: CD4/CD8 double negative; DP: CD4/CD8: double positive; ERYTH: erythrocyte; GDT: gamma delta T; hi: high; HSC: hematopoietic stem (and progenitor) cells; iNKT: invariant natural killer T; INT/int: intermediate; MAIT: Mucosal associated invariant T; MEM: memory; mito: mitochondrial; MNP: mononuclear phagocyte; ncMono: non-classical monocyte; neg: negative; NK: natural killer cell; PB: plasmablast; PBMC: peripheral blood mononuclear cell; pDC: plasmacytoid dendritic cell; PLT: platelet/CD34- megakaryocyte progenitor; prolif: proliferating; RET: reticulocyte; T: T cell; TCM: T central memory; TEFF: T effector; TEM(RA): T effector memory (CD45RA re-expressing); TREG: T regulatory cell. Comparator group abbreviations. HV: healthy volunteer; CM: COVID-19 in-patient mild; CS: COVID-19 in-patient severe; CC: COVID-19 in-patient critical; CComm: COVID-19 community case in the recovery phase (never admitted to hospital); CConv: COVID-19 convalescence (survivors from 28 days after discharge); Flu: influenza in-patient critical; Sepsis: in-patient severe and critical sepsis; SeConv: sepsis convalescence.

We first investigated changes in cellular composition associated with COVID-19 severity using mass cytometry of whole blood (Figure 1A; STAR Methods; Data S3), and Cellular Indexing of Transcriptomes and Epitopes by Sequencing (CITE-seq) to annotate peripheral blood mononuclear cell (PBMC) types, subsets, and clusters (Figures 1A–1C, S1D, S1E, S2A, and S2B; STAR Methods; Table S2; Data S3: Analysis of mass and flow cytometry, repertoire, and proteomics, related to STAR Methods, Data S4: Multimodal annotation of CITE-seq data, related to STAR Methods, Figure 1, and Table S2, Data S5: Compositional analysis of CITE-seq data, related to STAR Methods). We defined a “prioritized sample set” comprising for each patient a single sample closest to onset of maximal disease as defined by clinical features at the time of sampling. Mass cytometry of whole blood showed increased absolute and relative neutrophil abundance and reduced overall T and B lymphocyte, myeloid, dendritic, basophil, and natural killer (NK) cells in more severe/critical disease; differential abundance of specific cell populations included increased activated and cytotoxic CD8^+^ T cells and classical monocytes (Figures 1D, S2C, and S2D; Data S3). In community and convalescent COVID-19 patients, the cell composition was broadly comparable to healthy volunteers, although some differences in abundance persisted. The frequency of neutrophils, plasmablasts, plasmacytoid DCs (pDC), and basophils contributed significantly to the overall variance in cell composition between patient groups (Figure S2E).

We validated and further characterized these differences using CITE-seq. Cellular composition differed by patient group and by severity in hospitalized COVID-19 patients (Figures 1E–1H and S2F). By reducing the dimensionality of the data, we found the highest proportion of the observed variance (principal component [PC] 1) was associated with comparator group (*P*_*c*_ = 2.01×10^−15^, ANOVA) (Figure 1E; Data S5). When hospitalized COVID-19 cases were analyzed alone, the largest component of variance (PC1) was associated with group membership, oxygenation status (SaO_2_/FiO_2_ ratio, SOFA oxygenation, and ventilation and oxygen status), and lymphocyte count (all *P*_*c*_ < 0.05) (Figure 1F; Data S5). The cell subsets with higher abundance in more severe disease that contributed most to this component (largest negative loadings in PC1) were platelets/CD34^−^ megakaryocyte progenitors, hematopoietic stem (and progenitor) cells (HSCs), and cycling classical monocytes (cMono) (Figure 1G). Differential abundance analysis with age-/gender-matched healthy volunteers showed differences in the same cell populations, together with reduced dendritic cells (DCs), T, and NK cell subsets, particularly in COVID-19 critical cases (Figure S2F). Among hospitalized COVID-19 cases, higher abundance of platelets/CD34^−^ megakaryocyte progenitors and cycling cMono was associated with oxygenation status (SaO_2_/FiO_2_, SOFA oxygenation score, ventilation status), severity (WHO ordinal), and CRP (all *P*_*c*_ < 0.01). Higher platelet/CD34^−^ megakaryocyte progenitor abundance was also associated with the occurrence of thromboembolism during hospitalization (*P*_*c*_ = 0.036, ANOVA) (Figure 1H; Data S5), highlighting their potential pathophysiological significance in severe COVID-19.

### Whole blood hallmarks of COVID-19 and severity involving neutrophils, progenitor cells, lymphocyte exhaustion, clotting, immunoglobulins, and the interferon response

**Figure 2:**
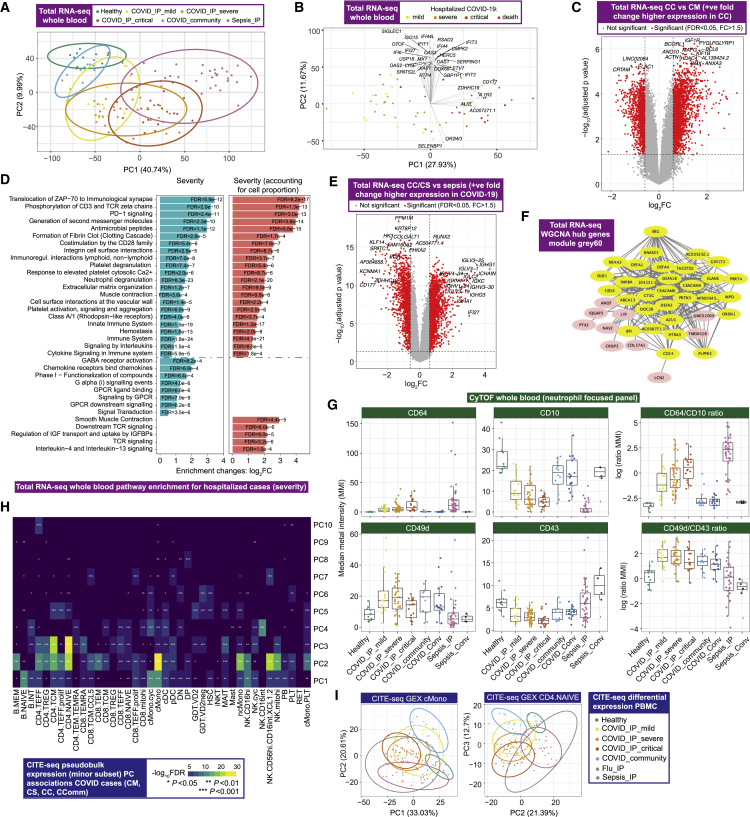
Signatures of COVID-19 response from transcriptomics (A–F) Whole blood total RNA-seq. (A and B) Principal component (PC) analysis of (A) all comparator groups and (B) hospitalized COVID-19 cases. (C) Differential gene expression critical versus mild COVID-19. (D) Pathway enrichment for COVID-19 severity as a quantitative trait ± inclusion cell proportion. (E) Differential gene expression COVID-19 versus sepsis. (F) Intramodular hub genes for weighted gene correlation network analysis module grey60. (G) Neutrophil cell surface proteins assayed by mass cytometry shown by marker or ratio of markers. Boxplots show median and first and third quartiles; whiskers show 1.5x interquartile range. (H and I) CITE-seq gene expression. (H) Association of PCs of expression variance within minor cell subsets in COVID-19 patients. (I) PC plots in classical monocytes and naive CD4^+^ T cells. See Figures S3 and S4.

**Figure S3:**
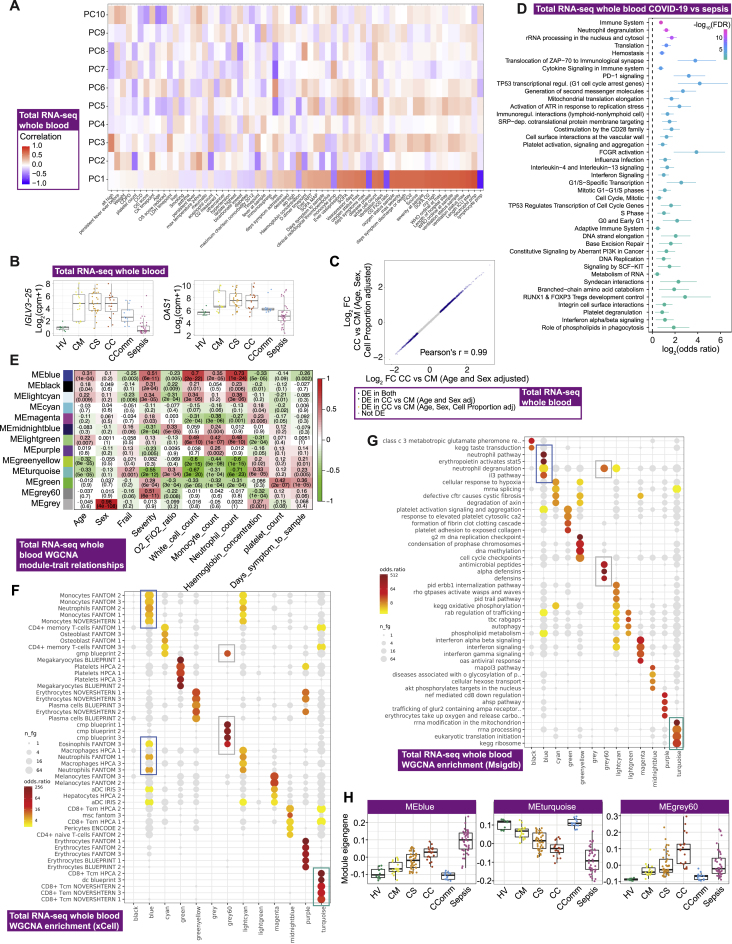
Signatures of COVID-19 severity revealed by bulk RNA-seq, related to Figure 2 Whole blood total RNA-seq for hospitalized COVID-19 patients showing (A) matrix correlation of principal components (PCs) with covariates (B) differentially expressed immunoglobulin lambda chain gene *IGLV3-25* and innate viral response gene *OAS1* and (C) correlation plot showing the influence of cell proportion on detection of differentially expressed genes. (D) Pathway enrichment for COVID-19 severe and critical versus sepsis using Reactome. Bars indicate 95% confidence intervals. (E-H) Weighted gene correlation network analysis (WGCNA) of whole blood total RNA-seq. (E) Heatmap showing module trait relationships. (F,G) Enrichment of WGCNA modules using gene expression data showing for (F) 64 immune and stroma cell types (xCell), (G) MSigDB canonical pathway genesets, and (H) module eigengene values plotted by patient group. Comparator group abbreviations HV: healthy volunteer; CM: COVID-19 in-patient mild; CS: COVID-19 in-patient severe; CC: COVID-19 in-patient critical; CComm: COVID-19 community case in the recovery phase (never admitted to hospital); Sepsis: in-patient severe and critical sepsis.

We next defined global transcriptomic signatures of the host response to COVID-19 by performing whole blood total RNA-sequencing (Figure 1A; STAR Methods). We investigated overall variance in gene expression for all individuals using the prioritized sample set and found clear separation by clinical group, namely between healthy volunteers, increasing severity among COVID-19 cases and patients with sepsis (Figure 2A). Among hospitalized COVID-19 patients, the largest component of variance was associated with 28-day mortality (PC1, p = 2.34×10^−6^, Kruskal-Wallis test) (Figure 2B), which also correlated with other measures of severity and differential cell count (Figure S3A). We found that genes contributing to this component (highest loadings for PC1) were strongly enriched for immune system function, notably neutrophil degranulation (fold change [FC] = 4.23, false discovery rate [FDR] = 2.4×10^−15^), PD-1 signaling (FC = 21.5, FDR = 9.2×10^−12^) (consistent with lymphocyte exhaustion), antimicrobial peptides (FC = 10.8, FDR = 9.2×10^−7^), and clotting cascade (FC = 10.6, FDR = 2.1×10^−5^). The second largest component of variance in the data (PC2) showed strong enrichment for genes involved in interferon signaling (FC = 10.7, FDR = 3×10^−33^) including key viral response network genes (*IFI1-3*, *IFI6*, *IFI44*, *IFIT3*, and *OAS1-3*) and specific immunoglobulin heavy and lambda genes (Figure S3B).

We further analyzed genes differentially expressed according to COVID-19 severity among hospitalized patients and found greatest enrichment for T cell receptor (TCR) and PD-1 signaling, antimicrobial peptides, fibrin clot formation, integrin and immunoregulatory interactions, and platelet and neutrophil degranulation. This was robust to inclusion of cell proportion as a covariate (Figures 2C, 2D, and S3C). We found these aspects of the response to COVID-19 were largely distinct from non-SARS-CoV-2 sepsis of comparable severity (Figure 2A). Specific features of COVID-19 compared to sepsis included upregulation in COVID-19 of many immunoglobulin heavy/kappa/lambda genes and unique pathway enrichments relating to cell proliferation and innate/adaptive immune function (Figures 2E and S3D).

Next, we identified clusters of highly interconnected genes (modules) correlated with COVID-19 severity using weighted gene correlation network analysis (WGCNA) (Star Methods; Figure S3E). The three modules most significantly correlated with severity (p < 1×10^−10^) were enriched for, respectively, cellular and functional neutrophil gene signatures and neutrophil count (MEblue module); CD8^+^ T cell signatures and relative lymphopenia (MEturquoise module); and granulocyte and common myeloid progenitor cell gene signatures, neutrophil degranulation, antimicrobial peptides, and defensin pathways (MEGrey60 module) (Figures S3F and S3G). The MEgrey60 module was more highly expressed in critical COVID-19 than sepsis (Figure S3H), and the ETS transcription factor related gene *ERG*, which regulates lineage plasticity, showed the highest intramodule connectivity (Figure 2F). These features indicate the MEgrey60 module represents a variety of progenitor cells that associate with severity, further supporting the importance of these cells.

**Figure S4:**
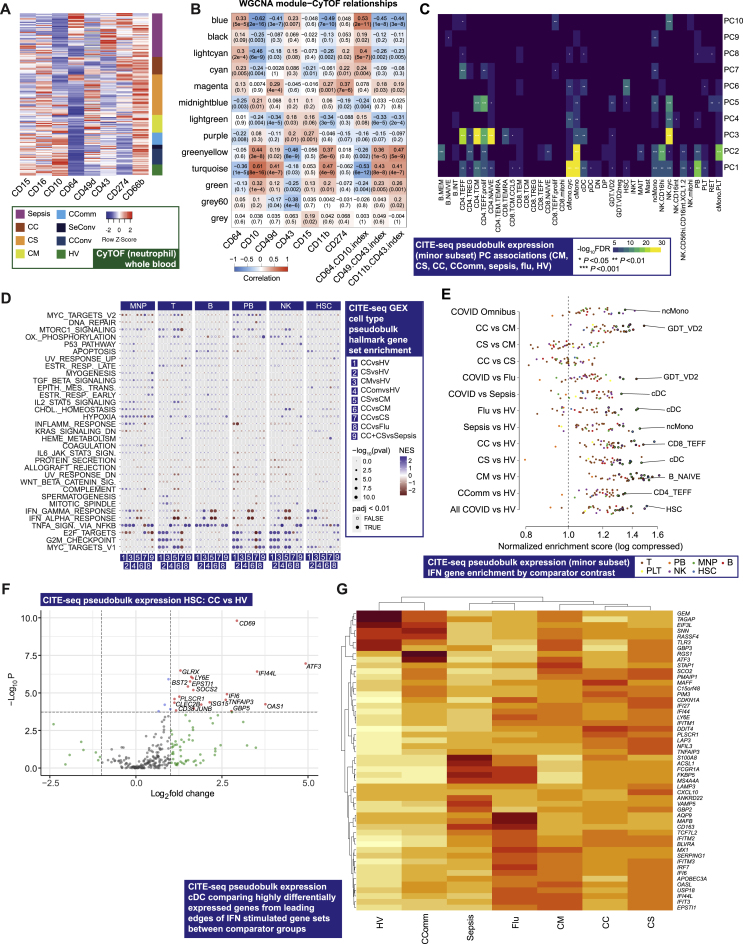
Signatures of COVID-19 severity revealed by single cell RNA-seq and mass cytometry, related to Figure 2 (A) Neutrophil marker expression whole blood assayed by mass cytometry comparing across patient groups. (B) Correlations between whole blood total RNA-seq WGCNA modules and neutrophil CyTOF markers. (C) Association p values between principal components of pseudobulk GEX for specific cell clusters (minor subsets) across all clinical groups. (D) scRNA-seq MSigDB hallmark gene set enrichment by cell type. All boxplots show median, first and third quartiles; whiskers show 1.5x interquartile range. (E) Enrichment of interferon-stimulated genes for each pair of minor subset and contrasts. Circled dots have p < 1e-5 (Bonferroni-corrected threshold for the number of subsets/contrasts pairs). The most significant cell subset is highlighted for each contrast. (F) Volcano plot of differential expression between critical COVID-19 and healthy controls, restricted to interferon-stimulated genes, in the HSC minor subset. (G) A hierarchically clustered heatmap of gene expression in classical dendritic cells (cDCs) of highly differentially expressed (FDR < 0.001, absolute fold change > 3) genes from the leading edges of interferon stimulated gene sets. Color shows mean zero-centered RPM in units of standard deviations within each group. Comparator group abbreviations HV: healthy volunteer; CM: COVID-19 in-patient mild; CS: COVID-19 in-patient severe; CC: COVID-19 in-patient critical; CComm: COVID-19 community case in the recovery phase (never admitted to hospital); CConv: COVID-19 convalescence (survivors from 28 days after discharge); Flu: influenza in-patient critical; Sepsis: in-patient severe and critical sepsis; SeConv: sepsis convalescence.

To better characterize neutrophil populations in COVID-19, we applied a myeloid-marker enriched mass cytometry panel to the same samples (STAR Methods). We found evidence for the presence of immature neutrophils and neutrophil progenitors (pro-neutrophils) based on high expression of CD64 (Fc gamma receptor 1) and CD49d (integrin alpha 4), and decreased expression of CD10 (neutral endopeptidase) (Figure 2G) (Evrard et al., 2018; Kwok et al., 2020; Marini et al., 2017). CD64 expression was raised in severe/critical COVID-19 and further elevated in sepsis, together with increased PD-L1 (CD274) expression (Figure S4A). Using CD64:CD10 ratio as an index score for immature neutrophil presence, we found association with the MEblue module eigengene that correlated with neutrophil count and function (Figure S4B). We further determined that neutrophil CD49d expression was elevated, while CD43 (leukosialin) was reduced, in COVID-19 patients but was largely unchanged in sepsis (Figure 2G). The CD49d:CD43 ratio remained high in convalescence (Figure 2G).

### Shared and cell-type-specific single-cell gene expression signatures of COVID-19 involving ZFN, ribosomal and cell-cycle genes, and AP-1 and interferon signaling

To further deconvolute biological pathways and cellular functions associated with COVID-19 we analyzed gene expression at single-cell resolution (STAR Methods; Data S6; Table S3). Among hospitalized and community COVID-19 cases (prioritized sample set), we found the principal components of variance in gene expression involved cMono, naive B cells, plasmablasts, CD4^+^ T cells (naive, effector, and effector memory), and cycling NK cells (Figures 2H, 2I, and S4C). We performed pathway analysis for differentially expressed genes in major cell types (Figure S4D; STAR Methods) (Liberzon et al., 2015). This showed enrichment for type I and II interferon pathways in the less severe hospitalized COVID-19 patients across cell types. Redox state (reflected by MTORC1 signaling and oxidative phosphorylation) was enriched across mononuclear phagocytes (MNP), T cells, NK cells, and plasmablasts in more severe COVID-19, as were cell cycle (MYC targets, E2F targets, G2M checkpoint) pathways (except for MNP), while IL2-STAT5 pathway enrichment in T cells was found in more severe disease. Interferon stimulated genes showed enrichment in a range of cell subsets in COVID-19 cases, notably cDCs (Figure S4E–S4G).

**Figure 3:**
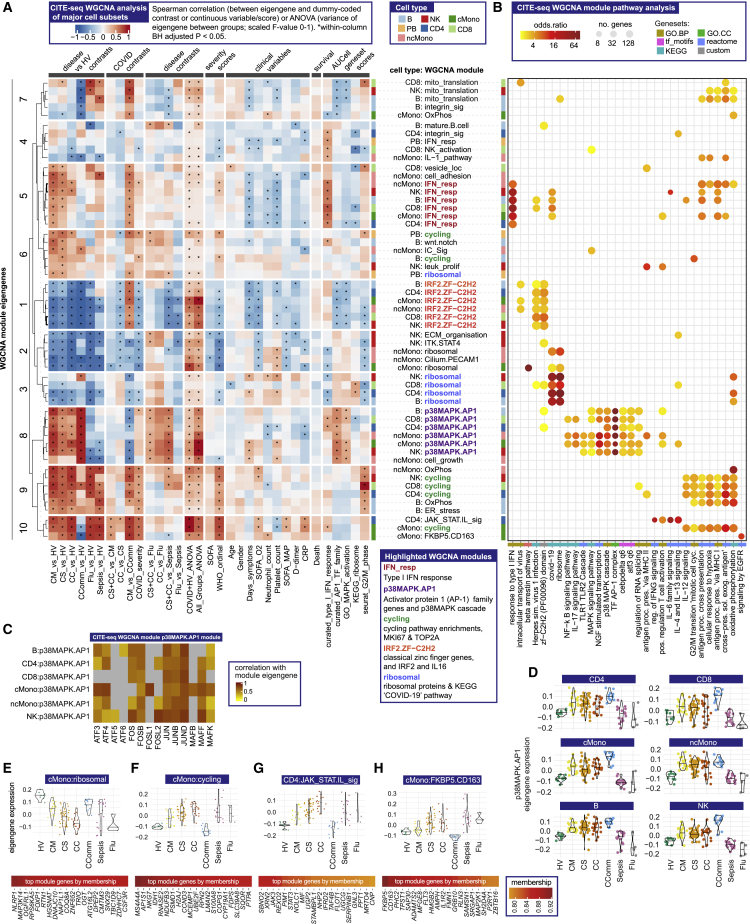
Single cell gene expression modules identify hallmarks of COVID-19 response (A–H) Weighted gene correlation network analysis (WGCNA) of CITE-seq gene expression for major cell types. (A) Association of module eigengenes with disease contrasts, clinical severity scores and variables, survival and gene set scores (^∗^all significant associations shown). (B) Module pathway enrichment. (C and D) p38MAPK.AP-1 module eigengene (C) correlation with AP-1 family genes (D) expression across patient groups. (E–H) Eigengene expression and top eigengene-gene correlations for (E) ribosomal module in cMono (F) cycling module in cMono (G) JAK-STAT.interleukin module in CD4 and (H) FKB5.CD163 module in cMono. For all violin plots, median indicated by horizontal bar.

We then determined network modules for major cell subsets by WGCNA (Figures 3A and 3B; Star Methods; Data S6). Analysis of module co-variation identified five distinct module sets. A set of type I IFN response modules was found across cell populations in which representative gene expression profiles (module eigengenes) correlated with milder disease, better oxygenation status, and earlier sampling from symptom onset (Figures 3A and 3B). The second module set, discovered in all cell types except plasmablasts, showed strong enrichment for activator protein 1 (AP-1) (FOS, JUN, ATF family genes) and the p38MAPK cascade. The module eigengenes were highly expressed in all COVID-19 patient groups, including recovery phase community cases; were distinct from influenza and sepsis; and did not show a consistent relationship with severity or other clinical features (Figures 3A–3D). The third module set was enriched for classical (C2H2) zinc finger (ZNF) genes and contained *IRF2* and *IL16*; expression of these eigengenes was lower in COVID-19 and influenza compared with healthy volunteers and sepsis cases (Figures 3A and 3B). The fourth set of modules involved ribosomal proteins and inflammasome function (top genes by membership included *NLRP1*, *MAP3K14*, and *FOXP1)* and was negatively correlated with COVID-19 severity in monocytes (Figures 3A, 3B, and 3E). Finally, we found a set of “cycling” modules, which in cMono correlated with severity, and included *S100A8/9* encoding calprotectin, a known severity biomarker (Silvin et al., 2020) (Figures 3A, 3B, and 3F). We also identified two cell-type-specific modules associated with severe disease, a JAK-STAT/interleukin signaling module in CD4^+^ T cells, and an EGFR pathway-enriched module in cMono including the stress response gene *FKBP5* and scavenger receptor *CD163* (Figures 3A, 3B, 3G, and 3H).

### Transcriptomic and epigenetic signatures of severity in monocyte populations

**Figure 4:**
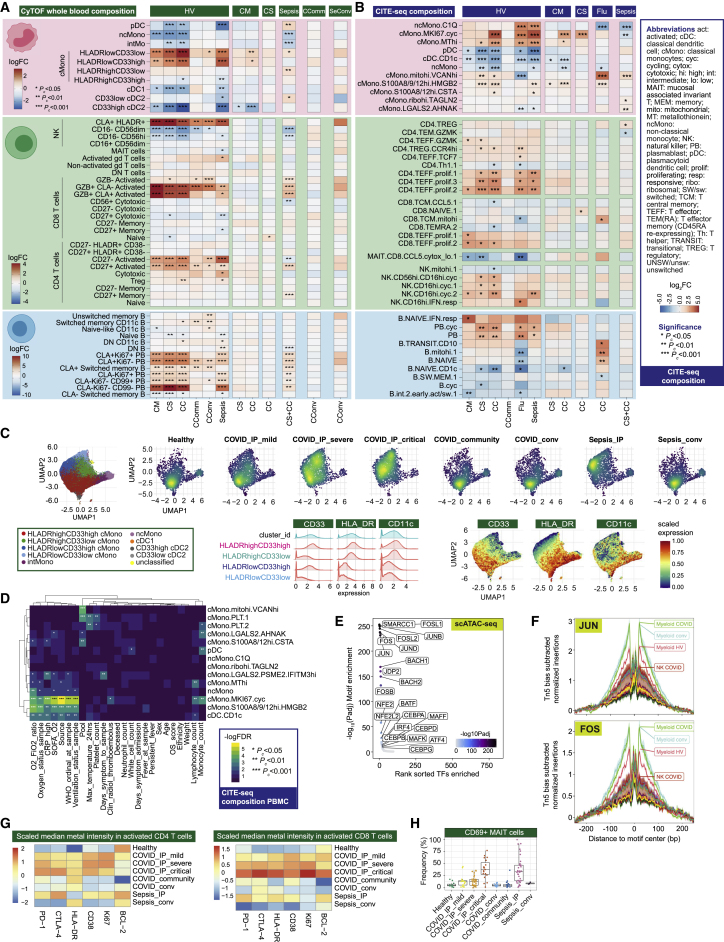
Changes in myeloid and lymphocyte cell populations associated with COVID-19 severity (A and B) Differential cell abundance in patients versus healthy volunteers, and between disease categories for myeloid, T, NK, and B cells for prioritized sample set assayed by (A) single cell mass cytometry and (B) CITE-seq, plotting cell populations where significant between comparator groups. (C) UMAP by patient group for myeloid cell clusters derived from mass cytometry and Mean Metal Intensity (MMI) of HLA-DR, CD33 and CD11c. (D) Covariate analysis of cell abundance assayed by CITE-seq and clinical, demographic, and experimental variables for hospitalized COVID-19 cases (BH adjusted ANOVA test for significance). (E and F) scATAC-seq (E) differential motif enrichment in myeloid cells, acute COVID-19 versus healthy volunteers and (F) transcription factor footprinting for myeloid enriched factors JUN and FOS. (G and H) Single cell mass cytometry (G) MMI of specific markers in activated CD4^+^ and CD8^+^ T lymphocytes (H) frequency of activated MAIT cells. Boxplots show median, first and third quartiles; whiskers 1.5x interquartile range. See Figures S5 and S6.

**Figure S5:**
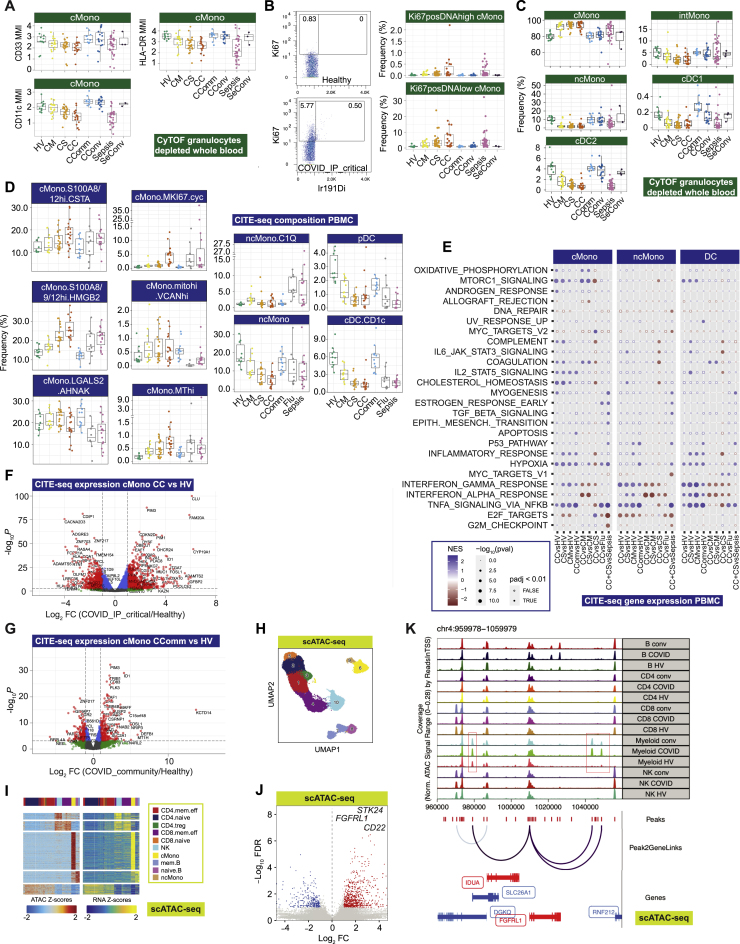
Changes in myeloid populations associated with COVID-19 severity, related to Figure 4 (A-C) Single cell mass cytometry. (A) Mean metal intensity (MMI) of HLA-DR, CD33 and CD11c for classical monocytes (cMono) by patient group. (B) Representative plot of Ki67^+^ expression and 191Iridium (DNA) labeling in a healthy volunteer and a COVID19 patient; two distinct population of Ki67^+^ proliferating cells were identified, one containing the same amount of DNA as Ki67^−^ cells (Ki67^+^DNA^low^) and a rarer population containing double the amount of DNA (Ki67^+^DNA^high^) which likely comprises proliferating cells in S, G2 and M phase. The boxplots describe the frequencies of Ki67^+^DNA^low^ and Ki67^+^DNA^high^ across different disease states. (C) Myeloid cell population frequencies by patient group. (D-G) CITE-seq PBMC myeloid cell clusters. (D) Differential abundance analysis with boxplots of cell cluster frequency by patient group where abundance significantly differs relative to healthy volunteers and (E) scRNA-seq MSigDB hallmark gene set enrichment for cMono, ncMono and DC. (F,G) Differential gene expression in classical monocytes comparing (F) critical COVID-19 patients versus healthy volunteers and (G) COVID-19 community cases versus healthy volunteers with volcano plots showing significant genes (FDR < 0.01 and logFC > 2) in red. (H-K) scATAC-seq with cell lysis, nuclear extraction and tagmentation on viability sorted PBMC prior to single nuclei capture and sequencing. Data shown for 42,000 cells post QC (ArchR pipeline) for 8 COVID-19 samples (paired acute and convalescent) and 2 healthy volunteers with (H) label transfer (unconstrained method) to assign cell clusters based on CITE-seq, (I) comparison of chromatin accessibility (scATAC-seq peaks linked to genes) to CITE-seq gene expression, (J) differential chromatin accessibility in myeloid cells comparing acute COVID-19 versus healthy volunteers, and (K) scATAC-seq tracks at *FGFRL1* locus comparing cell populations and condition (healthy, COVID-19 acute and convalescent). All boxplots show median, first and third quartiles; whiskers show 1.5x interquartile range. Abbreviations for CITE-seq (panels D-G). cDC: classical dendritic cell; cMono: classical monocytes; DC: dendritic cell; hi: high; MT, mitochondrial; ncMono: non-classical monocyte; pDC: plasmacytoid dendritic cell. Comparator group abbreviations. HV: healthy volunteer; CM: COVID-19 in-patient mild; CS: COVID-19 in-patient severe; CC: COVID-19 in-patient critical; CComm: COVID-19 community case in the recovery phase (never admitted to hospital); CConv: COVID-19 convalescence (survivors from 28 days after discharge); Flu: influenza in-patient critical; Sepsis: in-patient severe and critical sepsis; SeConv: sepsis convalescence.Comparator group abbreviations HV: healthy volunteer; CM: COVID-19 in-patient mild; CS: COVID-19 in-patient severe; CC: COVID-19 in-patient critical; CComm: COVID-19 community case in the recovery phase (never admitted to hospital); CConv: COVID-19 convalescence (survivors from 28 days after discharge); Flu: influenza in-patient critical; Sepsis: in-patient severe and critical sepsis; SeConv: sepsis convalescence.

We further investigated signatures of severity for specific mononuclear phagocyte populations (Figures 4A and 4B; STAR Methods; Data S3: Analysis of mass and flow cytometry, repertoire, and proteomics, related to STAR Methods, Data S4: Multimodal annotation of CITE-seq data, related to STAR Methods, Figure 1, and Table S2, Data S5: Compositional analysis of CITE-seq data, related to STAR Methods, Data S6: Gene expression analysis of CITE-seq data (principal component analysis, differential expression, and WGCNA parameters and modules), related to STAR Methods, Figure 3, and Table S3). Hospitalized COVID-19 patients with more severe disease had a relatively higher frequency of cMono and fewer intermediate monocytes (CD16^+^CD14^+^), ncMono (CD14^−^CD16^+^), and DCs (Figures 4A and S5A–S5C). With increasing disease severity, we found a shift in the phenotype of cMono to lower expression of HLA-DR, CD33, and CD11c, and evidence of proliferating monocytes based on expression of Ki-67 and DNA abundance, with comparable changes in sepsis patients (Figures 4C, S5A, and S5B). Lower levels of pDCs and CD33^low^cDC2 were found in sepsis compared with severe/critical COVID-19 (Figure 4A).

Further analysis of cMono using CITE-seq showed that a cycling cluster, and a cluster with high expression of the anti-oxidant metallothionein genes (MT^hi^), were significantly elevated in critical COVID-19 cases, influenza and sepsis compared to healthy volunteers (Figures 4B and S5D). We found that *S100A8/9/12*^hi^ *HMGB2*-expressing cMono correlated with COVID-19 severity and were also increased in sepsis. cMono expressing *VCAN*, which is implicated in cytokine release, were specifically increased in COVID-19 and reduced in influenza, while complement component *C1Q*-expressing ncMono were increased in influenza and sepsis, but not in COVID-19. pDCs showed reduced abundance in more severe COVID-19, influenza, and sepsis, as did CD1c^+^ cDCs. Consistent with the progressive changes in abundance according to COVID-19 severity, the frequencies of cycling cMono and *S100A8/9/12*^hi^ *HMGB2* expressing cMono, and CD1c^+^ cDCs, were associated with clinical variables relating to oxygenation and respiratory function in hospitalized cases (Figure 4D). Pathway enrichment analysis of differentially expressed genes identified inflammatory response/TNF signaling and interferon response in milder disease including community cases (cMono, ncMono, and DC); hypoxia (cMono, DC), and IL2_STAT5 pathways (cMono) across severity groups; and complement coagulation and cholesterol metabolism in more severe disease in cMono (Figures S5E–S5G).

To investigate epigenetic correlates of the COVID-19 response, we analyzed chromatin accessibility by single-cell ATAC-seq (Figures 1A, S5H, and S5I; STAR Methods). Overall, 750 and 303 accessible sites were up- and downregulated, respectively, in COVID-19 patients compared to healthy volunteers in myeloid cells (Figure S5J). Genes linked to top differentially open chromatin peaks included *STK24* (MAPK promoting apoptosis) and *FGFRL1* (cell adhesion promoting fibroblast growth factor receptor) (Figures S5J and S5K). We identified the most significant DNA binding motif enrichments in the differentially accessible sites involved AP-1, SW1/SNF, and BACH transcription factor family members, which regulate chromatin remodeling and immunity (Figure 4E). Moreover, motif footprint analysis revealed increased accessibility of genomic regions containing FOS and JUN motifs in COVID-19 patients relative to healthy volunteers in myeloid cells, a signal which was also seen in convalescence (Figure 4F).

### COVID-19 severity correlates with specific T and NK cell populations and features relating to cell cycle, redox state, and exhaustion

**Figure S6:**
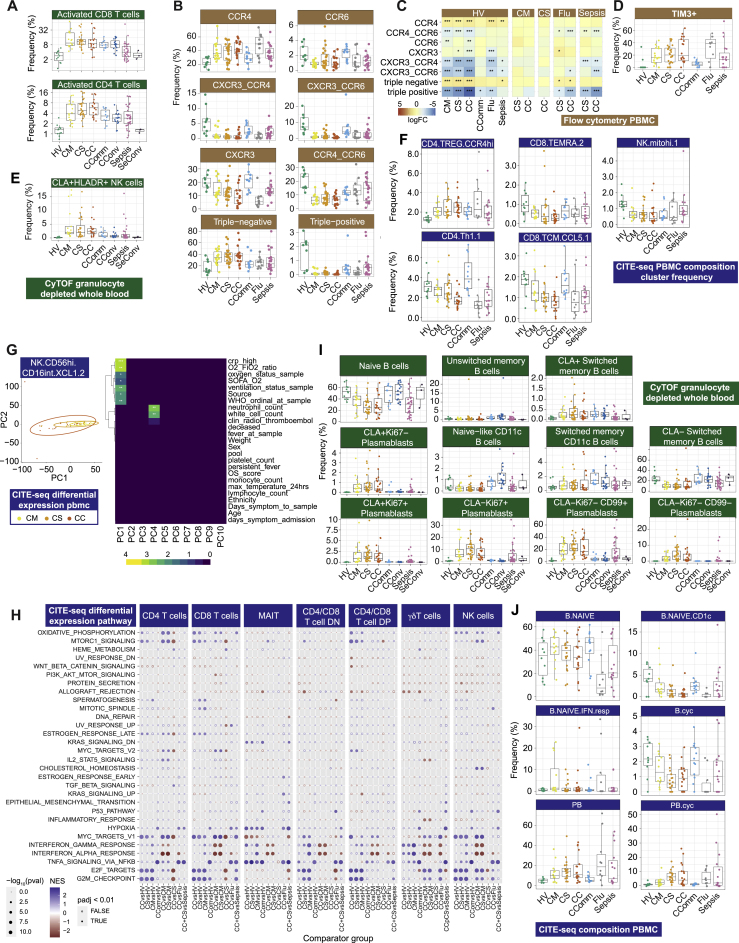
Dynamic changes in lymphocyte populations associated with COVID-19 severity, related to Figure 4 (A) Frequency of activated CD4 and CD8 T cells assayed by single cell mass cytometry. (B-D) Multicolor flow cytometry analysis of PBMC. (B,C) Boxplots, dotplots and heatmap describing the phenotype and frequency of subsets of memory CD4 T cell subsets defined based on the expression of CCR4, CCR6 and CXCR3. (D) Frequency of TIM3^+^CD38^+^HLADR^+^ CD8^+^ T cells. (E) Frequency of CLA^+^ HLADR^+^ NK cells assayed by single cell mass cytometry. (F-H) CITE-seq profiling CD4^+^ CD8^+^ T and NK cell clusters. (F) Frequency between comparator groups. (G) Principal Components Analysis (PCA) and correlation with clinical covariates and severity measures for gene expression in acute hospitalized cases (mild, severe, critical) in activated NK cells. (H) scRNA-seq MSigDB hallmark gene set enrichment for T cell populations. (I) Single cell mass cytometry composition analysis of B and plasmablast cell populations comparing study groups. (J) CITE-seq compositional differential abundance analysis of B and plasmablast cell clusters. All boxplots show median, first and third quartiles; whiskers show 1.5x interquartile range. Abbreviations for CITE-seq (panels F-H,J). B: B cell; cDC: classical dendritic cell; cyc: cycling; DN: CD4/CD8 double negative; DP: CD4/CD8: double positive; hi: high; IFN, interferon; int: intermediate; mito, mitochondrial; NK: natural killer cell; PB: plasmablast; PBMC: peripheral blood mononuclear cell; resp: responsive; TCM, T central memory; TEM(RA): T effector memory (CD45RA re-expressing); Th, T helper; TREG: T regulatory cell. Comparator group abbreviations. HV: healthy volunteer; CM: COVID-19 in-patient mild; CS: COVID-19 in-patient severe; CC: COVID-19 in-patient critical; CComm: COVID-19 community case in the recovery phase (never admitted to hospital); CConv: COVID-19 convalescence (survivors from 28 days after discharge); Flu: influenza in-patient critical; Sepsis: in-patient severe and critical sepsis; SeConv: sepsis convalescence.

We proceeded to further characterize T and NK cell populations using mass and multicolor flow cytometry (STAR Methods; Data S3). We found activated CD4^+^ and CD8^+^ T cells were increased in frequency in all COVID-19 patient groups and remained elevated in convalescence (Figures 4A and S6A). The proportion of CD27^+^ activated CD4^+^ T cells was higher than in sepsis, while the CD56^+^ cytotoxic CD8^+^ T cell frequency was reduced (Figure 4A). We investigated T cell subsets using markers of activation, proliferation, and exhaustion. While comparably expressed in activated CD4^+^ T cells across acute COVID-19 cases, these markers increased in CD8^+^ T cells with increasing disease severity (Figure 4G). We found differential chemokine receptor expression in the overall memory CD4^+^ T cell population (Figures S6B and S6C) and increased expression of the inhibitory receptor TIM3 in activated CD8^+^ T cells (Figure S6D). CLA^+^ HLADR^+^ NK cells were increased in all COVID-19 cases including convalescence (Figure S6E). We also found evidence of changes in innate-like lymphocytic cell populations with increasing COVID-19 severity, including mucosal associated invariant T (MAIT) cells, which showed a gradient of involvement across severity in terms of cell activation (higher % CD69^+^ MAIT cells in more severe disease, Figure 4H).

Complementing these findings, CITE-seq analysis (STAR Methods; Data S5) showed an increase in cycling and activated CD4^+^ and CD8^+^ T and NK cell populations in hospitalized COVID-19 cases, including CCR4^hi^ Tregs (Figures 4B and S6F). Conversely, we observed a decrease in CD4^+^ Th1, CCL5^+^ CD8^+^ T central memory, CD45RA^+^ CD8^+^ T effector memory, and NK cells with high mitochondrial gene expression. There was minimal compositional variation in these cell populations associated with severity or clinical covariates (Data S5). Analysis of gene expression in hospitalized COVID-19 patients showed the most significant clinical correlate involved activated NK cells (CD56^high^CD16^low^ *XCL1/2* expressing) where the largest component of variance was associated with WHO ordinal and oxygenation/ventilation status (Figure S6G). Across CD4^+^, CD8^+^, and NK cells, we found cell cycle and redox state pathways were enriched for differentially expressed genes in more severe hospitalized COVID-19 cases; interferon pathways in less severe disease; and TNF signaling in community cases versus healthy volunteers (Figure S6H). MAIT cells showed enrichment for TNF signaling and KRAS across COVID-19 groups and γδ T cells for cell cycle pathways (Figure S6H).

### Severe COVID-19 is associated with clonal expansion of unmutated B cells and activation of autoreactive B cells

We then investigated at high resolution how B cell populations vary in COVID-19. Mass cytometry (STAR Methods; Data S3) demonstrated significant lymphopenia in COVID-19 with reduced overall frequency and number of B cells, predominantly naive B cells, but an increase in terminally differentiated plasmablasts (significantly higher than in sepsis) and a relatively high proportion of CLA^+^ plasmablasts (Figures 1B, 4A, and S6I). We found the greatest increase in switched memory CD11c^+^ B cells in community COVID-19 cases, while unswitched memory B cells and naive CD11c^+^ B cells were higher in COVID-19 convalescent samples (Figure 4A). Analysis of CITE-seq-defined clusters (STAR Methods; Data S4) revealed increases in plasmablasts in severe disease; naive CD1c^+^ naive and cycling naive B cells were reduced in COVID-19, but overall, naive B cells were significantly more reduced in influenza than COVID-19 of comparable severity; and in mild hospitalized COVID-19, only the interferon-responsive naive B cell cluster showed an increase (Figures 4B and S6J).

**Figure 5:**
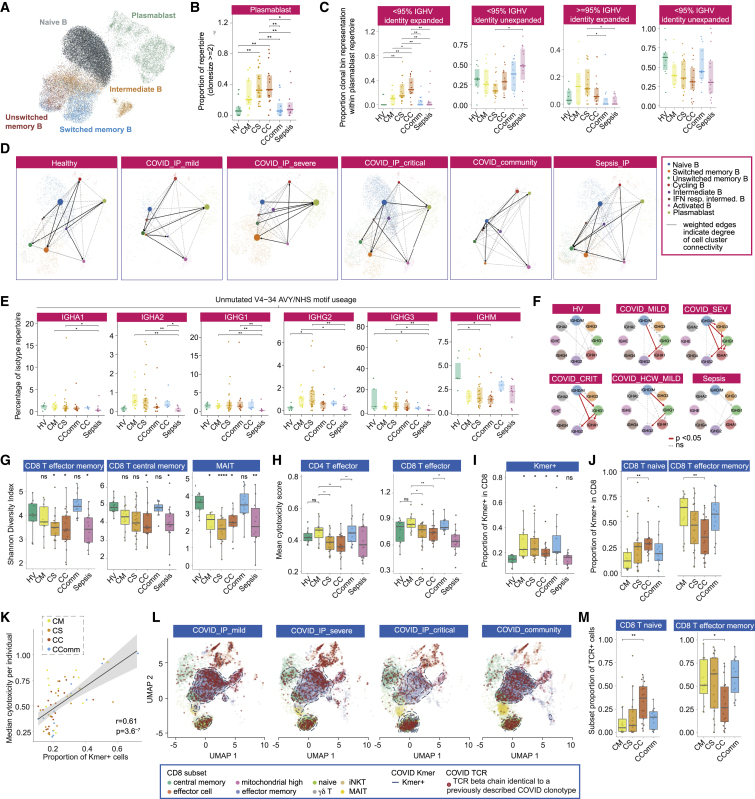
Differences in B and T cell repertoire associated with COVID-19 severity (A–F) B cells: (A) UMAP embedding with cluster identities from CITE-seq. (B) Plasmablast repertoire clonality. (C) Mutation and expansion proportions in plasmablast clone repertoire. (D) Partition-based graph abstraction plots of scRNA-seq by cell population and patient group. (E) IGHV4-34 AVY/NHS motif usage in unmutated VDJ sequences across IGH genes (bulk BCR-seq). (F) Class switch inference networks (RNA derived BRCs). Significance ^∗^ < 0.05, ^∗∗^ 0.005 Kruskal Wallis. (G–M) T cells: (G) Shannon Diversity Index for specific cell populations by comparator group. (H) Mean cytotoxicity score by comparator group. (I) Proportion of CD8^+^ T cells carrying TCR containing COVID-19 associated Kmers. (J) Frequency of COVID-19 Kmer positive cells in CD8^+^ naive and effector memory cells. (K) Correlation of COVID-19 Kmer containing CD8^+^ T cells per individual with median cytotoxicity score. (L) UMAP of CD8^+^ T cells by patient group indicating density of COVID-19 Kmer positive cells (blue dashed line) and cells with previously described COVID-19 clonotype. (M) Proportion of COVID-19 known clonotype matching cells in CD8^+^ naive and effector memory cells. Wilcoxon Test age and sample size adjusted linear model ^∗^p < 0.05, ^∗∗^p < 0.01, ^∗∗∗^p < 0.001. All boxplots show median, first and third quartiles; whiskers 1.5x interquartile range. See Figure S7.

**Figure S7:**
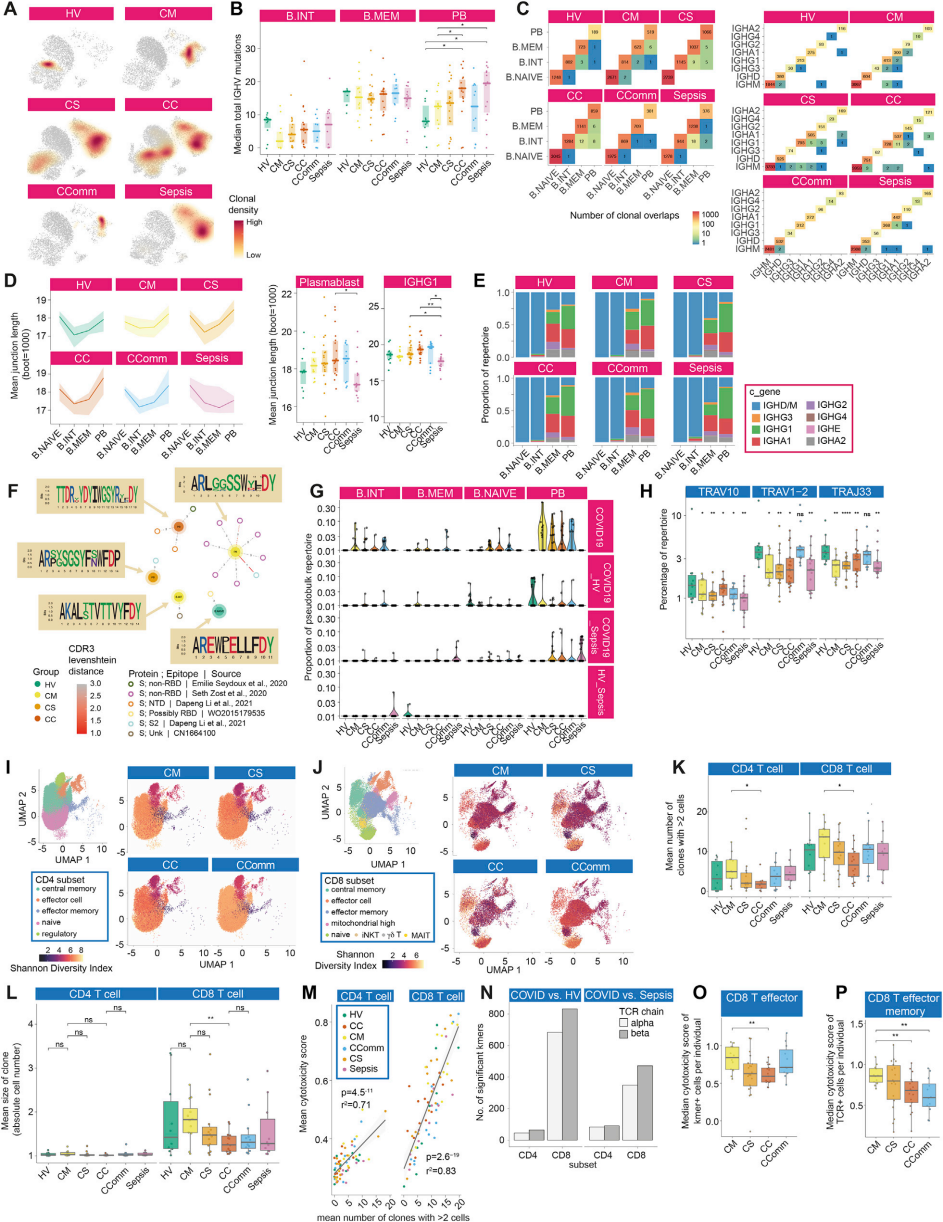
Differences in B and T cell repertoire associated with COVID-19 severity, related to Figure 5 (A-G) Analysis of B cell immune repertoire using bulk VDJ sequencing of whole blood (1,206,531 filtered BCR sequences analyzed) and single cells (CITE-seq). (A) Clonal density plots with Kernels density estimates overlaid onto UMAP embeddings by comparator group. (B) IGHV total mutations across B cell subsets per study group (naive B cells not shown, as no mutations). (C) Clonal overlaps across B cell clusters and across constant region genes per study group. Numbers reflect binary detection events mutation and expansion proportions in plasmablast clone repertoire. (D) Junction lengths from resampled repertoires by patient group per B cell cluster and in plasmablasts and in plasmablast immunoglobulin constant gene IGHG1. The line shows mean amino acid junction length; the ribbon range is the 0.25-0.75 quantiles of bootstrapped samplings. (E) Ig constant region genes per B cell cluster (single cell VDJ data). (F) Sequence similarity network of VDJ sequences, from single cell VDJ data (central nodes), to published monoclonal antibodies (peripheral nodes; references and epitopes described in legend). Edges depict pairwise Levenshtein’s distance of CDR3s. CDR3 sequence logos are shown following multiple sequence alignment. (G) The proportion of B cells across each B cell cluster per disease group of sequences shared between patient groups (observed in at least 2 patients). (H-P) Analysis of T cell immune repertoire. (H) TRAV and TRAJ repertoire analysis. (I,J) UMAP of CD4^+^ T cells (I) and CD8^+^ T cells (J) with associated clusters used in repertoire analysis indicating Shannon Diversity Index by patent group. For clusters used in repertoire analysis see Data S3. (K) Number of enlarged clones by comparator group in CD4^+^ and CD8^+^ subsets. (L) Mean clone size CD4 and CD8. (M) Using a pre-defined cytotoxicity metric the overall cytotoxicity was calculated per individual for both the CD4^+^ and CD8^+^ subsets. For each individual the number of enlarged clones in these subsets was determined (defined as > 2 cells with the same TCR chain). Mean cytotoxicity per individual is correlated with the number of expanded clones across each individual, irrespective of cohort origin (Pearson’s r^2^). For illustration of the method used to identify CDR3 Kmers associated with COVID-19 compared to cells from healthy volunteers and patients with sepsis see Data S3. (N) Number of Kmers comparing COVID-19 versus healthy volunteers and sepsis. (O) Cytotoxicity of CD8^+^ T effector cells positive for a COVID-19 associated Kmer across patient groups. (P) Cytotoxicity of CD8^+^ T effector memory cells with clonotypes matching published COVID-19 clonotypes. Comparator group abbreviations. HV: healthy volunteer; CM: COVID-19 in-patient mild; CS: COVID-19 in-patient severe; CC: COVID-19 in-patient critical; CComm: COVID-19 community case in the recovery phase (never admitted to hospital); Sepsis: in-patient severe and critical sepsis. Wilcoxon Test age and sample size adjusted linear model used ^∗^p < 0.05, ^∗∗^p < 0.01, ^∗∗∗^p < 0.001. All boxplots show median, first and third quartiles; whiskers show 1.5x interquartile range.

We then characterized the B cell immune repertoire using bulk VDJ sequencing of whole blood and CITE-seq (Figures 1A and 5A; STAR Methods; Data S3 and S4). In healthy volunteers, clonal expansions appeared to predominate within the memory B cell population, while in COVID-19 and sepsis patients, we found expansions in plasmablasts, with severe and notably critical COVID-19 patients also harboring clones within memory populations (Figure S7A). The clonal expansion in plasmablasts was statistically significant and also showed an association with COVID-19 severity, in contrast to sepsis where there was no significant change (Figure 5B).

Limited somatic hypermutation (SHM) of SARS-CoV-2 antibodies has been widely reported (Brouwer et al., 2020). We observed fewer somatic hypermutations in intermediate B cells in hospitalized mild COVID-19 but a severity-associated increase within plasmablasts, also seen in sepsis (Figure S7B). There were, however, COVID-19 specific differences in the proportion of expanded clones with few mutations (> 95% IGHV identity, Figure 5C). RNA velocity analysis suggested a differentiation directionality between naive B cells and plasmablasts in COVID-19 patients distinct from sepsis, and consistent with a predominant extrafollicular B cell response in COVID-19 (accumulating fewer SHMs) (Figure 5D). Moreover, we observed a higher number of shared clones between plasmablasts and intermediate or memory B cells in severe/critical COVID-19 patients, whereas sepsis patients exhibited higher clonal overlap between intermediate and memory B cells (Figure S7C).

These data together indicate substantial expansion of unmutated B cells associated with plasmablast populations in severe/critical COVID-19. We next explored differences in B cell selection and tolerance. First, in COVID-19 patients, we observed increased BCR complementarity-determining region 3 (CDR3) lengths compared to sepsis (Figure S7D); such increases have been associated with antibody polyreactivity and autoimmunity (Meffre et al., 2001). Second, we found multiple differentially utilized IGHV/J genes between COVID-19 groups indicating differential B cell selection and/or expansion of naive B cells, while the antigen experienced IgD/M mutated and class-switched B cell repertoire showed differentially utilized IGHV/J genes, revealing differential peripheral selection of B cells with increasing COVID-19 severity (Data S3). Third, we tested whether B cells targeting autoantigen and red blood cell antigen are associated with COVID-19. We found that autoreactive IGHV4-34 BCRs, which are elevated in autoimmunity (Pascual et al., 1991), were significantly depleted in IGHD/M but elevated in class-switched B cells, most notably for the IGHA2 and IGHG2 B cells (Figure 5E) consistent with class-switching of these autoreactive B cells during the response to SARS-CoV-2. In further support of this, the degree of class-switching, inferred from the BCR sequencing data (Bashford-Rogers et al., 2019), was significantly elevated between IgD/M and IgG1 and IgA1 and finally to IgG2 in COVID-19 patients (Figures 5F and S7E). No detectable differences in either IGHV4-34 autoreactive BCR levels or class-switching were observed in sepsis cases.

Previous reports indicate an unexpectedly high level of BCR convergence between unrelated COVID-19 patients (Galson et al., 2020). We also found clonal sharing within and between COVID-19 severity groups (Figures S7F and S7G; Data S3). Comparing to known receptor-binding domain antibodies, we observed that most highly similar patient BCRs have a plasmablast phenotype (Figure S7G). Overall, our data indicate that the plasmablast expansions in severe COVID-19 include high levels of broadly auto-reactive B cells, consistent with an emerging role for B cell driven immune pathology (Wang et al., 2021).

### Reduced diversity in CD8^+^ T cell populations on repertoire analysis

To further investigate the effect of disease on T cell subsets with reference to antigen recognition and clonality, we integrated TCR sequencing data performed across the same cell subsets. Given we saw clonotypes present across populations, we merged subsets to provide power for downstream clonal analysis (STAR Methods). For semi-invariant T cells, differences observed with severity and disease group by cell cluster were supported by consistent changes in TCR alpha variable (TRAV) gene usage. Hospitalized COVID-19 and sepsis cases displayed reductions in the percentage of repertoire occupied by *TRAV10*, specific to invariant NK T cells, and *TRAV1-2* and *TRAJ33* usage, in keeping with reductions in MAIT cells (Figure S7H).

To better understand the relationship between COVID-19 and T cell clonality, we calculated Shannon diversity indices across clones based on the TCRbeta chain, controlling for age. While CD4^+^ subsets showed higher diversity than CD8^+^ subsets, differences with disease severity were only seen in CD8^+^ T cells (Figures S7I and S7J; STAR Methods). Across disease states and accounting for age, CD8^+^ T effector memory (CD8.TEM/TEMRA), CD8^+^ T central memory (CD8.TCM/CD8.TCM.CCL5), and MAIT cell diversity were reduced in COVID-19 severe and critical disease with comparable changes in sepsis (Figure 5G).

Recent evidence suggests that effective CD8^+^ T cell responses involve increased numbers of expanded clones (Fairfax et al., 2020). Consistent with this, we found hospitalized COVID-19 patients with mild disease had higher numbers of expanded clones in both CD4^+^ and CD8^+^ subsets, and the mean clone size was higher within the CD8^+^ subset (Figures S7K and S7L). In keeping with the observation that expanded CD8 T cell clones show increased expression of cytotoxicity markers (Watson et al, 2020), using a composite gene score for cytotoxicity, we found that the number of expanded clones correlated with the average cytotoxicity score across all cells for that individual in both CD4^+^ T effector (CD4.TEFF/TEFF.prolif) and CD8^+^ T effector (CD8.TEFF/TEFF.prolif) populations (Figure S7M), and was higher in mild and community COVID-19 cases with reduced cytotoxicity observed in critical and severe disease (Figure 5H).

To further explore whether COVID-19 leads to generalized signatures of antigen presentation with reciprocal effects on TCR sequence and corresponding CDR3 usage, we devised an approach to identify COVID-19 associated amino acid sequences (Kmers of 4 amino acids) within the beta chain CDR3 region (STAR Methods). These were compared with chains from healthy volunteers and sepsis patients to exclude sequences non-specifically associated with infection (Data S3). We identified 125, 4-amino acid Kmers (referred to as COVSeqs) enriched in COVID-19 (*P*_*c*_ < 0.05 versus both groups), the vast majority in CD8^+^ T cells (Figure S7N), with the proportion of cells with TCRs containing at least one COVSeq in the beta chain specifically increased in all COVID-19 patients (Figure 5I). In hospitalized patients, we found a lower proportion of CD8^+^ T effector memory cells with COVSeq containing TCRs with increasing disease severity (Figure 5J). Critical disease was associated with naive CD8^+^ T cells containing COVSeqs, indicating failure of the SARS-CoV-2 reactive cells in critical patients to expand into the effector phenotype, or possibly a distinct redistribution of the expanded cells. Further supporting functionality of the COVID-19 Kmer containing cells, the proportion of COVSeq-containing cells was correlated with the median cytotoxicity of cells per individual among the COVID-19 patients (Figures 5K and S7N). Notably, COVSeq-positive CD8^+^ T effector cells from critical patients showed reduced cytotoxicity compared to mild disease (Figure S7O).

Finally, we addressed whether using previously published COVID-19 associated beta chain clonotypes could further resolve variation in the T cell response according to disease severity (STAR Methods). We observed many cells carrying such TCRs across the COVID-19 patients, often overlapping COVSeq-containing cells. Notably, the distribution of these cells across clusters varied markedly according to COVID-19 disease state (Figure 5L). Replicating the observations with COVSeq-positive cells, CD8^+^ T effector memory cells were relatively depleted for COVID-19 clonotypes in critical disease (Figures 5M and S7P).

### Correlates of severity and disease specificity in the COVID-19 plasma proteome involve acute phase proteins, metabolic processes, and markers of tissue injury

**Figure 6:**
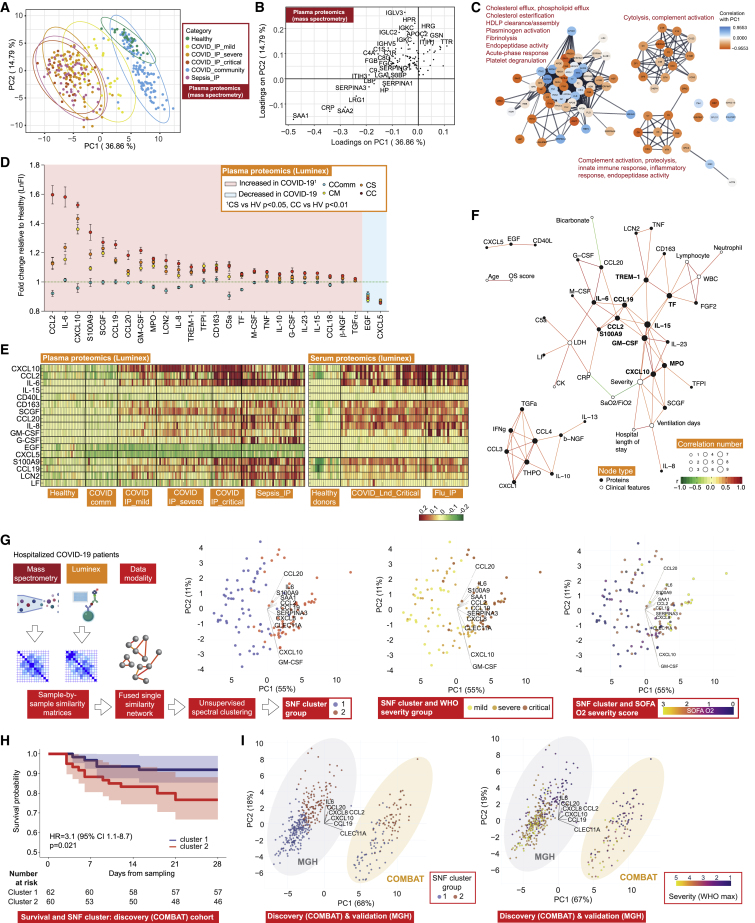
Plasma protein COVID-19 signatures and sub-phenotypes (A–C) HT-LC-MS/MS mass spectrometry of plasma proteins. (A) Principal components analysis (PCA) of all samples. (B) Proteins contributing to PC loadings (more negative loading values indicating higher positive correlation with disease severity). (C) Clusters based on protein-protein interaction network with enriched GOBP terms. (D–F) Proteins significantly differentially expressed between comparator groups assayed by Luminex. (D) Fold change in plasma proteins in hospitalized COVID-19 versus healthy volunteers. Data represented as mean ± SEM. (E) Plasma and serum protein abundance by comparator group. (F) Network of clinical feature−protein correlations in COVID−19 patients and healthy volunteers based on highly correlated events (r^2^ > 0.7 or < -0.5). (G) Similarity network fusion (SNF) using plasma proteins for hospitalized COVID-19 patients from COMBAT cohort showing approach and PCA colored by cluster (left) or WHO severity group (middle) or SOFA O_2_ score (right). (H) Kaplan-Meier survival plot by SNF cluster group (95% CIs shaded) (HR, hazard ratio calculated using Cox proportional hazard model). (I) Mass General Hospital (Olink) validation data and COMBAT (discovery) cohorts showing cluster groups (left) or colored by WHO max severity (right). See Figure S8.

We aimed to complement our multimodal cellular profiling with analysis of the COVID-19 plasma proteome. To do this, we performed high-throughput liquid chromatography with tandem mass spectrometry (LC-MS-MS), presenting data for 105 proteins on 257 individuals (340 samples) (Figure 1A; STAR Methods; Data S3). We found differences by severity and etiology by analyzing principal components of variance (Figure 6A) and on unsupervised hierarchical clustering and supervised correlation analysis (Data S3). Severe disease, reflected in variance component loadings, was associated with increased acute-phase proteins and complement system proteins, including recognized biomarkers of inflammation (SAA1, SAA2, and CRP), complement membrane attack complex components (C5, C6, C9, and CFB), and functionally related protein families such as protease inhibitors (SERPINA3, SERPINA1, and ITIH3) and serum amyloid P-component (APCS) (Figure 6B; Data S3). We also found differential protein abundance involving markers of tissue injury and necrosis, notably reduced extracellular actin scavenger plasma gelsolin (GSN); increased fibrinogens (FGA, FGB, and FGG); and an increase in proteins implicated in IL-6 mediated inflammation (LGALS3BP, LRG1, LBP, HP, and ITIH4). We further identified protein clusters based on the protein-protein interaction network, including a large cluster enriched for biological processes involving cholesterol transport and fibrin blood clots within which individual proteins showed positive and negative correlations with disease severity (PC1). Two smaller clusters enriched for cytolysis and complement activation positively correlated with disease severity (both showing negative correlations for all constituent proteins with PC1) (Figure 6C).

**Figure S8:**
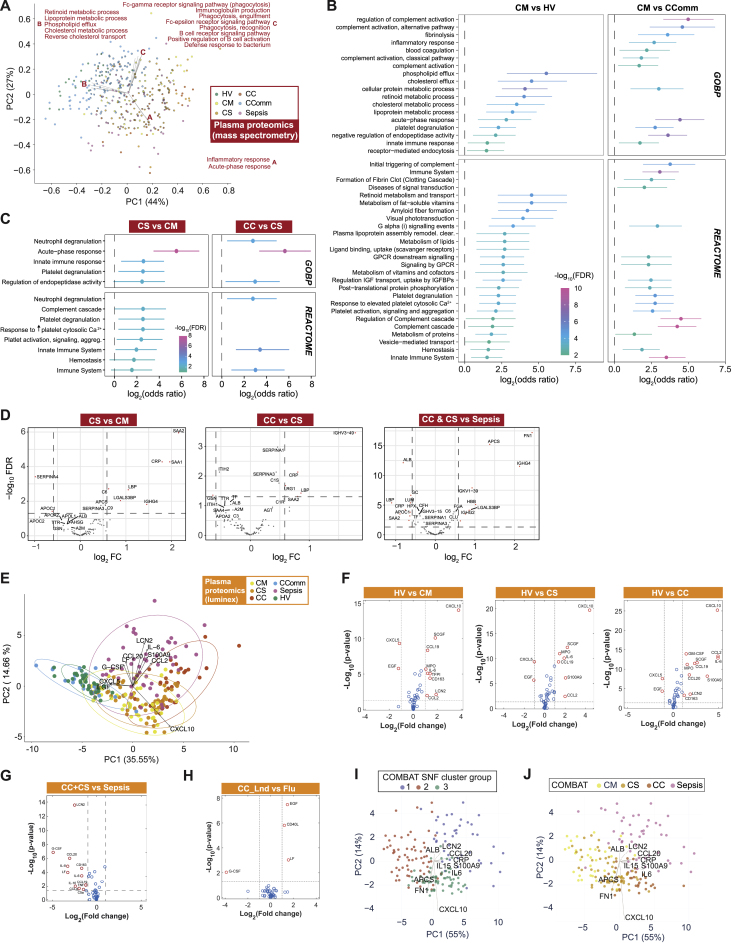
Plasma protein signatures and sub-phenotypes of COVID-19, related to Figure 6 (A-D) Plasma proteins assayed by HT-LC-MS/MS mass spectrometry. (A) Functional principal components analysis (PCA) in which a vector of biological process enrichment scores is generated from single-sample Gene Set Enrichment Analysis (ssGSEA) derived from ranked intensities of the identified proteins. (B,C) GOBP terms or Reactome pathways significantly enriched (FDR < 0.05) in proteins differentially abundant contrasting samples from (B) mild hospitalized COVID-19 patients with those from healthy volunteers or from mild community COVID-19 cases and (C) severe versus mild or critical. Bars indicate 95% confidence intervals. (D) Pairwise contrasts, severe versus mild, critical versus severe COVID-19, COVID-19 severe or critical versus sepsis for plasma proteins assayed. (E-H) Luminex blood proteins. (E) PCA of all plasma samples. (F-H) Volcano plots comparing differential abundance of plasma proteins for (F) COVID-19 severity groups versus healthy volunteers, (G) critical/severe COVID-19 versus sepsis, (H) critical COVID-19 versus influenza. (I,J) Similarity network fusion (SNF) when analyzing hospitalized COVID-19 and sepsis patients shaded by (I) cluster group and (J) patient comparator group. Comparator group abbreviations. HV: healthy volunteer; CM: COVID-19 in-patient mild; CS: COVID-19 in-patient severe; CC: COVID-19 in-patient critical; CC_Lnd: COVID-19 in-patient critical (London); CComm: COVID-19 community case in the recovery phase (never admitted to hospital); Sepsis: in-patient severe and critical sepsis.

We found the main processes associated with differences between samples were acute-phase response and inflammation, metabolic (retinoid and lipoprotein), and cholesterol transport (Figure S8A). Reduced levels of proteins associated with lipoprotein and cholesterol metabolism included apolipoproteins A-I, A-II, C-I, and C-II (APOA1/2 and APOC1/2) and transthyretin (TTR), consistent with their downregulation in systemic inflammation and differences in metabolic state specifically associated with disease severity. This was further evident on pairwise comparisons, with mild hospitalized COVID-19 patients differing from healthy volunteers in metabolic processes and vesicle transport of retinoid, cholesterol, lipoproteins, and fat-soluble vitamins; and from community cases by higher levels of complement activation and coagulation (Figure S8B). Severe COVID-19 patients differed from mild and from critically ill patients in processes relating to platelet degranulation and neutrophil degranulation respectively (Figures S8C and S8D). When we compared severe and critical COVID-19 with sepsis, 19 out of 105 proteins showed changes specific to COVID-19 (FDR < 0.05, FC > 1.5), enriched in acute-phase response, complement activation, and receptor-mediated endocytosis (Figure S8D).

### Plasma cytokine and chemokine profiling shows evidence for involvement of inflammatory mediators

To characterize inflammatory mediators of the response to SARS-CoV-2, we analyzed 51 circulating cytokine and chemokine proteins using the Luminex assay for 171 individuals (Figure 1A; STAR Methods; Data S3). There was clear clustering of hospitalized COVID-19 cases by severity on analysis of principal components of variance, while community cases overlapped with heathy controls and sepsis cases clustered separately (Figure S8E). The major proteins contributing to these axes of variance between groups were CXCL10, CXCL5, EGF, CCL2, S100A9, IL6, LCN2, CCL20, LF, and G-CSF (Figure S8E). Overall, we found 49% (25 of 51) analytes were differentially abundant in plasma from COVID-19 cases versus healthy volunteers (Figures 6D, S8F, and S8G; Data S3). Among these, CCL2, CCL19, CCL20, CXCL10, GM-CSF, IL-6, IL-8, IL-15, S100A9, and SCGF (all increased abundance) were strongly correlated with severity in hospitalized COVID-19 patients (r^2^ > 0.5, p < 0.001).

We further compared with sepsis and influenza to investigate disease specificity and found the plasma levels of G-CSF, IL-8, LF, CD163, LCN2, CCL20, IL-6, IL-10, CCL4, CCL19, TNF, and C5a were lower in critical and severe COVID-19 than sepsis (Figures 6E and S8G). Compared with influenza, serum EGF, LF, and CD40L were higher in serum from patients with critical COVID-19, while G-CSF was lower (Figure S8H). We then investigated protein-protein correlation network relationships of assayed plasma cytokines and chemokines. This identified S100A9, M-CSF, and CCL2/19 as nodal proteins. When we performed protein-clinical trait correlation network analysis for COVID-19 severity, we found strong correlations (|r| > 0.5) between clinical features (CRP, SaO_2_/FiO_2_, and ventilation days) and specific nodal proteins (GM-CSF, CXCL10, TREM-1, CCL2/19, TF, IL-6/15, MPO, and S100A9) at the center of the network (Figure 6F).

### Plasma proteome variation identifies patient sub-phenotypes of differing disease severity

We next investigated the utility of plasma proteins for patient sub-phenotyping within hospitalized COVID-19 cases (n = 122 samples) by integrating the LC-MS-MS and Luminex datasets using Similarity Network Fusion (SNF) (Wang et al., 2014) (Figure 6G; STAR Methods). We first constructed a sample-by-sample similarity matrix from which we derived a network for each of the two data types. Analyzing these individually in an unsupervised manner with spectral clustering, we could only discriminate a minority of cases (the most mild from all others). However, when we fused these networks into a single similarity network that maximized shared and complementary information, we discovered two clusters that separated by clinical measures of disease severity including inspired oxygen concentration and SOFA oxygen score (t test *P*_*c*_ < 0.05) (Figure 6G). Notably, this molecular classification stratified severe cases assigned based on WHO categorical criteria into those that group with more mild cases and those clustering with critical cases. We identified 11 proteins as the main discriminatory features distinguishing the clusters (mutual information score ≥ 0.15) (Figure 6G). The predictive protein set spanned key inflammatory mediators, including the cytokines and chemokines IL-6, IL-8 (CXCL8), CCL2, CCL19, CCL20, and CXCL10 together with S100A9 (calprotectin), the acute phase proteins serum amyloid protein (SAA1) and protease inhibitor (SERPINA3), GM-CSF, and the C-type lectin CLEC11A. When we compared the two clusters, we found that membership of cluster two was associated with higher 28-day mortality (Figure 6H).

We validated the clusters in an independent acute hospitalized COVID-19 cohort assayed using a different technology, targeted proteomics by Olink (Filbin et al., 2021) (STAR Methods). Clustering analysis, using 7 of the 11 predictive proteins for which data were available, identified two optimal clusters (Figures 6I). These showed a clear relationship with measures of disease severity, including WHO ordinal score (maximum) (Figure 6I), and patients in cluster 1 had lower mortality at 28 days (5/164 = 3.0%) compared with cluster 2 (33/105 = 31.4%) (Chi-square test p < 0.0001), validating the findings from our discovery cohort. We extended the approach to include a combination of hospitalized COVID-19 and sepsis patients from COMBAT. This revealed three clusters, two corresponding to the clusters seen with COVID-19 cases analyzed alone, indicating a high level of specificity (Figures S8I and S8J). Features that separated COVID-19 and sepsis included lipocalin2 (LCN2) and CCL20, which were elevated in sepsis, and CXCL10, APCS, and fibronectin (FN1), which were higher in COVID-19.

### Supervised machine learning identifies predictive protein biomarkers for disease severity

**Figure S9:**
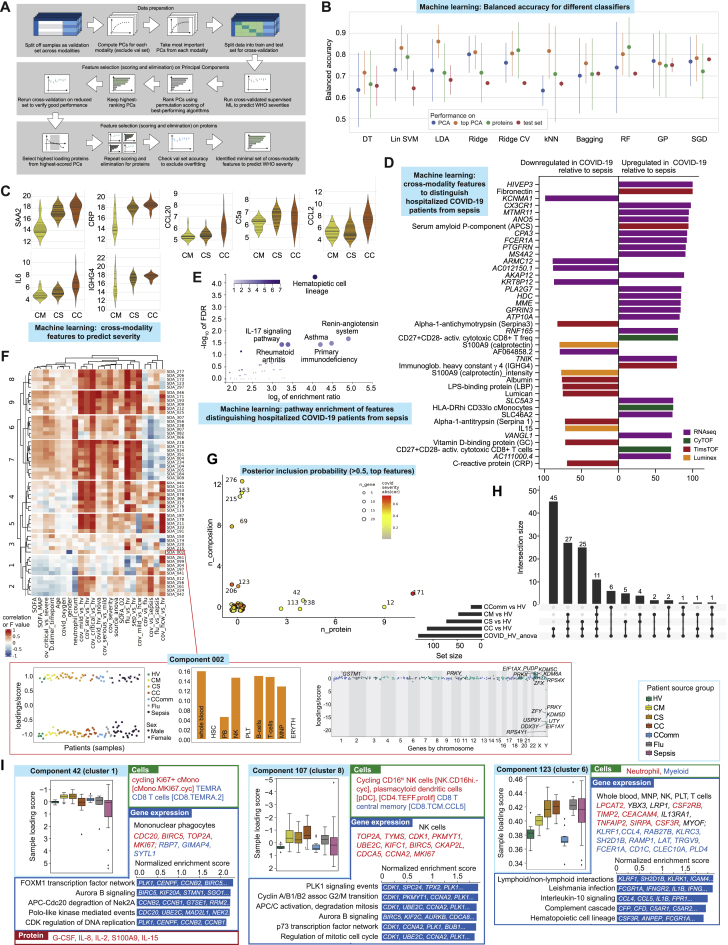
Integrative approaches define hallmarks of COVID-19 response, see Figure 7 (A-E) Machine learning feature selection for COVID-19 severity. (A) Summary of process followed. (B) Performance of the 10 best algorithms when run on all PCs, only the top-scored PCs, and the raw features extracted from the PCs (plot shows the mean balanced accuracy ± one standard deviation). We also show the accuracies from training the algorithms with the train+test sets and evaluating them on the validation set (averaged over 50 runs). (C) Violin plots showing distribution of final selected predictive feature set across WHO severity groups (horizontal lines in violin plots correspond to individual data points). Comparator group abbreviations. CM: COVID-19 in-patient mild; CS: COVID-19 in-patient severe; CC: COVID-19 in-patient critical. (D,E) Machine learning to discriminate between sepsis and COVID-19 using plasma proteins, whole blood total RNA-seq and mass cytometry as input variables in SIMON showing (D) discriminating features with variable score > 70 (E) enriched KEGG pathways on all features with variable importance score > 50. (F-I) Tensor and matrix decomposition across multi-omic datasets showing datasets including 152 samples by 8 cell lineage clusters (scRNA-seq, 22 missing samples) and whole blood (total RNA-seq, 9 missing samples) by 14,989 genes; cell composition from CITE-seq (152 samples by 64 pseudobulk cell types, 22 missing samples) and CyTOF (152 samples by 10 or 51 cell types, non-granulocyte depleted and depleted whole blood with 21 or 20 samples missing); and plasma proteins from Luminex (152 samples by 51 proteins, 20 missing samples) and high throughput liquid chromatography with tandem mass spectrometry (152 samples by 105 proteins with 17 samples missing). (F) Heatmap summarizing top components identified on pairwise contrasts involving clinical covariates, measures of severity and patient group with detail of tensor component 2 displayed for loading scores and relationship with gender, differential gene expression cell lineage clusters and whole blood. (G) Feature types contributing to loading scores of the top components according to the posterior inclusion probability. (H) Component inclusion where significant on analysis of variance between COVID-19 source group and healthy volunteers. BH adjusted p < 0.01 and absolute spearman’s p > = 0.5 (and BH adjusted p < 0.01) with at least one of the contrasts between the COVID-19 groups versus healthy volunteers. (I) Examples of components showing component number and cluster membership: sample loading scores across comparator groups and features (cells, gene expression, proteins) whose variance contributes to that component are shown; for gene expression, cell type and highest scoring genes listed (red upregulated, blue downregulated) together with top pathway enrichment (FDR < 0.05) with pathway genes listed within bars (features shown or included in pathway analysis where posterior inclusion probability > 0.5). Boxplots show median, first and third quartiles; whiskers show 1.5x interquartile range. Comparator group abbreviations. HV: healthy volunteer; CM: COVID-19 in-patient mild; CS: COVID-19 in-patient severe; CC: COVID-19 in-patient critical; CComm: COVID-19 community case in the recovery phase (never admitted to hospital); Flu: influenza in-patient critical; Sepsis: in-patient severe and critical sepsis.

**Figure 7:**
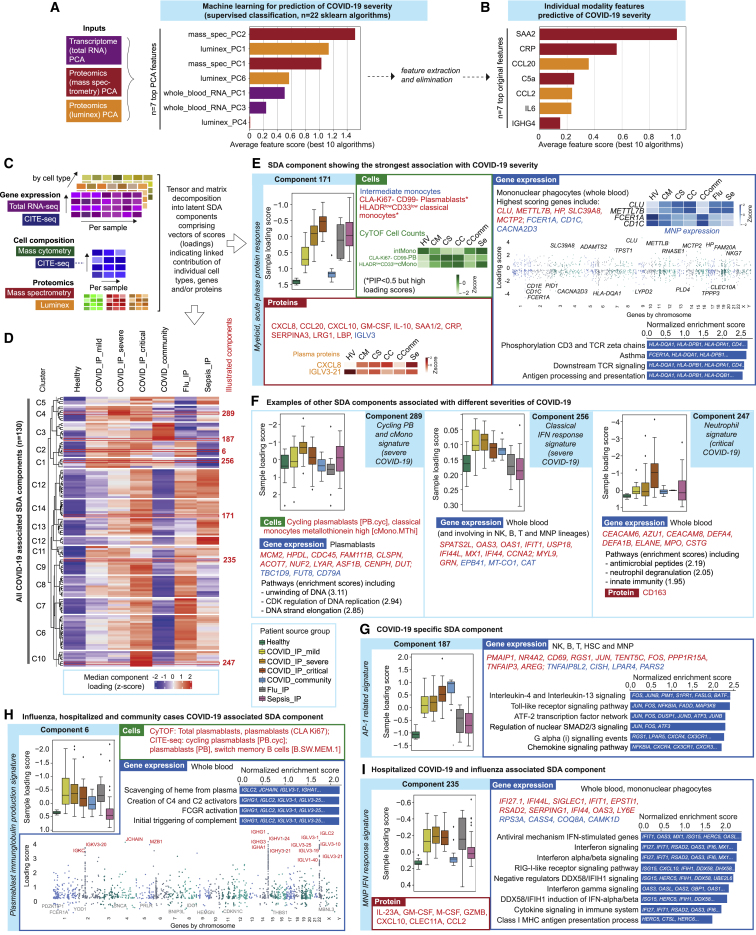
Integrative approaches define hallmarks of COVID-19 response (A and B) Machine learning for COVID-19 severity showing average feature score of (A) highest-scoring features (principal components, PCs), and (B) final feature set. (C–I) Tensor and matrix decomposition across multi-omic datasets for 152 samples showing (C) approach; (D) clustering of COVID-19 associated components (k-means clustering of row-scaled median sample loadings) and relationship with disease comparator groups; and (E–I) examples of components with sample loading scores differing by comparator group showing features (cells, gene expression, proteins) with high posterior inclusion probability whose variance contributes to that component; for gene expression, cell type and highest scoring genes listed (red upregulated, blue downregulated) together with top pathway enrichment (FDR < 0.05) with pathway genes listed within bars (features shown or included in pathway analysis where posterior inclusion probability > 0.5). (E) Component showing strongest association with COVID-19 severity. (F) Components associated with different severities of COVID-19. (G) COVID-19 specific component. (H) Influenza and COVID-19 associated component. (I) Hospitalized COVID-19 and influenza associated component. All boxplots show median, first, and third quartiles; whiskers 1.5x interquartile range. See Figure S9.

We next used machine learning to combine the two proteomics data types with whole blood total RNA-seq to determine which features were predictive of disease severity and their relative informativeness (Figure S9A; STAR Methods). We first identified assay-type-specific informative components of variance to reduce dimensionality for a training sample set and then determined which were most informative and the genes/proteins maximally contributing to each (Figure 7A). After feature elimination based on performance, we found the minimal set of cross-modality features to predict severity were the acute phase proteins SAA2 and CRP, an immunoglobulin (IGHG4), chemokines (CCL20 and CCL2), IL-6, and complement component C5a; the combined performance of these features in the hold-out validation set showed a balanced accuracy of 75%–80% to predict WHO category group (Figures 7B, S9B, and S9C). We also used machine learning to search for features that distinguish hospitalized COVID-19 patients from sepsis. A multi-omic set of 81 features was discovered using SIMON (Tomic et al., 2021) (STAR Methods) (AUC = 0.85, 95% CI = 0.59–1), identifying specific differentially abundant genes, proteins (including FCN1 and APCS as higher in COVID-19) and significant pathway enrichment for hematopoietic cell lineage and the renin-angiotensin system (Figures S9D and S9E).

### Integrated hallmarks of COVID-19 severity and specificity

We sought to dissect the immune response to COVID-19 across all assay types using a multi-omics tensor approach (Chang et al., 2021; Fanaee-T and Thoresen, 2019; Taguchi, 2017), specifically the sparse decomposition of arrays (SDA) algorithm (Hore et al., 2016). We analyzed 152 samples assayed for cellular composition, gene expression, and plasma proteomics, and found 381 latent SDA components, each comprising vectors of scores (loadings) that indicate the contribution of individual cell types, genes, or proteins linked by that component, and thereby offering insights into shared mechanism (Figures 7C and 7D; Table S4; STAR Methods). We identified components associated with specific clinical covariates, severity, or patient group, noting that while in some instances e.g., gender, there was a single associated component, typically several components were associated (Figure S9F). The strongest association with COVID-19 severity was for component 171 (*P*_*c*_ = 5.9x10^−14^, rho = 0.74 Spearman) (Figure 7E) which was unusual in having a high feature contribution from plasma proteins, whereas gene expression contributed most to the majority of the other components (Figure S9G). Contributing features to component 171 included raised plasma chemokines involved in chemotaxis and activation (CXCL8, CXCL10, and CCL20) and GM-CSF together with acute phase activating proteins (SAA1/2 and SERPINA3), LRG1, and LBP; reduced abundance of intermediate monocytes; high expression of cell stress chaperone *CLU* and methyltransferase *METTL7B*, and downregulation of IgE receptor and multiple HLA class II genes; and pathway enrichment for antigen presentation, TCR signaling, and asthma (Figure 7E).

To further delineate COVID-19 associated SDA components, we performed pairwise contrasts and analysis of variance involving COVID-19 patient groups. Overall, 130 of 381 components were associated with COVID-19 versus healthy volunteers (Figures 7D and S9H). Components associated with mild and severe but not critical disease included component 256 (upregulation of interferon response genes and downregulation of genes such as catalase and cytochrome *c* oxidase) which was specific to COVID-19 cases (Figure 7F); and component 42 (features of monocyte/granulocyte proliferation and function, elevated plasma proteins G-CSF, IL-2, IL-8, and IL-15, and enrichment of cell division related pathways) (Figure S9I). A further component strongly associated with severe disease involved plasmablast proliferation, combined with increased MT^hi^ cMono and a clear DNA replication signature (component 289) (Figure 7F). We found an innate response component specific to critical COVID-19 (component 247) with differential expression of granulocyte activation marker (*CEACAM8),* neutrophil elastase (*ELANE*) and defensins (*DEFA1B/4*), and increased soluble CD163 scavenger protein levels, reflected in pathway enrichment for neutrophil functions (Figure 7F). Neutrophil related features were also found in component 123 associated with COVID-19 severity, influenza, and sepsis (Figure S9I).

This approach also identified a further COVID-19 specific component (187) with high loading scores in hospitalized and community COVID-19 patients across NK, B, and T cells (Figure 7G). This was driven by upregulation of key stress and activation response genes including immediate early response protein (*PMAIP1*), AP-1 transcription factor genes *FOS* and *JUN*, the early activation marker, tissue residency and metabolic reprogramming gene *CD69,* and *TNFAIP3*, which limits NFkB mediated inflammation. The cytokine-induced STAT inhibitor (*CISH*) and immune checkpoint regulator of inflammation and metabolism *TNFAIP8L2* were downregulated. Pathway enrichment was seen for type-2 inflammation (IL4 and IL13), TLR signaling, and the ATF-2 network. We additionally identified COVID-19 and influenza-associated components (Figures 7H and 7I) including widespread upregulation of immunoglobulin heavy/kappa/lambda genes, *JCHAIN* (regulating multimerization and mucosal secretion of IgM/IgA), and *MZB1* (involved in antibody secretion and integrin-mediated cell adhesion) linking with possible antibody-dependent cellular toxicity (component 6); and significant upregulation of interferon pathway genes (component 235).

Overall, the latent component analysis identifies hallmarks of COVID-19 severity and specificity with respect to sepsis and influenza. Our findings highlight key cellular populations such as proliferating monocytes and plasmablasts, and features of innate and adaptive mechanisms ranging from interferon signaling to myelopoiesis, immunoglobulin production, and stress activation response signaling. The results prioritize and validate hallmarks seen on individual modality analysis such as AP-1 and generate hypotheses for how hallmarks may be related in terms of pathophysiology based on co-occurrence in a given component.

## Discussion

Our comprehensive multimodal integrated approach, applied to multiple well-defined cohorts, has identified blood hallmarks of COVID-19 severity and specificity involving particular immune cell populations and their development, components of innate and adaptive immunity, and connectivity with the inflammatory response (graphical abstract).

Identified hallmarks of severity involving myeloid related features include emergency myelopoiesis, immature neutrophils, increased HSC, and platelet/CD34^−^ megakaryocyte progenitors, with the latter associated with thromboembolism. These findings substantiate and add granularity to previous reports (Bernardes et al., 2020; Stephenson et al., 2021). We find evidence *ERG* is central to a gene network linked to these populations, encoding a transcription factor important in determining lineage plasticity, modulating inflammation, and maintaining an anti-thrombotic environment (Yuan et al., 2009). We further identify hallmarks supporting the importance of mononuclear phagocyte dysfunction in severe disease (Bost et al., 2021; Mann et al., 2020; Schulte-Schrepping et al., 2020), namely proliferating cMono, and specific monocyte populations showing reduced HLA-DR, CD33, and CD11c expression, high expression of antioxidant metallothionein and S100A8/9/12 (calprotectin), together with reduced pDCs.

Changes in the frequency of specific T cell subsets, and their activation and exhaustion, has been previously reported in severe COVID-19 (Chen and John Wherry, 2020; Jouan et al., 2020; Parrot et al., 2020). We found increased numbers of activated CD8^+^ T cells and NK cell populations in COVID-19, and, with increasing severity, failure of clonal expansion in CD8^+^ T effector and central memory cells and depletion of COVID-19 clonotypes. We further found association with severity for exhaustion markers and specific activated NK and CD69^+^ MAIT cell populations. In terms of adaptive immunity (Brouwer et al., 2020; Galson et al., 2020), we find increased numbers of terminally differentiated plasmablasts, with expansion of unmutated B cells differing in selection and tolerance, and a higher proportion of clonally related B cells. Persisting changes in cell composition were found in convalescent samples, including an increase in activated CD4^+^ and CD8^+^ T cells and unswitched memory B cells as well as reduced CD16^−^CD56^dim^ NK cells. These differences were also seen in community cases when compared to healthy volunteers; upregulation of TNF signaling was a consistent feature across cell populations in community cases. Redox state and cell cycle associate with more severe disease across cell populations. Our data are also consistent with the importance of the hyperinflammatory state (Moore and June, 2020) and interferon response (Hadjadj et al., 2020; Lei et al., 2020) but as features of less critical disease and earlier phase of illness.

Our proteomic analysis identified plasma levels of specific cytokines and chemokines as biomarkers of severe disease with evidence for acute phase response, complement activation/attack, fibrin clots, proteases, serum amyloid, tissue necrosis, receptor mediated endocytosis, and cholesterol transport as hallmarks. Moreover, we discovered plasma protein signatures that can be used to stratify acute hospitalized COVID-19 cases into disease sub-phenotypes not identified from WHO categorical criteria, with cluster membership informative for response state and associated with differential 28-day mortality. We validated this in an independent dataset using a predictive set of seven plasma proteins (cytokines IL-6, IL-8; chemokines CCL2, CCL19, CCL20, CXCL10; and C-type lectin CLEC11A, a key growth factor for primitive hematopoietic progenitor cells). Patient stratification is important given the observed clinical heterogeneity within severe COVID-19. Such variability has historically confounded clinical trials for targeted immune therapy in other severe infections (Davies et al., 2018; Marshall, 2014).

This work has demonstrated the informativeness of a multi-linear tensor approach to stratify and interpret the complexity of multi-omic datasets. Application of the SDA algorithm identified latent components of variance that linked signals from across cellular, gene expression and plasma protein measurements. For example, the most significant tensor component associated with COVID-19 severity involves myeloid chemotaxis and activation, the acute phase response, HLA class II downregulation, and TCR signaling. Our dataset provides an important resource to develop other approaches for identifying multimodal signals and associated mechanistic insights, such as leveraging algebraic systems biology (Gross et al., 2016), multi-layer networks (Kivela et al., 2014), topological data analysis (Cámara, 2017), or tensor clustering (Seigal et al., 2019).

Considering features specific to COVID-19, we found the CD49d:CD43 ratio is elevated across COVID-19 patients, including into recovery and in convalescence, suggesting this may be an informative index score for neutrophil activities specific to COVID-19 that could be prognostically useful acutely and in convalescence. A subset of tissue-infiltrating CD49d expressing neutrophils have been reported, activated by vascular endothelial growth factor A released from hypoxic sites (Massena et al., 2015), while reduction in CD43 expression leads to neutrophil retention in the blood and increased adherence to vessel walls (Woodman et al., 1998). This raises the possibility of a link to the enhanced thrombosis observed in COVID-19 patients, the basis of which remains unresolved (Ortega-Paz et al., 2021).

Our epigenetic, gene expression, and integrative SDA analyses all identified upregulation of the AP-1 p38 MAPK pathway as a specific feature of COVID-19 across different immune subsets. While not associated with severity, this observation further supports systemic immune activation and proliferation as a hallmark of COVID-19. AP-1 is a pioneer transcription factor that reversibly imprints the senescence enhancer landscape following stress and can be modulated to reverse T cell exhaustion (Lynn et al., 2019; Martínez-Zamudio et al., 2020). This raises the possibility of AP-1 acting as a mediator of inappropriate chromatin remodeling and cellular activation/senescence in multiple cell types, with evidence from our analysis of persisting differential chromatin accessibility, gene expression signatures, and cell protein markers of activation that may contribute to both acute disease and post-COVID-19 syndrome. Our findings are consistent with work involving convalescent COVID-19 patients showing evidence of trained immunity and persisting chromatin remodeling in myeloid and lymphoid cell populations involving high levels of activity of AP-1 transcription factors (You et al., 2021). Persistent reduced chromatin accessibility in myeloid cells at AP-1 targeted loci has been reported following influenza vaccination associated with innate immune refractoriness (Wimmers et al., 2021). A role for AP-1 in COVID-19 is also consistent with the reported efficacy of baricitinib improving recovery of hospitalized patients (Kalil et al., 2021). Baricitinib acts upstream of AP-1 (Zarrin et al., 2021) and controls macrophage inflammation and neutrophil recruitment in COVID-19 (Hoang et al., 2021).

Our findings involving immune activation and proliferation are of further relevance given the key nodal plasma cytokines, including GM-CSF and IL-6, we identify as hallmarks of disease severity in proteomic and integrative analysis, targeting of which could also benefit dysfunctional granulopoiesis and neutrophil subsets. This supports the efficacy of tocilizumab (IL-6 receptor inhibitor) in severe disease (Horby et al., 2021b). Moreover, we find upregulation of *IL6R* in more severe disease. Our data support inhibitors of GM-CSF in current clinical trials, use of anti-CCL2 (Gracia-Hernandez et al., 2020), the repurposing of immune checkpoint inhibitors (Pezeshki and Rezaei, 2021) to restore/enhance CD8^+^ T cell cytotoxicity, and potential targets such as FGFRs, cofactors for early-stage viral infection upregulated by MERS-CoV2 leading to lung damage (Hondermarck et al., 2020).

Establishing optimal biomarkers of response and relevant sample collection, as well as timely availability of results, are important considerations in current immunomodulatory trial design in COVID-19. We found the widely used clinical biomarker CRP has predictive utility but less than serum amyloid A and is one component of a discriminating biomarker marker set for severity. In terms of multi-omics, total RNA-seq of whole blood stabilized at the bedside, a tractable sample type for collection in a pandemic, was highly informative for understanding variance in disease severity while use of small volumes of rapidly fixed whole blood for later flow and mass cytometry gives complementary and highly granular resolution of the cellular immune response state and identifies specific cell populations important in severe disease.

An important question is how the hallmarks of severe disease in COVID-19 relate to non-SARS-CoV-2 sepsis (Beltrán-Garcia et al., 2020; Olwal et al., 2021). We compared patients with severe or critical illness, not only revealing shared features relating to emergency myelopoiesis and progenitors but also identifying discriminating neutrophil markers (CD49d:CD43 ratio) in COVID-19 (summarized in graphical abstract). In terms of monocytes, upregulation of cMono and specifically higher abundance of CD14^+^CD33^+^HLADR^−^ cells and S100A8/9/12 expressing cMono in severe/critical COVID-19 and sepsis is consistent with similar changes in monocytic myeloid-derived suppressor cells in both diseases, while similar reductions in ncMono and DCs were seen in both COVID-19 and sepsis. We found specific differences for other populations, for example increased complement expressing (C1Q) ncMono in sepsis and extracellular matrix protein versican expressing cMono (mito^hi^VCAN^hi^) in COVID-19. Lymphocyte exhaustion is proposed as a common mechanism in COVID-19 and sepsis (Boomer et al., 2012; Diao et al., 2020). We find overall higher levels of CD4^+^ and CD8^+^ T cell activation, and more marked changes in cell markers, in COVID-19, suggesting a greater degree of CD8^+^ T cell exhaustion. While we find no major changes in naive and transitional B cells in either condition, in sepsis we observed an absence of terminally differentiated plasmablasts and fewer clonally related B cells, class switching and autoreactive BCRs. We further identified plasma proteins discriminating COVID-19 and sepsis, for example LBP, lactoferrin, and lipocalin 2; and differential pathways including neutrophil degranulation, complement, AP-1/p38MAPK, TLR, renin-angiotensin, and the HSC lineage. We note that COVID-19-induced downregulation of *ACE2* may be involved in modulating both the renin-angiotensin system and activation of AP-1/p38MAPK signaling (Grimes and Grimes, 2020).

Similarly, while differences in target cells and control of viral replication are recognized between COVID-19 and influenza, shared immune responses and mechanisms are reported (Flerlage et al., 2021). Our data build on previous blood immune signatures (Lee et al., 2020; Mudd et al., 2020; Zhu et al., 2020) to gain cellular and proteomic insights into more severe forms of these two infections. We find similar changes in frequency of major immune cell populations between COVID-19 and influenza for HSC, the majority of T and NK cell populations, classical/cycling monocytes, and plasmablasts; but differences for many B cell populations (differential naive, intermediate, and memory B cell response) as well as specific cell subsets, notably ncMono subpopulations (summarized in graphical abstract). While the bulk of the circulating proteomic response is shared, specific differences involved EGF, G-CSF, and IL-15. We further found extensive sharing of pathways and networks including modules involving JAK-STAT and zinc finger proteins, as well as differences, notably enrichment of AP-1/MAPK in COVID-19.

In conclusion, our multi-omic integrated blood atlas comprehensively delineates the host immune response in COVID-19 from the start of the UK pandemic, prior to clinical trial-led implementation of approved treatments or vaccination. This provides the community with a unique reference resource for replication and meta-analysis, to interpret datasets generated from interventional trials and includes a browser for direct visualization of data (https://mlv.combat.ox.ac.uk/). Integrative approaches such as we have applied here are essential to better differentiate patients according to disease severity, underlying pathophysiology, and infectious etiology. This will be important as we seek novel therapeutic targets and the opportunity for a precision medicine approach to treatment that is appropriately timed and targeted to those patients most likely to benefit from a particular intervention.

### Limitations of the study

This study is limited to analysis of peripheral blood. Future multi-tissue studies including lung samples using multi-omics approaches are needed to further delineate the host response in COVID-19. It will also be important to more clearly establish changes from baseline, such as will be afforded by challenge studies with SARS-CoV-2, and more detailed temporal profiling over the course of acute illness into convalescence. Further work is needed to delineate the role of AP-1/p38 MAPK pathway upregulation in COVID-19, such as investigating the relationship with viral replication and specific disease mechanisms such as T cell exhaustion. AP-1 inhibitors may enable insights into reversibility, the extent and nature of persisting epigenetic differences in convalescence, and relationship with COVID-19 vaccination. Functional studies are needed to understand the relationship between COVID-19 associated Kmers, CD8+ T cell expansion, and cytotoxicity in critical disease but TCR sequences, including an agnostic Kmer analysis, have been used to define T cell responses linked to SARS-CoV-2 (Shoukat et al., 2021).

## STAR★Methods

### Key resources table

**Table:** 

REAGENT or RESOURCE	SOURCE	IDENTIFIER
**Antibodies**		

Mass cytometry panel antibodies (ˆ:intracellular)		
CD45 (HI30)-89Y	Fluidigm	Cat# 3089003, RRID:AB_2661851
CD19 (HIB19)-111Cd	BioLegend	Cat# 302247, RRID:AB_2562815
CD3 (UCHT1)-112Cd	BioLegend	Cat# 300438, RRID:AB_11146991
IgG (M1310G05)-113Cd	BioLegend	Cat# 410701, RRID:AB_2565624
CD4 (RPA-T4)-114Cd	BioLegend	Cat# 300570, RRID:AB_2810427
HLADR (G46-6)-116Cd	BD Bioscience	Cat# 556642, RRID:AB_396508
CD57 (HCD57)-115In	BioLegend	Cat# 322325, RRID:AB_2563757
CD11c (Bu15)-141Pr	BioLegend	Cat# 337202, RRID:AB_1236381
BCL2 (100)-142Ndˆ	BioLegend	Cat# 658702, RRID:AB_2562959
CD45RA (HI100)-143Nd	BioLegend	Cat# 304102, RRID:AB_314406
GZB (CLB-GB11)-144Ndˆ	Abcam	Cat# ab103159, RRID:AB_10715242
CD33 (P67.6)-145Nd	BioLegend	Cat# 366602, RRID:AB_2565538
Vd2 (123R3.5B8)-146Nd	Miltenyi	Custom made
CD20 (2H7)-147Sm	BioLegend	Cat# 302343, RRID:AB_2562816
CD66 (B1.1/CD66)-149Sm	BD Bioscience	Cat# 551354, RRID:AB_394166
CD69 (FN50)-150Nd	BioLegend	Cat# 310902, RRID:AB_314837
CD103 (Ber-ACT8)-151Eu	Fludigm	Cat# 3151011B, RRID:AB_2756418
TCRgd (11F2)-152Smˆ	Fluidigm	Cat# 3152008B, RRID:AB_2687643
IgD (IA6-2)-152Sm	BioLegend	Cat# 348235, RRID:AB_2563775
Va7.2 (3C10)-153Eu	Fluidigm	Cat# 3153024B; RRID: N/A
KLRG1 (14C2A07)-154Sm	BioLegend	Cat# 368602, RRID:AB_2566256
PD1 (EH12.2H7)-155Gd	Fluidigm	Cat# 3155009B, RRID:AB_2811087
CD161 (HP-3G10)-156Gd	BioLegend	Cat# 339902, RRID:AB_1501090
CD27 (L128)-158Gd	Fluidigm	Cat# 3158010B, RRID:AB_2858231
FoxP3 (PCH101)-159Tbˆ	eBioscence	Cat# 14-4776-82, RRID:AB_467554
CD14 (M5E2)-160Gd	BioLegend	Cat# 301843, RRID:AB_2562813
CTLA-4 (14D3)-161Dy	eBioscence	Cat# 14-1529-82, RRID:AB_467512
Siglec-8 (837535)-162Dy	R&D	Cat# MAB7975
CD28 (L293)-163Dy	BD Bioscience	Cat# 340975, RRID:AB_400197
Ki67 (Ki-67)-164Dyˆ	BioLegend	Cat# 350523, RRID:AB_2562838
CD45RO (UCHL1)-165Ho	BioLegend	Cat# 304202, RRID:AB_314418
CD56 (NCAM16.2)-166Er	BD Bioscience	Cat# 559043, RRID:AB_397180
CCR7 (150503)-167Er	R&D	Cat# MAB197, RRID:AB_2072803
CD127 (A019D5)-168Er	BioLegend	Cat# 351302, RRID:AB_10718513
CD38 (HIT2)-169Tm	BioLegend	Cat# 303502, RRID:AB_314354
CD99 (HCD99)-170Er	BioLegend	Cat# 318002, RRID:AB_604112
CD123 (6H6)-171Yb	BioLegend	Cat# 306002, RRID:AB_314576
CD25 (M-A251)-172Yb	BioLegend	Cat# 356102, RRID:AB_2561752
CD141 (M80)-173Yb	BioLegend	Cat# 344102, RRID:AB_2201808
CLA (HECA-452)-174Yb	BioLegend	Cat# 321302, RRID:AB_492894
CD39 (A1)-175Lu	BioLegend	Cat# 328202, RRID:AB_940438
CX3CR1 (2A9-1)-176Yb	BioLegend	Cat# 341602, RRID:AB_1595422
CD8 (RPA-T8)-198Pt	BioLegend	Cat# 301018, RRID:AB_314136
CD16 (3G8)-209 Bi	Fluidigm	Cat# 3209002B, RRID:AB_2756431

**Flow Cytometry Antibody Panels**		

Panel 1		N/A
CD11b-Alexa700 (M1/70)	BioLegend	Cat#101222,RRID:AB_493705
BDCA2-APC (201A)	BioLegend	Cat#354206,RRID:AB_11150412
HLADR-APCCy7 (L243)	BioLegend	Cat#307618,RRID:AB_493586
CD86-BB515 (FUN-1)	BD Bioscience	Cat#564544,RRID:AB_2744453
CD33-BB630 (WM-53)	BD Bioscience	Custom made
CD38-BB790 (HIT2)	BD Bioscience	Custom made
IgM-BUV395 (G20-127)	BD Bioscience	Cat#551062,RRID:AB_398487
CD20-BUV496 (2H7)	BD Bioscience	Cat#749954,RRID:AB_2874186
CD56-BUV563 (MY31)	BD Bioscience	Cat#748610,RRID:AB_2873017
PDL1-BUV615-P (MIH1)	BD Bioscience	Cat#751185,RRID:AB_2875207
CD3-BUV661 (UCHT1)	BD Bioscience	Cat#612964,RRID:AB_2870239
CD80-BV421 (2D10)	BioLegend	Cat#305222,RRID:AB_2564407
CD19-BV510 (HIB19)	BioLegend	Cat#302242,RRID:AB_2561668
CD14-BV605 (M5E2)	BioLegend	Cat#301834,RRID:AB_2563798
IgD-BV605 (IA6-2)	BD Bioscience	Cat#563313,RRID:AB_2738134
CD16-BV650 (3G8)	BioLegend	Cat#302042,RRID:AB_2563801
CD40-BV711 (5C3)	BioLegend	Cat#334334,RRID:AB_2564212
CD123-BV786 (6H6)	BioLegend	Cat#306032,RRID:AB_2566448
CD27-PE (LG.3A10)	BioLegend	Cat#124210,RRID:AB_1236459
CD137-PECF594 (4B4-1)	BioLegend	Cat#309826,RRID:AB_2566260
CD1c-PECy7 (L161)	BioLegend	Cat#331516,RRID:AB_2275574
CD141-PerCPCy5.5 (M80)	BioLegend	Cat#344112,RRID:AB_2561625
Panel 2		N/A
CD16-Alexa700 (3G8)	BioLegend	Cat#302026,RRID:AB_2278418
CD161-APC (HP3G10)	BioLegend	Cat#339912,RRID:AB_10900826
CCR7-APCCy7 (G043H7)	BioLegend	Cat#353212,RRID:AB_10916390
CD45RA-BB515 (HI100)	BD Bioscience	Cat#564552,RRID:AB_2738841
TCRgd-BB700 (11F2)	BD Bioscience	Cat#745944,RRID:AB_2743364
CD38-BB790 (HIT2)	BD Bioscience	Custom made
CD103-BUV (Ber-ACT8)	BD Bioscience	Cat#748501,RRID:AB_2872912
CD26-BUV395 (L272)	BD Bioscience	Cat#745724,RRID:AB_2743201
CD8-BUV496 (RPA-T8)	BD Bioscience	Cat#612942,RRID:AB_2870223
CD56-BUV563 (MY31)	BD Bioscience	Cat#748610,RRID:AB_2873017
CD4-BUV615-P (SK3)	BD Bioscience	Cat#612987,RRID:AB_2870258
6B11-BUV661 (6B11)	BD Bioscience	Cat#750268,RRID:AB_2874460
CD137-BUV737 (4B4-1)	BD Bioscience	Cat#741861,RRID:AB_2871191
CD69-BV421 (FN50)	BioLegend	Cat#310930,RRID:AB_2561909
CD14-BV510 (M5E2)	BioLegend	Cat#301841,RRID:AB_2561379
CD19-BV510 (HIB19)	BioLegend	Cat#302242,RRID:AB_2561668
Va7.2-BV605 (3C10)	BioLegend	Cat#351720,RRID:AB_2563991
IgD-BV605 (IA6-2)	BioLegend	Cat#348231,RRID:AB_2563336
CD3-BV650 (UCHT1)	BioLegend	Cat#300468,RRID:AB_2629574
CD39-BV711 (A1)	BioLegend	Cat#328228,RRID:AB_2632894
HLADR-BV786 (L243)	BioLegend	Cat#307642,RRID:AB_2563461
TIM3-PE (7D3)	BD Bioscience	Cat#563422,RRID:AB_2716866
PD1-PECF594 (EH12 2H7)	BioLegend	Cat#329940,RRID:AB_2563659
CD25-PECy7 (BC96)	BioLegend	Cat#302612,RRID:AB_314282
Panel 3		N/A
CD27-Alexa700 (O323)	BioLegend	Cat#313509,RRID:AB_416333
ICOS-APC (C398.4A)	BioLegend	Cat#313509,RRID:AB_416333
CD25-BB515 (M-A251)	BD Bioscience	Cat#565096,RRID:AB_2739065
CD38-BB790 (HIT2)	BD Bioscience	Custom made
CD8-BUV496 (RPA-T8)	BD Bioscience	Cat#612942,RRID:AB_2870223
CD4-BUV615-P (SK3)	BD Bioscience	Cat#612987,RRID:AB_2870258
CXCR3-BV421 (G025H7)	BioLegend	Cat#353715,RRID:AB_11124720
CD14-BV510 (M5E2)	BioLegend	Cat#301841,RRID:AB_2561379
CD19-BV510 (HIB19)	BioLegend	Cat#302242,RRID:AB_2561668
CCR4-BV605 (L291H4)	BioLegend	Cat#359417,RRID:AB_2562482
CD45RA-BV711 (HI100)	BioLegend	Cat#304138,RRID:AB_2563815
HLADR-BV786 (L243)	BioLegend	Cat#307642,RRID:AB_2563461
CD127-PE (A019D5)	BioLegend	Cat#351303,RRID:AB_10719960
PD1-PECF594 (EH12 2H7)	BioLegend	Cat#329940,RRID:AB_2563659
CCR6-PECy7 (29-2L17)	BioLegend	Cat#302813,RRID:AB_493756
CXCR5-PerCPCy5.5 (J252D4)	BioLegend	Cat#356910,RRID:AB_2561819

**Myeloid-marker enriched mass cytometry panel antibodies (ˆ:intracellular)**		

CD45 (HI30)-89Y	Fluidigm	Cat# 3089003, RRID:AB_2661851
CD5 (UCHT2)-111Cd	Biolegend	Cat# 300627, RRID:AB_2563756
CD3 (UCHT1)-112Cd	Biolegend	Cat# 300443, RRID:AB_2562808
CD9 (HI91)-113Cd	Biolegend	Cat# 312102, RRID:AB_314907
CD7 (CD7-6B7)-114Cd	Biolegend	Cat# 343111, RRID:AB_2563761
HLADR (L243)-116Cd	Biolegend	Cat# 307651, RRID:AB_2562826
CD49d (9F10)-141Pr	Fluidigm	Cat# 3141004B, RRID:AB_2892684
CD19 (HIB19)-142Nd	Fluidigm	Cat# 3142001B, RRID:AB_2651155
CD123 (6H6)-143Nd	Fluidigm	Cat# 3143014B, RRID:AB_2811081
CD15 (W6D3)-144Nd	Fluidigm	Cat# 3144019B, RRID:AB_2892685
CD38 (HIT2)-145Nd	Biolegend	Cat# 303535, RRID:AB_2562819
CD64 (10.1)-146Nd	Fluidigm	Cat# 3146006B, RRID:AB_2661790
CD11c (Bu15)-147Sm	Fluidigm	Cat# 3147008B, RRID:AB_2687850
CD16 (3G8)-148Nd	Fluidigm	Cat# 3148004B, RRID:AB_2661791
CD74 (LN2)-149Sm	Biolegend	Cat# 326802, RRID:AB_893401
CD43 (84-3C1)-150Nd	Fluidigm	Cat# 3150006B, RRID:AB_2892686
CD103 (Ber-ACT8)-151Eu	Fluidigm	Cat# 3151011B, RRID:AB_2756418
CD66b (80H3)-152Sm	Fluidigm	Cat# 3152011B, RRID:AB_2661795
BDCA2 (201A)-153Eu	Fluidigm	Cat# 3153007B, RRID:AB_2892687
CD163 (GHI/61)-154Sm	Fluidigm	Cat# 3154007B, RRID:AB_2661797
CD36 (5-271)-155Gd	Fluidigm	Cat# 3155012B, RRID:AB_2756286
CD10 (Hi10a)-156Gd	Fluidigm	Cat# 3156001B, RRID:AB_2802107
CD33 (WM53)-158Gd	Fluidigm	Cat# 3158001B, RRID:AB_2661799
CD22 (HIB22)-159Tb	Fluidigm	Cat# 3159005B, RRID:AB_2892688
CD14 (M5E2)-160Gd	Fluidigm	Cat# 3160001B, RRID:AB_2687634
CLEC9A (8F9)-161Dy	Fluidigm	Cat# 3161018B, RRID:AB_2810252
ki67 (B56)-162Dyˆ	Fluidigm	Cat# 3162012B, RRID:AB_2888928
CD172a/b (SE5A5)-163Dy	Fluidigm	Cat# 3163017B, RRID:AB_2864730
Siglec 8 (7C9)-164Dy	Fluidigm	Cat# 3164017B, RRID:AB_2892691
CD101 (BB27)-165Ho	Fluidigm	Cat# 3165029B, RRID:AB_2892692
CD141 (M80)-166Er	Fluidigm	Cat# 3166017B, RRID:AB_2892693
CD301 (H037G3)-167Er	Fluidigm	Cat# 3167015B, RRID:AB_2892694
CD71 (OKT9)-168Er	Fluidigm	Cat# 3168014B, RRID:AB_2892695
BDCA4 (12C2)-169Tm	Fluidigm	Cat# 3169018B, RRID:AB_2892697
CD114 (38660)-170Er	R&D	Cat# MAB381, RRID:AB_2245228
CD226 (DX11)-171Yb	Fluidigm	Cat# 3171013B, RRID:AB_2756427
CD354 (TREM-26)-172Yb	Fluidigm	Cat# 3172022B, RRID:AB_2756428
CD371 (50C1)-173Yb	Fluidigm	Cat# 3173007B, RRID:AB_2892698
CD142 (NY2)-174Yb	Biolegend	Cat# 365202, RRID:AB_2564564

**CITE-seq antibody panel**		

192 TotalSeq-C antibody panel, as detailed below:	BioLegend	Cat# 99814
anti-human CD80 (clone 2D10)	BioLegend	Cat# 99814
anti-human CD86 (clone IT2.2)	BioLegend	Cat# 99814
anti-human CD274 (B7-H1, PD-L1) (clone 29E.2A3)	BioLegend	Cat# 99814
anti-human CD273 (B7-DC, PD-L2) (clone 24F.10C12)	BioLegend	Cat# 99814
anti-human CD275 (B7-H2, ICOSL) (clone 2D3)	BioLegend	Cat# 99814
anti-mouse/human CD11b (clone M1/70)	BioLegend	Cat# 99814
anti-human CD252 (OX40L) (clone 11C3.1)	BioLegend	Cat# 99814
anti-human CD137L (4-1BB Ligand) (clone 5F4)	BioLegend	Cat# 99814
anti-human CD155 (PVR) (clone SKII.4)	BioLegend	Cat# 99814
anti-human CD112 (Nectin-2) (clone TX31)	BioLegend	Cat# 99814
anti-human CD47 (clone CC2C6)	BioLegend	Cat# 99814
anti-human CD70 (clone 113-16)	BioLegend	Cat# 99814
anti-human CD30 (clone BY88)	BioLegend	Cat# 99814
anti-human CD40 (clone 5C3)	BioLegend	Cat# 99814
anti-human CD154 (clone 24-31)	BioLegend	Cat# 99814
anti-human CD52 (clone HI186)	BioLegend	Cat# 99814
anti-human CD3 (clone UCHT1)	BioLegend	Cat# 99814
anti-human CD8 (clone SK1)	BioLegend	Cat# 99814
anti-human CD56 (NCAM) (clone 5.1H11)	BioLegend	Cat# 99814
anti-human CD19 (clone HIB19)	BioLegend	Cat# 99814
anti-human CD33 (clone P67.6)	BioLegend	Cat# 99814
anti-human CD11c (clone S-HCL-3)	BioLegend	Cat# 99814
anti-human CD34 (clone 581)	BioLegend	Cat# 99814
anti-human CD269 (BCMA) (clone 19F2)	BioLegend	Cat# 99814
anti-human HLA-A,B,C (clone W6/32)	BioLegend	Cat# 99814
anti-human CD90 (Thy1) (clone 5E10)	BioLegend	Cat# 99814
anti-human CD117 (c-kit) (clone 104D2)	BioLegend	Cat# 99814
anti-human CD10 (clone HI10a)	BioLegend	Cat# 99814
anti-human CD45RA (clone HI100)	BioLegend	Cat# 99814
anti-human CD123 (clone 6H6)	BioLegend	Cat# 99814
anti-human CD7 (clone CD7-6B7)	BioLegend	Cat# 99814
anti-human/mouse CD49f (clone GoH3)	BioLegend	Cat# 99814
anti-human CD194 (CCR4) (clone L291H4)	BioLegend	Cat# 99814
anti-human CD4 (clone RPA-T4)	BioLegend	Cat# 99814
anti-mouse/human CD44 (clone IM7)	BioLegend	Cat# 99814
anti-human CD14 (clone M5E2)	BioLegend	Cat# 99814
anti-human CD16 (clone 3G8)	BioLegend	Cat# 99814
anti-human CD25 (clone BC96)	BioLegend	Cat# 99814
anti-human CD45RO (clone UCHL1)	BioLegend	Cat# 99814
anti-human CD279 (PD-1) (clone EH12.2H7)	BioLegend	Cat# 99814
anti-human TIGIT (VSTM3) (clone A15153G)	BioLegend	Cat# 99814
Mouse IgG1, κ isotype Ctrl (clone MOPC-21)	BioLegend	Cat# 99814
Mouse IgG2a, κ isotype Ctrl (clone MOPC-173)	BioLegend	Cat# 99814
Mouse IgG2b, κ isotype Ctrl (clone MPC-11)	BioLegend	Cat# 99814
Rat IgG2b, κ Isotype Ctrl (clone RTK4530)	BioLegend	Cat# 99814
anti-human CD20 (clone 2H7)	BioLegend	Cat# 99814
anti-human CD335 (NKp46) (clone 9E2)	BioLegend	Cat# 99814
anti-human CD294 (CRTH2) (clone BM16)	BioLegend	Cat# 99814
anti-human CD326 (Ep-CAM) (clone 9C4)	BioLegend	Cat# 99814
anti-human CD31 (clone WM59)	BioLegend	Cat# 99814
anti-Human Podoplanin (clone NC-08)	BioLegend	Cat# 99814
anti-human CD146 (clone P1H12)	BioLegend	Cat# 99814
anti-human CD324 (E-Cadherin) (clone 67A4)	BioLegend	Cat# 99814
anti-human IgM (clone MHM-88)	BioLegend	Cat# 99814
anti-human CD5 (clone UCHT2)	BioLegend	Cat# 99814
anti-human TCR γ/δ (clone B1)	BioLegend	Cat# 99814
anti-human CD183 (CXCR3) (clone G025H7)	BioLegend	Cat# 99814
anti-human CD195 (CCR5) (clone J418F1)	BioLegend	Cat# 99814
anti-human CD32 (clone FUN-2)	BioLegend	Cat# 99814
anti-human CD196 (CCR6) (clone G034E3)	BioLegend	Cat# 99814
anti-human CD185 (CXCR5) (clone J252D4)	BioLegend	Cat# 99814
anti-human CD103 (Integrin αE) (clone Ber-ACT8)	BioLegend	Cat# 99814
anti-human CD69 (clone FN50)	BioLegend	Cat# 99814
anti-human CD62L (clone DREG-56)	BioLegend	Cat# 99814
anti-human CD197 (CCR7) (clone G043H7)	BioLegend	Cat# 99814
anti-human CD161 (clone HP-3G10)	BioLegend	Cat# 99814
anti-human CD152 (CTLA-4) (clone BNI3)	BioLegend	Cat# 99814
anti-human CD223 (LAG-3) (clone 11C3C65)	BioLegend	Cat# 99814
anti-human KLRG1 (MAFA) (clone SA231A2)	BioLegend	Cat# 99814
anti-human CD27 (clone O323)	BioLegend	Cat# 99814
anti-human CD107a (LAMP-1) (clone H4A3)	BioLegend	Cat# 99814
anti-human CD95 (Fas) (clone DX2)	BioLegend	Cat# 99814
anti-human HLA-DR (clone L243)	BioLegend	Cat# 99814
anti-human CD1c (clone L161)	BioLegend	Cat# 99814
anti-human CD64 (clone 10.1)	BioLegend	Cat# 99814
anti-human CD141 (Thrombomodulin) (clone M80)	BioLegend	Cat# 99814
anti-human CD1d (clone 51.1)	BioLegend	Cat# 99814
anti-human CD314 (NKG2D) (clone 1D11)	BioLegend	Cat# 99814
anti-human CD66b (clone 6/40c)	BioLegend	Cat# 99814
anti-human CD35 (clone E11)	BioLegend	Cat# 99814
anti-human CD57 Recombinant (clone QA17A04)	BioLegend	Cat# 99814
anti-human CD366 (Tim-3) (clone F38-2E2)	BioLegend	Cat# 99814
anti-human CD272 (BTLA) (clone MIH26)	BioLegend	Cat# 99814
anti-human/mouse/rat CD278 (ICOS) (clone C398.4A)	BioLegend	Cat# 99814
anti-human CD58 (LFA-3) (clone TS2/9)	BioLegend	Cat# 99814
anti-human CD96 (TACTILE) (clone NK92.39)	BioLegend	Cat# 99814
anti-human CD39 (clone A1)	BioLegend	Cat# 99814
anti-human CD178 (Fas-L) (clone NOK-1)	BioLegend	Cat# 99814
anti-human CX3CR1 (clone K0124E1)	BioLegend	Cat# 99814
anti-human CD24 (clone ML5)	BioLegend	Cat# 99814
anti-human CD21 (clone Bu32)	BioLegend	Cat# 99814
anti-human CD11a (clone TS2/4)	BioLegend	Cat# 99814
anti-human IgA (clone HP6123)	BioLegend	Cat# 99814
anti-human CD79b (Igβ) (clone CB3-1)	BioLegend	Cat# 99814
anti-human CD66a/c/e (clone ASL-32)	BioLegend	Cat# 99814
anti-human CD244 (2B4) (clone C1.7)	BioLegend	Cat# 99814
anti-human CD235ab (clone HIR2)	BioLegend	Cat# 99814
anti-human CD206 (MMR) (clone 15-2)	BioLegend	Cat# 99814
anti-human CD169 (Sialoadhesin, Siglec-1) (clone 7-239)	BioLegend	Cat# 99814
anti-human CD370 (CLEC9A/DNGR1) (clone 8F9)	BioLegend	Cat# 99814
anti-human XCR1 (clone S15046E)	BioLegend	Cat# 99814
anti-human/mouse integrin β7 (clone FIB504)	BioLegend	Cat# 99814
anti-human CD268 (BAFF-R) (clone 11C1)	BioLegend	Cat# 99814
anti-human CD54 (clone HA58)	BioLegend	Cat# 99814
anti-human CD62P (P-Selectin) (clone AK4)	BioLegend	Cat# 99814
anti-human TCR α/β (clone IP26)	BioLegend	Cat# 99814
anti-human CD106 (clone STA)	BioLegend	Cat# 99814
anti-human CD122 (IL-2Rβ) (clone TU27)	BioLegend	Cat# 99814
anti-human CD267 (TACI) (clone 1A1)	BioLegend	Cat# 99814
anti-human FcεRIα (clone AER-37 (CRA-1))	BioLegend	Cat# 99814
anti-human CD41 (clone HIP8)	BioLegend	Cat# 99814
anti-human CD137 (4-1BB) (clone 4B4-1)	BioLegend	Cat# 99814
anti-human CD254 (TRANCE, RANKL) (clone MIH24)	BioLegend	Cat# 99814
anti-human CD163 (clone GHI/61)	BioLegend	Cat# 99814
anti-human CD83 (clone HB15e)	BioLegend	Cat# 99814
anti-human CD357 (GITR) (clone 108-17)	BioLegend	Cat# 99814
anti-human CD309 (VEGFR2) (clone 7D4-6)	BioLegend	Cat# 99814
anti-human CD124 (IL-4Rα) (clone G077F6)	BioLegend	Cat# 99814
anti-human CD184 (CXCR4) (clone 12G5)	BioLegend	Cat# 99814
anti-human CD2 (clone TS1/8)	BioLegend	Cat# 99814
anti-human CD226 (DNAM-1) (clone 11A8)	BioLegend	Cat# 99814
anti-human CD29 (clone TS2/16)	BioLegend	Cat# 99814
anti-human CD303 (BDCA-2) (clone 201A)	BioLegend	Cat# 99814
anti-human CD49b (clone P1E6-C5)	BioLegend	Cat# 99814
anti-human CD81 (TAPA-1) (clone 5A6)	BioLegend	Cat# 99814
anti-human CD98 (clone MEM-108)	BioLegend	Cat# 99814
anti-human IgG Fc (clone M1310G05)	BioLegend	Cat# 99814
anti-human IgD (clone IA6-2)	BioLegend	Cat# 99814
anti-human CD18 (clone TS1/18)	BioLegend	Cat# 99814
anti-human CD28 (clone CD28.2)	BioLegend	Cat# 99814
anti-human TSLPR (TSLP-R) (clone 1D3)	BioLegend	Cat# 99814
anti-human CD38 (clone HIT2)	BioLegend	Cat# 99814
anti-human CD127 (IL-7Rα) (clone A019D5)	BioLegend	Cat# 99814
anti-human CD45 (clone HI30)	BioLegend	Cat# 99814
anti-human CD15 (SSEA-1) (clone W6D3)	BioLegend	Cat# 99814
anti-human CD22 (clone S-HCL-1)	BioLegend	Cat# 99814
anti-human CD71 (clone CY1G4)	BioLegend	Cat# 99814
anti-human B7-H4 (clone MIH43)	BioLegend	Cat# 99814
anti-human CD26 (clone BA5b)	BioLegend	Cat# 99814
anti-human CD193 (CCR3) (clone 5E8)	BioLegend	Cat# 99814
anti-human CD204 (clone 7C9C20)	BioLegend	Cat# 99814
anti-human CD144 (VE-Cadherin) (clone BV9)	BioLegend	Cat# 99814
anti-human CD1a (clone HI149)	BioLegend	Cat# 99814
anti-human CD304 (Neuropilin-1) (clone 12C2)	BioLegend	Cat# 99814
anti-human CD36 (clone 5-271)	BioLegend	Cat# 99814
anti-human CD158 (KIR2DL1/S1/S3/S5) (clone HP-MA4)	BioLegend	Cat# 99814
anti-mouse/human CD207 (clone 4C7)	BioLegend	Cat# 99814
anti-human CD49d (clone 9F10)	BioLegend	Cat# 99814
anti-human CD73 (Ecto-5′-nucleotidase) (clone AD2)	BioLegend	Cat# 99814
anti-human TCR Vα7.2 (clone 3C10)	BioLegend	Cat# 99814
anti-human TCR Vδ2 (clone B6)	BioLegend	Cat# 99814
anti-human TCR Vγ9 (clone B3)	BioLegend	Cat# 99814
anti-human TCR Vα24-Jα18 (iNKT cell) (clone 6B11)	BioLegend	Cat# 99814
anti-human CD305 (LAIR1) (clone NKTA255)	BioLegend	Cat# 99814
anti-human LOX-1 (clone 15C4)	BioLegend	Cat# 99814
anti-human CD158b (KIR2DL2/L3, NKAT2) (clone DX27)	BioLegend	Cat# 99814
anti-human CD133 (clone S16016B)	BioLegend	Cat# 99814
anti-human CD209 (DC-SIGN) (clone 9E9A8)	BioLegend	Cat# 99814
anti-human CD158e1 (KIR3DL1, NKB1) (clone DX9)	BioLegend	Cat# 99814
anti-human CD158f (KIR2DL5) (clone UP-R1)	BioLegend	Cat# 99814
anti-human CD337 (NKp30) (clone P30-15)	BioLegend	Cat# 99814
anti-human CD336 (NKp44) (clone P44-8)	BioLegend	Cat# 99814
anti-human CD307d (FcRL4) (clone 413D12)	BioLegend	Cat# 99814
anti-human CD307e (FcRL5) (clone 509f6)	BioLegend	Cat# 99814
anti-human CD319 (CRACC) (clone 162.1)	BioLegend	Cat# 99814
anti-human CD138 (Syndecan-1) (clone DL-101)	BioLegend	Cat# 99814
anti-human CD99 (clone 3B2/TA8)	BioLegend	Cat# 99814
anti-human CLEC12A (clone 50C1)	BioLegend	Cat# 99814
anti-Tau Phospho (Thr181) (clone M7004D06)	BioLegend	Cat# 99814
anti-human CD257 (BAFF, BLYS) (clone 1D6)	BioLegend	Cat# 99814
anti-human CD94 (clone DX22)	BioLegend	Cat# 99814
anti-human CD150 (SLAM) (clone A12 (7D4))	BioLegend	Cat# 99814
anti-human Ig light chain κ (clone MHK-49)	BioLegend	Cat# 99814
anti-mouse/human Mac-2 (Galectin-3) (clone M3/38)	BioLegend	Cat# 99814
anti-human CD85j (ILT2) (clone GHI/75)	BioLegend	Cat# 99814
anti-human CD23 (clone EBVCS-5)	BioLegend	Cat# 99814
anti-human Ig light chain λ (clone MHL-38)	BioLegend	Cat# 99814
anti-human HLA-A2 (clone BB7.2)	BioLegend	Cat# 99814
anti-human GARP (LRRC32) (clone 7B11)	BioLegend	Cat# 99814
anti-human CD328 (Siglec-7) (clone 6-434)	BioLegend	Cat# 99814
anti-human TCR Vβ13.1 (clone H131)	BioLegend	Cat# 99814
anti-human CD82 (clone ASL-24)	BioLegend	Cat# 99814
anti-human CD101 (BB27) (clone BB27)	BioLegend	Cat# 99814
anti-human CD360 (IL-21R) (clone 4B2.9)	BioLegend	Cat# 99814
anti-human CD88 (C5aR) (clone S5/1)	BioLegend	Cat# 99814
anti-human HLA-F (clone 3D11/HLA-F)	BioLegend	Cat# 99814
anti-human NLRP2 (clone 8F10B51)	BioLegend	Cat# 99814
anti-human Podocalyxin (clone mAb 84)	BioLegend	Cat# 99814
anti-human CD224 (clone KF29)	BioLegend	Cat# 99814
anti-c-Met (clone 12.1)	BioLegend	Cat# 99814
anti-human CD258 (LIGHT) (clone T5-39)	BioLegend	Cat# 99814
anti-human DR3 (TRAMP) (clone JD3)	BioLegend	Cat# 99814

**Chemicals, peptides, and recombinant proteins**		

Water, LiChrosolv grade	Merck	1-15333.2500
Acetonitrile, LiChrosolv grade	Merck	1.00030.2500
Methanol, LiChrosolv grade	Merck	1.06035.2500
Formic Acid Optima LC-MS grade	Fisher Scientific	10596814
1-Propanolol	Sigma-Aldrich	34871-1L
Trifluoroacetic acid (TFA)	Sigma-Aldrich	74664-10ml
Triethylammonium bicarbonate buffer (TEAB, 1M)	Sigma-Aldrich	T7408-100ml
Ammonium formate	Sigma-Aldrich	70221-100 g
TPCK treated Trypsin (100μg)	Worthington	LS003740
C18 Evotips	Evosep One (Odense, Denmark)	EV-2001
96-well S-trap	Profiti (Huntington, NY, USA)	C02-96wel
PQ500 reference peptide	Biognosys	K-3019
SOLA HRP SPE 10mg/1ml	Fisher Scientific	11879163
Benzonase	Thermo Scientific	Cat# 88700; RRID: N/A
Maxpar® Cell Staining Buffer	Fluidigm	Cat# 201068; RRID: N/A
Maxpar® Nuclear Antigen Staining Buffer Set	Fluidigm	Cat# 201063; RRID: N/A
eBioscience™ IC Fixation Buffer	Thermo Scientific	Cat# 00-8222-49; RRID: N/A
Cell-ID Cisplatin Pt198	Fluidigm	Cat# 201198; RRID: N/A
Cell-ID Intercalator-Ir	Fluidigm	Cat# 201192B; RRID: N/A
EQ Four Element Calibration Beads	Fluidigm	Cat# 201078; RRID: N/A
Maxpar Cell Acquisition Solution (CAS)	Fluidigm	Cat# 201240; RRID; N/A
Maxpar Water	Fluidigm	Cat# 201069; RRID: N/A
Metal isotopes as chloride salts (In-115)	Trace Sciences International	Customized
Protein Stabilizer PBS	Candor Bioscience	Cat# 131125, RRID: N/A
Brilliant stain buffer	BD Bioscience	Cat# 563794; RRID: N/A
7-AAD dye	BioLegend	Cat# 420404
FcX	BioLegend	Cat# 422301
Diluted Nuclei Buffer	10x Genomics	Cat# 2000153

**Critical commercial assays**		

Whole blood (human) processing kit	Cytodelics	C001-500; RRID: N/A
Cell-ID 20-Plex Pd Barcoding Kit	Fluidigm	Cat# 201060; RRID: N/A
EasySep™ HLA Chimerism Whole Blood CD66b Positive Selection Kit	Easysep	Cat# 17882; RRID: N/A
Cell-ID 20-Plex Pd Barcoding Kit	Fluidigm	Cat# 201060; RRID: N/A
Maxpar MCP9 Antibody Labeling Kit −110Cd	Fluidigm	Cat# 201110A; RRID: N/A
Maxpar MCP9 Antibody Labeling Kit −111Cd	Fluidigm	Cat# 201111A; RRID: N/A
Maxpar MCP9 Antibody Labeling Kit −112Cd	Fluidigm	Cat# 201112A; RRID: N/A
Maxpar MCP9 Antibody Labeling Kit −113Cd	Fluidigm	Cat# 201113A; RRID: N/A
Maxpar MCP9 Antibody Labeling Kit −114Cd	Fluidigm	Cat# 201114A; RRID: N/A
Maxpar X8 Multimetal Labeling Kit (40 rxn)	Fluidigm	Cat# 201300; RRID: N/A
Zombie Violet™ Fixable Viability Kit	BioLegend	Cat# 423102; RRID: N/A
NEBNext® Globin & rRNA Depletion Kit (Human/Mouse/Rat)	New England Biolabs	E7750X
NEBNext® Ultra™ II Directional RNA Library Prep Kit for Illumina®	New England Biolabs	E7760L
NEBNext Poly(A) mRNA Magnetic Isolation Module	New England Biolabs	E7490L
MiSeq Reagent Nano Kit v2 (300-cycles): MS-103-1001	Illumina Cambridge Ltd	MS-103-1001
MiSeq Reagent Kit v3 (600-cycle): MS-102-3003	Illumina Cambridge Ltd	MS-102-3003
NextSeq 500/550 High Output Kit v2.5 (150 Cycles)	Illumina Cambridge Ltd	20024907
NovaSeq 6000 S4 Reagent Kit (200 cycles): 20027466	Illumina Cambridge Ltd	20027466
NovaSeq 6000 S2 Reagent Kit (200 cycles): 20012861	Illumina Cambridge Ltd	20012861
NovaSeq 6000 SP Reagent Kit (200 cycles): 20040326	Illumina Cambridge Ltd	20040326
NovaSeq 6000 SP Reagent Kit (300 cycles): 20027465	Illumina Cambridge Ltd	20027465
NovaSeq XP 2-Lane Kit	Illumina Cambridge Ltd	20021664
NovaSeq Xp 4-Lane Kit: 20021665	Illumina Cambridge Ltd	20021665
Nextera® XT DNA Sample Preparation Kit (96 Samples)	Illumina UK Ltd	FC-131-1096
Single Index Kit T Set A, 96 rxns	10XGenomics	1000213
Chromium™ Single Cell 5′ Library Construction Kit, 16 rxns	10XGenomics	1000020
Chromium Next GEM Single Cell 5′ Library and Gel Bead Kit v1.1, 16 rxns	10XGenomics	1000165
Chromium Next GEM Chip G Single Cell Kit	10XGenomics	1000127
Chromium™ Single Cell V(D)J Enrichment Kit, Human T Cell, 96 rxns	10XGenomics	1000005
Chromium™ Single Cell V(D)J Enrichment Kit, Human B Cell, 96 rxns	10XGenomics	1000016
Single Index Kit N Set A, 96 rxns	10XGenomics	1000212
Chromium Single Cell 5′ Feature Barcode Library Kit, 16 rxns	10XGenomics	1000080
Chromium Next GEM Chip H Single Cell Kit	10XGenomics	1000162
Chromium Next GEM Single Cell ATAC Library & Gel Bead Kit v1.1	10XGenomics	1000176
TotalSeqTM-C Custom Human panel - All Ab’s (pouch)	BioLegend	99814
KAPA dual Indexed Adaptor Kit	Roche	KK8722
KAPA Hyper Prep Kit	Roche	KK8504
Human Magnetic Luminex Assay kit	Biotechne	LXSAHM-04
Human Magnetic Luminex Assay kit	Biotechne	LXSAHM-23
Human Magnetic Luminex Assay kit	Biotechne	LXSAHM-24

**Deposited data**		

**Processed (KEY) datasets**		N/A
**CBD-KEY-CLINVAR:** Anonymized patient metadata and associated data dictionary	This paper	Zenodo: https://doi.org/10.5281/zenodo.6120249
**CBD-KEY-ATAC**: Peak locations, normalized enrichments, differential expression results, and motif enrichment results generated using single-cell ATAC-seq	This paper	Zenodo: https://doi.org/10.5281/zenodo.6120249
**CBD-KEY-CITESEQ-ANNDATA**: *Contains two anndata objects:*	This paper	Zenodo: https://doi.org/10.5281/zenodo.6120249 For the purpose of online visualization a limited version of the gene expression data is also provided via the CZI Science cellxgene Data Portal: https://cellxgene.cziscience.com/collections/8f126edf-5405-4731-8374-b5ce11f53e82
(1) “COMBAT-CITESeq-DATA”: the raw and normalized gene expression and ADT data, cluster annotations, summary repertoire information and detailed metadata as reported and analyzed in this study.		
(2) “COMBAT-CITESeq-EXPRESSION-ATLAS”: raw and normalized gene expression data from an alternative mapping of the data to a transcriptome index that additionally included genes belonging to the “lncRNA” biotype. With ADT data, cluster annotations and a subset of clinical metadata. Suitable for visualization with cellxgene.		
**CBD-KEY-CITESEQ-GEX-COMPOSITION**: Per sample cell counts and frequencies (at different levels of cell subpopulation resolution)	This paper	Zenodo: https://doi.org/10.5281/zenodo.6120249
**CBD-KEY-CITESEQ-GEX-PSEUDOBULKS**: Raw and normalized pseudobulk counts generated for each combination of gene and sample at minor subset, major subset and cell type level by summing together the within-group gene counts	This paper	Zenodo: https://doi.org/10.5281/zenodo.6120249
**CBD-KEY-CITESEQ-GEX-DIFFEXP**: Differential expression and pathway enrichment results.	This paper	Zenodo: https://doi.org/10.5281/zenodo.6120249
**CBD-KEY-CITESEQ-GEX-WGCNA**: WGCNA analysis of selected cell subpopulations. Module overview, eigengenes and gene membership.	This paper	Zenodo: https://doi.org/10.5281/zenodo.6120249
**CBD-KEY-CITESEQ-VDJ-B**: B cell receptor sequences (amino acid) and a summary of clones (by patient).	This paper	Zenodo: https://doi.org/10.5281/zenodo.6120249
**CBD-KEY-CITESEQ-VDJ-T**: T cell receptor sequences (amino acid), summary of clones (by patient), diversity scores, Kmers, cytotoxicity score.	This paper	Zenodo: https://doi.org/10.5281/zenodo.6120249
**CBD-KEY-CYTOF-MYELOID**: CyTOF data generated from whole blood and granulocyte depleted whole blood, with a focus on myeloid populations. Processed data files in FCS format; CSV files of selected marker expression for monocytes and neutrophils.	This paper	Zenodo: https://doi.org/10.5281/zenodo.6120249
**CBD-KEY-CYTOF-WB**: CyTOF data generated from whole blood. Expression matrices, per sample cell counts and frequencies, and composition analysis results	This paper	Zenodo: https://doi.org/10.5281/zenodo.6120249
**CBD-KEY-CYTOF-WB-D**: CyTOF data generated from granulocyte depleted whole blood. Dataset contains expression matrices, per sample cell counts and frequencies, and composition analysis results.	This paper	Zenodo: https://doi.org/10.5281/zenodo.6120249
**CBD-KEY-FACS**: Flow cytometry data from PBMC cells. Dataset contains a summary of patient by run, frequencies of populations calculated according to the gating strategy depicted in the analysis workspace file (.wps), and linkers between original sample IDs and FACS file nomenclature.	This paper	Zenodo: https://doi.org/10.5281/zenodo.6120249
**CBD-KEY-LUMINEX**: Raw and normalized expression matrices from Luminex-based assays of blood proteins	This paper	Zenodo: https://doi.org/10.5281/zenodo.6120249
**CBD-KEY-ML**: Principal components and features for supervised machine learning to classify samples according to their WHO severity.	This paper	Zenodo: https://doi.org/10.5281/zenodo.6120249
**CBD-KEY-PROTEOMICS**: Raw and processed proteomics data generated using Tims-TOF mass spectrometry	This paper	ProteomeXchange Consortium via the PRIDE partner repository with dataset identifier PXD023175
**CBD-KEY-REPERTOIRE-B**: B cell bulk repertoire datasets. Contains B cell repertoire feature tables including repertoire metrics and V gene usages	This paper	Zenodo: https://doi.org/10.5281/zenodo.6120249
**CBD-KEY-REPERTOIRE-T**: T cell bulk repertoire datasets. Contains T cell repertoire feature tables including repertoire metrics and V gene usages	This paper	Zenodo: https://doi.org/10.5281/zenodo.6120249
**CBD-KEY-RNASEQ-GENOTYPES**: gVCF file per patient obtained from the bulk/mini-bulk RNaseq data.	This paper	EGAD00001007959
**CBD-KEY-RNASEQ-WB**: Raw and log normalized gene counts derived from bulk RNA sequencing of whole blood.	This paper	Zenodo: https://doi.org/10.5281/zenodo.6120249
**CBD-KEY-RNASEQ-WGCNA**: WGCNA analysis of whole blood RNaseq data. Module eigengenes and gene membership.	This paper	Zenodo: https://doi.org/10.5281/zenodo.6120249
**CBD-KEY-SDA**: Input data and results from tensor and matrix decomposition (SDA) analyses.	This paper	Zenodo: https://doi.org/10.5281/zenodo.6120249
**CBD-KEY-SIMON**: Processed datasets generated from key data modalities for machine learning analyses using SIMON to distinguish COVID-19 and sepsis	This paper	Zenodo: https://doi.org/10.5281/zenodo.6120249
**CBD-KEY-SNF**: Preprocessed/normalized inputs and results matrices from similarity network fusion (SNF) analysis	This paper	Zenodo: https://doi.org/10.5281/zenodo.6120249
**RAW datasets**		N/A
**CBD-RAW-CLINVAR**: Anonymized patient metadata, data dictionary.	This paper	EGAD00001007931
**CBD-RAW-CYTOF-MYELOID**: Single cell resolution mass cytometry (CyTOF) data generated from whole blood and granulocyte depleted whole blood, with a focus on myeloid populations. Dataset contains ungated, raw data files in fcs format and the list of antibodies used.	This paper	Zenodo: https://doi.org/10.5281/zenodo.6120249
**CBD-RAW-CYTOF-WB**: Single cell resolution mass cytometry (CyTOF) data generated from whole blood. Dataset contains ungated, raw data files in fcs format and the list of antibodies used.	This paper	Zenodo: https://doi.org/10.5281/zenodo.6120249
**CBD-RAW-CYTOF-WB-D**: Single cell resolution mass cytometry (CyTOF) data generated from granulocyte depleted whole blood. Dataset contains ungated, raw data files in fcs format and the list of antibodies used.	This paper	Zenodo: https://doi.org/10.5281/zenodo.6120249
**CBD-RAW-FACS**: Flow cytometry data from PBMC cells. Dataset contains .fcs files and FlowJo workspaces from FACS data.	This paper	Zenodo: https://doi.org/10.5281/zenodo.6120249
**CBD-RAW-REPERTOIRE-B**: FASTQ and filtered FASTA files for B cell receptor sequencing.	This paper	EGAD00001007960
**CBD-RAW-REPERTOIRE-T**: FASTQ and filtered FASTA files for T cell receptor sequencing.	This paper	EGAD00001007961
**CBD-RAW-RNASEQ**: Bulk RNA-seq data from whole blood.	This paper	EGAD00001007957
**CBD-RAW-RNASEQ-MINI**: SmartSeq2 RNA-seq data from 16 samples.	This paper	EGAD00001007932
**CBD-RAW-SC-ADT**: 10X Single-Cell Features Barcode (CITE-seq). Raw Illumina sequencing data and CellRanger BAM output files.	This paper	EGAD00001007962
**CBD-RAW-SC-ATAC**: 10X Single-Cell ATAC-Seq. Raw Illumina sequencing data from single-cell ATAC-Seq experiments	This paper	EGAD00001007963
**CBD-RAW-SC-GEX**: 10X Single-Cell Gene Expression. Raw Illumina sequencing data and CellRanger BAM output files.	This paper	EGAD00001008007
**CBD-RAW-SC-VDJ-B**: 10X Single-Cell VDJ BCR. Raw Illumina sequencing data.	This paper	EGAD00001007964
**CBD-RAW-SC-VDJ-T**: 10X Single-Cell VDJ TCR. Raw Illumina sequencing data.	This paper	EGAD00001007965
**Study accession (EGA)**		N/A
COMBAT Consortium	All linked datasets	EGAS00001005493
**Study website**		N/A
COMBAT Consortium	This paper	https://www.combat.ox.ac.uk/

**RT primers for TCR analysis**		

Primer: FT_TRBC_RT Oligo sequence (5′ to 3′): CAGCACGCTTCAGGCTNNNNTNNNNTNNNN AGATCTCTGCTTCTGATGGCTC	This paper	N/A

**PCR primers for TCR analysis**		

Primer: FT_TRBV3 oligo sequence (5′ to 3′): GACACAGCCGTTTCCCAGA	This paper	N/A
Primer: FT_TRBV4 oligo sequence (5′ to 3′): ACKGRAGTTACSCAGACA	This paper	N/A
Primer: FT_TRBV5 oligo sequence (5′ to 3′): RCTGGAGTCACHCAAAST	This paper	N/A
Primer: FT_TRBV7 oligo sequence (5′ to 3′): TGCTGGKRTCACYCAG	This paper	N/A
Primer: FT_TRBV7 oligo sequence (5′ to 3′): WDCTGGAGTYTCCCA	This paper	N/A
Primer: FT_TRBV9 oligo sequence (5′ to 3′): GATTCTGGAGTCACACAAA	This paper	N/A
Primer: FT_TRBV10 oligo sequence (5′ to 3′): GATGCTGRAATCACCCAGA	This paper	N/A
Primer: FT_TRBV11 oligo sequence (5′ to 3′): GAAGCTGRAGTGGYYCAGT	This paper	N/A
Primer: FT_TRBV12 oligo sequence (5′ to 3′): GATGCTRGAGTYAYCCAG	This paper	N/A
Primer: FT_TRBV13B oligo sequence (5′ to 3′): GCTGCTGGAGTCATCCAGT	This paper	N/A
Primer: FT_TRBV14 oligo sequence (5′ to 3′): GAAGCTGGAGTTACTCAGT	This paper	N/A
Primer: FT_TRBV15 oligo sequence (5′ to 3′): GATGCCATGGTCATCCAGA	This paper	N/A
Primer: FT_TRBV16 oligo sequence (5′ to 3′): GGTGAAGAAGTCGCCCAGA	This paper	N/A
Primer: FT_TRBV19 oligo sequence (5′ to 3′): GATGGTGGAATCACTCAGT	This paper	N/A
Primer: FT_TRBV18B oligo sequence (5′ to 3′): AATGCCGGCGTCATGCAGA	This paper	N/A
Primer: FT_TRBV20 oligo sequence (5′ to 3′): GGTGCTGTCGTCTCTCAAC	This paper	N/A
Primer: FT_TRBV24 oligo sequence (5′ to 3′): GATGCTGATGTTACCCAGA	This paper	N/A
Primer: FT_TRBV25 oligo sequence (5′ to 3′): GAAGCTGACATCTACCAGA	This paper	N/A
Primer: FT_TRBV27 oligo sequence (5′ to 3′): GAAGCCCAAGTGACCCAGA	This paper	N/A
Primer: FT_TRBV28 oligo sequence (5′ to 3′): GATGTGAAAGTAACCCAGA	This paper	N/A
Primer: FT_TRBV29 oligo sequence (5′ to 3′): AGTGCTGTCATCTCTC	This paper	N/A
Primer: FT_TRBV30 oligo sequence (5′ to 3′): TCTCAGACTATTCATCAAT	This paper	N/A
Primer: CNUS2_BC1 oligo sequence (5′ to 3′): CGCTCTTCCGATCTTaggtattCAGCACGCTTCAGGCT	This paper	N/A
Primer: CNUS2_BC2 oligo sequence (5′ to 3′): CGCTCTTCCGATCTTattaaggCAGCACGCTTCAGGCT	This paper	N/A
Primer: CNUS2_BC3 oligo sequence (5′ to 3′): CGCTCTTCCGATCTTtaattctCAGCACGCTTCAGGCT	This paper	N/A
Primer: CNUS2_BC4 oligo sequence (5′ to 3′): CGCTCTTCCGATCTTataagctCAGCACGCTTCAGGCT	This paper	N/A
Primer: CNUS2_BC5 oligo sequence (5′ to 3′): CGCTCTTCCGATCTTttatgatCAGCACGCTTCAGGCT	This paper	N/A
Primer: CNUS2_BC6 oligo sequence (5′ to 3′): CGCTCTTCCGATCTTgagttaaCAGCACGCTTCAGGCT	This paper	N/A
Primer: CNUS2_BC7 oligo sequence (5′ to 3′): CGCTCTTCCGATCTTattcttgCAGCACGCTTCAGGCT	This paper	N/A
Primer: CNUS2_BC8 oligo sequence (5′ to 3′): CGCTCTTCCGATCTTttagcttCAGCACGCTTCAGGCT	This paper	N/A
Primer: CNUS2_BC9 oligo sequence (5′ to 3′): CGCTCTTCCGATCTTatcttgcCAGCACGCTTCAGGCT	This paper	N/A
Primer: CNUS2_BC10 oligo sequence (5′ to 3′): CGCTCTTCCGATCTTtctccatCAGCACGCTTCAGGCT	This paper	N/A
Primer: CNUS2_BC11 oligo sequence (5′ to 3′): CGCTCTTCCGATCTTacctaggCAGCACGCTTCAGGCT	This paper	N/A
Primer: CNUS2_BC12 oligo sequence (5′ to 3′): CGCTCTTCCGATCTTttgatccCAGCACGCTTCAGGCT	This paper	N/A

**RT primers for BCR analysis**		

Primer: IGHA_human_BC oligo sequence (5′ to 3′): TGTCCAGCACGCTTCAGGCTNNNNTNNNNTNN NNGAYGACCACGTTCCCATCT	This paper	N/A
Primer: IGHM_human_BC oligo sequence (5′ to 3′): TGTCCAGCACGCTTCAGGCTNNNNTNNNNTNN NNTCGTATCCGACGGGGAATTC	This paper	N/A
Primer: IGHD_human_BC oligo sequence (5′ to 3′): TGTCCAGCACGCTTCAGGCTNNNNTNNNNTN NNNGGGCTGTTATCCTTTGGGTG	This paper	N/A
Primer: IGHE_human_BC oligo sequence (5′ to 3′): TGTCCAGCACGCTTCAGGCTNNNNTNNNNTN NNNAGAGTCACGGAGGTGGCATT	This paper	N/A
Primer: IGHG_human_BC oligo sequence (5′ to 3′): TGTCCAGCACGCTTCAGGCTNNNNTNNNNTN NNNAGTAGTCCTTGACCAGGCAG	This paper	N/A

**PCR primers for BCR analysis**		

Primer: VH1-FR1_F1 oligo sequence (5′ to 3′): AAGCAGTGGTATCAACGCAGAGTCGCAGGGGC CTCAGTGAAGGTCTCCTGCAAG (barcode 1)	This paper	N/A
Primer: VH2-FR1_F1 oligo sequence (5′ to 3′): AAGCAGTGGTATCAACGCAGAGTCGCAGGGT CTGGTCCTACGCTGGTGAAACCC (barcode 1)	This paper	N/A
Primer: VH3-FR1_F1 oligo sequence (5′ to 3′): AAGCAGTGGTATCAACGCAGAGTCGCAGGCTGG GGGGTCCCTGAGACTCTCCTG (barcode 1)	This paper	N/A
Primer: VH4-FR1_F1 oligo sequence (5′ to 3′): AAGCAGTGGTATCAACGCAGAGTCGCAGGCT TCGGAGACCCTGTCCCTCACCTG (barcode 1)	This paper	N/A
Primer: VH5-FR1_F1 oligo sequence (5′ to 3′): AAGCAGTGGTATCAACGCAGAGTCGCAGGC GGGGAGTCTCTGAAGATCTCCTGT (barcode 1)	This paper	N/A
Primer: VH6-FR1_F1 oligo sequence (5′ to 3′): AAGCAGTGGTATCAACGCAGAGTCGCAGGT CGCAGACCCTCTCACTCACCTGTG (barcode 1)	This paper	N/A
Primer: VH1-FR1_F2 oligo sequence (5′ to 3′): AAGCAGTGGTATCAACGCAGAGCTCTGCAGG CCTCAGTGAAGGTCTCCTGCAAG (barcode 2)	This paper	N/A
Primer: VH2-FR1_F2 oligo sequence (5′ to 3′): AAGCAGTGGTATCAACGCAGAGCTCTGCAGT CTGGTCCTACGCTGGTGAAACCC (barcode 2)	This paper	N/A
Primer: VH3-FR1_F2 oligo sequence (5′ to 3′): AAGCAGTGGTATCAACGCAGAGCTCTGCACT GGGGGGTCCCTGAGACTCTCCTG (barcode 2)	This paper	N/A
Primer: VH4-FR1_F2 oligo sequence (5′ to 3′): AAGCAGTGGTATCAACGCAGAGCTCTGCACT TCGGAGACCCTGTCCCTCACCTG (barcode 2)	This paper	N/A
Primer: VH5-FR1_F2 oligo sequence (5′ to 3′): AAGCAGTGGTATCAACGCAGAGCTCTGCAC GGGGAGTCTCTGAAGATCTCCTGT (barcode 2)	This paper	N/A
Primer: VH6-FR1_F2 oligo sequence (5′ to 3′): AAGCAGTGGTATCAACGCAGAGCTCTGCAT CGCAGACCCTCTCACTCACCTGTG (barcode 2)	This paper	N/A
Primer: VH1-FR1_F3 oligo sequence (5′ to 3′): AAGCAGTGGTATCAACGCAGAGCCTAGGTG GCCTCAGTGAAGGTCTCCTGCAAG (barcode 3)	This paper	N/A
Primer: VH2-FR1_F3 oligo sequence (5′ to 3′): AAGCAGTGGTATCAACGCAGAGCCTAGGTG TCTGGTCCTACGCTGGTGAAACCC (barcode 3)	This paper	N/A
Primer: VH3-FR1_F3 oligo sequence (5′ to 3′): AAGCAGTGGTATCAACGCAGAGCCTAGGTC TGGGGGGTCCCTGAGACTCTCCTG (barcode 3)	This paper	N/A
Primer: VH4-FR1_F3 oligo sequence (5′ to 3′): AAGCAGTGGTATCAACGCAGAGCCTAGGTC TTCGGAGACCCTGTCCCTCACCTG (barcode 3)	This paper	N/A
Primer: VH5-FR1_F3 oligo sequence (5′ to 3′): AAGCAGTGGTATCAACGCAGAGCCTAGGTC GGGGAGTCTCTGAAGATCTCCTGT (barcode 3)	This paper	N/A
Primer: VH6-FR1_F3 oligo sequence (5′ to 3′): AAGCAGTGGTATCAACGCAGAGCCTAGGTT CGCAGACCCTCTCACTCACCTGTG (barcode 3)	This paper	N/A
Primer: VH1-FR1_F4 oligo sequence (5′ to 3′): AAGCAGTGGTATCAACGCAGAGGGATCAAGG CCTCAGTGAAGGTCTCCTGCAAG (barcode 4)	This paper	N/A
Primer: VH2-FR1_F4 oligo sequence (5′ to 3′): AAGCAGTGGTATCAACGCAGAGGGATCAA GTCTGGTCCTACGCTGGTGAAACCC (barcode 4)	This paper	N/A
Primer: VH3-FR1_F4 oligo sequence (5′ to 3′): AAGCAGTGGTATCAACGCAGAGGGATCAAC TGGGGGGTCCCTGAGACTCTCCTG (barcode 4)	This paper	N/A
Primer: VH4-FR1_F4 oligo sequence (5′ to 3′): AAGCAGTGGTATCAACGCAGAGGGATCAAC TTCGGAGACCCTGTCCCTCACCTG (barcode 4)	This paper	N/A
Primer: VH5-FR1_F4 oligo sequence (5′ to 3′): AAGCAGTGGTATCAACGCAGAGGGATCAA CGGGGAGTCTCTGAAGATCTCCTGT (barcode 4)	This paper	N/A
Primer: VH6-FR1_F4 oligo sequence (5′ to 3′): AAGCAGTGGTATCAACGCAGAGGGATCAAT CGCAGACCCTCTCACTCACCTGTG (barcode 4)	This paper	N/A
Primer: VH1-FR1_F5 oligo sequence (5′ to 3′): AAGCAGTGGTATCAACGCAGAGGCAAGATG GCCTCAGTGAAGGTCTCCTGCAAG (barcode 5)	This paper	N/A
Primer: VH2-FR1_F5 oligo sequence (5′ to 3′): AAGCAGTGGTATCAACGCAGAGGCAAGATGT CTGGTCCTACGCTGGTGAAACCC (barcode 5)	This paper	N/A
Primer: VH3-FR1_F5 oligo sequence (5′ to 3′): AAGCAGTGGTATCAACGCAGAGGCAAGATCT GGGGGGTCCCTGAGACTCTCCTG (barcode 5)	This paper	N/A
Primer: VH4-FR1_F5 oligo sequence (5′ to 3′): AAGCAGTGGTATCAACGCAGAGGCAAGATC TTCGGAGACCCTGTCCCTCACCTG (barcode 5)	This paper	N/A
Primer: VH5-FR1_F5 oligo sequence (5′ to 3′): AAGCAGTGGTATCAACGCAGAGGCAAGATC GGGGAGTCTCTGAAGATCTCCTGT (barcode 5)	This paper	N/A
Primer: VH6-FR1_F5 oligo sequence (5′ to 3′): AAGCAGTGGTATCAACGCAGAGGCAAGATT CGCAGACCCTCTCACTCACCTGTG (barcode 5)	This paper	N/A
Primer: VH1-FR1_F6 oligo sequence (5′ to 3′): AAGCAGTGGTATCAACGCAGAGATGGAGAG GCCTCAGTGAAGGTCTCCTGCAAG (barcode 6)	This paper	N/A
Primer: VH2-FR1_F6 oligo sequence (5′ to 3′): AAGCAGTGGTATCAACGCAGAGATGGAGAG TCTGGTCCTACGCTGGTGAAACCC (barcode 6)	This paper	N/A
Primer: VH3-FR1_F6 oligo sequence (5′ to 3′): AAGCAGTGGTATCAACGCAGAGATGGAGAC TGGGGGGTCCCTGAGACTCTCCTG (barcode 6)	This paper	N/A
Primer: VH4-FR1_F6 oligo sequence (5′ to 3′): AAGCAGTGGTATCAACGCAGAGATGGAGAC TTCGGAGACCCTGTCCCTCACCTG (barcode 6)	This paper	N/A
Primer: VH5-FR1_F6 oligo sequence (5′ to 3′): AAGCAGTGGTATCAACGCAGAGATGGAGAC GGGGAGTCTCTGAAGATCTCCTGT (barcode 6)	This paper	N/A
Primer: VH6-FR1_F6 oligo sequence (5′ to 3′): AAGCAGTGGTATCAACGCAGAGATGGAGAT CGCAGACCCTCTCACTCACCTGTG (barcode 6)	This paper	N/A
Primer: VH1-FR1_F7 oligo sequence (5′ to 3′): AAGCAGTGGTATCAACGCAGAGCTCGATGG GCCTCAGTGAAGGTCTCCTGCAAG (barcode 7)	This paper	N/A
Primer: VH2-FR1_F7 oligo sequence (5′ to 3′): AAGCAGTGGTATCAACGCAGAGCTCGATGG TCTGGTCCTACGCTGGTGAAACCC (barcode 7)	This paper	N/A
Primer: VH3-FR1_F7 oligo sequence (5′ to 3′): AAGCAGTGGTATCAACGCAGAGCTCGATGCT GGGGGGTCCCTGAGACTCTCCTG (barcode 7)	This paper	N/A
Primer: VH4-FR1_F7 oligo sequence (5′ to 3′): AAGCAGTGGTATCAACGCAGAGCTCGATGC TTCGGAGACCCTGTCCCTCACCTG (barcode 7)	This paper	N/A
Primer: VH5-FR1_F7 oligo sequence (5′ to 3′): AAGCAGTGGTATCAACGCAGAGCTCGATGC GGGGAGTCTCTGAAGATCTCCTGT (barcode 7)	This paper	N/A
Primer: VH6-FR1_F7 oligo sequence (5′ to 3′): AAGCAGTGGTATCAACGCAGAGCTCGATGTC GCAGACCCTCTCACTCACCTGTG (barcode 7)	This paper	N/A
Primer: VH1-FR1_F8 oligo sequence (5′ to 3′): AAGCAGTGGTATCAACGCAGAGGCTCGAAG GCCTCAGTGAAGGTCTCCTGCAAG (barcode 8)	This paper	N/A
Primer: VH2-FR1_F8 oligo sequence (5′ to 3′): AAGCAGTGGTATCAACGCAGAGGCTCGAAGT CTGGTCCTACGCTGGTGAAACCC (barcode 8)	This paper	N/A
Primer: VH3-FR1_F8 oligo sequence (5′ to 3′): AAGCAGTGGTATCAACGCAGAGGCTCGAAC TGGGGGGTCCCTGAGACTCTCCTG (barcode 8)	This paper	N/A
Primer: VH4-FR1_F8 oligo sequence (5′ to 3′): AAGCAGTGGTATCAACGCAGAGGCTCGAAC TTCGGAGACCCTGTCCCTCACCTG (barcode 8)	This paper	N/A
Primer: VH5-FR1_F8 oligo sequence (5′ to 3′): AAGCAGTGGTATCAACGCAGAGGCTCGAACG GGGAGTCTCTGAAGATCTCCTGT (barcode 8)	This paper	N/A
Primer: VH6-FR1_F8 oligo sequence (5′ to 3′): AAGCAGTGGTATCAACGCAGAGGCTCGAATC GCAGACCCTCTCACTCACCTGTG (barcode 8)	This paper	N/A
Primer: VH1-FR1_F9 oligo sequence (5′ to 3′): AAGCAGTGGTATCAACGCAGAGACCAACTGG CCTCAGTGAAGGTCTCCTGCAAG (barcode 9)	This paper	N/A
Primer: VH2-FR1_F9 oligo sequence (5′ to 3′): AAGCAGTGGTATCAACGCAGAGACCAACTGTC TGGTCCTACGCTGGTGAAACCC (barcode 9)	This paper	N/A
Primer: VH3-FR1_F9 oligo sequence (5′ to 3′): AAGCAGTGGTATCAACGCAGAGACCAACTC TGGGGGGTCCCTGAGACTCTCCTG (barcode 9)	This paper	N/A
Primer: VH4-FR1_F9 oligo sequence (5′ to 3′): AAGCAGTGGTATCAACGCAGAGACCAACT CTTCGGAGACCCTGTCCCTCACCTG (barcode 9)	This paper	N/A
Primer: VH5-FR1_F9 oligo sequence (5′ to 3′): AAGCAGTGGTATCAACGCAGAGACCAAC TCGGGGAGTCTCTGAAGATCTCCTGT (barcode 9)	This paper	N/A
Primer: VH6-FR1_F9 oligo sequence (5′ to 3′): AAGCAGTGGTATCAACGCAGAGACCAACT TCGCAGACCCTCTCACTCACCTGTG (barcode 9)	This paper	N/A
Primer: VH1-FR1_F10 oligo sequence (5′ to 3′): AAGCAGTGGTATCAACGCAGAGCCGGTA CGGCCTCAGTGAAGGTCTCCTGCAAG (barcode 10)	This paper	N/A
Primer: VH2-FR1_F10 oligo sequence (5′ to 3′): AAGCAGTGGTATCAACGCAGAGCCGGTA CGTCTGGTCCTACGCTGGTGAAACCC (barcode 10)	This paper	N/A
Primer: VH3-FR1_F10 oligo sequence (5′ to 3′): AAGCAGTGGTATCAACGCAGAGCCGGTA CCTGGGGGGTCCCTGAGACTCTCCTG (barcode 10)	This paper	N/A
Primer: VH4-FR1_F10 oligo sequence (5′ to 3′): AAGCAGTGGTATCAACGCAGAGCCGGTA CCTTCGGAGACCCTGTCCCTCACCTG (barcode 10)	This paper	N/A
Primer: VH5-FR1_F10 oligo sequence (5′ to 3′): AAGCAGTGGTATCAACGCAGAGCCGGTA CCGGGGAGTCTCTGAAGATCTCCTGT (barcode 10)	This paper	N/A
Primer: VH6-FR1_F10 oligo sequence (5′ to 3′): AAGCAGTGGTATCAACGCAGAGCCGGTA CTCGCAGACCCTCTCACTCACCTGTG (barcode 10)	This paper	N/A
Primer: VH1-FR1_F11 oligo sequence (5′ to 3′): AAGCAGTGGTATCAACGCAGAGAACTC CGGGCCTCAGTGAAGGTCTCCTGCAAG (barcode 11)	This paper	N/A
Primer: VH2-FR1_F11 oligo sequence (5′ to 3′): AAGCAGTGGTATCAACGCAGAGAACTCC GGTCTGGTCCTACGCTGGTGAAACCC (barcode 11)	This paper	N/A
Primer: VH3-FR1_F11 oligo sequence (5′ to 3′): AAGCAGTGGTATCAACGCAGAGAACTC CGCTGGGGGGTCCCTGAGACTCTCCTG (barcode 11)	This paper	N/A
Primer: VH4-FR1_F11 oligo sequence (5′ to 3′): AAGCAGTGGTATCAACGCAGAGAACTC CGCTTCGGAGACCCTGTCCCTCACCTG (barcode 11)	This paper	N/A
Primer: VH5-FR1_F11 oligo sequence (5′ to 3′): AAGCAGTGGTATCAACGCAGAGAACTC CGCGGGGAGTCTCTGAAGATCTCCTGT (barcode 11)	This paper	N/A
Primer: VH6-FR1_F11 oligo sequence (5′ to 3′): AAGCAGTGGTATCAACGCAGAGAACTC CGTCGCAGACCCTCTCACTCACCTGTG (barcode 11)	This paper	N/A
Primer: VH1-FR1_F12 oligo sequence (5′ to 3′): AAGCAGTGGTATCAACGCAGAGTTGAA GTGGCCTCAGTGAAGGTCTCCTGCAAG (barcode 12)	This paper	N/A
Primer: VH2-FR1_F12 oligo sequence (5′ to 3′): AAGCAGTGGTATCAACGCAGAGTTGAA GTGTCTGGTCCTACGCTGGTGAAACCC (barcode 12)	This paper	N/A
Primer: VH3-FR1_F12 oligo sequence (5′ to 3′): AAGCAGTGGTATCAACGCAGAGTTGAAG TCTGGGGGGTCCCTGAGACTCTCCTG (barcode 12)	This paper	N/A
Primer: VH4-FR1_F12 oligo sequence (5′ to 3′): AAGCAGTGGTATCAACGCAGAGTTGAA GTCTTCGGAGACCCTGTCCCTCACCTG (barcode 12)	This paper	N/A
Primer: VH5-FR1_F12 oligo sequence (5′ to 3′): AAGCAGTGGTATCAACGCAGAGTTGAAG TCGGGGAGTCTCTGAAGATCTCCTGT (barcode 12)	This paper	N/A
Primer: VH6-FR1_F12 oligo sequence (5′ to 3′): AAGCAGTGGTATCAACGCAGAGTTGAA GTTCGCAGACCCTCTCACTCACCTGTG (barcode 12)	This paper	N/A
Primer: CNU Barcode primer_S oligo sequence (5′ to 3′): TGTCCAGCACGCTTCAGGCT	This paper	N/A

Software and algorithms		

ALDEx2	(Fernandes et al., 2013)	v1.18.0
ArchR	(Granja et al., 2021)	v0.9.3 https://www.archrproject.com/
AUCell	(Aibar et al., 2017)	v1.12.0
BBKNN	(Polański et al., 2020)	https://github.com/Teichlab/bbknn
BLAST	(Altschul et al., 1990)	https://blast.ncbi.nlm.nih.gov/Blast.cgi
CATALYST	(Nowicka et al., 2017)	v1.10.3
CD-HIT		https://github.com/thomasp85/FindMyFriends
Cell Ranger	10x genomics	v3.1.0
Cell Ranger ATAC	10x genomics	https://support.10xgenomics.com/single-cell-atac/software/overview/welcome
Cellranger VDJ	10x genomics	v3.1.0
Clinical Knowledge Graph	(Santos et al., 2020)	https://doi.org/10.1101/2020.05.09.084897
corrplot	Wei	v0.84 https://github.com/taiyun/corrplot
CoV-AbDab	(Raybould et al., 2021)	http://opig.stats.ox.ac.uk/webapps/covabdab/
CyTOF Software		v7.0 http://www.fluidigm.com/products-services/software
CytoNorm	(Van Gassen et al., 2020)	v0.0.5
Cytoscape	(Shannon et al., 2003)	v3.8.0
Cytosplore	(van Unen et al., 2017)	https://www.cytosplore.org/
demuxlet	(Kang et al., 2018)	v2 https://github.com/statgen/demuxlet
diffcyt	(Weber et al., 2019)	v1.8.8
edgeR	(Robinson et al., 2010)	v3.28.1 https://bioconductor.org/packages/release/bioc/html/edgeR.html
EmptyDroplets	(Lun et al., 2019)	v1.8.0
Entropy	(Hausser, 2009)	https://strimmerlab.github.io
eXploring Genomic Relations (XGR)	(Fang et al., 2016b)	http://galahad.well.ox.ac.uk/XGR
Fastcluster	(Mulner, 2013)	v1.1.25
FastQC	(Andrews, 2010)	v0.11.9 https://github.com/s-andrews/FastQC
featureCounts	(Liao et al., 2014)	v1.6.4
fgsea	(Korotkevich et al., 2021)	https://bioconductor.org/packages/release/bioc/html/fgsea.html
FlowJo	BD Biosciences	v10.6 https://www.flowjo.com
Fragpipe	(Yu et al., 2021)	v13.0
GATK variant calling	(Van der Auwera and O’Connor, 2020)	v4.1.7.0
ggraph	Pedersen	v2.05 https://github.com/thomasp85/ggraph
gsfisher	(Croft et al., 2019)	https://github.com/sansomlab/gsfisher
Harmony	(Korsunsky et al., 2019)	https://github.com/immunogenomics/harmony
HTSlib v1.10.2	Samtools	http://www.htslib.org/
imagesc	MATLAB	https://uk.mathworks.com/help/matlab/ref/imagesc.html
IMGT database	(Lefranc, 2011)	https://www.imgt.org/
IMGT V-QUEST	(Giudicelli et al., 2004)	https://www.imgt.org/IMGTindex/V-QUEST.php
InstantClue	(Nolte et al., 2018)	v0.5.3 http://www.instantclue.uni-koeln.de/
IonQuant	(Yu et al., 2020)	https://ionquant.nesvilab.org/
KeplerMapper	(van Veen et al., 2019)	https://openprojectrepo.com/project/scikit-tda-kepler-mapper-python-data-validation
Limma	(Ritchie et al., 2015)	https://bioconductor.org/packages/release/bioc/html/limma.html
MATLAB		https://uk.mathworks.com/help/matlab/
Matplotlib	(Hunter, 2007)	https://matplotlib.org/
MSFragger	(Kong et al., 2017)	v3.0
MSigDB	(Subramanian et al., 2005)	https://www.gsea-msigdb.org/gsea/msigdb/index.jsp
Pandas		v1.2.4 https://pandas.pydata.org/
Pegasus	(Li et al., 2020)	https://pegasus.readthedocs.io/en/stable/index.html
PeptideProphet	(Keller et al., 2002)	Embedded in Fragpipe
Perseus 1.6.14.0	(Tyanova and Cox, 2018)	https://maxquant.net/perseus/
Philosopher	(da Veiga Leprevost et al., 2020)	v3.2.9
Picard	http://broadinstitute.github.io/picard/	v2.23 https://github.com/broadinstitute/picard
prcomp		v3.6.2 https://www.rdocumentation.org/packages/stats/versions/3.6.2/topics/prcomp
Priority Index (Pi)	(Fang et al., 2016a)	http://galahad.well.ox.ac.uk/Pi
ProteinProphet	(Nesvizhskii et al., 2003)	Embedded in Fragpipe
Python		v3.8.2 https://www.python.org/
QTLtools	(Delaneau et al., 2017)	https://qtltools.github.io/qtltools/
QUASR	(Watson et al., 2013)	https://sourceforge.net/projects/quasr/
R Studio and R environment	The R project for Statistical Computing	https://www.rstudio.com/ and https://cran.r-project.org/
RNASeQC	(DeLuca et al., 2012)	v2.3.5 https://github.com/getzlab/rnaseqc
Scanorama	(Hie et al., 2019)	https://github.com/brianhie/scanorama
Scanpy	(Wolf et al., 2018)	https://github.com/theislab/scanpy
Scater	(McCarthy et al., 2017)	v3.12 http://bioconductor.org/packages/release/bioc/html/scater.html
Scikit-learn	(Pedregosa et al., 2011)	https://github.com/scikit-learn/scikit-learn
Scipy	(Virtanen et al., 2020)	https://scipy.org/
ScVelo	(Bergen et al., 2020)	v0.1.24 https://github.com/theislab/scvelo
Sparse Decomposition of Arrays	(Hore et al., 2016)	https://jmarchini.org/software/#sda
Seaborn	Waskom	v0.11.1 https://seaborn.pydata.org/
Seurat	(Stuart et al., 2019)	v3.9.9.9010
SIMON	(Tomic et al., 2019)	https://genular.org/
singleR	(Aran et al., 2019)	https://github.com/dviraran/SingleR
STAR	(Dobin et al., 2013)	v2.7.3
stringdist	(van der Loo, 2014)	https://github.com/markvanderloo/stringdist
Survival	(Therneau and Grambsch, 2000)	v3.2-11
Survminer	Kassambara	v0.4.8
sva R	(Johnson et al., 2007; Leek et al., 2012)	v3.36.0
Swissprot human Proteome database + SARSCov2		https://www.uniprot.org/ retrieved 17/07/2020
TrimGalore	Krueger	v0.6.2 https://github.com/FelixKrueger/TrimGalore
ttest2	MATLAB	https://uk.mathworks.com/help/stats/ttest2.heml
UMAP	McInnes, Healy, Melville	arXiv:1802.03426v2
Velocyto	(La Manno et al., 2018)	http://velocyto.org/
Vireo	(Huang et al., 2019)	v0.4.0 https://huangyh09.github.io/vireo-manual/about.html
WGCNA	(Langfelder and Horvath, 2008)	https://horvath.genetics.ucla.edu/html/CoexpressionNetwork/Rpackages/WGCNA/
xCell	(Aran et al., 2017)	https://github.com/dviraran/xCell

### Resource availability

#### Lead contact

Further information and requests for resources and reagents should be directed to and will be fulfilled by Julian Knight (julian.knight@well.ox.ac.uk).

#### Materials availability

This study did not generate new unique reagents.

### Experimental model and subject details

#### Cohorts

The study was designed to allow deep molecular, multi-omic and immunological profiling of COVID-19 in peripheral blood at the outset of the pandemic in the United Kingdom during a public health emergency. Samples used were derived from multiple sources to allow comparison between adult patients with varying severities of COVID-19 and comparator disease or health states. A summary of the cohorts included in this study is provided below followed by a description of the methods used to define clinical parameters and handling of the blood samples. Demographic information and clinical phenotyping for all study subjects is summarized in Table S1, together with numbers of patients/samples assayed by cohort.

##### Hospitalized COVID-19 patients

Patients admitted to Oxford University Hospitals NHS Foundation Trust, UK were recruited into the Sepsis Immunomics study (a prospective observational cohort study applying an integrated immune -omic approach to understand why some patients have a severe response to infection) if they were found to have a syndrome consistent with COVID-19 and a positive test for SARS-CoV-2 using reverse transcriptase polymerase chain reaction (RT-PCR) from an upper respiratory tract (nose/throat) swab tested in accredited laboratories, with the majority of patients co-recruited using the ISARIC/WHO Clinical Characterization Protocol for Severe Emerging Infections. Written informed consent was obtained from adults or personal/nominated consultees for patients lacking capacity, with retrospective consent obtained from the patient once capacity was regained. Ethical approval was given by the South Central–Oxford C Research Ethics Committee (REC) in England for Sepsis Immunomics (REC reference 19/SC/0296). For ISARIC WHO Clinical Characterization Protocol for Severe Emerging Infections, ethical approval was given by the South Central-Oxford C Research Ethics Committee in England (REC reference 13/SC/0149), the Scotland A Research Ethics Committee (Ref 20/SS/0028), and the WHO Ethics Review Committee (RPC571 and RPC572, 25 April 2013). Patients had whole blood sampled on days 1, 3 and 5 of either hospital or intensive care admission and were recruited during the SARS-COV-2 pandemic between 13 March and 28 April 2020. A selection of survivors were approached and asked to provide samples with consent under both research protocols at day 28 or more following discharge from hospital.

##### Healthcare workers with COVID-19

In order to provide a time of symptom matched set of samples from individuals with mild COVID-19 disease in the community, healthcare workers based at Oxford University Hospitals NHS Foundation Trust, UK with symptoms consistent with mild COVID-19 and a positive test for SARS-CoV-2 using RT-PCR from an upper respiratory tract (nose/throat) swab tested in an accredited lab were recruited into the Gastro-intestinal illness in Oxford: COVID substudy [Sheffield REC, reference: 16/YH/0247]. Individuals were consented and sampled at or after 7 days from the start of symptoms when the participants were returning to work.

##### Hospitalized patients with all-cause sepsis

In order to include samples from patients with sources of severe sepsis other than COVID-19, patients older than 18 years of age admitted to Oxford University Hospitals NHS Foundation Trust, UK with symptoms and signs of established sepsis (suspected infection with an acute change in total Sequential Organ Failure Assessment (SOFA) score of ≥2 points or a change in quick SOFA score by ≥2 points) were consented into the Sepsis Immunomics project [Oxford REC C, reference:19/SC/0296]) between 12 October 2019 and 13 March 2020. Patients were sampled on days 1, 3 and 5 of either hospital or intensive care admission and a selection of survivors were sampled, with additional consent at up to 6 months post-discharge.

##### Healthy volunteers

Following advertisement, interested individuals 55 years or over and self-reporting as healthy were consented and recruited into the Genetic diversity and gene expression in white blood cells study [South Central Oxford REC B, reference 06/Q1605/55]. Blood samples were collected on one occasion only.

##### Critically unwell patients with COVID-19 and influenza in London

In order to provide comparator samples from patients critically unwell with influenza, samples were included from patients ≥18 years old managed in an intensive care unit (ICU) for ≥24 hours requiring ventilator support with either a diagnosis of COVID-19 or severe influenza using the Aspergillosis in patients with severe influenza (AspiFlu ISRCTN51287266) study [Wales REC 5, reference 19/WA/0310] (Youngs et al., 2021). Patients had blood sampled within 72 hours of enrolment that may occur within 7 days of admission onto ICU. Patients were recruited at one of three UK units including St George’s University Hospitals NHS Foundation Trust, Guy’s and St Thomas’ NHS Foundation Trust and King’s College Hospital NHS Foundation Trust. PCR diagnostics for influenza or SARS-CoV2 performed by accredited laboratories were used for diagnosis.

#### Clinical phenotyping

##### Clinical data capture

Healthy volunteers and healthcare workers with COVID-19 had age, sex and, where possible, self-reported ethnic background information collected. Information on previous medical history was not collected or stored for the healthy volunteer cohort but participants were invited on the basis of their self-reporting being ‘healthy’ and were deemed capable of self-presenting for study assessment. Healthcare workers with COVID-19 were asked for the date of onset of symptoms (‘when they started to feel unwell’) and the number of days between this date and sampling was calculated for every participant. Any healthcare worker who was admitted to hospital or required oxygen had this information collected and retained and defined as maximal severity of illness using the World Health Organization Criteria (see below).

A range of clinical data was collected and stored for downstream analysis using structured methodology from the hospitalized patients included in the study (COVID-19, all-cause sepsis and influenza).

##### Patient demographics

Sex and ethnicity were captured using electronic healthcare records (EHR). Age was calculated at the time of sampling or maximal severity of illness using dates of birth registered in EHR.

##### Patient medical history and risk factors

Smoking status was derived from clinical clerking or direct patient or next-of-kin questioning where possible. Estimated or calculated weight was available for the vast majority of patients. In London COVID-19 and influenza patients, body-mass index (BMI) was calculated through availability of estimated or measured height. In Oxford patients, height was rarely available and so BMI was estimated using sex and weight and correlation with measured BMI was acceptable in cases where BMI was available. BMI of 25 or over was defined as overweight. Index of multiple deprivation (IMD) quintile and Charlson comorbidity indices (2012 definitions) were derived from the EHR in Oxford and were not available for London patients. Previous medical problems in patients admitted to Oxford hospitals were defined using NHS UK Read Codes Clinical Terms Version 3 and converted into ICD-10 codes using ‘TRUD’ (https://isd.digital.nhs.uk/trud/user/guest/group/0/pack/9). Pre-existing medical conditions defined through ICD-10 were defined through the consensus of at least one clinical opinion and in cases where there was uncertainty if a code was assigned to an acute presentation, pre-existing conditions were only assigned if they were present in the first episode of an admission, and not if they were only present in later admission episodes. For patients recruited in London, pre-existing diseases were defined by the study teams using clinical records and patient or relative questioning.

After defining pre-existing ICD-10 codes or reported diagnoses, patients were classified as having the following conditions: hypertension, chronic respiratory disease (not explicitly available for London patients), asthma (not explicitly available for London patients but chronic obstructive pulmonary disease was), chronic cardiovascular disease, diabetes, hematological malignancy, other malignancy, liver disease, neurological disease (not available for London patients), chronic kidney disease, solid organ transplant, rheumatological condition (not available for London patients), significant immunosuppression (not available for London patients) and stroke / dementia (not available for London patients). An OpenSafely (OS) score was calculated for all patients using these classifications combined with BMI and IMD (Williamson et al., 2020). An adjusted OS score was calculated for the London cases that did not include some missing pre-morbid conditions and IMD quintile and the correlation was very high with the standard OS score (r = 0.98).

##### Admission and disease timescales

Length of hospital stay was defined using hospital records for all hospitalized patients. Length of intensive care admission and duration of vasopressor, ventilation or, where appropriate extracorporeal membrane oxygenation (ECMO) and/or renal replacement therapy were all defined for hospitalized patients using EHR. All intervention and maximal severity time points were defined according to the date of onset of symptoms for each patient. This was defined by at least 2 independent clinicians through review of the clinical notes or direct questioning of the patient according to any unusual symptoms related to the current clinical condition. COVID-19 was defined by presence of at least one symptom consistent with COVID-19 and a positive microbiological test. Patients without symptoms were not approached for recruitment. Time between symptom onset and sampling was measured in days. All London patients were classified as critical requiring mechanical ventilation. No specific physiological observations were available for the London patients at the time of sampling and were only available at the time of admission onto ICU for calculation of the Acute Physiology and Chronic Health Evaluation (APACHE) and SOFA scores. Physiological observations were available from EHR for all hospitalized Oxford patients and were defined on the day of sampling at midday or the closest time before or after midday for each patient and time point including oxygen saturation, delivered fraction of inspired oxygen (either exact or estimated depending on delivery method: 0.24 for nasal cannula, 0.28 for simple face mask, 0.8 for non-rebreathe mask), pulse rate and blood pressure. Oxygen saturation: fraction of inspired oxygen ratio (SaO_2_:FiO_2_) was calculated as a proxy for partial pressure of oxygen:FiO_2_ ratio in the absence of complete arterial blood sampling for all patients. Days of oxygen therapy were defined in relation to sampling and maximal illness and total hospital stay for hospitalized COVID-19 patients where 2 or more time points of oxygen therapy were recorded. Fever was defined as 38.0°C and, if present at midday, was defined at present at time of sampling. Highest measured temperature 24 hours prior to midday was also captured using EHR for every time point and the presence of ‘persistent fever’ more measures of temperature ≥ 38°C in 24 hours before midday than temperatures recorded < 38°C was also recorded for all sampling time points in hospitalized Oxford patients. In those patients where persistent fever was observed the time in days between onset of symptoms and end of persistent fever was calculated.

Maximal severity was defined for Oxford COVID-19 patients using SaO_2_:FiO_2_ ratio and the date of lowest value was defined through manual inspection of longitudinal physiological parameters available through EHR. The individual levels of SaO_2_, method and exact or estimated concentration of oxygen delivered and additional recruitment strategies such as paralysis or proning were captured. In cases where death occurred, this was defined as maximum severity of illness and time between symptom onset and maximum severity was defined thereafter.

All together this information was used to understand the illness response and severity including (1) to generate a correlation matrix of clinical features, severity scores and markers illness response in the hospitalized COVID-19 cohort (Data S2); (2) to perform unsupervised machine learning (see section Statistical and unsupervised analysis of clinical phenotyping using clustering; also Data S2); (3) to define WHO categorical (mild as no oxygen, severe as ≤ 93% oxygen saturation, and critical as requiring mechanical ventilation) and ordinal scales (https://www.who.int/blueprint/priority-diseases/key-action/COVID-19_Treatment_Trial_Design_Master_Protocol_synopsis_Final_18022020.pdf) for each time point of sampling and maximal severity of illness and these times were all aligned in days from onset of symptoms (summarized in Data S2). Severity was also classified according to the WHO Ordinal scale (https://www.who.int/blueprint/priority-diseases/key-action/COVID-19_Treatment_Trial_Design_Master_Protocol_synopsis_Final_18022020.pdf) and Sequential Organ Failure Assessment score (Ferreira et al., 2001) together with the extent of lung disease and compromise (SOFA oxygenation score).

##### Other clinical and therapeutic tests and interventions

Full blood count differentials (hemoglobin concentration, platelet count, total white cell, neutrophil, lymphocyte, monocyte and eosinophil counts), highest lactate, C-reactive protein (CRP), alkaline phosphatase (ALP), alanine transaminase (ALT) and lowest bicarbonate were captured at the time of sampling for all hospitalized COVID, and sepsis patients using EHR (Data S2). Equivalent results at the time of sampling were not available for London patients. D-dimer, lactate dehydrogenase (LDH) creatine kinase (CK) levels at the time of admission, sampling and maximal severity of illness were available for hospitalized Oxford patients (Data S2) but not London patients. Decisions on maximal levels of care based on senior lead clinician decision (frequently with consensus from senior intensive care clinicians) were captured and patients were defined as frail if the plan was not to offer multi-organ support.

Patients with computed tomography (CT) images of the lungs and thorax had images assessed independently by three radiology or respiratory experts to define ground-glass or consolidation patterns of lung involvement, presence or absence of pulmonary embolus and extent of involvement.

The clinical suspicion and/or radiological confirmation of occurrence of any major arterial (e.g., stroke) or venous thromboembolic (deep venous thrombosis, pulmonary embolus) event was captured for each hospitalized Oxford patient and defined as a single binary outcome. Radiological diagnosis of pulmonary embolus was defined separately but compared to this definition to ensure consistency.

Co-prescription of agents hypothesized or proven to be effective in the management or treatment of COVID-19 were logged for all patients with COVID-19. These agents included dexamethasone, remdesivir, interferon 1beta, tocilizumab, anakinra, azithromycin, convalescent plasma or other experimental immunomodulatory agents.

##### Inclusion / exclusion criteria and early stage matching

Patients < 18 years old or those with active malignancy or receiving significant immunosuppression (greater than an equivalent of 40mg once a day of prednisolone) prior to admission, or those with a clear alternative cause for symptoms and hospital presentation were excluded from analyses. For most modalities, samples were prioritized following a stepwise algorithm to match for age, sex and severity of illness. The steps for matching were as follows:

Select severe/critical hospitalized COVID-19 patients first

1. As many critical samples were identified at the earliest point of their maximal severity and samples from patients sampled during severe disease (not defined as frail) were matched in terms of age, sex and time between onset of symptoms and sampling as closely as possible.

2. Rejection sampling was used to select individuals from the comparator groups (mild COVID-19 including both hospitalized and community healthcare workers), healthy volunteers, influenza and all-cause sepsis) to match confounder distributions.

Some downstream analyses required multiple samples from the same individuals transiting through different disease states. In cases where data was available from multiple samples for the same individual a cross-sectional analysis involved prioritizing a single sample based on closeness to maximal severity of illness.

### Method details

#### Blood sample processing

Whole blood from hospitalized Oxford patients, healthcare workers and healthy volunteers were sampled into Tempus tubes (Life Technologies Corporation) for extraction of whole blood total RNA for sequencing or DNA for genotyping or sequencing, or EDTA buffered vacutainers (Fisher Scientific) for processing within 4 hours of sampling. Processing consisted of mixing of whole blood in a 1:1 ratio with Cytodelics (Cytodelics), and then fractionated using Leucosep (Griener Bio-One) into peripheral blood mononuclear cells (PBMC) and EDTA-buffered plasma. PBMCs and plasma were frozen and thawed in batches for specific experiments to minimize the risk of batch effects. Tempus tubes were frozen at −80°C until extraction of RNA performed in batches.

#### Immune cell profiling using mass cytometry

##### Sample processing and antigen staining

On the day of staining, Cytodelics stabilized samples were thawed, processed to remove red blood cells and fixed using Whole Blood Processing Kit (Cytodelics) as per manufacturer instruction. Two control samples from 2 healthy volunteers (Control A and Control B) were included to each batch to assess batch to batch variations. Fixed cells were distributed in 96 deep well V-bottomed plate, washed once with CSB containing Benzonase, and then barcoded using the Cell-ID 20-Plex Pd Barcoding Kit (Fluidigm).

Briefly, cells were washed once with Barcode Perm Buffer, resuspended in Barcode Perm Buffer supplemented with Heparin to reduce nonspecific eosinophil staining artifacts (Rahman et al., 2016) and loaded with half the amount of metal barcodes recommended by the manufacturer. After 30 m incubation at room temperature, cells were washed twice in CSB, pooled and counted. 40%–70% of cells in whole blood are CD66^+^ granulocytes, and their frequency can increase to up to > 95% in septic patients. To maintain both the possibility to measure changes in the frequency of granulocytes and to analyze with a reasonable efficiency mononuclear cells in samples from COVID-19 and sepsis patients, each batch was split in two aliquots. The first aliquot was stained with no enrichment/depletion done (i.e., unaltered). The second aliquot was enriched for mononuclear cells using a granulocytes depletion kit based on the magnetic separation of CD66+ cells using beads coated with anti-CD66 antibodies (EasySep; 17882). Both granulocyte depleted and non-depleted samples were first stained with the surface antibody cocktail for 30 m at room temperature), washed with CSB, then fixed with Nuclear Antigen Staining Buffer and permeabilized with Nuclear Antigen Staining Perm before staining with an intracellular antibody cocktail. After staining at room temperature for 45 m cells were washed in CSB buffer, and fixed in 1.6% FA solution before overnight incubation with Cell-ID Intercalator–Ir. On the day of acquisition, cells were washed once in CSB buffer supplemented with Benzonase, resuspended in water and run at an acquisition rate of < 300 events/second and acquired on a Helios CyTOF machine. An additional aliquot of whole blood was thawed and stained for those samples where yield was too low after staining and acquisition. In total 31 samples were acquired twice (in different batches).

#### Flow cytometry

For flow cytometry we analyzed one aliquot of the same PBMC samples at the same time they were being processed for 10x Genomics CITE-seq. Each aliquot was split into three (to have 5-10x10^5^ cells/staining) and distributed in 96 well V-bottom plates. Cells were spun at 500*g* for 10 min, then pellets stained form 10 min at RT in PBS with 15 μL live dead Aqua, diluted as per manufacturer’s instructions) supplemented with 165 μg/well human Ig (Gammanorm, Octopharma), to block non-specific Fc receptors binding. Cells were washed with 200 μL FACS buffer (PBS 10% FCS), 10 min 300*g* and pellets were stained with each antibody mix in Brilliant Stain Buffer (BD Biosciences), 20 min at RT. Cells were with 200 μL FACS buffer, 10 min 300*g* and fixed in IC Fixation Buffer (Thermo Scientific) diluted 1:2 in PBS, total 200 μl/well. Cells were fixed a minimum of 30 min and a maximum of overnight before being acquired at the BD Symphony X50.

#### Whole blood total RNA-seq

Total RNA-seq was performed with libraries prepared by Oxford Genomics Centre with the NEBNext Ultra II Directional RNA Library Prep Kit for Illumina after rRNA and globin depletion. Libraries sequenced as a single pool of 144 samples (124 patients) on one NovaSeq S4 flow cell (4 lanes) with a target of 50M 100bp read pairs per sample. Due to limited material availability influenza samples were sequenced used a small-bulk RNA-sequencing method in order to facilitate genetic demultiplexing of the CITE-seq data.

#### Bulk BCR and TCR sequencing

Bulk isotype-resolved B cell receptor sequencing was performed on RNA from whole blood using a protocol adapted from (Bashford-Rogers et al., 2019), with moderations including the use of a more sensitive reverse transcriptase (Superscript IV) and primer concentrations (primer sequences provided in Key Resources Table). Bulk TCR sequencing was based on the same protocol adapted from (De Mattos-Arruda et al., 2019), in which the TRB primers were redesigned to capture the full productive repertoire (primer sequences provided in Key Resources Table). Sequencing libraries were prepared using Illumina protocols and sequenced using 300bp paired-ended sequencing on a MiSeq (Illumina).

#### 10x Genomics Chromium CITE-seq

To minimize batch effects, cryopreserved PBMCs were processed in ten batches of 14 samples per batch, with each batch containing at least one sample from each comparator group and similar distribution of patients age and sex across the batches. After thawing, all 14 samples were mixed together to form a single pool (at a ratio that yielded the same number of live cells pooled per sample based on live/dead counting) followed by viability staining (7-AAD dye, BioLegend 420404) and live/dead sorting on a BD FACSAria Fusion sorter.

Post sort, each pool was incubated with FcX block (BioLegend) for ten minutes on ice, washed, and stained with a 192 TotalSeq-C antibody panel (BioLegend 99814) for 30 min on ice. Cells were washed three times in PBS + 1% BSA, counted on the BioRad TC20 Automated Cell Counter and loaded onto the 10X Genomics Chip G at 50,000 cells per channel. Each pool was loaded across seven channels.

#### scVDJ-seq data generation

scVDJ data was generated on the same cells as the scCITE-seq and GEX datasets. Cell Ranger software (v3.1.0) was used to process the Chromium scRNA-seq output data. The FASTQ files were filtered per sequence library plex (i.e., per-pool) using the 10x Genomics index-hopping-filter (https://support.10xgenomics.com/docs/index-hopping-filter) that implements a strategy to mitigate the known index hopping issue with the Illumina machines that use patterned flow cells. The IMGT’s reference genome was used as a reference for the BCR and TCR VDJ libraries. Cell Ranger was also used to filter read alignments, cell barcodes and UMIs, excluding those droplets with low numbers of reads (e.g., erythrocytes or droplets with encapsulating ambient RNA from dying cells).

#### 10x Genomics scATAC-seq

Similar to the CITE-seq approach, cryopreserved PBMCs were processed in two batches of five samples per batch with each batch containing at least one sample from each comparator group. Each sample underwent viability staining (7-AAD dye, BioLegend 420404) followed by live/dead sorting on a BD FACSAria Fusion sorter. Post sort, five samples of an equal number of cells were mixed together to form a single pool, and each pool underwent cell lysis and nuclear extraction according to the 10X demonstrated protocol Nuclei Isolation for Single Cell ATAC Sequencing CG000169 Rev D. Briefly, 200,000 cells from each sample were added to form the pool and cell lysis was performed with 100 μL chilled Lysis Buffer (10 mM Tris-HCl (pH 7.4), 10 mM NaCl, 3 mM MgCl_2_, 0.1% Tween-20, 0.1% Nonidet P40 Substitute, 0.01% digitonin and 1% BSA) for three minutes on ice. The lysis reaction was quenched with 1 mL chilled Wash Buffer (10 mM Tris-HCl (pH 7.4), 10 mM NaCl, 3 mM MgCl_2_, 0.1% Tween-20 and 1% BSA) and the nuclei were centrifuged (500*g* for 5 min at 4°C). After removal of supernatant, the nuclei were resuspended in chilled diluted nuclei buffer (10X Genomics; 2000153) and concentration determined with the Bio-Rad TC20 Automated Cell Counter. Each pool of nuclei was loaded across four channels of the 10X Genomics Chip E (15,000 nuclei per channel).

#### Tims-TOF mass spectrometry

##### Sample preparation of non-depleted plasma/serum for LC-MS/MS analysis

###### Samples

Plasma and serum samples were stored at −80°C then thawed overnight at 4°C before use.

###### Protein precipitation and protein digestion using S-trap

For the bottom-up proteomics approach, proteins were precipitated using Isopropanol (IPA) and loaded into the S-trap 96-well plates (Profiti, Huntington, NY, USA) where proteins were retained for subsequent trypsin digestion following S-trap manufacturer instructions (Zougman et al., 2014). Briefly, five microliters of plasma or serum were pipetted under a category 2 fume hoods into standard 96-well plates pre-filled with 200 μl of isopropanol following the 96-well plate layouts. Precipitation plates were kept at room temperature for 1h before transferring the isopropanol – plasma (serum) mixture into the 96-well S-trap plates. 200 μl of S-trap binding buffer (90% methanol, 100mM Triethylammonium bicarbonate (TEAB)) were added into each well of the precipitation plate to recover any protein precipitates and transferred into the corresponding 96-well S-trap plates. Plates were then spun at 1500*g* for 2 min. Protein colloidal precipitates were retained into the S-trap mesh. S-trap plates were then subjected to four consecutive washes with S-trap binding buffer (200 μl for the first wash and 350 μl the three remaining), followed by a 2 min spin at 1500*g*. 125 μl 50 mM TEAB containing trypsin (Worthington) to a 1:25 wt:wt ratio was added into each well and incubated overnight at 37°C. Tryptic peptides were sequentially eluted from the S-trap into a clean 96-well plate with 80 μl of 50 mM TEAB, 80 μl of 0.2% formic acid (FA) in LC-MS water and 80 μl of 0.2% FA in 50% acetonitrile with 2min spins at 1500*g* after each solvent addition. Finally, plates containing tryptic digests were dried in the speed vac and reconstituted with 70 μl of 0.1% FA for subsequent LC-MS/MS analysis after two serial dilutions (1/100 and 1/10) in 0.1% FA for a final 1/1000 dilution plate to achieve suitable peptide concentration for LC-MS/MS loading.

###### Pools

For quality control (QC) purposes and for repeat injections, a pool for each clinical sample group was created and processed as described above. In addition to the sample group pools, an overarching master pool for each of the plasma and serum cohorts was also built from the individual non-diluted tryptic peptide sample group pool with master pools built to reflect the contribution of each individual group to the overall study.

##### High pH fractionation of Master pools to generate plasma/serum libraries

To increase the depth of the non-depleted plasma/serum proteome, master pools were subjected to a high pH fractionation using the SOLA HRP cartridges (Thermo Fisher) with the aim to create a protein library. In brief, an equivalent of 150μg of peptides from each master pool was fractionated into eight different fractions going from 0% to 70% acetonitrile in 10 mM ammonium formate at pH 10. Prior sample loading, SOLA HRP columns were conditioned with 0.1% trifluoroacetic acid (TFA) in 70% acetonitrile and washed with 0.1% TFA. Following the sample loading, columns were washed with 0.1% TFA before starting the stepwise high pH fractionation elution with 200μl of 0%, 10%, 15%, 20%, 25%, 30%, 40% and 70% ACN in 10 mM ammonium formate pH 10. Eluted fractions were then dried in a speed vac and reconstituted with 40μl of 0.1% FA prior to LC-MS analysis.

##### LC_MS/MS using the high-throughput Evosep One - Bruker TimsTOF Pro platform

C18 Evotips (Evosep one, Odense Denmark) were prepared following manufacturer’s instructions. Briefly, Evotips were conditioned with 1-propanol, washed with solvent B (0.1% FA in 100% acetonitrile) and equilibrated in solvent A (0.1% FA in LC-MS water) before loading them with a total volume of twenty microliters containing 10 μl of sample (peptides) from dilution 1/1,000 plate (equivalent to an estimated 30 ng of peptides). Evotips were washed once with solvent A and stored in 100 μl solvent A till the sample injection.

Samples were analyzed using an Evosep One LC system connected to the TimsTOF Pro mass spectrometer (Bruker Daltonics). Peptides were analyzed using the pre-built 100 samples / per day method (Evosep) with an 11.5 min gradient (total cycle time of 14.4 min) at a 1.2 μl/min flow rate (Bache et al., 2018). In brief, tryptic peptides were transferred from the pre-loaded C18 Evotips with a pre-built gradient to a sample loop and separated on an 8 cm C18 analytical column (Evosep Pepsep, 3 μm beads, 100 μm ID) with an overall gradient from 3 to 40% acetonitrile.

Mass spectrometry data were acquired in PASEF mode (oTOF control v6.0.0.12). The ion mobility window was set to 1/k0 start = 0.85 Vs/cm^2^ to 1/k0 end = 1.3 Vs/cm^2^, ramp time 100 ms with locked duty cycle, mass range 100 - 1,700 m/z. MS/MS were acquired in 4 PASEF frames (3 cycles overlap). Target intensity was set to 6,000 and threshold intensity 200. The data acquisition method has been deposited within the raw data in the ProteomeXchange data repository (PXD023175) (Perez-Riverol et al., 2019).

To assess the technical reproducibility, master pools were run before each sample group and sample group pools were run after the master pools, every twenty sample runs and after the last sample of the group. Blanks were injected in between groups to control for carry over.

#### Luminex assay

The concentrations of selected proteins in the plasma and serum were measured with Human Magnetic Luminex Kits (Bio-techne) with 3 panels containing total 51 analytes: C-C motif ligand (CCL)2/3/4/11/17/18/19/20, CD40 Ligand (CD40L), CD163, complement component 5a (C5a), C-X-C motif chemokine ligand (CXCL)1/5/10, epidermal growth factor (EGF), basic fibroblast growth factor (FGF2), granulocyte colony-stimulating factor (G-CSF), granulocyte-macrophage colony-stimulating factor (GM-CSF), granzyme B (GrB), interferon (IFN)a/b/g, interleukin (IL)-1a/1b/2/3/5/6/8/10/12/13/15/17A/23/33, lactoferrin (LF), Lipocalin-2 (LCN2), Lymphotoxin-alpha (LT-a), macrophage colony-stimulating factor (M-CSF), Myeloperoxidase (MPO), beta-nerve growth factor (b-NGF), Oncostatin M (OSM), S100 calcium-binding protein A9 (S100A9), stem cell growth factor (SCGF), tissue factor (TF), tissue factor pathway inhibitor (TFPI), transforming growth factor alpha (TGF-a), Thrombopoietin (THPO), tumor necrosis factor (TNF) and triggering receptor expressed on myeloid cells 1 (TREM-1). The assays were conducted according to the manufacturer’s instruction. Results were obtained with a Bio-Rad Bio-Plex® 200 Systems. Fluorescence intensity (FI) data from the assays were used for further analysis.

### Quantification and statistical analysis

#### Statistical and unsupervised analysis of clinical phenotyping using clustering

To analyze demographic and clinical features cohorts the following statistical tests were applied. For approximately Gaussian distributed continuous data we used the anova test (using the function f_oneway from Python’s scipy.stats package). For other distributions of continuous variables, we used the Kruskal-Wallis-test (using the function kruskal from Python’s scipy.stats package). For categorical data we used the chi-square test (using the function chi2_contingency from python’s scipy.stats package).

Unsupervised and consensus k-means clustering were used to analyze the clinical data (Moniti et al., 2003; Seymour et al., 2019). We prepared the data by applying quantile-normalization to counts and shift+rescaling to zero mean and unit variance to all features (applying quantile normalization to all features, not just counts, further improves the correlation of the ordered consensus matrix with the WHO severity_at_sample classes). We then ran consensus k-means clustering to identify the best number of clusters. This means we sample from our clinical dataset 50% of the entries, run k-means clustering (where we vary k, i.e., the number of clusters), and record how often two elements were clustered together out of the time where they were in the 50% of samples data points in a so-called consensus matrix. We repeated this process 600 times for k = 2,3,4,5,6 clusters. For each k, we computed the empirical cumulative distribution (CDF) and the change in the area under the CDF curves for different k. The optimal cluster number is obtained for the k after which the CDF curves (and hence the areas) do not change significantly anymore. We then performed an unsupervised consensus k-means clustering analysis of (subsets) of the clinical variables (using python’s sklearn library). In order to mitigate the question of which features are independent, we performed this analysis on the principal components (using sklearn). After consensus clustering, we sorted the consensus matrix using hierarchical clustering. The methods were implemented in python and used the packages numpy, scipy and pandas. We also performed hierarchical clustering directly on the PCs, rather than on the consensus matrix (using AgglomerativeClustering and dendrogram from sklearn).

#### Mass cytometry data analysis

##### CyTOF data pre-processing

After acquisition, data was normalized and concatenated on CyTOF Software v7.0, compensated on CATALYST (version 1.5.3.23). Then, data was further processed for removal of beads and of DNA negative events and for Gaussian parameters using a manual gating strategy using FlowJo v10.6 as described in Data S3. Then, samples were manually debarcoded (FlowJo v10.6) and CD45^+^ events (non-depleted samples), or CD66-Siglec8-CD45^+^ (granulocytes-depleted samples) events were selected for further processing. For each batch, two control samples were run together with the patients’ samples to allow for correction for batch effects. Batch correction was performed with CytoNorm software (version 0.0.5) separately for granulocyte depleted and non-depleted samples. For both analyses, the training of the algorithm was done with 20,000 cells per sample and 5 clusters (parameter nClus) using one of the control samples and the results were assessed using the other control sample. Doublet cells were removed by manual gating on FlowJo v10.6 (Data S3) and were deemed as Iridium^high^Ki67^−^ cells to avoid removing proliferating cells. Finally, to correct for potential biases due to highly varying cell numbers per sample, down-sampling was performed to a maximum 75,000 cells per sample for the granulocyte depleted samples and 40,000 for the non-depleted samples.

##### Clustering

Clustering analysis and the identification of the different immune cell population was done using the analytical pipeline described in Data S3. Clustering analysis was performed separately for granulocyte depleted and non-depleted samples using a self-organizing map algorithm through an implementation of FlowSOM via the CATALYST R package (version 1.10.3). The initial clustering was performed using a resolution of 144 clusters (dimensions 12x12) and metacluster merging by consensus clustering was performed at a resolution of 25 clusters for both datasets. The clusters were then manually annotated to define the populations. Three major populations (T/NK, B/Plasmablasts and Myeloid) from the granulocyte depleted samples were further clustered to identify finer subpopulations using a resolution of 225 (dimensions 15x15) for T/NK and 144 (12x12) for the other two. The metacluster merging was performed at a resolution of 50 clusters for the T/NK population, 25 for the B/Plasmablast and 30 clusters for the Myeloid population. Clusters were manually annotated and further managed to provide cell type resolution at two different depths - one to define major cell types, and a finer one to define subpopulations. The cell type assigned to each cell ID in each of the three major population was then used to reconstitute the frequency of each population inside the granulocyte depleted sample. The finer annotation was used to evaluate the frequency of cell subtypes.

##### Differential abundance analysis

Differential abundance analysis was performed using code from the diffcyt package (version 1.8.8) with the option testDA_edgeR. The analysis was adjusted for the confounding variables batch, age and sex. For the analysis of the non-depleted samples the normalization for composition bias was deemed necessary to account for the pronounced differences in neutrophil proportions. For all other analyses no normalization was performed. The subpopulations of the granulocyte depleted samples were analyzed independently of each other to avoid composition effects.

The full results of the clustering and differential abundance analysis results are shown for non-depleted whole blood together with principal components analysis (Data S3); and clustering and differential abundance analysis for granulocyte depleted whole blood broad populations, myeloid, B cell and T/NK populations (Data S3).

##### Manual gating and CD38 median intensity (MI)

Two subpopulations of cells were more precisely defined by manual gating rather than by clustering. To do that, the cluster of the parent population (cMono or MAIT) was exported from the R environment and gated in FlowJo. Also, the median intensity of CD38 expression were identified in different cell types within the granulocyte depleted samples.

##### Density plots

The density plots were made by down-sampling to have the same number of events across all different conditions and using the geom_pointdensity function of the ggpointdensity package, with an adjust of 4, to generate the ggplot.

#### Integration of CyTOF and CITE-Seq data

The two datasets were aligned using Seurat (v3.9.9.9010) using only samples that had been analyzed on both technologies (115 samples, after exclusion of one sample due to low number of cells in CITE-Seq). The integration was based on an overlapping set of 38 markers that were common between the CyTOF and ADT antibody sets. Both datasets were filtered to exclude cells with unclassified or uncertain annotations. The CITE-Seq dataset was further filtered to exclude potentially low-quality cells with i) number of genes, ii) number of features of ADT, iii) number of total UMI (RNA) or iv) number of total UMI of ADT lower than 0.001 of their relevant distributions. The two datasets were then down-sampled to 1,000 cells per sample and the integration was performed on 230,000 cells in total.

The anchors between the query and reference datasets were found based on a CCA with 30 dimensions and the labels were transferred using a PCA for the weight reduction of the query dataset. The analysis was performed with either the CITE-seq or the CyTOF as reference datasets in turn and all cells were given a predicted annotation from their counterpart dataset. Then the predicted annotation of the cells was compared to their original annotation and all the common major cell types were validated with very high accuracy (Figure S2B). Finally, the visualization of the two datasets was performed with the CITE-seq data as a reference. The anchors were used to impute the ADT markers for the CyTOF cells which were then merged with the CITE-seq data and centered in order to run a UMAP analysis to visualize all the cells together (Figure S2A).

#### Flow cytometry data analysis

Data were analyzed with FlowJo version 10. Cells were gated on live leukocytes and recompensated in FlowJo. Frequencies of individually gated populations were exported and plotted in Prism. Alternatively, 15,000 CD3^+^ cells per sample were concatenated and exported for subsequent analysis in R. Supporting data for analysis of memory CD4^+^ T cell subsets is shown (Data S3).

#### Whole blood total RNA-seq analysis

##### RNA-sequencing data processing

We trimmed adaptor sequences using TrimGalore (v0.6.2, https://github.com/FelixKrueger/TrimGalore), and aligned reads to the reference genome (GRCh38.100) using multi-sample 2-pass mapping with STAR v2.7.3 (Dobin et al., 2013) (ENCODE Best Practices recommended parameters). We quantified gene expression using featureCounts (v1.6.4) (Liao et al., 2014) and annotations from Ensembl (v100). We calculated and checked QC metrics from FastQC, mapping metrics from STAR, duplication rates and other RNASeq metrics from Picard (v2.23) (http://broadinstitute.github.io/picard/) and RNASeQC (v2.3.5) (DeLuca et al., 2012) and checked for outliers in principal component analysis. We removed 1 sample with high proportions of duplicates and short reads, low exonic rate, number of mapped reads and number of genes identified, and which was also clearly outlying on PCA. We also confirmed that there were no sex mismatches that could indicate sample mix-ups based on sex chromosome gene expression using QTLTools (Delaneau et al., 2017). This resulted in a dataset of 143 samples from 123 patients. We filtered out features that did not have at least 10 reads in at least 10 samples, retaining 23,063 features for downstream analysis. We then normalized the data using the trimmed mean of M-values method R package from edgeR; https://doi.org/10.1093/bioinformatics/btp616 and log2-transformation of counts-per-million. Code is available on GitHub (https://github.com/COMBATOxford/bulkrnaseq/mapping/).

##### Exploratory analysis

We carried out principal component analysis (PCA) on the normalized filtered data for 123 patients using prcomp (R v 3.6.2) with default parameters. We additionally performed PCA on just the samples from hospitalized COVID-19 patients (n = 64). We selected informative PCs by taking the first n PCs that cumulatively described at least 80% of variance in the data. We explored the relationship between these PCs and the clinical variables available (transformed and filtered as described above) using Spearman correlation (Figure S3A). Additionally, we investigated the biological relevance of the PCs through pathway enrichment analysis with the R package XGR (Fang et al., 2016b) and Reactome pathways. For each PC, we took the 500 genes with the highest absolute loading scores as input and compared to a background of all detected genes.

##### Data ellipses

For PCA plots, to aid visualization 95% data ellipses were generated assuming a multivariate t-distribution. These were drawn using the stat_ellipse method from the R package ggplot2, using default parameters. Data ellipses were prepared in the same fashion for CITE-seq composition and expression analysis, and proteomics.

##### Differential expression

We performed differential expression analysis on the normalized data with one sample per patient using the limma R package (Ritchie et al., 2015) . We included age, age^2^ and sex as fixed effects and compared each patient subgroup with all others, as well as investigating COVID-19 severity as a quantitative trait (mild = 1, severe = 2, critical = 3). We defined significance for downstream analysis as FDR < 0.05 and fold change > 1.5. We investigated the impact of variation in cell proportions across samples calculated from hospital measurements for neutrophils, monocytes, and lymphocytes, by adding neutrophil and monocyte proportions to the model and comparing the estimated fold changes to the results to the more basic model (correlation plot showing the influence of cell proportion on detection of differentially expressed genes in Figure S3C). Pathway enrichment analysis was performed using Reactome pathways via the XGR R package (Fang et al., 2016b), with Fisher’s exact test and filtering of redundant terms by the xEnrichConciser function.

##### Weighted gene correlation network analysis

We applied weighted gene correlation network analysis (WGCNA) to describe modules of highly correlated genes within the whole blood total RNA-sequencing data (i.e., 143 samples from 123 patients) (Langfelder and Horvath, 2008). We utilized the “cornet” pipeline to wrap the WGCNA R package and perform gene set enrichment analyses (https://github.com/sansomlab/cornet). In brief, a stepwise approach of correlation network construction and module detection was adopted using log_2_-transformed counts-per-million RNA-sequencing data. Using the soft thresholding power of 4, a signed-hybrid network was built implementing the biweight midcorrelation as the adjacency function. The adjacency matrix was transformed into a topological overlap matrix in order to calculate the dissimilarity and a dissimilarity threshold of 0.3 was used to merge modules with very similar expression profiles. Module eigengene values (module first principal component) were used to summarize modules and perform module-trait correlation analyses (Pearson correlation). Gene set over representation analyses were performed using the default settings of the “cornet” pipeline (https://github.com/sansomlab/cornet).

#### CITE-seq: pre-processing and multimodal annotation

##### Pre-processing of 10X Libraries (Gene expression, ADT, TCR, BCR)

As described above a total of n = 140 PBMC samples from COVID-19, sepsis, influenza and healthy volunteers were mixed into n = 10 pools. Each pool comprised of n = 14 samples (each from a different individual) and cells from each pool were captured using n = 7 10X channels (Figure S1D). From each channel, four libraries were generated, Gene expression (GEX), Surface proteome (ADT), TCR and BCR repertoires. The libraries were sequenced on an Illumina NovaSeq 6000 sequencer across nine S4 flowcells (4 lanes per flow cell). For each of the sample pools, the n = 7 GEX and ADT libraries were sequenced on 3 dedicated lanes (to prevent index hopping between sample pools). The BCR/TCR libraries were sequenced on the remaining 6 lanes. Mapping indexes for the bulk and single cell data were built using GRCh38 genome sequences and Ensembl version 100 annotations. For construction of the Cellranger reference transcriptome we included genes with the following Ensembl biotypes: protein_coding, IG_V_gene, IG_V_pseudogene, IG_D_gene, IG_J_gene, IG_J_pseudogene, IG_C_gene, IG_C_pseudogene, TR_V_gene, TR_V_pseudogene, TR_D_gene, TR_J_gene, TR_J_pseudogene, TR_C_gene (n = 20,615 genes; lncRNA not included). FASTQ files were generated using Cellranger (v3.1.0) mkfastq and individually QC’ed using FastQC (Andrews, 2010). To mitigate index hopping between channel libraries within sample pool FASTQ files were filtered per pool using the 10x Genomics index-hopping-filter (https://support.10xgenomics.com/docs/index-hopping-filter) (v1.0.1). Per-channel (n = 70) GEX and CITE-seq matrices were prepared using Cellranger (v3.1.0) count. Cell identification was performed on the GEX modality with both Cellranger (v3.1.0) and EmptyDroplets (Lun et al., 2019) (1.8.0) and the union of the calls from both algorithms taken forward as the set of identified cells.

##### Genetic demultiplexing of 10X GEX data

The GATK variant calling pipeline (https://github.com/gatk-workflows/gatk4-rnaseq-germline-snps-indels) (v4.1.7.0) was applied to the bulk RNA-seq data to identify sequence variants for the genetic demultiplexing of the single cell data. Minor modifications were made to the variant calling pipeline to allow execution from FASTQ files, incorporate metadata into the BAM alignment files, and produce a block gVCF file. We applied both Demuxlet (Kang et al., 2018) (v2; https://github.com/statgen/popscle/commit/038044660337b50ec89b0b355493ceb707f18ecd) and Vireo (Huang et al., 2019) (v0.4.0; https://github.com/single-cell-genetics/vireo/commit/4aecb54a4f2fa3a3413e2cd3e9f3515744385cba), proceeding with the demultiplexing calls from Vireo as they were more robust to variation in read depth. The dataset was demultiplexed using full genotypes (per-pool gVCF files) from the bulk sequencing. For each of the n = 70 10x channel sequence libraries, Vireo consistently demultiplexed 60 – 75% of cells. A total of n = 884,587 singlet cells were demultiplexed.

##### QC of 10X GEX data and preparation of GEX matrices

The pre-processing workflow was encapsulated in a set of CGAT python pipelines (Cribbs et al., 2019) and version controlled. Cell quality was assessed with a suite of metrics that included total UMI (GEX), number of genes detected, percent mitochondrial gene expression, percent ribosomal gene expression, percent IgG expression, Scrublet doublet score (Wolock et al., 2019) and total UMI (ADT). Ambient RNA was assessed. Cell QC statistics, demultiplexing assignments and cell and patient metadata were centrally warehoused in an sqlite database. Following inspection of the QC metrics, the dataset was filtered to retain cells with ngenes > 300 and pct_mitochondrial < 10%. In total, n = 836,148 cells were selected for downstream analysis. Expression data for selected cells was extracted from the per-channel count matrices using R and combined into a single market matrix with AWK. RNA velocity matrices were computed for each of the channels with Velocyto (http://velocyto.org/) (La Manno et al., 2018) (v0.17.5). Velocity data for selected cells was extracted and combined into a single matrix using the Loompy Python library (http://loompy.org).

##### Multimodal cell annotation strategy overview

We used expert immunological knowledge to guide a curated integration of the data from the different modalities (GEX, ADT and VDJ) to identify and label the cell sub-populations present in the CITE-seq dataset (Figure 1B). As detailed below, we first performed separate clustering of gene expression, clustering of surface protein expression, and analyses of T and B cell receptor V(D)J sequences. Next, led by expert understanding of the three feature spaces we prioritized use of ADT surface phenotype for definition of major cell lineages and subsets where definitive marker expression was available. Cell types and subsets were further refined using information from the repertoire and GEX layers, or in the absence of definitive ADT information were identified by GEX cluster phenotype. Finally, the identified cell types and subsets were further divided by inferred functional state based on targeted assessment of information from all three modalities. For example, cell cycle phase was determined by GEX phenotype, T cell memory versus effector status was distinguished using information from both the GEX and ADT layers, while assignment of B cell maturation status involved use of information from all three modalities (including BCR mutational status). Information from all three modalities was used to identify and exclude doublets from downstream analysis.

##### Alignment and clustering of 10X GEX data

Prior to alignment data was normalized and log1p transformed using Scanpy (Wolf et al., 2018). The top n = 3000 highly variable genes (HVG) were selected (Seurat flavor with n_top_genes set). The following MHC and variant immune receptor genes were excluded from HVG identification A/B/C/D/E/F/G]^∗^, IGH[D/J/V]^∗^, IGK[J/V]^∗^, IGL[J/JCOR/L/ON/V]^∗^, TRA[J/V]^∗^, TRB[D/J/V/VA/VB]^∗^,TRD[D/J]^∗^ and TRG[J/JP/V/VA/VB]^∗^). Effects associated with total number of UMIs were regressed out and PCA components computed. We evaluated alignment with Harmony (Korsunsky et al., 2019), Scanorama (Hie et al., 2019) and BBKNN (Polański et al., 2020) and chose to proceed with Harmony following inspection of UMAP projections of the alignment results (data not shown). Following inspection of the PCA scree (knee/elbow) plot, Harmony alignment of samples (n = 140 levels) was performed in Python using the top n = 65 PCs. Leiden clustering, marker discovery (wilcox tests) and visualization (with violin plots, UMAPs, volcano plots, MA plots and heatmaps), automatic cell type identification (with singleR (Aran et al., 2019) and via over-representation analysis of xCell (Aran et al., 2017) genesets), basic composition analysis, geneset over-representation analysis (including of GO, KEGG and Biocarta genesets) and visualization of the aligned datasets was performed using pipeline_scxl.py (https://github.com/sansomlab/tenx). Neighbor graphs were built using Euclidean distance and the “HNSW” algorithm (as implemented in ScVelo (Bergen et al., 2020), following the example of Pegasus (Li et al., 2020)). Clustering was performed using Scanpy, marker discovery with Seurat (Stuart et al., 2019) and pathway over-representation analysis with gsfisher (Croft et al., 2019) (https://github.com/sansomlab/gsfisher).

After alignment of the full manifold, we iteratively divided, re-aligned and re-clustered the dataset to achieve high-resolution clustering of different cell subsets. Variable gene identification was performed separately within each subset as described above. In the first step the major cell types – T/NK, myeloid and B/plasmablast cells were extracted into n = 3 separate subsets. Each of these three subsets was then separately aligned and clustered (n = 45 PCs for n = 482k T/NK cells; n = 45 PCs for n = 279k myeloid cells; n = 40 PCs for n = 66k B/plasmablast cells). This process was then repeated with a final (third) round of alignment and clustering being performed separately on six subsets: (A) cells in the CD4 region of the manifold (with n = 50PCs, n = 302k cells), (B) cells in the CD8 region of the manifold (with n = 45PCs, n = 180k cells), (C) cells in the myeloid region of the manifold (with n = 50PCs, n = 261k cells), (D) cells in the B cell/Plasmablast cell region of the manifold (with n = 40PCs, n = 57k cells), (E) cells identified as doublets based on scrublet scores and marker gene expression (with n = 40PCs, n = 26k cells), (F) other cells types not falling into any of the first five categories (with n = 50PCs, n = 10k cells). The clustering strategy is shown in Figure S1E. Choice of principal component number was guided by inspection of the scree plots.

Each of the cell six subsets was subject to Leiden clustering with a range of resolutions (1, 1.5, 2, 2.5, 3, 3.5, 4, 4.5, 6, 8) and cluster phenotypes assessed using pipeline_scxl.py (https://github.com/sansomlab/tenx) as described above, and ADT surface phenotype. For each subset a base resolution was selected and sub-populations further refined by manual splitting or merging of clusters based on alternate cluster resolutions guided by expert knowledge of cell identities and phenotypes to define a total of n = 131 GEX clusters. These clusters were intersected with information from the ADT and repertoire modalities for the final multimodal annotation of the dataset as described below.

##### Pre-processing and analysis of the surface protein (ADT) data

For each channel the ADT signal was normalized independently to its background, using the DSB algorithm (Mulè et al., 2020) taking the set of cells identified from the GEX data as the foreground and unassigned GEMs with log_10_(GEX_nUMI +1) ≥ 1.5 as the background. The workflow was written in R and Python, encapsulated in CGAT pipelines and version controlled. The normalized ADT data was subject to hierarchical stochastic neighbor embedding (hSNE) and unsupervised Gaussian mean shift (GMS) clustering as implemented in Cytosplore (van Unen et al., 2017) (https://www.cytosplore.org). This analysis, which was based on the expression of all n = 192 ADT tags, partitioned the dataset into 66 discrete clusters. Based on expression of known lineage markers (CD3, CD4, CD8, CD56, CD19, CD20, CD27, CD38, CD14, CD123, CD1c, CD33, CD235, CD34, Vδ2, Vα72 and CD161) these clusters were apportioned to NK cells, B cells, Plasmablasts, Vδ2^+^ T cells, CD14^+^ Monocytes, CD123^+^ pDC, CD1c^+^ DC, red blood cells (RBC), CD34^+^ cells and distinct populations of CD3^+^ T cells (Data S3) (clustering of surface protein ADT data). For identification of Vα25^+^Jα18^+^ cells (iNKT cells), Vδ2^-^Vγ9^+^ cells, TCRγδ^+^ cells, Vα7.2^+^CD161^+^ (MAIT) cells and non MAIT T cells a sequential manual gating strategy was employed. After exclusion of likely contaminant CD33+ CD3+ and CD56+ cells subpopulations of B cells and plasmablasts were defined by re-clustering these cells based upon expression of CD10, CD24, CD25, CD19, CD20, CD22, HLADR, CD21, CD23, IgD, CD1c, IgM, CD38, CD29, CD71, CD39, CD27, HLA-ABC and IgA. In total, this analysis of the ADT data identified 12 T cell subpopulations, 7 B cell/plasmablast subpopulations and five distinct sets of doublets (with markers of more than one major immune lineage). These clusters were intersected with information from the GEX and repertoire modalities for the final multimodal annotation of the dataset as described below.

##### Pre-processing and analysis of 10x V(D)J B and T cell repertoire data

T and B cell V(D)J gene usage and receptor sequences were quantified using Cellranger VDJ (v3.1.0) with reference sequences from the IMGT. For TCR chain usage we recorded additional information for each cell including (i) “clone proportion,” computed as the proportion of the entire T cell repertoire occupied by the clone in the sample, (ii) whether the cell was a doublet (defined as a singlet clone with TRA-TRA-TRB-TRB chains), (iii) whether the cell possessed an iNKT receptor phenotype (TRAV10 with TRAJ18), and (iv) whether the cell carried a MAIT receptor phenotype (TRAV1-2, with either TRAJ12/TRAJ20/TRAJ33). Immunoglobulin sequences were further analyzed using IMGT High V-Quest. B cell/plasmablast doublets were identified as cells with multiple heavy chain (HC) contigs where the ratio of the number of UMIs for the top two ranked HC contigs was > 0.125. Light chain (LC) information was not used for doublet identification as it is known that 1% of B cells may express dual light chains. The TCR and immunoglobulin chain usage information was intersected with information from the GEX and ADT modalities for the final annotation of the dataset as described below.

##### Multimodal cluster annotation

As described below, we performed curated intersections of the information from the different modalities to discern the cellular identities and functional phenotypes of the different PBMC populations. Information from all three modalities was used to screen and exclude doublets: in addition to inspection of Scrublet scores (computed from the GEX layer), lineage according to surface phenotype, immune receptor sequence and gene expression profile was required to be congruent. In total, following curated intersection of GEX, ADT and repertoire information we obtained n = 853 clusters. Of these we retained n = 346 clusters (n = 787,928 cells) for downstream analysis. The retained cells comprised of n = 343 clusters (n = 782,245 cells) that were assessed to represent genuine singlet cell clusters based on expert knowledge. We assigned each of these clusters to a “minor subset,” “major subset” and “cell type” category in order to accommodate downstream analyses at different levels of granularity as summarized in Figure 1C. In addition, we retained two clusters that appeared to comprise monocyte-platelet doublets (n = 5562 cells) and a minor, lower confidence cluster of AXL+ DCs (n = 121 cells) for inclusion in cluster-level analyses. We excluded n = 92 clusters (n = 20,391 barcodes) that likely represented cell-cell doublets/multiplets and n = 286 clusters (n = 19,858 cells) which appeared to be comprised of mixtures of cell types. A further n = 129 clusters (n = 7,971 cells) of uncertain phenotype were also excluded. Scripts were written in Python and R and version controlled.

Known T and NK cell subsets were identified by surface protein (ADT) and immune receptor (TCR) phenotype as recorded in Table S2. These identity assignments were then intersected with the gene expression clusters (GEX) to identify subsets of the different lineages that displayed distinct transcriptional phenotypes (e.g., naive versus memory T cells). These sub-populations are shown in Data S4 for CD4 T cells, for CD8 T cells, for double positive (DP) T cells, for double negative (DN) T cells, for mucosal associated invariant T (MAIT) cells, for Vδ2+ gamma-delta (γδ) T cells, Vδ2-ve Vγ9-ve γδ T cells, Vδ2-ve Vγ9+ve γδ T cells and invariant natural killer T cells (iNKT). The identified NK cell subsets are also shown in Data S4.

B cell and plasmablast cell subsets were defined using information from the GEX, ADT and immunoglobin repertoire modalities as detailed and shown in Data S4. Sub populations of innate immune cells and other blood cells were delineated according to gene expression clusters and annotated based on their GEX and ADT phenotypes. The identified sub-populations are shown in Data S4 for mononuclear phagocytes (MNPs), for hematopoietic stem (and progenitor) cells (HSCs) and for platelets and erythrocytes.

To assess the relative immune receptor diversity of the different T, B and plasmablast populations we computed repertoire Shannon and Gini indices (detailed in the Repertoire analysis section, and as shown in Data S4). Because of the large variance in cell number between clusters we estimated the indices by bootstrapping with a sample size of n = 30, n = 1,000 times. Individual bootstrap samples were drawn without replacement to avoid introduction of artificial clonality. The indices were not estimated for clusters with fewer than n = 30 cells.

#### CITE-seq: cell composition analysis

##### Overview

Three samples with < 500 cells in total, and three samples with confirmed or suspected malignancy, were excluded from all composition analyses. For the hospitalized COVID-19 and sepsis clinical categories, only samples closest to maximum severity and only one sample per individual were included, such that for each category the following numbers of samples were analyzed: healthy volunteers, n = 10; COVID-19 acute in-patient mild (OUH), n = 12; COVID-19 acute in-patient severe (OUH), n = 20; COVID-19 acute in-patient critical (OUH), n = 18; COVID-19 community COVID-19, n = 12; influenza, acute in-patient, n = 10; and sepsis acute in-patient, n = 15. Scripts for all analysis were written in Python and R and version controlled.

Composition analysis was performed for the different levels of cellular granularity summarized in Data S5, including for cell types; major cell subsets; minor cell subsets; T and natural killer (NK) cell clusters; B and plasmablast (PB) cell clusters; and mononuclear phagocyte (MNPs) clusters. To initially inspect cluster abundance across clinical categories, the percentage frequencies of cell subpopulations were quantified per sample and visualized as boxplots. For cell types, major cell subsets and minor cell subsets (Data S5), percentages were calculated out of total PBMCs. For higher resolution immune cell clusters (Data S5), frequencies were calculated out of total T and NK cells, total B and PB cells, or total MNPs. Selected panels are reproduced in the main and supplementary figures.

##### Principal component analysis

Principal component analysis (PCA) was used for exploratory analysis, with pre-filtering to remove clusters with a count of n < 10 cells in < 10 individuals. Cluster counts were converted to centered log-ratios (CLRs) using the ALDEx2 R package, v1.18.0. Briefly, 1,000 Monte Carlo samples of the Dirichlet distribution were generated from the cluster counts for each sample and converted to CLRs. The median CLR for each cluster-sample combination was used for PCA which was performed using prcomp (R version 3.6.2) with default parameters. Association tests for clinical variables were performed using an omnibus analysis of variance (ANOVA) to test for association between the top 15 PCs (collectively explaining > 80% of the total variance) and clinical category (source), age, sex, and sample pool, with the Benjamini-Hochberg procedure for multiple testing correction and a significance threshold of *P*_*c*_ < 0.05. For the hospitalized COVID-19 cases, association tests with additional variables that were related to acute disease or were secondary to infection were also performed. All variables had one or no missing values. These additional variables included: ethnicity; weight; days from symptom to sample and from symptom to admission; maximum temperature in the 24 hours preceding sampling; persistent fever in the 24 hours preceding sampling; fever, WHO ordinal, ventilation status, SaO_2_/FiO_2_ ratio, and SOFA oxygenation score at the time of sampling; quantile normalized white cell, neutrophil, lymphocyte, monocyte and platelet counts in the 24 hours before or after sampling; quantile normalized highest concentration of C-reactive protein in the 24 hours before or after sampling; clinical and/or radiological evidence of thromboembolism during hospitalization; length of hospital stay; OpenSAFELY COVID-19 mortality propensity score; and death in the hospital.

The full results of the PCA and association test results are shown in Data S5 for the cell types; for the major cell subsets for the minor cell subsets; for the T and NK clusters; for the B and PB clusters; and for the MNP clusters with selected panels reproduced in the main and supplementary figures.

##### Differential abundance analysis

To compare cell subset/cluster abundance across clinical categories, the percentage frequencies of cells were quantified per sample and visualized as boxplots. For major and minor cell subsets, percentages were calculated out of total PBMCs. For higher resolution immune cell clusters, frequencies were calculated out of total myeloid cells, total B and plasmablast cells, or total T and natural killer (NK) cells. To determine the statistical significance of differences in cell subset/cluster abundance between groups, differential abundance analysis was performed using edgeR, v3.28.1 (Amezquita et al., 2020). Cell subset/cluster counts were modeled adjusting for age, sex and sample pool using a quasi-likelihood negative binomial generalized log-linear model (glmQLFit function), with pre-filtering to remove clusters with a count of n < 10 cells in < 10 individuals. Counts were normalized by the total number of cells in each sample for the major and minor cell subset analyses, or by the total myeloid, the total B and plasmablast, or the total T and NK cells for higher resolution immune cell cluster analyses. Differential abundance testing was performed using the quasi-likelihood F-test. The Benjamini-Hochberg procedure was implemented to correct for the number of clusters and the number of pairwise clinical category comparisons, and a significance threshold of *P*_*c*_ < 0.05 was used. Testing for composition effects did not provide evidence for biases in cluster abundance. In addition, for the hospitalized COVID-19 patients, edgeR analysis was performed using ANOVA to identify statistically significant associations between cluster abundance and patient characteristics/clinical variables (as detailed for the PCA association tests).

The full results of the differential abundance and covariate analysis results are shown in Data S5 for the cell types; for the major cell subsets; for the minor cell subsets; for the T and NK clusters; for the B and PB clusters; and for the MNP clusters.

#### CITE-seq: GEX PCA, differential expression and pathway analysis

##### Data pre-processing

Pseudobulk counts were generated for each combination of gene and sample at minor subset, major subset and cell type level by summing together the within-group gene counts. These were then converted to reads-per-million (RPM) by normalizing by the total count across all genes for each combination of sample and cell type. Finally, residuals were calculated by taking the log(1 + count) and subtracting the predicted value from a linear model with pool as the independent variable. The six poorly performing samples mentioned in the Composition section above were removed from all further processing.

##### PCA analysis

PCA was carried out on the residuals using the prcomp function in R. We generated PCs separately at minor subset, major subset and cell type level using residual values from genes with a mean RPM > 1 in among all sources, and with six poorly performing samples removed. Outlying samples (more than 5 SDs away from the mean across the top 10 PCs) were then removed and PCs recalculated. Plots are shown in Data S6 for the first two principal components of pseudobulk gene expression for each of the major subsets. To test differences in major subset PCs across categories, we used an omnibus ANOVA (i.e., a test of a linear model including all diagnoses/severity categories as dummy variables, against a model with no difference between these groups) to test for association between the first 10 PCs and diagnosis/severity (across all samples) and disease severity (measured by WHO criteria, within COVID-19 samples) (Data S6). The linear model also includes terms for age and sex. Omnibus p values were corrected for multiple testing using the Benjamini-Hochberg procedure. We also repeated the principal component analysis only within hospitalized COVID-19 samples and tested their association with clinical covariates. Only two major subsets showed adjusted p values < 0.01, classical monocytes and DCs, with association patterns shown in Data S6 for clinical covariates in hospitalized patients.

We generated UMAPs from the top n = 10 PCs for each level of cell clustering (using the R package “umap”). For this analysis we excluded clusters with n = 0 cells for ≥ 10 samples and excluded samples with n = 0 cells in remaining clusters. The resulting UMAP plots are shown in Data S6 combining PCs across all clusters at the minor subset, major subset or cell type levels.

##### Differential Expression Analysis

We carried out differential expression tests for a range of contrasts (including subgroups of COVID-19 against healthy volunteers, sepsis and influenza versus healthy volunteers, COVID-19 versus sepsis, COVID-19 versus influenza and COVID-19 subgroups against one another. These tests were performed using the R edgeR library (McCarthy et al., 2012). We included age, sex and pool as covariates, and filtered out samples with < 5 cells and gene with mean RPM < 1, with significant genes selected based on log fold change (logFC) > 2 and false discovery rate (FDR) < 0.01 (calculated by Benjamini-Hochberg). To test for a set of genes that differed across COVID-19 severity categories, we also carried out a “COVID-19 omnibus” likelihood ratio test, with dummy variables for WHO severity and a separate variable for mild recording healthcare workers (in this case, genes were selected if they had logFC > 2 between any pair of categories).

The number of differentially expressed genes (FDR < 0.01, absolute log-fold change > 2) in each cell cluster for each contrast is shown in Data S6, volcano plots of differentially expressed genes for critical COVID-19 patients versus healthy volunteers for each major subset are shown in Data S6, and the top 10 most differentially expressed genes for each contrast (across all cell clusters) in Table S3. We also investigated the relationship between the frequency of a cell subset and the number of differentially expressed genes (Data S6), and the relationship between differential composition and number of differentially expressed (Data S6).

##### Pathway Analysis

Gene-set enrichment analysis GSEA of differentially expressed genes were performed using the FGSEA algorithm (Korotkevich et al., 2021). We performed GSEA for biological pathways from the MSigDB database (including for KEGG, GO, canonical pathways, regulatory sets, immune signatures and Hallmark genesets). Enrichment analysis was carried out separately for each pair of cell cluster and contrast, with genes ranked by p value. For the Hallmark genesets, we also carried out a signed enrichment test (ranking by log(pvalue) x sign(logFC)), shown as a heatmap for major subsets and a range of contrasts in Figures S4D, S5E and S6H. The top 10 pairs of cell cluster and GO term, canonical pathway and immunologic signature for critical COVID-19 versus healthy volunteers are shown in Table S3.

In addition, we applied GSEA to a set of experimentally derived interferon-stimulated genes (ISGs) (Schoggins et al., 2011). Genes within this gene set were differentially expressed across a wide range of minor subsets and contrasts (Figure S4E), with the most significant enrichment being seen in HSCs in critical COVID-19 patients (Figure S4F). We focused in on classical dendritic cells, as this minor subset showed enrichment of differential expression in ISGs (p < 1e-5) in 9 out of 13 contrasts tested. A subset of ISGs were identified both as driving the ISG enrichment signature (leading edge genes) and showing individually strong differential expression (FDR < 0.001, absolute fold change > 3), shown in a hierarchically clustered heatmap of gene expression in classical dendritic cells of highly differentially expressed genes from the leading edges of interferon stimulated gene sets (Figure S4G).

#### CITE-seq: WGCNA analysis

We performed separate WGCNA (Langfelder and Horvath, 2008) analyses of n = 7 selected “major cell types” consisting of cell populations that were annotated as “cell types” (B cells, Plasmablasts, NK cells) or “major subsets” (cMono, ncMono, CD4 T cells, CD8 T cells). For this analysis per-patient pseudobulk-summarized RPM-normalized counts from the prioritized sample set were used as input. Patients with low total cell numbers (< 500 cells; n = 3), with confirmed or suspected malignancies (n = 3), or from the London COVID-19 cohort (n = 2) were excluded from the analysis.

Input gene expression matrices were filtered to retain genes with expression above a certain threshold in a given minimum number of samples. Based on inspection of expression distributions RPM thresholds of 1 (cMono, NK cells, CD4 T cells and CD8 T) and 3 (ncMono, PB and B cells) were chosen for the indicated “major cell types.” The number samples that constituted the smallest patient group was used as the minimum number of samples. After filtering we retained 9,000-12,000 genes per “major cell type.” Log_2_ RPM+1 values were batch corrected using the ComBat algorithm (sva R package v 3.36.0) (Johnson et al., 2007; Leek et al., 2012) specifying the multiplexing sample pool as the adjustment variable (together with an intercept term).

The WGCNA analysis was performed using pipeline_wgcna.py from the https://github.com/sansomlab/cornet repository, and geneset over-representation analysis was carried out using the gsfisher R library https://github.com/sansomlab/gsfisher. The parameters used for WGCNA runs are given in Data S6. We excluded 3 “ambient_rna” modules whose eigengene gene loadings correlated strongly with ambient RNA species abundance (as quantitated in “empty” droplets) (Spearman’s rho ≥ 0.83, p < 2.2 × 10^−16^; data not shown) and unassigned gray modules from downstream analysis.

Modules were characterized and named by inspection of their gene membership, over-representation of biological pathways (GP Biological Process, Go Cellular Component, KEGG, MSigDB REACTOME and MSigDB transcription factor motifs) and correlation with AUCell (Aibar et al., 2017) (v 1.12.0) expression scores for specific sets of genes (Data S6) (identified WGCNA modules). The “curated type I IFN response” geneset comprised of GBP2, IFI27, IFI44L, IFIT1, IFITM1, IFITM3, IFNA1, IFNA10, IFNA13, IFNA14, IFNA16, IFNA17, IFNA2, IFNA21, IFNA4, IFNA5, IFNA6, IFNA7, IFNA8, IFNB1, IRF1, IRF3, IRF7, IRF9, ISG15, MX1 MX2, OASL, RSAD2, SIGLEC1, TRIM56, USP18 and XAF1.The “curated AP1 TF family” geneset comprised of the genes shown in Figure 3C. The custom “zf-C2H2 (PF00096) domain” geneset comprised of genes containing the PF00096 domain (Pfam database; Figure 3B). For geneset over-representation analysis only genesets with a BH corrected p value < 0.05 are reported (separate corrections performed within major cell type and ontology).

Module eigengene correlations with disease were computed by numerically coding the given x_vs_y disease groups as x = 1 and y = 0 respectively (Figure 3A). Module eigengene associations with variance between patient groups were assessed by ANOVA (COVID+HV_ANOVA, All_Groups_ANOV-A, Figure 3A). Module eigengene correlations with clinical variables were computed using pairwise-complete observations. Where appropriate clinical variables were quantile normalized as described below for analysis of clinical phenotype. Stars in Figure 3A indicate a significant association between the contrast, clinical variable or geneset score and the module eigengene (BH corrected p value < 0.05, p value correction performed separately for each contrast, variable or score).

#### Repertoire analysis

##### Strategic approach

Here, we used both single cell (sc) and bulk cell VDJ sequencing to decipher the immune responses in COVID-19 patients. The benefit of scVDJ-seq is the joint analysis of the VDJ BCR or TCR sequence along with the gene expression and CITE-seq for each individual cell, allowing for a detailed linking of antigen receptor function or type with cellular phenotype or function. However, this is limited by the number of cells that can be captured in a single experiment, usually between a few 10’s to 1,000’s. Therefore, bulk VDJ-seq can also be used to capture a more holistic view of the immune cell repertoire. While this does not capture VDJ sequences on a single cell level, many aspects of the repertoire may be investigated, such as changes in clonality, dynamics, selection and tolerance. Because bulk VDJ-seq is able to capture the immune receptors of 1,000’s to 100,000’s of cells, this means that the resulting repertoire is more representative of the individual than single cell VDJ-seq, and has greater power to detect low frequency clones or repertoire features. Finally, through capturing a higher magnitude of BCR/TCR sequencing, imputing the germline IGHV/J germline alleles becomes possible, and therefore allowing for analyses of differential genetics between groups.

##### Additional pre-processing of scBCR-seq data for repertoire analysis

The BCR outputs from CellRanger were run through IMGT V-QUEST (Giudicelli et al., 2004) to determine the TCR chain types and annotation. In addition, only BCRs from droplets that were confidently annotated as (a) singlets, (b) within the B cell annotated clusters in the GEX data, and (c) were confidently genetically demultiplexed were included. Processed FASTA sequences, corresponding to high confidence VDJ contigs from 10X Genomic’s Cell Ranger v4.0.0 pipeline, were annotated using IMGT HIGHV-QUEST. To ensure high quality contigs, non-productive rearranged sequences were removed and only sequences corresponding to cell barcodes that past QC cut-offs for gene expression data were analyzed. For B cell VDJ sequences where multi-chains were detected, e.g., two or more heavy or light chain sequences, only contigs ranked above the first derivative of log10 ranked contig ratios were selected for downstream analysis. Contig ratios were defined by:

$$Contig_{rank2umis}/Contig_{rank1umis}$$

Where multi-chains passed this quality threshold, the contig corresponding to the lowest UMIs detected was considered ambient RNA contamination and removed. Where multi-chains did not pass this threshold, we considered these to be low confidence of being true single cell droplets and likely to represent homotypic doublets; thus, they were removed from further analysis.

Cells with filtered BCRs were concentrated in the regions annotated as B cells or plasmablasts/plasma cells through gene expression analysis (Data S3). Plasmablasts/plasma cells exhibited higher BCR IGH/ IGK/L expression compared to non-plasmablasts/plasma cell populations.

##### Additional pre-processing of scTCR-seq data for repertoire analysis

The TCR outputs from CellRanger were filtered based on called productivity, and chain identity (TRA or TRB). Only T cells that contained either (a) 1 beta chain, (b) 1 alpha and 1 beta, (c) 2 alpha chains and 1 beta were retained. Given that bona fide T cells with UMI counts of 1 have been validated in previous datasets (data not shown), the minimum number of UMIs required per cell to accept was 1. In addition, only TCRs from droplets that were confidently annotated as (a) singlets, (b) within the T cell annotated clusters in the GEX data, and (c) were confidently genetically demultiplexed were included.

The IMGT annotation of cell types demonstrated that the majority of droplets for which TCRs were captured were able to pass the chain type filters (Table S2). After removal of low-quality droplets, there remain 94 samples corresponding to the maximal disease severity per patient. Four samples were excluded due to low T cell capture (< 200 cells). Age was used as a co-variate in downstream analyses.

##### scBCR clonality measurements

Clonal assignment was performed using a custom procedure. First, concatenated VH/VJ nucleotide sequences were clustered using an adapted R implementation of CD-HIT (https://github.com/thomasp85/FindMyFriends). Subsequently, within-cluster CDR3 normalized Levenshtein distances were generated using the “stringdist” R package. The clonal threshold was set as the local minimum of the CDR3 distance distribution. Convergent clones were assigned using the same procedure without constraints accounting for biological replicates. The presence of published SARS-CoV-2 binding antibody sequences from CoV-AbDab (Raybould et al., 2021) within the dataset was identified by first requiring identical VH and VJ gene pairings, followed by CDR3 distance thresholding (as described above).

The clonal expansion index (CEI) was calculated as the Gini index (unevenness) of the number of total BCRs per clone. The clonal diversification index (CDI) was calculated as the Gini index of the number of unique BCRs per clone. This is a measure of unevenness based on how many non-identical members of each clone are diversified from their inferred germline ancestor.

##### scTCR clonality measurements

A clone ID was defined by a concatenation of amino acid CDR3 chains present, with cells sharing identical clone IDs classed as members of the same clonotype. Clonal proportions were calculated by dividing the number of cells in each clone per sample by the total of number of cells per sample. Shannon diversity was calculated from count data of clones per sample using the R package entropy (Hausser, 2009). Statistical analysis was performed using a linear model with covariates for age and sample size.

For the scTCR analysis the cluster annotations were merged to create simplified clusters for analysis. The following reannotated clusters were used in TCR clonality analysis, with constituent “minor subsets” shown in brackets: CD8 T effector memory (CD8.TEMRA + CD8.TEM); CD8 T central memory (CD8.TCM + CD8.TCM.CCL5); CD8 T effector (CD8.TEFF + CD8.TEFF.prolif); CD8 naive (CD8.NAIVE); CD4 T effector (CD4.TEFF + CD4.TEFF.prolif); MAIT (MAIT). Where comparisons of clone size across samples has been performed in terms of absolute number of member cells each sample was randomly down-sampled without replacement (n = 1250 for CD4, n = 250 for CD8) to generate repertoires of identical size across all samples. The mean size of clone was then calculated from the down-sampled repertoires. This process was bootstrapped 100 times and the mean size from these iterations is presented. This approach allows for accurate comparisons between samples containing different cell numbers and controls for sample size.

##### scTCR cytotoxicity and Kmer analysis

Cytotoxicity score was calculated using the AddModuleScore function in Seurat (Butler et al., 2018) with a gene-set of the top 50 genes that significantly correlated with IFNG expression in an independent single cell dataset (Watson et al., 2020) and were identified as variable features in Seurat. The gene-set was then used as an input to generate phenotype scores.

CDR3b sequences of T cells identified as CD4^+^ or CD8^+^ T cells were broken down into 4-amino acid length sequences (Kmer). A Fisher’s exact test was performed per Kmer across each group of patients (HV, COVID-19 or sepsis patients) and p values adjusted using a Bonferroni’s correction, with a leave-one-out re-sampling of individuals employed to ensure inter-individual reliability. Kmer sequences significantly enriched in COVID-19 patients over HV or sepsis patients across more than 95% of the re-samples were identified as COVID-enriched Kmers. The proportion and cellular phenotype (i.e., subset and cytotoxicity) of T cells with a CDR3b sequence containing a COVID-enriched Kmer was subsequently compared between COMBAT clinical groups. The method used to identify CDR3 Kmers associated with COVID-19 is illustrated (Data S3).

##### Identification of SARS-CoV2 specific T cell clones using public CDR3 databases

Putative viral antigen specificity of clonotypes within COMBAT cohort was determined using publicly available databases of viral antigen specific CDR3 sequences. At the time, two databases containing TCR sequences with reported binding to SARS-CoV-2 epitopes were available: VDJdb (Bagaev et al., 2020) and ImmuneCODE (Nolan et al., 2020). From VDJdb, all unique CDR3 amino acid sequences (alpha as well as beta chain of human TCR) of all lengths and MHC-restrictions with reported binding to known SARS-CoV2 associated antigens were selected. Sequences with low confidence scores assigned to TCR:peptide:MHC complexes or missing sequencing/ specificity validation data were then filtered out. ImmuneCODE database includes deeply sampled TCRb repertoires from over 1,400 subjects exposed to or infected with the SARS-CoV-2 virus. Within these, over 135,000 TCRs were deemed to be SARS-CoV-2-specific with high-confidence using Multiplex Identification of Antigen-Specific T Cell Receptors Assay (MIRA) and were included(Klinger et al., 2015). Unique CDR3 amino acid sequences thus identified were collated and interrogated against TCR sequences within COMBAT cohort for matches within CDR3a, CDR3a2 and CDR3b regions. A similar strategy was used to identify CMV, EBV and Influenza specific clones. Finally, proportions of individual repertoires occupied by viral clones were compared between COMBAT clinical groups.

##### Bulk BCR and TCR quality control and filtering

Raw sequencing reads were filtered for base quality (median Phred score > 32) using QUASR (Watson et al., 2013). Forward and reverse reads were merged if they contained identical overlapping region of > 50bp, or otherwise discarded. Universal barcoded regions were identified in reads and orientated to read from V-primer to constant region primer. The barcoded region within each primer was identified and checked for conserved bases. Primers and constant regions were trimmed from each sequence, and sequences were retained only if there was > 80% per base sequence similarity between all sequences obtained with the same barcode, otherwise discarded. The constant region allele with highest sequence similarity was identified by 10-mer matching to the reference constant region genes from the IMGT database (Lefranc, 2011), and sequences were trimmed to give only the region of the sequence corresponding to the variable (VDJ) regions. Isotype usage information for each BCR was retained throughout the analysis hereafter. Sequences without complete reading frames and non-immunoglobulin/TCR sequences were removed and only reads with significant similarity to reference IGHTCR V and J genes from the IMGT database using BLAST (Altschul et al., 1990) were retained. Ig/TCR gene usages and sequence annotation were performed in IMGT V-QUEST, where repertoire differences were performed by custom scripts in Python.

Bulk BCR sequencing resulted in 2,356,813 BCRs consisting of 1,905,867 unique BCR sequences, resulting in 79 samples passing all QC measures and with the minimum number of BCR sequences per sample at 1,000. Bulk TCR sequencing resulted in 1,200,656 TCRs consisting of 1,159,363 unique TCR sequences, resulting in 77 samples passing all QC measures and with the minimum number of TCR sequences per sample at 1,000.

##### Isotype frequencies, somatic hypermutation, CDR3 lengths and IGHV gene usages

Analysis methods are based on (Bashford-Rogers et al., 2019). To account for the greater numbers of BCR RNA molecules per plasmablast compared to other B cell subsets, the normalized isotype usages, defined as the percentage unique VDJ sequences per isotype, thus controlling for differential RNA per cell and reducing potential biases from differential RNA per cell. Similarly, mean somatic hypermutation levels and CDR3 lengths were calculated per unique VDJ region sequence to reduce potential biases from differential RNA per cell. IGHV gene usages were determined using IMGT, and proportions were calculated per unique VDJ region sequence. The representation of IGHV genes in the BCR repertoire reflects their presence in the germline, the naive repertoire and their expansion after antigenic exposure. We therefore compared the frequency of IGHV gene use in PBMC-derived BCRs identified by sequence as being enriched for naive (IgM^+^D^+^SHM^−^: > 78% naive B cells by flow cytometry) and antigen-experienced B cells (including both unswitched (IgM^+^D^+^SHM^+^) and class-switched memory (IgA^+^/G^+^/E^+^) subsets) as shown in (Bashford-Rogers et al., 2019).

##### Bulk BCR class-switching event analyses

Relative class-switch event frequency was the frequency of unique VDJ regions expressed as two isotypes (i.e., from more than one B cell, where one has undergone class-switch recombination). This was determined as proportion of unique BCRs present as both isotypes IgX and IgY within a random subsample of 8,000 BCRs, where the mean of 1,000 repeats was generated. This provides information on the frequency of BCRs observed associated with any two isotypes (class-switching events) while accounting for total read depth, but not accounting for differences in the relative frequencies of BCRs per isotype.

The per-isotype normalized class-switch event frequencies determine frequency of unique VDJ regions expressed as two isotypes while normalizing for differences in isotype frequencies. To account for differences in isotype proportions, BCRs from each isotype were randomly subsampled to a fixed depth of 100 BCRs, and the proportion of unique VDJ sequences present between each pair of isotypes was counted. The mean of 1,000 repeats was generated.

##### RNA-velocity B cell analysis

The single-cell RNA-seq datasets were subjected to the standard RNA-velocity pipeline, and the trailing analyses were performed using the scVelo package (v0.1.24). The scVelo package was used to normalize the counts and select highly variable genes based on spliced counts. Following this, the dynamical model implemented in scVelo was used to estimate the RNA velocity for the cells. The estimated velocities were then visualized using Partition-based graph abstraction (PAGA) plots using the previously computed UMAP embeddings.

Supporting plots are provided in Data S3 for B cell cluster proportions; plasmablast diversity indices, clonal expansion index and clonal diversification index; correlation analysis of IGHV genes by single cell VDI and RNA VDJ; and IGHV gene usage of IGHD/M unmutated sequences, IGHD/M mutated sequences and class-switched sequences; constant region genes across B cell clusters; clonal overlap across comparator groups; and frequencies of known SARS-CoV-2 binding BCRs.

#### scATAC-seq data analysis

Raw data pre-processing was performed with Cell Ranger ATAC (10X Genomics). ‘cellranger-atac count’ pipeline was used to align reads and generate single-cell accessibility counts for the cells. The reference genome assembly file was Ensembl GRCh38 v100 Primary Assembly corresponding to hg38, downloaded from http://ftp.ensembl.org/pub/release-100/fasta/homo_sapiens/dna/Homo_sapiens.GRCh38.dna.primary_assembly.fa.gz. This file was used as the reference genome file for alignment and generation of single-cell accessibility counts. Annotations were from Ensembl GRCh38 v100 Gene Annotation downloaded from http://ftp.ensembl.org/pub/release-100/gtf/homo_sapiens/Homo_sapiens.GRCh38.100.gtf.gz. VIREO (Huang et al., 2019) and demuxlet (Kang et al., 2018) packages were used to demultiplex patient samples within a channel and identify inter-sample nuclei doublets. Concordantly filtered barcodes from individualized samples were then used to create individual fragment files for each patient using HTSlib –c function. Following the creation of fragment files, downstream analysis of the scATAC-seq data was performed using the ArchR v0.9.3 R package (Granja et al., 2021). Fragment files were first checked using the reformatFragmentFiles function. Arrow files were then generated by reading each sample’s specific fragment files and a tile matrix was created using 500-bp bins. Cells with a transcription start site enrichment score < 4, with fewer than 1,000 detected fragments or containing intra-sample nuclei doublets were removed, resulting in ∼46,000 cells, which were subjected to dimensionality reduction with iterative Latent Semantic Indexing (LSI) and Singular Value Decomposition (SVD), followed by Uniform Manifold Approximation and Projection (UMAP) embedding calculation to visualize the data structure in the two-dimensions. ∼4,000 cells were manually removed at this stage, as we identified putative batch effects. The resulting ∼42,000 cells were subjected to UMAP and were clustered using the implementation from Seurat R package (Stuart et al., 2019). Cluster-specific gene activity scores were identified based on the local chromatin state, and marker genes were identified (FDR ≤ 0.01 & log_2_FC ≥ 1.25). Unconstrained integration with cognate scRNA-seq profiles was performed using the addGeneIntegrationMatrix (ArchR) method and scRNA-seq cell type annotations were used to label scATAC-seq clusters. We performed peak calling using MACS2 with the addReproduciblePeakSet (ArchR) function using pseudo-bulk replicates. Such replicates were grouped with different variables, such as cell-type, condition and patient, as well as a combination of these variables. Differentially accessible peaks (FDR ≤ 0.1 & log_2_FC ≥ 0.5) were identified between pairwise comparisons, and peak-to-gene linkages were calculated using the addPeak2GeneLinks (ArchR) method using a correlation cut-off of 0.45 and resolution = 1,000. We then used the ‘cisbp’ motif set to annotate motifs in accessible peaks using the addMotifAnnotations (ArchR) function. Motif enrichments in differentially accessible peaks were calculated using the peakAnnoEnrichment (ArchR) method. Finally, motif footprinting was performed by measuring Tn5 insertions in genome-wide motifs and normalized by subtracting the Tn5 bias from the footprinting signal.

#### Luminex data analysis

##### Cytokine enrichment profile analysis

The concentrations of 51 circulating proteins in plasma were presented as mean fluorescence intensity (FI) ± SEM and compared between HV and each disease severity group of COVID-19 using unpaired Student’s t test, and then depicted with Prism (Data S3).

##### Principal Component Analysis (PCA)

PCA on 171 plasma samples was conducted with the scater package in R. The values of FI were normalized with logNormCounts function, and then calculated with the RunPCA function. The loadings were generated by the value of first two components of eigenvectors multiplied by 10.

##### Heatmap

The heatmap was colored by the log_10_ of the fold change in the natural log of the FI, normalized against the mean value of HV for the plasma cohort and HS for the serum cohort. When comparing mortality in severe and critical COVID-19 patients, the data is normalized to the survivor group. The color-scale is bounded at ± 5-fold change (0.7 in log_10_), with an increased FI shaded red; decreased FI shaded green; unchanged FI shaded yellow (Data S3).

##### Volcano plots

The p values in the Volcano plots are calculated using a two-tailed two-sample unpaired t test (ttest2, MATLAB). The t test was taken for the natural log of the FI of the test and control conditions. The p values are plotted against the log_2_ of the fold change in the natural log of the FI between the test and control conditions.

##### Uniform manifold approximation (UMAP)

The UMAPs [McInnes L., Healy, J., and Melville, J. (2018)] UMAP: Uniform Manifold Approximation and Projection for Dimension Reduction, arXiv:1802.03426] (Data S3) were calculated using the MATLAB package (https://uk.mathworks.com/matlabcentral/fileexchange/71902-uniform-manifold-approximation-and-projection-umap) The UMAP is partially supervised, with 2/3rds of the patients in each condition randomly chosen to train the network. The UMAPs are set to have 45 nearest neighbors, with a minimum distance of 0.3 with a correlation metric. The UMAP was reduced over 2,000 epochs.

##### Linear correlation between analytes / Linear correlation between clinical traits

The correlation coefficient, r, was calculated using a Pearson correlation coefficient (corcoeff, MATLAB). All quoted r values have an associated p value (also computed by corcoeff) of less than 0.05.

##### Correlation network analysis

All values used for the correlation network analysis were the FI of Luminex results and/or the clinical readout. Severities were scored as HV = 0, CH = 1, CM = 2, CS = 3 and CC = 4. Quality control (QC) was conducted on the matrix of expression values. Seven out of total 51 proteins were removed from the correlation analysis due to their low FI (< 10 after subtracting FI in blank) and small SD (< 10). Pearson correlation coefficient was performed using pairwise-complete correlation. Correlation matrix plots were generated using a modified package corrplot. Correlation matrix summary plots were made manually by R. Network plots were created by ggraph, and nodes were selected based on |r|>0.5 and p < 0.05.

#### Tims-TOF mass spectrometry analysis

##### Primary data analysis (Identification and Quantitation)

Data was analyzed by the Fragpipe pipeline consisting of Fragpipe 13.0 (Kong et al., 2017), MSFragger 3.0, Philosopher 3.2.9 (da Veiga Leprevost et al., 2020) and Python 3.8.2. Blank runs were excluded and each file defined as experiment to facilitate LFQ. Data were searched against a fused target/decoy database generated by Philosopher and consisting of human UniProt SwissProt sequences and UniProt SARS-nCov02 (retrieved 17/07/2020), plus common contaminants. The database had 40,860 entries (including 50% decoy entries). MSFragger parameters were set to allow a precursor mass tolerance of plus/minus 10ppm and a fragment tolerance of 20ppm. Isotope error was left at 0/1/2 and masses were set to re-calibrate. Protein digestion was set to semi specific trypsin with up to 2 allowed missed cleavage sites, allowing peptides between 7 and 50 residues and mass range 500 to 5,000 Da. N-terminal protein acetylation and Methionine oxidation were set as variable modifications. ID validation was done with PeptideProphet and ProteinProphet (Nesvizhskii et al., 2003) with default settings.

Label free quantitation was conducted with IonQuant (Yu et al., 2020) and Match-Between-Runs enabled (with default parameters) and using Top-3 quantitation. Feature detection tolerance was set to 10ppm and RT Window to 0.6 min with an IM Window of 0.05 1/k0. For matching, ion, peptide and protein FDRs were relaxed to 0.1 and min correlation set to 0 in order to allow pre-fractionated library samples to be included. MBR top runs was set to 600.

##### Data handling

The proteomics dataset was processed as follows: (1) Protein filtering such that proteins with at least 50% of valid values in one group were kept; (2) Sample filtering such that samples with more than 50% of missing values were removed from the dataset; (3) Data normalization with log_2_ transformation and median-centering of the dataset. Imputation of missing values was performed using a mixed model that combines a K-Nearest Neighbor approach (KNN) when at least 60% of valid values are present, otherwise a Minimum probability approach is used where missing values are randomly drawn from a Gaussian distribution (shift = 1.8, nstd = 0.3). The resulting data matrix contains 353 samples and 105 proteins. Thirteen samples were further excluded from analysis for malignancy, immunosuppression, or being alternative samples.

##### Statistical analysis

Unsupervised hierarchical clustering was performed based on Euclidean distance and Ward’s method for calculating linkage (Data S3). Differential abundance analysis was performed by fitting protein abundance in linear models with the limma package, using only one sample at the maximal severity of the patient and including age and sex as covariates. The Benjamini-Hochberg procedure was applied to correct for multiple comparisons. FDR < 0.05 and fold change > 1.5 was taken as statistical significance. Pathway enrichment analysis was performed using the XGR package with annotations either from Gene Ontology Biological Process or the Reactome pathway database. Significantly enriched terms were defined by FDR < 0.05 in hypergeometric tests with all proteins detected in plasma (including in library samples) as the background. Statistical analysis was performed in R.

##### Protein-protein interaction network

Protein-protein interaction data was retrieved from STRING v11 database with a confidence score cut-off of 0.7 and zero additional interactors. The network was visualized through Cytoscape v3.8.0 platform (Shannon et al., 2003) using perfuse force directed layout, and divided into clusters with the Markov cluster algorithm applied in the “clusterMaker” plugin. Node color was mapped to Pearson’s correlation coefficients of PC1 scores and the protein level across samples, as lower PC1 score was shown to correlate with higher disease severity.

##### Clinical knowledge graph

The MS-based proteomics data were also analyzed using the Clinical Knowledge Graph (CKG) (Santos et al., 2020). CKG provides a Python framework for downstream analysis and visualization of proteomics data: protein ranking, dimensionality reduction, functional Principal Component Analysis (PCA), Analysis of Variance (ANOVA), protein-clinical variable correlation analysis and network summarization.

CKG runs 3 feature reduction algorithms: Principal Component Analysis (PCA), Uniform Manifold Approximation and Projection (UMAP) and functional PCA. The functional PCA is based on the results of the method single-sample Gene Set Enrichment Analysis (Subramanian et al., 2005), which identifies enrichment of Biological processes (Gene Ontology) (GOBP) in single samples derived from the ranked intensities of the identified proteins. This method generates a vector of biological processes enrichment scores for each sample. Loadings of the top 15 proteins and GOBP driving the separation of the conditions studied are included in the PCA and functional PCA respectively. In this analysis, the drivers are biological processes such as acute-phase response and inflammation and retinoid and lipoprotein metabolic processes and cholesterol transport.

We performed ANOVA analysis to identify differentially regulated proteins across conditions. Further, we run posthoc analysis (pairwise t test) to show specific differences when comparing disease conditions to healthy volunteers or community COVID-19 and also between severity levels.

We performed functional enrichment analysis (Fisher’s exact test) to identify enriched GO among the up- and downregulated proteins in each pairwise comparison. Enrichments are plotted as scatterplots showing up- or downregulated enriched GOBP and their adjusted p values (Benjamini-Hochberg FDR: cutoff 0.05).

CKG performed a Spearman correlation analysis using the clinical metadata and the proteomics dataset. This Protein-clinical correlation is shown in a network where nodes are either clinical variables (diamond shape) or proteins (circle shape), and edges represent correlation (red = positive correlation, blue = negative correlation; Spearman correlation cutoff ≥ 0.5; Benjamini-Hochberg FDR: cutoff 0.05). CKG applied a clustering algorithm (Louvain community detection) to identify clusters of highly connected nodes (nodes colored by cluster).

The results from all CKG analyses are summarized in a single visualization all the findings in the different analyses and all relevant biomedical context associated (diseases, drugs, biological processes, pathways, protein complexes, publications). The summarization algorithm prioritizes what nodes are shown in the network based on betweenness centrality. The top 15 central nodes are shown for each node type.

Plasma protein abundance for specific proteins is summarized (Data S3).

#### Similarity network fusion analysis

The similarity network fusion (SNF) analysis was performed using the function in CKG that makes use of the python library pySNF. The method was used to analyze the proteomics datasets (MS-based proteomics and Luminex) in combination to identify in an unsupervised manner clusters of similar patients. The number of clusters was not defined initially and optimized using the eigengap method and the clusters identified using Spectral clustering. The SNF analysis used Euclidean distance to calculate similarity (k_affinity = 20, mu_affinity = 0.6). The function returns the clusters and a mutual information score for each feature included in the analysis (MIscore). We used this score to prioritize a reduced number of features mainly driving the separation of the clusters (11 features) (MIscore ≥ 0.15). The clusters are visualized using PCA plots.

##### SNF Cluster validation

In order to validate the identified clusters using SNF on the proteomics data, we used an independent cohort studied using a different technology, namely targeted proteomics by Olink (Filbin et al., 2021). Access to the dataset was granted by Olink https://info.olink.com/mgh-covid-study-overview-page. The processing of the data was done using CKG analytics core functions to map protein identifiers to names, transform the data into wide format and impute missing values using a mixed model as previously described. The clustering on these data followed a similar approach to how the SNF clusters were calculated (optimal clusters and Spectral clustering) but using only the selected features in the SNF analysis (7/11 features).

##### SNF clusters survival analysis

To evaluate the clinical relevance of the identified clusters in COMBAT, we performed a survival analysis and plotted the Kaplan-Meier curve using R packages survival (Therneau and Grambsch, 2000) and survminer. The input data is a data frame specifying the time to event, the event (death or end of observation) and the groups (SNF clusters). The comparison of the survival distributions between clusters was performed and the p value given using log-rank test. The hazard ratio was calculated using Cox proportional hazard model. In the Olink dataset survival status is only available at 4 time points: 0, 3, 7 and 28 days. Deaths at these time points were collected according to WHO category 1, defined as death, in these time points (WHO 0, WHO 3, WHO 7 and WHO 28). We compared the 28-day mortality between the two SNF clusters by chi-square test.

##### Olink and COMBAT correlation analysis

To evaluate the robustness of the identified clusters between technologies/studies and to eliminate the possibility that only the 7 selected features used for the clustering of the Olink data were similar, we calculated the correlation (Pearson correlation) of fold-changes between clusters for all the common proteins in these studies (n = 43).

#### Tensor and matrix decomposition

A tensor and matrix decomposition method, Sparse Decomposition of Arrays (SDA), as defined in (Hore et al., 2016), was used to integrate 152 samples from the different ‘omics datasets defined above, allowing that some samples were missing certain ‘omics data.

The whole blood total RNA-seq (9 missing samples) and the pseudobulk from 10X CITE-seq scRNA-seq (22 missing samples) were combined into a three-dimensional tensor consisting of 152 samples by 9 tissue types by 14,989 genes (which passed QC in both datasets). This expression was normalized by sample in each tissue type by log_2_-transformation of counts-per-million + 1.

The number of cells per cell type was included as defined by 10X CITE-seq (a two-dimensional matrix of 152 samples by 97 cell types, with 22 samples missing); and mass cytometry (CyTOF) (One two-dimensional matrix of 152 samples by 10 cell types for the all cells dataset, with 21 samples missing, and a further two-dimensional matrix of 152 samples by 51 cell types for the granulocytes-depleted cells dataset, with 20 samples missing). We filtered out any samples with fewer than 500 cells in any matrix. The data in each matrix was normalized by a log_2_ transformation of counts-per-million + 1.

The proteomics data from Luminex (in a two-dimensional matrix of 152 samples by 51 proteins, with 20 samples missing) and mass spectrometry (Tims-TOF) (in a two-dimensional matrix of 152 samples by 105 proteins, with 17 samples missing) were used, with the data normalized as described in the Luminex and Tims-TOF sections.

As described in (Hore et al., 2016), to find robust components we ran the tensor and matrix decomposition ten times for 1,000 components. Each time, around 290 components were estimated to be zero. Once again, similar to the (Hore et al., 2016) method the absolute correlation (r) was calculated for the sample scores for each pair of components, clustered using hierarchical clustering on 1-r (dissimilarity measure) and formed flat clusters in which the components in each flat cluster have no greater a cophenetic distance than 0.4. We chose the flat clusters that had components from at least 5 of the 10 runs. The final sample, tissue and gene or protein or cell score was the mean of all the components within the chosen clusters. This resulted in 381 clusters.

Components were identified as associated with COVID-19 if they (1) showed significant variation (BH adjusted p < 0.01) in an analysis of variance between the COVID-19 categories and healthy volunteers and (2) showed a significant |spearman’s rho| ≥ 0.5 (and Benjamini/Hochberg adjusted p < 0.01) in at least one of the contrasts between the COVID-19 groups versus healthy volunteers (in total we found n = 130 such components) (Figure S11C. To identify COVID-19 specific components the median loadings of the components for the different comparator (source) groups (i.e., including influenza and all-cause sepsis) were clustered (k-means). Individual component associations, for example with comparator group, severity or clinical features were assessed with Spearman correlation (between component loadings and numerical variables) or ANOVA (between component loadings and categorical variables). The overview heatmap (Figure S11A) was generated by combining the top significant (Benjamini/Hochberg adjusted p < 0.05, abs(r) > 0.5, max 10) components from each of the individual analyses. Pathway enrichment of gene expression (those with posterior inclusion probability > 0.5, weighted by their ranking in loading score magnitude) for individual components was done using gene set enrichment analysis as implemented in Pi’s xPierGSEA (Fang et al., 2019).

#### Integrated data analysis of multi-omics data using machine learning feature selection to distinguish COVID-19 severity groups

We used supervised machine learning (from sklearn) to classify samples according to their WHO severity based on PCs obtained from data across the different modalities (timsTOF, Luminex, and total RNA-seq) (Figure S10A). We performed permutation feature scoring to find the most important PCs to predict severity. After that, we extracted the most important features of the most important PCs and reran the algorithm directly on these features, again ranking them according to their importance.

#### Machine learning using SIMON to distinguish COVID-19 and sepsis

##### Generation of an integrated COMBAT dataset

The pre-processed data from individual COMBAT assay datasets were automatically processed using the standard extract-transform-load (ETL) procedure to generate an integrated dataset. Datasets were merged using shared variables, the COMBAT sample ID and assay-specific sample IDs. Next, we generated novel features, such as name of specific assays to indicate if a sample was analyzed in a specific assay (yes/no), ordering of samples shared between assays (*first_sample_across_assays*), day when sample was obtained from the maximum disease (*day_sampling_from_max_disease*), state of the disease when sample was acquired (recovered/ongoing), *hospitalized, ventilation, oxygenation, sampling* (£10 days as early or > 10 days as late phase of the disease), samples acquired before or after maximum disease (*sampling_from_max_disease) and disease progress for longitudinal samples (deterioration or recovery).* Donors that were excluded or frail were not included in the integrated dataset. We standardized names of the immune cells subsets and analytes to reflect the measurement, such as frequency (*freq*_cell subsets) or intensity (Luminex parameter_*intens*). In total, the integrated COMBAT dataset contained information on 428 samples from 268 donors on more than a million parameters.

##### Data pre-processing

For the multi-omics data integration, after filtering for samples not analyzed across all assay modalities and restricting to the first available sample after admission from sepsis and hospitalized COVID-19 patients, the final dataset included 15 sepsis patients and 53 hospitalized COVID-19 patients with 184 features analyzed using CyTOF, 79 using flow cytometry, 8 using GSA, 105 mass spectrometry, 102 Luminex and 23,063 features from whole blood total RNA-seq. The data for each assay was centered and scaled, missing values were imputed, features with zero-variance and near-zero-variance were removed and finally, highly correlated features with cut-off 0.85 were also removed.

##### Feature selection process

To avoid ‘curse of dimensionality’, reduce overfitting and improve accuracy, we implemented a wrapper approach as described (Kohavi and John, 1997). Briefly, the initial dataset containing all 68 donors was partitioned into training (52 donors) and test set (16 donors) with balanced class distribution of sepsis and COVID-19 patients using the data partitioning function (Kuhn, 2008) as described previously (Tomic et al., 2019). The same training/testing dataset split was used for each assay. The reduction of features using the recursive feature elimination (RFE) algorithm was performed on the training set and the model was evaluated using the 10-fold cross-validation repeated 5 times. For whole blood total RNA-seq, prior to training the model using RFE algorithm, we have performed analysis of differentially expressed genes between sepsis and hospitalized COVID-19 patients, reducing the number of genes to 2,989 based on the FDR < 0.05 and fold change > 1.5, and accounted for age and sex in the model. The RFE method removes the features that do not contribute to the final model, while it keeps the features that contribute the most to the final model as evaluated using the variable importance score (Kuhn and Johnson, 2019). Finally, after the RFE analysis, the selected features from each assay (36 features from CyTOF, 20 from flow cytometry, 20 from Luminex, 32 from mass spectroscopy, 28 from whole blood total RNA-seq and 1 from GSA dataset) were merged in the final training dataset containing 52 donors and 137 features.

##### Machine learning using SIMON

To identify immunological and molecular signatures that can discriminate between sepsis and COVID-19 patients, we used SIMON (Sequential Iterative Modeling “Over Night”) (Tomic et al., 2019). SIMON is a free and open-source software that provides a standardized ML method for data pre-processing, data partitioning, building predictive models, evaluation of model performance and selection of features. For the analysis, we applied four machine learning algorithms, naive Bayes, Shrinkage discriminant analysis (*sda*), Support vector machine with linear kernel (*‘svmLinear2’*) and C5.0 decision tree. Since the entire ML process in SIMON is unified, resulting models built with different algorithms can be compared and the best performing models can be selected. First, models are built on training set and the performance is evaluated using a 10-fold cross-validation repeated five times and cumulative error rate is calculated. To prevent overfitting, in the second step, each model is evaluated on the withheld test set. The performance of classification models was determined by calculating the area under the receiver operating characteristic curve (AUROC) for test set (test AUROC). The best performing model was built using the naive Bayes (testing AUROC 0.85, 95% CI 0.59-1.00). In the final step, SIMON calculated the contribution of each feature to the model as variable importance score (scaled to maximum value of 100).

#### Data management

To support consistent and coherent communication of data and metadata within the project, a unified identifier system for all samples was implemented. The COMBAT sample identifier system encodes information regarding the sample providence in terms of cohort, de-identified patient ID, location where the sample was taken, the time point of the sample relative to the initial collection and details regarding the processing of the sample itself.

Datasets from each modality were stored within the consortium via the COMBAT datawarehouse, consisting of over 100TB of fast storage connected to a research computing cluster. This enabled data processing to occur within the datawarehouse, reducing the risk of duplication of datasets and the possibility of uncontrolled changes. Once datasets were ready to be shared within the consortium, to support (for example) data integration work, they were formally given a unique identifier and placed in a dedicated dataset directory. The existence of this deposition and its associated metadata, including information regarding associated samples and status was then made available via a web application which captures this information in a back-end database. This application allows consortium members to search by modality and status, providing information about the purpose of each dataset and its location in the datawarehouse.

The governance of data management was supported by the existence of a short but well-defined Data Access Agreement, which all consortium members were required to sign before gaining access to the datawarehouse. Furthermore, granular permissions within the datawarehouse enabled careful access controls to be applied to particularly sensitive data (such as rich clinical data). Web applications supporting the consortium are all protected by a federated Shibboleth-based authentication approach, allowing collaborators from outside of Oxford to gain access as required.

#### Data visualization

##### Multi-locus viewer

In order to visualize the data from the different modalities (experiments), a module, Multi Experiment Viewer (MEV https://github.com/Hughes-Genome-Group/MEV) was built for Multi Locus View (MLV DOI 10.1101/2020.06.15.151837) such that the data was pivoted on sample rather than genome location. The data was loaded in from each modality and for the CITE-seq data, pseudobulk values (on a sample/cell type basis) were used. In order to compare radically different datatypes (read counts, fluorescence, % cell type etc.), percentiles for each observation were calculated from the 10th to the 90th, in steps of 10. Values were then placed into 10 bins, e.g., if a value was ≤ 10th percentile it would be given a value of 1 and if > 2nd and ≤ 3rd, it would get a value of 2 etc. This was used as the default value, although depending on the modality other values e.g., raw counts, log transformed values were also added and can be selected by the user. Users can create their own views by searching for genes or loading in specific datasets and then combine them with a limited set of clinical data. Charts such as histograms, heatmaps and scatterplots can then be added and cross-filtered to identify samples or features of interest. An instance can be found at https://mlv.combat.ox.ac.uk, with links to predefined views of the data.

##### Shiny apps

Shiny apps (https://shiny.combat.ox.ac.uk) were developed using the R package shiny to display the results of the whole blood total RNaseq differential expression analysis and the principal component analysis. In each, derived data are loaded (limma fitted models and pre-calculated principal components respectively) together with limited metadata, and plots are generated within the app using ggplot2.

## Consortia

The members of the COvid-19 Multi-omics Blood ATlas (COMBAT) Consortium are David J. Ahern, Zhichao Ai, Mark Ainsworth, Chris Allan, Alice Allcock, Brian Angus, M. Azim Ansari, Carolina V. Arancibia-Cárcamo, Dominik Aschenbrenner, Moustafa Attar, J. Kenneth Baillie, Eleanor Barnes, Rachael Bashford-Rogers, Archana Bashyal, Sally Beer, Georgina Berridge, Amy Beveridge, Sagida Bibi, Tihana Bicanic, Luke Blackwell, Paul Bowness, Andrew Brent, Andrew Brown, John Broxholme, David Buck, Katie L. Burnham, Helen Byrne, Susana Camara, Ivan Candido Ferreira, Philip Charles, Wentao Chen, Yi-Ling Chen, Amanda Chong, Elizabeth A. Clutterbuck, Mark Coles, Christopher P. Conlon, Richard Cornall, Adam P. Cribbs, Fabiola Curion, Emma E. Davenport, Neil Davidson, Simon Davis, Calliope A. Dendrou, Julie Dequaire, Lea Dib, James Docker, Christina Dold, Tao Dong, Damien Downes, Hal Drakesmith, Susanna J. Dunachie, David A. Duncan, Chris Eijsbouts, Robert Esnouf, Alexis Espinosa, Rachel Etherington, Benjamin Fairfax, Rory Fairhead, Hai Fang, Shayan Fassih, Sally Felle, Maria Fernandez Mendoza, Ricardo Ferreira, Roman Fischer, Thomas Foord, Aden Forrow, John Frater, Anastasia Fries, Veronica Gallardo Sanchez, Lucy C. Garner, Clementine Geeves, Dominique Georgiou, Leila Godfrey, Tanya Golubchik, Maria Gomez Vazquez, Angie Green, Hong Harper, Heather A. Harrington, Raphael Heilig, Svenja Hester, Jennifer Hill, Charles Hinds, Clare Hird, Ling-Pei Ho, Renee Hoekzema, Benjamin Hollis, Jim Hughes, Paula Hutton, Matthew A. Jackson-Wood, Ashwin Jainarayanan, Anna James-Bott, Kathrin Jansen, Katie Jeffery, Elizabeth Jones, Luke Jostins, Georgina Kerr, David Kim, Paul Klenerman, Julian C. Knight, Vinod Kumar, Piyush Kumar Sharma, Prathiba Kurupati, Andrew Kwok, Angela Lee, Aline Linder, Teresa Lockett, Lorne Lonie, Maria Lopopolo, Martyna Lukoseviciute, Jian Luo, Spyridoula Marinou, Brian Marsden, Jose Martinez, Philippa C. Matthews, Michalina Mazurczyk, Simon McGowan, Stuart McKechnie, Adam Mead, Alexander J. Mentzer, Yuxin Mi, Claudia Monaco, Ruddy Montadon, Giorgio Napolitani, Isar Nassiri, Alex Novak, Darragh P. O'Brien, Daniel O'Connor, Denise O'Donnell, Graham Ogg, Lauren Overend, Inhye Park, Ian Pavord, Yanchun Peng, Frank Penkava, Mariana Pereira Pinho, Elena Perez, Andrew J. Pollard, Fiona Powrie, Bethan Psaila, T. Phuong Quan, Emmanouela Repapi, Santiago Revale, Laura Silva-Reyes, Jean-Baptiste Richard, Charlotte Rich-Griffin, Thomas Ritter, Christine S. Rollier, Matthew Rowland, Fabian Ruehle, Mariolina Salio, Stephen Nicholas Sansom, Raphael Sanches Peres, Alberto Santos Delgado, Tatjana Sauka-Spengler, Ron Schwessinger, Giuseppe Scozzafava, Gavin Screaton, Anna Seigal, Malcolm G. Semple, Martin Sergeant, Christina Simoglou Karali, David Sims, Donal Skelly, Hubert Slawinski, Alberto Sobrinodiaz, Nikolaos Sousos, Lizzie Stafford, Lisa Stockdale, Marie Strickland, Otto Sumray, Bo Sun, Chelsea Taylor, Stephen Taylor, Adan Taylor, Supat Thongjuea, Hannah Thraves, John A. Todd, Adriana Tomic, Orion Tong, Amy Trebes, Dominik Trzupek, Felicia Anna Tucci, Lance Turtle, Irina Udalova, Holm Uhlig, Erinke van Grinsven, Iolanda Vendrell, Marije Verheul, Alexandru Voda, Guanlin Wang, Lihui Wang, Dapeng Wang, Peter Watkinson, Robert Watson, Michael Weinberger, Justin Whalley, Lorna Witty, Katherine Wray, Luzheng Xue, Hing Yuen Yeung, Zixi Yin, Rebecca K. Young, Jonathan Youngs, Ping Zhang, Yasemin-Xiomara Zurke, and the ISARIC4C Consortium.

## Data Availability

Derived and processed data for all the datasets generated during this study and reported in this paper are available including through this paper, the European Genome-phenome Archive (EGA), Zenodo and Chan Zuckerberg Initiative (CZI) Science cellxgene Data Portal (as detailed in key resources table). For sequence level RAW datasets deposited at EGA, access is managed by the COMBAT Consortium Data Access Committee. Web-based interfaces for visualizing COMBAT datasets and outputs reported here are available at https://mlv.combat.ox.ac.uk/ and https://shiny.combat.ox.ac.uk. All code used for every algorithm followed in data processing and analysis is fully referenced within the specific methods text sections and Key Resources Table.

## Supplemental information

Table S1: Demographics and clinical phenotyping for overall cohorts and hospitalized COVID-19 cohort, related to STAR Methods and Data S2

Table S2: Definition of lymphoid clusters for CITE-seq data, related to STAR Methods, Figure 1, and Data S4

Table S3: Differential expression and pathway enrichment for CITE-seq data, related to STAR Methods and Data S6

Table S4: Tensor and matrix decomposition analysis for 152 samples across modality types and all patient groups, related to Figure 7

Data S1: COMBAT Consortium author details and contributions

Data S2: Clinical phenotyping, related to STAR Methods and Table S1

Data S3: Analysis of mass and flow cytometry, repertoire, and proteomics, related to STAR Methods

Data S4: Multimodal annotation of CITE-seq data, related to STAR Methods, Figure 1, and Table S2

Data S5: Compositional analysis of CITE-seq data, related to STAR Methods

Data S6: Gene expression analysis of CITE-seq data (principal component analysis, differential expression, and WGCNA parameters and modules), related to STAR Methods, Figure 3, and Table S3
