# Supplementary material for: A blood atlas of COVID-19 defines hallmarks of disease severity and specificity
Source: Cell. 2022 Mar 3;185(5):916–938.e58. doi: 10.1016/j.cell.2022.01.012 (PMC8776501; doi:10.1016/j.cell.2022.01.012)
Supplement: Table S2: Definition of lymphoid clusters for CITE-seq data, related to STAR Methods, Figure 1, and Data S4 [file mmc2.pdf]

Table S2: Definition of lymphoid clusters for CITE-seq data, Related to STAR Methods, Figure 1 and Data S4

Related to **Figure 1B, C** and the **STAR Methods**: CITE-seq: multi-modal cluster annotation. The phenotypes of the identified sub-populations are shown in **Data S4: Multi-modal annotation of CITE-seq data**. Supporting GEX and ADT clustering analyses are shown in **Supplementary Figure 1E** and **Data S3: Analysis of Mass and Flow Cytometry, Repertoire and Proteomics, Related to STAR Methods**.

Table of Contents

T and NK cell sub-population phenotypes 1
B cell and plasmablast sub-population phenotypes 3

T and NK cell sub-population phenotypes

Population	Subset	ADT phenotype	TCR phenotype	BCR phenotype	GEX phenotype	Cell count
**CD4**		singlet, CD3+ CD4+	singlet	<1% IgG, <20 HC umi	CD4+	n = 270,003
**CD8**		singlet, CD3+ CD8+	singlet	<1% IgG, <20 HC umi	CD8A+	n = 106,245
**DP**		singlet, CD3+ CD4+ CD8+	singlet	<1% IgG, <20 HC umi		n = 7,102
**DN**		singlet, CD3+ CD4- CD8-	singlet	<1% IgG, <20 HC umi		n = 4,644
**MAIT**	**CD8+**	singlet, CD3+ CD8+ Vα7.2+ CD161+	singlet, TRAV1-2, TRAJ33 , TRAJ12 , TRAJ20	<1% IgG, <20 HC umi	Various	} n = 3,935
		singlet, CD3+ CD8+ Vα7.2+ CD161+	singlet, TCR chains include Mait sequences	<1% IgG, <20 HC umi	MAIT	
	**DN**	singlet, CD3+ Vα7.2+ CD161+	singlet, TRAV1-2, TRAJ33 , TRAJ12 , TRAJ20	<1% IgG, <20 HC umi	Various-	} n = 639
		singlet, CD3+ Vα7.2+ CD161+	singlet, TCR chains include Mait sequences	<1% IgG, <20 HC umi	MAIT	
**γδ T**	**Vδ2+ve**	singlet, CD3^{+ve} Vδ2+ Vγ9+	singlet	<1% IgG, <20 HC umi	TRDV2+	n = 6,239
	**Vδ2-ve Vγ9-ve**	singlet, CD3+ TCRγδ+	singlet	<1% IgG, <20 HC umi	TRDV1+ (TRDV3+)	n = 2,238
	**Vδ2-ve Vγ9+ve**	singlet, CD3+ Vδ2- Vγ9+	singlet	<1% IgG, <20 HC umi	TRDV1+ (TRDV3+)	n = 1,227
**iNKT**	**true**	singlet, Vα24Jα18+	singlet, TRAV10, TRAJ18	<1% IgG, <20 HC umi		n = 293
	**ADT**	singlet, Vα24Jα18+	singlet, TRAV10, TRAJ18 + additional V/J chains	<1% IgG, <20 HC umi		n = 93

**NK**	singlet, NCAM1+	<50 TCRA & TCRB reads	<1% IgG, <20 HC umi	NCAM1+	n = 69,997

Definition of T and NK cell identities. red: identifying features, green: additional criteria, blue: confirmatory observations.

B cell and plasmablast sub-population phenotypes

Population	Subset	ADT phenotype	TCR phenotype	BCR phenotype	GEX phenotype	Cell count
**B**		singlet, CD19+	<50 TCRA & TCRB reads	singlet	CD19+	n = 43,043
	B.NAIVE.1	IgD+ IgM+ CD27-		IGHD, IGHM (<2 mutations)	IGHD+ IGHM+ TCL1A+	n = 16,032
	B.NAIVE.2	IgD+ IgM+ CD27-			IGHD+ IGHM+ TCL1A+	n = 2,547
	B.NAIVE.3	IgDint IgM+ CD27-		IGHD, IGHM (<2 mutations)	IGHD+ IGHM+ TCL1A+	n = 531
	B.NAIVE.IgDlo	IgDlow IgMlow CD27-		IGHD, IGHM (<2 mutations)	IGHD+ IGHM+ TCL1A+	n = 71
	B.NAIVE.IFN.resp	IgD+ IgM+ CD27-		IGHD, IGHM (<2 mutations)	IGHD+ IGHM+ TCL1A+; IFN resp gene +ve (e.g. MX1+)	n = 1,176
	B.NAIVE.CD1c	IgD+ IgM+ CD27- CD1c+		IGHD, IGHM (<2 mutations), or none	IGHD+ IGHM+ TCL1A+	n = 922
	B.int.1.IFN.resp	IgD+ IgM+ CD27int			IFN resp gene +ve (e.g. MX1+)	n = 199
	B.int.1.early.act	IgD+ IgM+ CD27int		IGHD, IGHM (≥2 mutations)	IGHD+ IGHM+ TCL1Aint	n = 388
	B.int.1.early.act/sw.1	IgDlow IgM+ CD27int		IGHD, IGHM (≥2 mutations), or IGHA, IGHE, IGHG	IGHDlow IGHM+ TCL1Aint	n = 433
	B.int.1.early.act/sw.2	IgD- IgM-CD27int		IGHD, IGHM (≥2 mutations), or IGHA, IGHE, IGHG	IGHD- IGHMlow TCL1A-	n = 168
	B.int.2.unsw	IgD+ IgM+ CD27int		IGHD, IGHM (<2 mutations)	IGHD+ IGHM+ TCL1Aint	n = 835
	B.int.2.IFN.resp	IgDlow IgM+ CD27int			IGHDlow IGHM+ TCL1Aint; IFN resp gene +ve (e.g. MX1+)	n = 152
	B.int.2.early.act.IFN. resp	IgD+ IgM+ CD27int		IGHD, IGHM (≥2 mutations)	IGHD+ IGHM+ TCL1Aint; IFN resp gene +ve (e.g. MX1+)	n = 68
	B.int.2.early.act/sw.1	IgDlow IgM+ CD27int		IGHD, IGHM (≥2 mutations), or IGHA, IGHE, IGHG	IGHDlow IGHM+ TCL1Aint	n = 3,978
	B.int.2.early.act/sw.2	IgD+ IgM+ CD27int		IGHD, IGHM (≥2 mutations), or IGHA, IGHE, IGHG	IGHDlow IGHM+ TCL1Aint	n = 1,016
	B.TRANSIT.CD10	IgD+ IgM+ CD27- CD10+			IGHD+ IGHM+ TCL1A+	n = 1,116
	B.mitohi.1				>4% mito	n = 1,632
	B.mitohi.2				>4% mito	n = 470
	B.cyc				MKI67+	n = 744
	B.UNSW.MEM	IgD+ IgM+ CD27+		IGHD, IGHM	IGHD+ IGHM+ TCL1Aint	n = 2,597
	B.SW.MEM.1	IgD- IgM- CD27+ IgA+ or IgG+		IGHA, IGHE, IGHG	IGHD- IGHMlow TCL1A-	n = 5,855
	B.SW.MEM.2	IgD- IgM- CD27+ IgA+ or IgG+			IGHD- IGHMlow TCL1A-	n = 1,970
	B.SW.MEM.IFN.resp	IgD- IgM- CD27+ IgA+ or IgG+		IGHA, IGHE, IGHG or none	IGHD- IGHMlow TCL1A- ; IFN resp gene +ve (e.g. MX1+)	n = 93
**Plasma-blasts**		singlet, CD19low CD20- CD38hi CD39hi	<50 TCRA & TCRB reads	singlet	XBP1+ SLAMF7+ PRDM1+	n = 8,596
	PB			>100 HC UMI		n = 5,948
	PB.IFN.resp			>100 HC UMI	IFN resp gene +ve (e.g. MX1hi)	n = 38
	PB.mitohi			>100 HC UMI	>4% mito	n = 36
	PB.cyc			>100 HC UMI	MKI67hi	n = 2,574

Definition of B and plasmablast subsets. red: identifying features, green: additional criteria, blue: confirmatory observations (thresholds checked but not applied).
