# Supplementary material for: A blood atlas of COVID-19 defines hallmarks of disease severity and specificity
Source: Cell. 2022 Mar 3;185(5):916–938.e58. doi: 10.1016/j.cell.2022.01.012 (PMC8776501; doi:10.1016/j.cell.2022.01.012)
Supplement: Data S1: COMBAT Consortium author details and contributions [file mmc5.pdf]

Data S1: COMBAT Consortium Author Details and Contributions

Table of Contents

COMBAT Consortium members and affiliations *2*

Affiliations 2

COMBAT Consortium author contributions *4*

1. Patient recruitment and cohorts 4
2. Sample processing and extraction 4
3. Clinical phenotyping 5
4. Mass and flow cytometry 5
5. Whole blood total RNA-seq 6
6. 10X CITE-seq and repertoire 7
7. ATAC-seq 9
8. Genetics 9
9. Proteomics (mass spectrometry) 9
10. Proteomics (Luminex) 10
11. Data integration 10
12. Data management 11
13. Oversight and writing 12

Consortium team membership, contributions (CRediT taxonomy), ORCID, funding, conflict of interest and acknowledgements *14*

COMBAT Consortium members and affiliations

David J Ahern (2), Zhichao Ai (2), Mark Ainsworth (19), Chris Allan (1), Alice Allcock (1), Brian Angus (19), M Azim Ansari (1,6), Carolina V Arancibia-Cárcamo (16, 18), Dominik Aschenbrenner (16), Moustafa Attar (1,2), J Kenneth Baillie (28), Eleanor Barnes (6,16,18,19), Rachael Bashford-Rogers (1), Archana Bashyal (19), Sally Beer (39), Georgina Berridge (5), Amy Beveridge (8), Sagida Bibi (8), Tihana Bicanic (33), Luke Blackwell (8), Paul Bowness (13,18), Andrew Brent (19,38), Andrew Brown (1), John Broxholme (1), David Buck (1), Katie L Burnham (27), Helen Byrne (7), Susana Camara (8), Ivan Candido Ferreira (3), Philip Charles (4,5), Wentao Chen (20), Yi-Ling Chen (3), Amanda Chong (1), Elizabeth A Clutterbuck (8), Mark Coles (2), Christopher P Conlon (17,19,31), Richard Cornall (25), Adam P Cribbs (13), Fabiola Curion (1), Emma E Davenport (27), Neil Davidson (19), Simon Davis (5), Calliope A Dendrou (1), Julie Dequaire (19), Lea Dib (2), James Docker (1), Christina Dold (8), Tao Dong (17,25), Damien Downes (3), Hal Drakesmith (3), Susanna J Dunachie (6,19,25,31), David A Duncan (3,21), Chris Eijsbouts (1,4), Robert Esnouf (1,4), Alexis Espinosa (39), Rachel Etherington (3,25), Benjamin Fairfax (3,10,18,19), Rory Fairhead (19), Hai Fang (1), Shayan Fassih (19), Sally Felle (8), Maria Fernandez Mendoza (39), Ricardo Ferreira (1), Roman Fischer (5,17), Thomas Foord (19), Aden Forrow (7), John Frater (6,19), Anastasia Fries (19,20), Veronica Gallardo Sanchez (19), Lucy C Garner (3,16), Clementine Geeves (1), Dominique Georgiou (39), Leila Godfrey (1), Tanya Golubchik (1,4), Maria Gomez Vazquez (2), Angie Green (1), Hong Harper (1), Heather A Harrington (1,7), Raphael Heilig (5), Svenja Hester (5), Jennifer Hill (8), Charles Hinds (34), Clare Hird (19), Ling-Pei Ho (20,25), Renee Hoekzema (7), Benjamin Hollis (12), Jim Hughes (3,26), Paula Hutton (19), Matthew A Jackson-Wood (2), Ashwin Jainarayanan (2), Anna James-Bott (13), Kathrin Jansen (2), Katie Jeffery (19), Elizabeth Jones (8), Luke Jostins (2,4), Georgina Kerr (36), David Kim (19), Paul Klenerman (6,16), Julian C Knight (1,17,18,19), Vinod Kumar (2), Piyush Kumar Sharma (3,10), Prathiba Kurupati (3,25), Andrew Kwok (1), Angela Lee (1), Aline Linder (8), Teresa Lockett (19), Lorne Lonie (1), Maria Lopopolo (1), Martyna Lukoseviciute (3), Jian Luo (20), Spyridoula Marinou (8), Brian Marsden (2,22), Jose Martinez (39), Philippa C Matthews (6), Michalina Mazurczyk (3,25), Simon McGowan (3), Stuart McKechnie (19), Adam Mead (3,18,19), Alexander J Mentzer (1,19), Yuxin Mi (1), Claudia Monaco (2), Ruddy Montadon (1), Giorgio Napolitani (3,25), Isar Nassiri (3,10), Alex Novak (39), Darragh P O'Brien (5), Daniel O'Connor (8,18), Denise O'Donnell (19), Graham Ogg (3,18,25), Lauren Overend (1), Inhye Park (2), Ian Pavord (20), Yanchun Peng (3,17,25), Frank Penkava (13), Mariana Pereira Pinho (3,25), Elena Perez (39), Andrew J Pollard (8,18), Fiona Powrie (2), Bethan Psaila (3,18), T Phuong Quan (32), Emmanouela Repapi (3,26), Santiago Revale (1), Laura Silva-Reyes (8), Jean-Baptiste Richard (2,3), Charlotte Rich-Griffin (1), Thomas Ritter (19), Christine S Rollier (8), Matthew Rowland (19), Fabian Ruehle (11), Mariolina Salio (3,25), Stephen Nicholas Sansom (2), Raphael Sanches Peres (2), Alberto Santos Delgado (4,23,24), Tatjana Sauka-Spengler (3), Ron Schwessinger (3), Giuseppe Scozzafava (1), Gavin Screaton (1,36), Anna Seigal (7), Malcolm G Semple (29), Martin Sergeant (3), Christina Simoglou Karali (3), David Sims (3), Donal Skelly (6,14,19), Hubert Slawinski (1), Alberto Sobrinodiaz (39), Nikolaos Sousos (3,18,19), Lizzie Stafford (19), Lisa Stockdale (8), Marie Strickland (3), Otto Sumray (7,37), Bo Sun (1), Chelsea Taylor (3,10), Stephen Taylor (3), Adan Taylor (19), Supat Thongjuea (3), Hannah Thraves (39), John A Todd (1), Adriana Tomic (8), Orion Tong (3,10), Amy Trebes (1), Dominik Trzupek (1), Felicia Anna Tucci (1), Lance Turtle (29,30), Irina Udalova (2), Holm Uhlig (16,19), Erinke van Grinsven (2), Iolanda Vendrell (5), Marije Verheul (8), Alexandru Voda (2), Guanlin Wang (3), Lihui Wang (2), Dapeng Wang (1), Peter Watkinson (14,19), Robert Watson (3,10,19), Michael Weinberger (3,35), Justin Whalley (1), Lorna Witty (1), Katherine Wray (3,19), Luzheng Xue (20), Hing Yuen Yeung (1), Zixi Yin (17,25), Rebecca K Young (19), Jonathan Youngs (33), Ping Zhang (1), Yasemin-Xiomara Zurke (2), ISARIC4C Consortium (40).

Affiliations

1: Wellcome Centre for Human Genetics, Nuffield Department of Medicine, University of Oxford, UK
2: Kennedy Institute for Rheumatology, Nuffield Department of Orthopaedics, Rheumatology and Musculoskeletal Diseases, University of Oxford, UK
3: MRC Weatherall Institute of Molecular Medicine (WIMM), University of Oxford, UK
4: Big Data Institute, Nuffield Department of Medicine, University of Oxford, UK
5: Target Discovery Institute, Nuffield Department of Medicine, University of Oxford, UK
6: Peter Medawar Building for Pathogen Research, Nuffield Department of Medicine, University of Oxford, UK
7: Mathematical Institute, University of Oxford, UK
8: Oxford Vaccine Group, Department of Paediatrics, University of Oxford, UK
9: Department of Paediatrics, University of Oxford, UK
10: Department of Oncology, University of Oxford, UK
11: Department of Physics, University of Oxford, UK
12: Jenner Institute, Nuffield Department of Medicine, University of Oxford, UK
13: Botnar Research Centre, Nuffield Department of Orthopaedics, Rheumatology and Musculoskeletal Diseases, University of Oxford, UK
14: Nuffield Department of Clinical Neuroscience, University of Oxford, UK
15: Department of Statistics, University of Oxford, UK
16: Translational Gastroenterology Unit, University of Oxford, UK
17: Chinese Academy of Medical Science Oxford Institute (COI), University of Oxford, UK
18: NIHR Oxford Biomedical Research Centre, Oxford, UK
19: Oxford University Hospitals NHS Foundation Trust, Oxford, UK
20: Respiratory Medicine Unit, Nuffield Department of Medicine, University of Oxford, UK
21: Diamond Light Source, Didcot, UK
22: Centre for Medicines Discovery, Nuffield Department of Medicine, University of Oxford, UK
23: Center for Health Data Science, University of Copenhagen, DK
24: Novo Nordisk Foundation Center for Protein Research, University of Copenhagen, DK
25: MRC Human Immunology Unit, Radcliffe Department of Medicine, University of Oxford, UK
26: MRC WIMM Centre for Computational Biology, Radcliffe Department of Medicine, University of Oxford, UK
27: Wellcome Sanger Institute, Cambridge, UK
28: Genetics and Genomics, Roslin Institute, University of Edinburgh, Easter Bush, Midlothian, UK
29: NIHR Health Protection Research Unit in Emerging and Zoonotic Infections, Institute of Infection, Veterinary and Ecological Sciences, University of Liverpool, Liverpool
30: Tropical and Infectious Diseases Unit, Liverpool University Hospitals NHS Foundation Trust, Liverpool, UK
31: Centre for Tropical Medicine and Global Health, Nuffield Department of Medicine, University of Oxford, UK
32: Experimental Medicine Division, Nuffield Department of Medicine, University of Oxford, UK
33: Institute for Infection and Immunity, St George's University of London, UK
34: The William Harvey Research Institute, Bart’s and The London, Queen Mary University of London, UK
35: Department of Physiology, Anatomy and Genetics, University of Oxford, UK
36: Medical Sciences Division, University of Oxford, UK
37: Ludwig Institute for Cancer Research, Nuffield Department of Medicine, University of Oxford, UK
38: Nuffield Department of Medicine, University of Oxford, UK
39: Emergency Medicine Research Oxford, Oxford University Hospitals NHS Foundation Trust, Oxford, UK
40: https://isaric4c.net/about/authors/

COMBAT Consortium author contributions

1. Patient recruitment and cohorts

Alexander J Mentzer, Mark Ainsworth, Alice Allcock, Carolina V Arancibia-Cárcamo, J Kenneth Baillie, Brian Angus, Archana Bashyal, Sally Beer, Tihana Bicanic, Andrew Brent, Andrew Brown, Christopher P Conlon, Neil Davidson, Julie Dequaire, Susanna J Dunachie, Alexis Espinosa, Rory Fairhead, Shayan Fassih, John Frater, Maria Fernandez Mendoza, Thomas Foord, Anastasia Fries, Veronica Gallardo Sanchez, Dominique Georgiou, Charles Hinds, Clare Hird, Paula Hutton, Katie Jeffery, David Kim, Paul Klenerman, Julian C Knight, Andrew Kwok, Teresa Lockett, Jose Martinez, Philippa C Matthews, Stuart McKechnie, Denise O'Donnell, Alex Novak, Ian Pavord, Elena Perez, Thomas Ritter, Matthew Rowland, Malcolm G Semple, Donal Skelly, Alberto Sobrinodiaz, Lizzie Stafford, Adan Taylor, Hannah Thraves, Lance Turtle, Holm Uhlig, Peter Watkinson, Rebecca K Young, Jonathan Youngs.

<u>COVID-19 patients (Oxford)</u>
Patient recruitment, consent and phenotyping by Alexander J Mentzer, Mark Ainsworth, Brian Angus, Archana Bashyal, Sally Beer, Neil Davidson, Julie Dequaire, Alexis Espinosa, Rory Fairhead, Shayan Fassih, Maria Fernandez Mendoza, Thomas Foord, Anastasia Fries, Veronica Gallardo Sanchez, Dominique Georgiou, Clare Hird, Paula Hutton, David Kim, Andrew Kwok, Teresa Lockett, Jose Martinez, Elena Perez, Thomas Ritter, Alberto Sobrinodiaz, Adan Taylor, Hannah Thraves, Peter Watkinson, Rebecca K Young. Patients were recruited as part of Sepsis Immunomics (Julian C Knight, Chief Investigator for Sepsis Immunomics; ISARIC/WHO Clinical Characterisation Protocol for Severe Emerging Infections (J Kenneth Baillie, Paul Klenerman, Malcolm G Semple, Lance Turtle); OPTIC study (Carolina V Arancibia-Cárcamo, Christopher P Conlon, Susanna J Dunachie, John Frater, Katie Jeffery, Philippa C Matthews, Denise O'Donnell, Donal Skelly, Lizzie Stafford).

<u>Influenza patients and COVID-19 patients (non-Oxford)</u>
Patients were recruited by Jonathan Youngs, members of the AspiFlu team and clinical research nurses. Supervised by Tihana Bicanic (Chief Investigator for AspiFlu).

<u>Sepsis patients</u>
Sepsis patients were recruited by Andrew Kwok, Sally Beer and the clinical research nurse team at Oxford University Hospitals Trust, with supervision and input by Julian C Knight (Chief Investigator for Sepsis Immunomics), Stuart McKechnie (local PI), Charles Hinds, Alexander J Mentzer, Andrew Brent, Alex Novak and Matthew Rowland.

<u>Healthy volunteers</u>
Healthy volunteers were recruited and sampled by Andrew Kwok with support from Alexander J Mentzer, Andrew Brown and Alice Allcock. Supervised by Julian C Knight as Chief Investigator (healthy volunteer study).

<u>Supervision of recruitment and cohorts</u>
Overall supervision and direction by Alexander J Mentzer, with input from Julian C Knight and Andrew Kwok.

2. Sample processing and extraction

Alexander J Mentzer, Alice Allcock, Chris Allan, Amy Beveridge, Sagida Bibi, Tihana Bicanic, Luke Blackwell, Andrew Brown, David Buck, Susana Camara, Elizabeth Clutterbuck, Fabiola Curion, Christina Dold, Tao Dong, Hal Drakesmith, Sally Felle, Leila Godfrey, Angie Green, Jennifer Hill, Elizabeth Jones, Julian C Knight, Andrew Kwok, Angela Lee, Aline Linder, Lorne Lonie, Maria Lopopolo, Spyridoula Marinou, Adam Mead,

Yanchun Peng, Andrew J Pollard, Laura Silva-Reyes, Christina Simoglou Karali, Christine S Rollier, Giuseppe Scozzafava, Hubert Slawinski, Lisa Stockdale, Amy Trebes, Marije Verheul, Lorna Witty Katherine Wray, Zixi Yin, Jonathan Youngs.

Sample processing
Preparation of samples, separation and cryopreservation of peripheral blood mononuclear cells Chris Allan, Amy Beveridge, Sagida Bibi, Luke Blackwell, Susana Camara, Elizabeth Clutterbuck, Christina Dold, Sally Felle, Angie Green, Jennifer Hill, Elizabeth Jones, Andrew Kwok, Aline Linder, Spyridoula Marinou, Yanchun Peng, Laura Silva-Reyes, Christina Simoglou Karali, Christine S Rollier, Marie Strickland, Lisa Stockdale, Marije Verheul, Katherine Wray, Zixi Yin, Jonathan Youngs.

Purification of genomic DNA and RNA
Alice Allcock, Andrew Brown, Andrew Kwok, Giuseppe Scozzafava.

Sample management, curation and cataloguing
Andrew Kwok, Alexander J Mentzer, Giuseppe Scozzafava.

Oxford Genomics Centre (OGC) core sequencing facility
Fabiola Curion, Angela Lee, Maria Lopopolo, Lorne Lonie, Hubert Slawinski, Amy Trebes, Lorna Witty

Supervision
Supervised in specific labs by Tihana Bicanic (London), David Buck (OGC), Tao Dong (WIMM), Julian C Knight (WHG), Andrew J Pollard (CCVTM); and with overall direction by Alexander J Mentzer and Julian C Knight.

3. Clinical phenotyping

Alexander J Mentzer, J Kenneth Baillie, Eleanor Barnes, Tihana Bicanic, Calliope A Dendrou, Lucy C Garner, Ling-Pei Ho, Julian C Knight, Andrew Kwok, Luke Jostins, T Phuong Quan, Fabian Ruehle, Stephen N Sansom, Malcolm G Semple, Alberto Santos Delgado, Lance Turtle, Jonathan Youngs.

4. Mass and flow cytometry

Giorgio Napolitani, Mariolina Salio, Claudia Monaco, Irina Udalova, David J Ahern, Yasemin- Xiomara Zurke, Zhichao Ai, Lea Dib, Rachel Etherington, Julian C Knight, Prathiba Kurupati, Andrew Kwok, Michalina Mazurczyk, Graham Ogg, Inhye Park, Mariana Periera Pinho, Emmanouela Repapi, Dominik Trzupek, Erinke van Grinsven, Lihui Wang.

Mass Cytometry - Whole Blood (figs 1, 3, 4, 5)

Experimental design and data generation
Andrew Kwok developed the antibody panel with help from Rachel Etherington and Michalina Mazurczyk and input from Giorgio Napolitani. Andrew Kwok processed the samples and performed the staining with help from Giorgio Napolitani. Rachel Etherington and Giorgio Napolitani acquired the CyTOF samples on the Helios Mass Cytometer.

Data analysis
Giorgio Napolitani performed manual de-barcoding and initial processing. Emmanouela Repapi performed processing and normalization of the data. Mariana Pereira Pino, and Emmanouela Repapi performed overall analysis with inputs from Giorgio Napolitani and Andrew Kwok. Mariana Pereira Pino performed cluster annotation and manual gating

analysis with inputs from Giorgio Napolitani. Emmanouela Repapi performed the statistical analysis.

Data integration:
Emmanouela Repapi performed the CITE-seq Mass Cytometry data integration.

Supervision
Giorgio Napolitani supervised the experimental design, data generation and data analysis with inputs from Julian C Knight.

Mass Cytometry - Myeloid panel (fig 2)

Experimental design and data generation
David J Ahern developed the myeloid CyTOF panel with input from Claudia Monaco, Irina Udalova, Lea Dib, Nikolaos Sousos and Lihui Wang. David J Ahern, Lea Dib and Inhye Park performed the staining.

Data analysis
Yasemin-Xiomara Zurke and David J Ahern performed the CyTOF analysis and clustering.

Supervision
Claudia Monaco and Irina Udalova supervised the data analysis and performed the interpretation of the data.

Multicolor Flow Cytometry

Data generation
Mariolina Salio developed and tested the flow cytometry panels. Mariolina Salio processed, stained and acquired the samples for Multicolor Flow Cytometry and did the data acquisition with help from Giorgio Napolitani.

Flow Cytometry Data Analysis
Mariana Pereira Pinho and Mariolina Salio performed data analysis with inputs from Giorgio Napolitani.

Supervision
Mariolina Salio supervised the experimental design, data generation and data analysis.

5. Whole blood total RNA-seq

Alexander J Mentzer, Moustafa Attar, Katie L Burnham, Emma E Davenport, James Docker, Clementine Geeves, Charles Hinds, Julian C Knight, Andrew Kwok, Angela Lee, Daniel O'Connor, Santiago Revale, Justin Whalley.

Data generation
Data was generated by Andrew Kwok, Moustafa Attar, James Docker, Clementine Geeves.

Data analysis
Data analysis was performed by Katie L Burnham, Andrew Kwok, Daniel O'Connor (WGCNA), Santiago Revale, Justin Whalley.

Supervision
Supervised by Alexander J Mentzer and Julian C Knight with Emma E Davenport and Charles Hinds.

6. 10X CITE-seq and repertoire

Stephen N Sansom, Calliope A Dendrou, Luke Jostins, Julian C Knight, Rachael Bashford-Rogers, Benjamin Fairfax, Dominik Aschenbrenner, Moustafa Attar, Paul Bowness, Adam P Cribbs, Fabiola Curion, Tao Dong, Ricardo Ferreira, Lucy C Garner, Maria Gomez Vazquez, Anna James-Bott, Ashwin Jainarayanan, Kathrin Jansen, Paul Klenerman, Piyush Kumar Sharma, Andrew Kwok, Angela Lee, Adam Mead, Alexander J Mentzer, Ruddy Montadon, Giorgio Napolitani, Isar Nassiri, Lauren Overend, Yanchun Peng, Frank Penkava, Bethan Psaila, Emmanouela Repapi, Santiago Revale, Jean-Baptiste Richard, Charlotte Rich-Griffin, Hubert Slawinski, Bo Sun, Chelsea Taylor, Supat Thongjuea, Orion Tong, Felicia A Tucci, Alexandru Voda, Guanlin Wang, Robert Watson, Hing Yuen Yeung, Yasemin-Xiomara Zurke.

Experimental design and data generation

Fabiola Curion, Julian C Knight, Andrew Kwok, Luke Jostins, Alexander J Mentzer, Rachael Bashford-Rogers and Stephen N Sansom contributed to sample selection and experimental design. Sample preparation, cell QC and single-cell capture was performed by Andrew Kwok, Hubert Slawinski and Moustafa Attar.

Analysis of the gene expression (GEX) data

Quantitation and genetic demultiplexing was performed by Fabiola Curion and Santiago Revale. Kathrin Jansen computed velocity matrices. Pre-processing and QC assessment of the GEX data was performed by Bo Sun, Fabiola Curion, Kathrin Jansen and Maria Gomez Vasquez with input from and Lauren Overend. Databasing of cell metrics was performed by Adam P Cribbs with help from Stephen N Sansom. Extraction of matrices for downstream analysis was performed by Stephen N Sansom. Alignment of the data was performed by Kathrin Jansen with advice from Supat Thongjuea and Stephen N Sansom. Clustering, marker and pathway analysis was performed by Stephen N Sansom. Initial clustering results were independently confirmed by Charlotte Rich-Griffin and Calliope A Dendrou. Analysis of viral transcripts was performed by Adam P Cribbs, Anna James-Bott and Lauren Overend.

Analysis of the antibody-derived tag (ADT) data

Analysis of the ADT data was performed by Fabiola Curion and Giorgio Napolitani with help from Emmanouela Repapi and input from Calliope A Dendrou and Stephen N Sansom.

Analysis of the CITE-seq and bulk BCR and TCR data

Santiago Revale performed CITE-seq VDJ pre-processing. Bo Sun performed single cell BCR repertoire analysis. Single cell TCR repertoire analysis with exploration of clonal size, integration with gene expression and Kmer usage was performed by Rob Watson, Orion Tong, Chelsea Taylor and Piyush Kumar Sharma. Felicia A Tucci performed the bulk BCR and TCR repertoire amplification, library preparation and sample handling. Rachael Bashford- Rogers performed the bulk BCR and TCR repertoire analysis. Repertoire team members who have provided guidance and feedback to the analyses and manuscript were Lauren Overend, Tao Dong, Charlotte Rich-Griffin, Yanchun Peng, Isar Nassiri, Paul Bowness, and Frank Penkava.

Multimodal annotation of the CITE-seq dataset

Annotation of B-cell subpopulations was performed by Bo Sun, Calliope A Dendrou and

Charlotte Rich-Griffin with input from Felicia A Tucci, Hing Yuen Yeung and Rachael Bashford-Rogers. Annotation of T/NK subpopulations was performed by Calliope A Dendrou, Lucy C Garner, Ricardo Ferreira, Giorgio Napolitani and Stephen N Sansom with input from Paul Klenerman, Andrew Kwok and Hing Yuen Yeung. Annotation of the myeloid subsets was performed by Andrew Kwok and Stephen N Sansom. Annotation of HSC and platelets was performed by Stephen N Sansom and Calliope A Dendrou with input from Bethan Psaila. Annotation of doublet populations was performed by Fabiola Curion and Kathrin Jansen. Initial marker visualization was performed by Charlotte Rich-Griffin and Fabiola Curion. Curation of annotations was performed by Stephen N Sansom with review from Andrew Kwok, Lucy C Garner and Calliope A Dendrou. Manuscript figures were prepared by Stephen N Sansom.

Composition analysis

Composition PCA and association testing was performed by Lucy C Garner and Charlotte Rich- Griffin with input from Calliope A Dendrou. Comparison of multiple differential abundance analysis methods and final analyses were performed by Charlotte Rich-Griffin and Lucy C Garner with input from Calliope A Dendrou. Clinical variable selection and covariate analysis were performed by Lucy C Garner, Charlotte Rich-Griffin and Calliope A Dendrou. Calliope A Dendrou interpreted the results and prepared final manuscript figures with input from Charlotte Rich-Griffin, Julian C Knight and Lucy C Garner.

Pseudobulk analysis

Initial pseudobulk counts were generated by Stephen N Sansom and assessed by Fabiola Curion and Luke Jostins. Normalized pseudobulk counts, principal components and UMAP co-ordinates were generated by Luke Jostins with advice from Stephen N Sansom and Fabiola Curion. Alexandru Voda performed differential expression analyses with input from Luke Jostins and Stephen N Sansom. Luke Jostins collected pathway gene lists with input from Lucy C Garner, Adam P Cribbs, Andrew Kwok, Jean-Baptiste Richard and Dominik Aschenbrenner, and carried out gene set enrichment analyses. Clinical covariate analysis was carried out by Luke Jostins with input from Lucy C Garner, Charlotte Rich-Griffin and Calliope A Dendrou. Luke Jostins interpreted results and generated manuscript figures with input from Stephen N Sansom, Calliope A Dendrou and JulianC Knight.

WGCNA and network analysis

Lucy C Garner performed initial exploratory analyses. WGCNA network analysis was performed by Charlotte Rich-Griffin, Fabiola Curion and Stephen N Sansom. Charlotte Rich-Griffin and Fabiola Curion identified sets of modules common to multiple cell types and characterized their relationship with the type I IFN signaling response. Stephen N Sansom tested module associations, performed additional analyses and interpreted the results with input from Calliope A Dendrou, Charlotte Rich-Griffin, Fabiola Curion, Andrew Kwok and Julian C Knight. Charlotte Rich-Griffin, Fabiola Curion and Stephen N Sansom prepared the figures.

Cell-cell interaction analysis

Cell-cell interaction was performed by Guanlin Wang with input from Supat Thongjuea.

Supervision

Luke Jostins, Julian C Knight, Rachael Bashford-Rogers and Stephen N Sansom supervised the experimental design. Rachael Bashford-Rogers supervised the BCR and repertoire analysis. Benjamin Fairfax supervised the TCR analysis. Luke Jostins supervised

the pseudobulk analysis and provided expert statistical guidance. Calliope A Dendrou supervised the composition analysis and co-supervised the multimodal annotation. Supat Thongjuea co- supervised the alignment work. Supat Thongjuea and Adam Mead supervised the cell-cell interaction analysis. Stephen N Sansom supervised the GEX pre-processing, alignment and clustering, the multimodal annotation and the WGCNA network analysis. Overall supervision and facilitation of work by Stephen N Sansom with Calliope A Dendrou (CITE-seq) and Rachael Bashford-Rogers (repertoire).

7. ATAC-seq

Tatjana Sauka-Spengler, Ivan Candido Ferreira, Martyna Lukoseviciute, Moustafa Attar, Santiago Revale, Julian C Knight, Andrew Kwok, Angela Lee, Hubert Slawinski, Michael Weinberger.

Data generation
Data was generated by Andrew Kwok, Moustafa Attar and Hubert Slawinski.

Data analysis
Data analysis was performed by Ivan Candido Ferreira, Martyna Lukoseviciute, Santiago Revale, Andrew Kwok, Tatjana Sauka-Spengler and Michael Weinberger.

Supervision
Tatjana Sauka-Spengler and Julian C Knight.

8. Genetics

Luke Jostins, Amanda Chong, Calliope A Dendrou, Damien Downes, Chris Eijsbouts, Ben Fairfax, Jim Hughes, Julian C Knight, Alexander J Mentzer, Yuxin Mi, Isar Nassiri, Stephen N Sansom, Ron Schwessinger, Ping Zhang.

Alexander J Mentzer and Andrew Kwok prepared samples for genotyping, Ben Hollis and Luke Jostins carried out quality control, principal component analysis and imputation. Amanda Chong carried out association analysis. Damien Downes, Chris Eijsbouts, Luke Jostins, Yuxin Mi, Isar Nassiri, Ron Schwessinger and Ping Zhang carried out integration with other data modalities. Calliope A Dendrou, Ben Fairfax, Jim Hughes, Luke Jostins, Julian C Knight, Alexander J Mentzer and Stephen N Sansom supervised specific analyses, and Luke Jostins provided overall supervision.

9. Proteomics (mass spectrometry)

Roman Fischer, Alberto Santos Delgado, Georgina Berridge, Philip Charles, Simon Davis, Raphael Heilig, Svenja Hester, Julian C Knight, Yuxin Mi, Darragh P O'Brien, Iolanda Vendrell.

Experimental design
Iolanda Vendrell, Georgina Berridge and Roman Fischer developed the experimental design with inputs from Philip Charles, Simon Davis, Raphael Heilig.

Data generation
Georgina Berridge, Iolanda Vendrell, Svenja Hester, Darragh P O’Brien and Roman Fischer processed samples. Data was generated by Georgina Berridge, Iolanda Vendrell, Simon Davis and Raphael Heilig.

Data analysis

Roman Fischer and Philip Charles performed identification and quantitation. Yuxin Mi and Alberto Santos Delgado performed downstream analysis and interpretation.

Supervision
Iolanda Vendrell supervised the sample processing, Roman Fischer supervised the team.

Acknowledgements
The Proteomics team is grateful for the help of Zuzana Bencokova, Darren Blase and Benedikt Kessler (all TDI) for providing the environment for safe sample handling and analysis. We also thank Alexey Nesvizhskii and Fengchao Yu (both Department of Pathology. University of Michigan Medical School) for providing help and advice for data processing in Fragpipe.

10. Proteomics (Luminex)

Luzheng Xue, Wentao Chen, Yi-Ling Chen, David A Duncan, Paul Klenerman, Jian Luo, Claudia Monaco, Graham Ogg, Ian Pavord, Irina Udalova.

Experimental design
Wentao Chen and Luzheng Xue designed the Luminex assay panels with inputs from Claudia Monaco, Irina Udalova, Ian Pavord, Graham Ogg and Paul Klenerman.

Data generation
Yi-Ling Chen and Jian Luo processed the samples, performed the assays and generated the data.

Data analysis
Yi-Ling Chen, Jian Luo, Wentao Chen and David A Duncan performed data analysis.

Supervision
Luzheng Xue supervised the experimental design, data generation and data analysis.

11. Data integration

Julian C Knight, M Azim Ansari, Rachael Bashford-Rogers, Katie L Burnham, Helen Byrne, Mark Coles, Fabiola Curion, Calliope A Dendrou, Aden Forrow, Roman Fischer, Tanya Golubchik, Heather A Harrington, Ling-Pei Ho, Renee Hoekzema, Jim Hughes, Matthew A Jackson-Wood, Luke Jostins, Simon McGowan, Alexander J Mentzer, Yuxin Mi, Giorgio Napolitani, Emmanouela Repapi, Fabian Ruehle, Raphael Sanches Peres, Alberto Santos Delgado, Stephen N Sansom, Martin Sergeant, Anna Seigal, David Sims, Otto Sumray, Stephen Taylor, Adriana Tomic, Justin Whalley.

Clinical phenotyping
Statistical and machine learning analysis by Fabian Ruehle with input and interpretation from Ling-Pei Ho, Julian C Knight, Alexander J Mentzer and Stephen N Sansom.

Integrative analysis of mass cytometery and CITEseq cell composition
Analysis by Emmanouela Repapi and Yasemin-Xiomara Zurke with input from Fabiola Curion, Calliope A Dendrou, Giorgio Napolitani and Stephen N Sansom.

Similarity network fusion and patient subphenotyping
Analysis by Alberto Santos Delgado with input and interpretation from Katie L Burnham, Roman Fischer, Yuxin Mi, Julian C Knight.

Machine learning

Fabian Ruehle and Matthew A Jackson-Wood undertook machine learning analysis of phenotyping and experimental datasets with input from Ling-Pei Ho, Stephen N Sansom, Luke Jostins and Julian C Knight. Adriana Tomic performed analysis using Sequential Iterative Modeling "Over Night" (SIMON).

Topological data analysis
Otto Sumray and Renee Hoekzema performed the analysis with supervision by Heather A Harrington and input from Ling-Pei Ho and Stephen N Sansom.

Tensor and matrix decomposition
Data was analysed and interpreted by Justin Whalley, Heather A Harrington, Julian C Knight, Anna Seigal and Stephen N Sansom.

Data mining
Datasets identified and curated by Calliope A Dendrou with input from Guanlin Wang and Charlotte Rich-Griffin.

Data visualization
Martin Sergeant, Simon McGowan and Stephen Taylor developed software for data visualization and hosting through Multi Locus View (MLV), with input from Jim Hughes and Stephen N Sansom. Hallmark collation Katie L Burnham, Julian C Knight and Yuxin Mi with graphical visualization (Julian C Knight) and interactive visualization in MLV (Martin Sergeant, Stephen Taylor) with input from Stephen N Sansom. COMBAT logo and manuscript artwork Julian C Knight.

Supervision
Julian C Knight led and facilitated work by the group; Stephen N Sansom helped supervise the work.

12. Data management

Brian Marsden, John Broxholme, Adam P Cribbs, Robert Esnouf, Hai Fang, Hong Harper, Luke Jostins, Julian C Knight, Georgina Kerr, Paul Klenerman, Vinod Kumar, Alexander J Mentzer, Fiona Powrie, Stephen N Sansom, David Sims, John A Todd, Dapeng Wang.

Data Management
Hai Fang and Dapeng Wang managed data set registration and curation. Brian Marsden, Vinod Kumar and Hong Harper developed and implemented data management solutions. Alexander J Mentzer, Brian Marsden, Julian C Knight, Vinod Kumar, Georgina Kerr and Giuseppe Scozzafava implemented and managed processes for sample tracking and management. Brian Marsden and Luke Jostins designed the protocol for capture of project dataset metadata.

Digital Communications
Brian Marsden, Julian C Knight, Georgina Kerr, Stephen N Sansom and Dapeng Wang set up and managed communication platforms, website and channels for the consortium.

High-performance computing environment and Code management
A bespoke high-performance computational platform was provided by the University of Oxford's Biomedical Research Computing facility under the guidance of Robert Esnouf. Stephen N Sansom, Brian Marsden and Hai Fang helped configure and manage use of the platform with input from Adam P Cribbs. Stephen N Sansom documented use of the platform, configured the COMBATOxford Team Github and defined coding practice and management policies with input from Adam P Cribbs and David Sims.

Data management operational group
Brian Marsden, Hai Fang, Hong Harper, Julian C Knight, Vinod Kumar, Alexander J Mentzer, Dapeng Wang.

Data Access Committee
Julian C Knight, Paul Klenerman, Brian Marsden, Alexander J Mentzer, Fiona Powrie, John A Todd.

Supervision
Brian Marsden supervised the overall data management strategy and implementation with Julian C Knight. Brian Marsden, Stephen N Sansom and Georgina Kerr supervised development and use of the computational platform. Stephen N Sansom and Adam P Cribbs supervised management of code. Alexander J Mentzer supervised sample tracking and management.

13. Oversight and writing

Julian C Knight, Rachael Bashford-Rogers, Katie L Burnham, Richard Cornall, Fabiola Curion, Calliope A Dendrou, Benjamin Fairfax, Hai Fang, Roman Fischer, Lucy C Garner, Heather A Harrington, Charles Hinds, Luke Jostins, Paul Klenerman, Andrew Kwok, Brian Marsden, Helen McShane, Alexander J Mentzer, Yuxin Mi, Giorgio Napolitani, Graham Ogg, Andrew J Pollard, Fiona Powrie, Charlotte Rich-Griffin, Fabian Ruehle, Stephen N Sansom, Alberto Santos Delgado, Tatjana Sauka-Spengler, Gavin Screaton, Anna Seigal, Martin Sergeant, Stephen Taylor, Bo Sun, Felicia A Tucci, John A Todd, Adriana Tomic, Justin Whalley, Luzheng Xue.

Writing group
Julian C Knight, Rachael Bashford-Rogers, Calliope A Dendrou, Ben Fairfax, Roman Fischer, Luke Jostins, Paul Klenerman, Alexander J Mentzer, Giorgio Napolitani, Stephen N Sansom, Tatjana Sauka-Spengler, John A Todd, Luzheng Xue.

Living reports contributing to manuscript
Living reports on individual assay modalities writing leads Rachael Bashford-Rogers and Ben Fairfax (B and T cell repertoire); Katie L Burnham and Alexander J Mentzer (whole blood total RNA-seq); Fabiola Curion, Charlotte Rich-Griffin, Stephen N Sansom (WGCNA scRNA-seq); Calliope A Dendrou (10X CITE-seq composition), Roman Fischer (proteomics mass spectrometry); Heather A Harrington (topological data analysis); Luke Jostins (genetics); Alexander J Mentzer (clinical phenotyping); Giorgio Napolitani (flow and mass cytometry); Fabian Ruehle (statistical analysis clinical data, machine learning); Martin Sergeant and Stephen Taylor (Multi Experiment Viewer); Stephen N Sansom (10X CITE-seq pre-processing, alignment and clustering); Adriana Tomic (Sequential Iterative Modeling “Over Night” SIMON); Justin Whalley and Stephen N Sansom (Tensor analysis); Luzheng Xue (proteomics luminex). Contributions to reports by team members detailed within teams sections.

Manuscript writing, review and editing
Original draft written by Julian C Knight. Review and editing by Rachael Bashford-Rogers, Katie Burnham, Richard Cornall, Calliope A Dendrou, Hai Fang, Ben Fairfax, Roman Fischer, Lucy C Garner, Heather A Harrington, Charles Hinds, Luke Jostins, Andrew Kwok, Paul Klenerman, Alexander J Mentzer, Yuxin Mi, Giorgio Napolitani, Fiona Powrie, Stephen N Sansom, Alberto Santos Delgado, Anna Seigal, Bo Sun, Felicia A Tucci, John A Todd, Luzheng Xue. All other Consortium authors saw, had the opportunity to comment on, and approved the final draft.

Leadership and oversight

Julian C Knight led the COMBAT consortium including the work described in this paper. Paul Klenerman led the wider Oxford COVID-19 Immunology Research programme. Stephen N Sansom helped lead data analysis and interpretation. COMBAT team leadership and oversight Rachael Bashford-Rogers, Calliope A Dendrou, Ben Fairfax, Roman Fischer, Luke Jostins, Julian C Knight, Paul Klenerman, Brian Marsden, Alexander J Mentzer, Giorgio Napolitani, Stephen N Sansom, John A Todd, Tatjana Sauka-Spengler, Luzheng Xue. Georgina Kerr provided project management.

Steering committee

Richard Cornall, Fiona Powrie, Julian C Knight, Paul Klenerman, Alexander J Mentzer, Helen McShane, Graham Ogg, Andrew J Pollard, Gavin Screaton, John A Todd.

Consortium team membership, contributions (CRediT taxonomy), ORCID, funding, conflict of interest and acknowledgements

NAME	MEMBERSHIP OF COMBAT TEAM(S)<br><br>(1) 10X CITE-seq; (2) ATAC-seq; (3) Whole blood total RNA-seq; (4) Clinical phenotyping; (5) Data integration; (6) Data management; (7) Flow and mass cytometry; (8) Genetics; (9) Serology and biochemistry; (10) Proteomics (Luminex); (11) Proteomics (mass spectrometry); (12) Repertoire; (13) Sample processing; (14) Viral sequencing; (15) Recruitment and sampling; (16) Oversight and strategic	CONTRIBUTION (CRediT taxonomy)[1]<br><br>(1) Conceptualization; (2) Methodology; (3) Software; (4) Validation; (5) Formal analysis; (6) Investigation; (7) Resources; (8) Data curation; (9) Writing-original draft; (10) Writing-review and editing; (11) Visualization; (12) Supervision; (13) Project administration; (14) Funding acquisition.	ORCID iD	FUNDING	CONFLICT OF INTEREST	ACKNOWLEDGEMENTS
David J Ahern (D.J.A.)	7	5	0000-0002-7006-7396			
Zhichao Ai (Z.A.)	7	6				
Mark Ainsworth (M.A.)	15	6,8,13				
Chris Allan (C.A.)	13	6,7		Wellcome Trust [Core Award 203141/Z/16/Z]		
Alice Allcock (A.A.)	13,15	6,7				
Brian Angus (B.A.)	13	7				
M Azim Ansari (M.A.A.)	14	3,5,7,8,10	0000-0003-2790-8353	MAA is supported by Wellcome Trust and Royal Society (220171/Z/20/Z)		
Carolina V Arancibia-Cárcamo (C.V.A-C.)	15	7,13	0000-0003-1006-3630	Oxford Biomedical Research Centre	nil	TGU Biobanking Team

Dominik Aschenbrenner (D.A.)	1, 7	5	0000-0001-7580-2567	Oxford Biomedical Research Centre		
Moustafa Attar (M.At.)	1, 2, 3	2,6,7	0000-0003-4116-1276	Kennedy Trust for Rheumatology Research KENN192004		
J Kenneth Baillie (J.K.B.)	4,6,15	6,7,8,12,13,14				
Eleanor Barnes (E.B.)	4,15	7,10,12,13,14	0000-0002-0860-0831	CIC-UK, Huo Familiy foundation, and MSD grant from OU		EB is funded NIHR as an NIHR Senior Investigator.
Rachael Bashford-Rogers (R.B-R.)	1,12,16	1,2,3,5,6,9,10,11,12,14	0000-0002-6838-0711	Wellcome Trust	R.J.M.B.-R. is a co-founder and consultant of Alchemab Therapeutics Ltd.	
Archana Bashyal (A.B.)	15	6,7				
Sally Beer (S.B.)	15	6,8,13	0000-0002-0988-3458	NIHR Clinical Research Network		
Georgina Berridge (G.B.)	11	2,4,6				
Amy Beveridge (A.Be)	13	6				
Sagida Bibi (S.Bi)	13	6	0000-0002-0855-2737			
Tihana Bicanic (T.B.)	4,13	7,10	0000-0002-2676-838X	Gilead UK & Ireland Fellowship in Invasive Fungal Disease	none	research nurses at the AspiFlu study sites who helped with flu patient enrolment

Luke Blackwell (L.B.)	13	6				
Paul Bowness (P.B.)	12	1,12	0000-0003-4597-0484	18 NIHR Oxford Biomedical Research Centre		
Andrew Brent (A.Br.)	15	7	0000-0002-5349-0723			
Andrew Brown (A.Bro)	13,15	6,7				
John Broxholme (J.B.)	3,6,8	6	0000-0001-5638-0258	Wellcome Trust [Core Award 203141/Z/16/Z]		
David Buck (D.B.)	13	7,12,14	0000-0001-9986-3874	Wellcome Trust [Core Award 203141/Z/16/Z]		
Katie L Burnham (K.L.B.)	3,5	2,5,6,7,8,11	0000-0001-8680-2933	Wellcome Trust Grant [206194]		
Helen Byrne (H.B.)	5	7	0000-0003-1771-5910	EPSRC [EP/R018472/1]		
Susana Camara (S.C.)	13	6				
Ivan Candido Ferreira (I.C.F.)	1,2	2,5	0000-0002-4916-4212			
Philip Charles (P.C.)	5,11	1,2,3,4	0000-0001-5278-5354			
Wentao Chen (W.C.)	10	2,5,11	0000-0003-1705-2299	NIHR Oxford BRC		
Yi-Ling Chen (Y-L.C.)	10	2,5,6,10,11	0000-0003-3758-0638			
Amanda Chong (A.C.)	8	5	0000-0003-4919-2632			
Elizabeth A Clutterbuck (E.A.C.)	13	6	0000-0003-1701-1390			
Mark Coles (M.C.)	5	1	0000-0001-8079-9358			

Christopher P Conlon (C.P.C.)	15	7,14	0000-0002-3531-2464	Chinese Academy of Medical Sciences (CAMS) Innovation Fund for Medical Science (CIFMS), China (grant number: 2018-I2M-2-002		
Richard Cornall (R.C.)	9,16	12			Founder of MIROBio	
Adam P Cribbs (A.P.C.)	1,5,14	2,3,4,7,8,12	0000-0001-5288-3077	MRC Career Development (MR/V010182/1)		
Fabiola Curion (F.C.)	1,5,13	1,2,3,4,5,6,7,8,9,10,11,12	0000-0003-2502-8803	Wellcome Trust [Core Award 203141/Z/16/Z]		
Emma E Davenport (E.E.D.)	3	2,12	0000-0002-8768-346X	Wellcome Trust Grant [206194]		
Neil Davidson (N.D.)	15	6,7				
Simon Davis (S.D.)	11	6	0000-0001-7840-2411			
Calliope A Dendrou (C.A.D.)	1,4,10,16	1,2,5,6,7,9,10,11,12	0000-0003-1179-4021	Wellcome Trust and Royal Society (204290/Z/16/Z) ; Rosetrees Trust (R35579/AA002/ M85-F2); NIHR Oxford BRC (BRCRCF19-04)		
Julie Dequaire (J.D.)	15	6,7				
Lea Dib (L.D.)	7	6,7	0000-0001-5889-4738			
James Docker (J.Do.)	3	6,7		Wellcome Trust [Core Award 203141/Z/16/Z]		

Christina Dold (C.D.)	13	6,12	0000-0003-2314-507X			
Tao Dong (T.D.)	13	6,7,8	0000-0003-3557-1850	Chinese Academy of Medical Sciences (CAMS) Innovation Fund for Medical Science (CIFMS), China (grant number: 2018-I2M-2-002 and MRC Unit Core		
Damien Downes (D.D.)	8	5	0000-0002-5034-0869		Nucleome Therapeutics (paid consultancy)	
Hal Drakesmith (H.D.)	9	12	0000-0002-8503-6103	MRC HIU Core grant, Haematology Theme Oxford Biomedical Research Centre		
Susanna J Dunachie (S.J.D.)	15	7,12	0000-0001-5665-6293	Supported by the UK Department of Health and Social Care as part of the PITCH (Protective Immunity from T cells to Covid-19 in Health workers) Consortium, the UK Coronavirus		

				Immunology Consortium (UK-CIC) and the Huo Family Foundation. SJD is supported by an NIHR Global Research Professorship.		
David A Duncan (D.A.D.)	10	5,11	0000-0002-0827-2022			
Chris Eijsbouts (C.E.)	8	5	0000-0001-5179-0653	Wellcome Trust Grant 215097/Z/18/Z		
Robert Esnouf (R.E.)	6	7,12	0000-0002-5425-6955			The computational aspects of this research were made possible by the Oxford Biomedical Research Computing (BMRC) platform which receives underpinning support from the Wellcome Trust Core Award Grant Number 203141/Z/16/Z and the NIHR Oxford BRC. The views expressed are those of the author(s) and not necessarily those of the NHS, the NIHR or the Department of Health
Alexis Espinosa (A.E.)	15	6,7	0000-0002-5091-6768	NIHR Clinical Research Network		
Rachel Etherington (R.Et.)	7	2, 6	0000-0002-4348-4565			

Benjamin Fairfax (B.F.)	12,16	1,2,3,5,6,9,10,11,12	0000-0001-7413-5002	201488/Z/16/Z		
Rory Fairhead (R.F.)	15	6,7				
Hai Fang (H.F.)	6	3,8,10	0000-0003-3961-8572			
Shayan Fassih (S.F.)	15	6,7	0000-0003-2968-658X			
Sally Felle (S.Fe.)	13	6				
Maria Fernandez Mendoza (M.F.M)	15	6,7		NIHR Clinical Research Network		
Ricardo Ferreira (R.F.)	1	5	0000-0001-5733-3285			
Roman Fischer (R.Fi.)	11,16	1,2,5,6,7,9,10,11,12,14	0000-0002-9715-5951	BRD00230, COI Chinese Academy of Medical Sciences (CAMS) Innovation Fund for Medical Science (CIFMS), China (grant number: 2018-I2M-2-002)	n/a	Benedikt Kessler (TDI), Zuzana Bencokova (TDI), Darren Blase (TDI), Alexey Nesvizhskii (UMICH), Fengchao Yu (UMICH)
Thomas Foord (T.F.)	15	6,7				
Aden Forrow (A.Fo.)	1	5	0000-0001-8316-5369	Royal Commission for the Exhibition of 1851 Research Fellowship		
John Frater (J.F.)	12,15	2,5,7,14				
Anastasia Fries (A.F.)	15	7	0000-0003-1609-5991	AF is an NIHR Academic Clinical Fellow		
Veronica Gallardo Sanchez (V.G.S.)	15	6,7				

Lucy C Garner (L.C.G.)	1,12	2,3,5,8,10,11	0000-0002-6461-252X	NIHR (RCF19/043); Wellcome (204826/Z/16/Z)		
Clementine Geeves (C.G.)	3	6,7	0000-0001-7394-8546	Wellcome Trust [Core Award 203141/Z/16/Z]		
Dominique Georgiou (D.G.)	15	6,7		NIHR Clinical Research Network		
Leila Godfrey (L.G.)	1	5				
Tanya Golubchik (T.G.)	14	2,3,7,8	0000-0003-2765-9828	Viral sequencing funded by the UK COVID-19 Genomics Consortium (COG-UK). COG-UK is supported by funding from the Medical Research Council (MRC) part of UK Research & Innovation (UKRI), the National Institute of Health Research (NIHR) and Genome Research Limited, operating as the Wellcome Sanger Institute. The research was supported by the Wellcome		Christophe Fraser and COG-UK

				Trust Core Award Grant Number 203141/Z/16/Z with funding from the NIHR Oxford BRC. TG was supported by COG-UK.		
Maria Gomez Vazquez (M.G.V.)	1	2,3,5,6		Kennedy Trust for Rheumatology Research KENN151612		
Angie Green (A.G.)	13	12		Wellcome Trust [Core Award 203141/Z/16/Z]		
Hong Harper (H.H.)	6	3				
Heather A Harrington (H.A.H.)	5	2,5,6	0000-0002-1705-7869	HAH gratefully acknowledges funding from EPSRC EP/R018472/1, EP/R005125/1 and EP/T001968/1, the Royal Society RGF\EA\201074 and UF150238, and the Emerson Collective		HAH thanks Mark Coles for introducing me to Julian C Knight

Raphael Heilig (R.H.)	11	6				
Svenja Hester (S.H.)	11	6				
Jennifer Hill (J.H.)	13	6				
Charles Hinds (C.H.)	3,15	10				
Clare Hird (C.Hi.)	15	6,7				
Ling-Pei Ho (L-P.H.)	4,5	1,5,10				
Renee Hoekzema (R.Ho.)	5	6	0000-0002-2347-6648	Emerson Collective		
Benjamin Hollis (B.H.)	2	5,8				
Jim Hughes (J.Hu.)	5	12	0000-0002-8955-7256	MRC grant code MC_UU_12009/14; Wellcome Trust Strategic Award, grant code 106130/Z/14/Z	Director and shareholder of Nucleome Therapeutics	
Paula Hutton (P.H.)	15	6,7				
Matthew A Jackson-Wood (M.A.J-W)	5	2,3,5,6,11	0000-0002-7891-6217	Kennedy Trust for Rheumatology Research, Junior Research Fellowship from Linacre College, Oxford	Currently employed by MiroBio Ltd.	
Ashwin Jainarayanan (A.J.)	1,12	1,2,3,4,5,6,8,10,11	0000-0003-3523-1477	ERC-2014-AdG_670930, UKRI-BBSRC grant BB/M011224/1 and the Oxford Interdisciplinary BioScience DTP		
Anna James-Bott (A.J-B.)	1	3	0000-0001-9658-5354			

Kathrin Jansen (K.J.)	1	2,3,5,6	0000-0002-3803-7680	Medical Research Council (MRC) MR/S035850/1		
Katie Jeffery (K.Je)	15	7	0000-0002-6506-2689			
Elizabeth Jones (E.J.)	13	6				
Luke Jostins (L.J.)	1,4,8,16	1,2,3,5,8,9,11,12				
Georgina Kerr (G.K.)	6,16	13				
David Kim (D.K.)	15	6,7	0000-0002-4345-661X			
Paul Klenerman (P.K.)	5,14,15,16	1,7,9,10,12,13,14	0000-0003-4307-9161	WT109965MA, UKRI UK-CIC, Huo Family Foundation, DHSC PITCH, National Institutes of Health (U192U19AI082 630)		

Julian C Knight (J.C.K.)	1,3,4,5,6,8,11,13,15,16	1,2,4,5,6,7,8,9,10,11,12,13,14	0000-0002-0377-5536	The research was supported by the COVID-19 Research Response Fund, University of Oxford, Wellcome Trust Core Award Grant Number 203141/Z/16/Z and the NIHR Oxford Biomedical Research Centre. The views expressed are those of the author(s) and not necessarily those of the NHS, the NIHR or the Department of Health. JCK is supported by Wellcome Investigator award (WT204969/Z/16/Z); the NIHR Oxford Biomedical Research Centre; Chinese Academy of Medical Sciences (CAMS) Innovation Fund for Medical		Wellcome Centre for Human Genetics Flow Cytometry Facility

				Science (CIFMS) (2018-I2M-2-002)		
Vinod Kumar (V.K.)	6	3,7,8		Kennedy Trust for Rheumatology Research		
Piyush Kumar Sharma (P.K.S.)	12	1,2,3,5,6,8,9,10,11	0000-0002-6247-3503			
Prathiba Kurupati (P.Ku.)	7	6		MRC HIU core funding		
Andrew Kwok (A.K.)	1,2,3,4,7,8,13,15	1,2,3,4,5,6,7,8,9,10,11	0000-0001-8928-5054			
Angela Lee (A.L.)	1,2,3,14	2,4,12,13,14	0000-0002-7591-825X	Wellcome Trust [Core Award 203141/Z/16/Z]		We thank the Oxford Genomics Centre at the Wellcome Centre for Human Genetics (funded by Wellcome Trust grant reference 203141/Z/16/Z) for the generation and initial processing of sequencing data
Aline Linder (A.Li)	13	6				

Teresa Lockett (T.L.)	15	6,7				
Lorne Lonie (L.L.)	13	12		Wellcome Trust [Core Award 203141/Z/16/Z]		
Maria Lopopolo (M.L.)	13	6,7	0000-0001-8502-9264	Wellcome Trust [Core Award 203141/Z/16/Z]		
Martyna Lukoseviciute (M.L u.)	1,2	2, 5	0000-0002-8915-5249			
Jian Luo (J.L.)	10	2,4,5,6,8,9,11	0000-0001-9009-065X			
Spyridoula Marinou (S.M.)	13	6				
Brian Marsden (B.M.)	6,16	7,8,12	0000-0002-1937-4091	Kennedy Trust for Rheumatology Research		
Jose Martinez (J.M.)	15	6,7		NIHR Clinical Research Network		
Philippa C Matthews (P.C.M.)	15	7	0000-0002-4036-4269	Wellcome funding (grant ref 110110/Z/15/Z)		
Michalina Mazurczyk (M.M.)	7	2	0000-0001-7727-9036	MRC HIU core funding		
Simon McGowan (S.McG)	5	11	0000-0001-9720-1914			
Stuart McKechnie (S.McK.)	15	7				
Adam Mead (A.M.)	1,13	12	0000-0001-8522-1002	CRUK Senior Cancer Research Fellowship		

Alexander J Mentzer (A.J.M.)	3,4,8,13,14,15,16	1,2,5,6,7,8,9,10,14	0000-0002-4502-2209	Oxford Biomedical Research Centre, Medical Research Council (grant MC_PC_19059) , Oxfordshire Health Services Research Committee		
Yuxin Mi (Y.M.)	5,8,11	5,6,8,11	0000-0002-8194-2977			
Claudia Monaco (C.M.)	7	1,5,6,10,12,13,14	0000-0003-1985-4914	BRD00230 - MSD COVID award for WP3		
Ruddy Montadon (R.M.)	1	6,7		Wellcome Centre for Human Genetics Flow Cytometry Facility		
Giorgio Napolitani (G.N.)	1,7,16	1,2,4,5,6,10,11,12,14	0000-0002-3605-6394	MRC HIU core funding		
Isar Nassiri (I.N.)	8,12	2,3,5,6,9	0000-0003-1886-1151			
Alex Novak (A.N.)	15	7	0000-0002-5880-8235			
Darragh P O'Brien (D.P.O'B.)	11	6	0000-0003-4924-7795			
Daniel O'Connor (D.O'C.)	3	5,11	0000-0002-6902-9886			
Denise O'Donnell (D.O'D.)	15	7				
Graham Ogg (G.O.)	7,10	1				
Lauren Overend (L.O.)	1,12	1,2,3,6	0000-0002-2802-048X	Wellcome Trust		
Inhye Park (I.P.)	7	6,7	0000-0002-8084-139X			
Ian Pavord (I.Pav.)	10	1		NIHR Oxford BRC		

Yanchun Peng (Y.P.)	13	7		Chinese Academy of Medical Sciences (CAMS) Innovation Fund for Medical Science (CIFMS), China (grant number: 2018-I2M-2-002 and MRC Unit Core		
Frank Penkava (F.P.)	12	1,3,5,6	0000-0001-9163-8576			
Mariana Pereira Pinho (M.P.P.)	7	2,4,5,8,10,11	0000-0002-5759-2436	MRC HIU core funding		
Elena Perez (E.P.)	15	6,7		NIHR Clinical Research Network		
Andrew J Pollard (A.J.P.)	13	12	0000-0001-7361-719X	Oxford NIHR BRC3	University of Oxford has entered a partnership with AstraZeneca for the development of a covid19 vaccine	
Fiona Powrie (F.Po.)	6,16	12				
Bethan Psaila (B.P.)	1	12	0000-0001-8198-9663	CRUK Advanced Clinician Scientist Fellowship; BRC Senior Fellowship		

T Phuong Quan (T.P.Q.)	4	6,8	0000-0001-8566-1817	Oxford Biomedical Research Centre, National Institute for Health Research Health Protection Research Unit (NIHR HPRU) in Healthcare Associated Infections and Antimicrobial Resistance at Oxford University in partnership with Public Health England (PHE) (grant HPRU-2012-10041)		
Emmanouela Repapi (E.R.)	5,7	2,4,5,8,10,11	0000-0001-8085-0731	UKRI Rutherford Fund (MR/S005471/1)		
Santiago Revale (S.R.)	1,2,3,12	2,3,4,5,6,7,8	0000-0001-5219-9582	This work was supported by the Wellcome Trust [Core Award 203141/Z/16/Z]		
Laura Silva-Reyes (L.S-R.)	13	6				
Jean-Baptiste Richard (J-B.R.)	1,5	5	0000-0002-4052-9772			
Charlotte Rich-Griffin (C.R-G.)	1	2,3,5,6,7,9,11	0000-0001-8212-9542	NIHR Oxford BRC (BRCRCF19-04)		
Thomas Ritter (T.R.)	15	6,7	0000-0002-3153-342X			

Christine S Rollier (C.S.R.)	13	6	0000-0002-9712-8080	NIHR Oxford BRC		
Matthew Rowland (M.R.)	15	7				
Fabian Ruehle (F.R.)	4,5	2,3,5	0000-0002-8409-9823			
Mariolina Salio (M.S.)	7	1,2,4,5,6,9,10,11,12	0000-0002-4821-3801	MRC HIU core funding		
Stephen Nicholas Sansom (S.N.S.)	1,4,5,6,16	1,2,3,4,5,6,7,8,9,10,11,12, 13	0000-0003-4569-8513	Kennedy Trust for Rheumatology Research, KENN171803 Core support		
Raphael Sanches Peres (R.S.P.)	1	5				
Alberto Santos Delgado (A.S.D.)	4,5,11	1,2,3,4,5,6,12	0000-0002-9163-7730			
Tatjana Sauka-Spengler (T.S-S.)	2,16	2,5,12				
Ron Schwessinger (R.S.)	8	5				
Giuseppe Scozzafava (G.S.)	13	6,7		RRF BRD00230		
Gavin Screaton (G.Sc.)	9	12		WT SIA and Schmidt Futures	Sits on GSK Vaccines SAB, founder RQ Biotechnology	
Anna Seigal (A.S.)	5	1,2				

Malcolm G Semple (M.G.S.)	4,6,13,15	1,6,7,8,12,13,14	0000-0001-9700-0418		MGS reports grants from DHSC, NIHR UK, MRC UK, HPRU in Emerging and Zoonotic Infections, and University of Liverpool, during the conduct of the study; and is chair of the Infectious Diseases Science Advisory Board and minority shareholder of Integrum Scientific, Greensboro NC, outside the submitted work.	
Martin Sergeant (M.S.)	5,6	11	0000-0001-7264-2668	Medical Research Council (MRC) Core Funding (MC_UU_00016/14), National		

				Institutes of Health (USA) grant number R24DK106766		
Christina Simoglou Karali (C.S.K.)	1,13	6,7	0000-0003-2169-6580			
David Sims (D.S.)	1	12		204826/Z/16/Z		
Donal Skelly (D.Sk.)	15	7	0000-0002-2426-3097	DS is an NIHR Academic Clinical Fellow		
Hubert Slawinski (H.S.)	1,2,13	6,7	0000-0002-8996-8477	Wellcome Trust [Core Award 203141/Z/16/Z]		
Alberto Sobrinodiaz (A.S.)	15	6,7		NIHR Clinical Research Network		
Nikolaos Sousos (N.S.)	1,13	6,7	0000-0002-9100-2954	CRUK Clinical Research Training Fellowship (C130623/A249 471)		
Lizzie Stafford (L.S.)	15	7				
Lisa Stockdale (L.St.)	13	6	0000-0002-2576-8783			
Marie Strickland (M.St.)	1,13	6,7				
Otto Sumray (O.S.)	5	2,3,5	0000-0003-1735-0595	UK Centre for Topological Data Analysis EPSRC grant EP/R018472/1		
Bo Sun (B.S.)	1,12	1,2,3,5,6,8,9,10,11	0000-0002-5507-6657	Patrick Berthoud Charitable Trust		
Chelsea Taylor (C.T.)	12	1,2,3,5,6,8,10,11	0000-0002-1635-4167			

Stephen Taylor (S.T.)	5	1,11,12	0000-0002-3559-4334	Medical Research Council (MRC) Core Funding (MC_UU_00016 /14)		
Adan Taylor (A.T.)	15	6,7				
Supat Thongjuea (S.Th.)	1	12	0000-0002-9129-4694			
Hannah Thraves (H.T.)	15	6,7		NIHR Clinical Research Network		
John A Todd (J.A.T.)	6,16	12,14	0000-0003-2740-8148	Wellcome Core Award Grant Number 203141/Z/16/Z supporting the Oxford Genomics Centre in the Wellcome Centre for Human Genetics, University of Oxford, and a Strategic Award to the Diabetes and Inflammation Laboratory from the JDRF (4-SRA-2017-473-A-A) and the Wellcome (107212/A/15/Z)	JAT is a member of the Human Genetics Advisory Board of GSK	
Adriana Tomic (A.T.)	5	2,3,5,6,8,12	0000-0001-9885-3535			
Orion Tong (O.T.)	12	2,3,5,6,9,11	0000-0002-0659-5944			

Amy Trebes (A.Tr.)	14	6,7	0000-0002-3715-9109	Wellcome Trust [Core Award 203141/Z/16/Z]		
Dominik Trzupek (D.T.)	7	7				
Felicia Anna Tucci (F.A.T.)	1,12	1,2,9,11,12	0000-0001-5813-9708	Wellcome Trust		
Lance Turtle (L.T.)	4,6,15	6,7,8,12,13,14	0000-0002-0778-1693	NIHR HPRU EZI, ISARIC4C MRC, Wellcome fellowship 205228/Z/16/Z		
Irina Udalova (I.U.)	7	1,5,6,10,12,13,14				
Holm Uhlig (H.U.)	15	7				
Erinke van Grinsven (E.V.G.)	7	6				
Iolanda Vendrell (I.V.)	11	2,4,6,10,12	0000-0001-7187-4061	COI		
Marije Verheul (M.V.)	13	6				
Alexandru Voda (A.V.)	1	3,5	0000-0003-2974-6992			
Guanlin Wang (G.W.)	1	5	0000-0002-5622-3890			
Lihui Wang (L.W.)	7	6				
Dapeng Wang (D.W.)	6	8	0000-0002-9925-4574			
Peter Watkinson (P.W.)	15	7				
Robert Watson (R.W.)	12	1,2,3,5,6,8,10,11	0000-0002-8838-9406			
Michael Weinberger (M.W.)	1,2	5	0000-0001-8235-6825	BHF Programme Grant RG/13/9/303269		
Justin Whalley (J.W.)	3,5,14	2,3,5	0000-0002-0403-3091			

Lorna Witty (L.W.)	13	12		Wellcome Trust [Core Award 203141/Z/16/Z]		
Katherine Wray (K.W.)	13	6,7		Haematology Theme Oxford Biomedical Research Centre		
Luzheng Xue (L.X.)	10,16	1,2,4,9,10,11,12	0000-0003-0042-1262	NIHR Oxford BRC		
Hing Yuen Yeung (H.Y.Y.)	1	5	0000-0002-2353-7076	Wellcome (204290/Z/16/Z) ; Rosetrees (R35579/AA002/M85-F2)		
Zixi Yin (Z.Y.)	13	7		Chinese Academy of Medical Sciences (CAMS) Innovation Fund for Medical Science (CIFMS), China (grant number: 2018-I2M-2-002 and MRC Unit Core		
Rebecca K Young (R.K.Y.)	15	6,7				
Jonathan Youngs (J.Y.)	4,13	7,10				
Ping Zhang (P.z.)	8	5	0000-0001-7063-7769			
Yasemin-Xiomara Zurke (Y-X.Z.)	1,7	2,3,5,6,8	0000-0002-3473-7129	BHF graduate studentship FS/18/63/34184		

ISARIC4C Consortium	15	7		National Institute for Health Research (NIHR; award CO-CIN-01), the Medical Research Council (MRC; grant MC_PC_19059), the NIHR Health Protection Research Unit in Emerging and Zoonotic Infections at University of Liverpool in partnership with Public Health England (PHE), in collaboration with Liverpool School of Tropical Medicine and the University of Oxford (NIHR award 200907)		The ISARIC WHO CCP-UK study protocol is available at https://isaric4c.net/protocols; study registry https://www.isrctn.com/ISRCTN66726260. This work uses data provided by patients and collected by the NHS as part of their care and support #DataSavesLives. We are grateful to the 2648 frontline NHS clinical and research staff and volunteer medical students who collected the data in challenging circumstances; and the generosity of the participants and their families for their individual contributions in these difficult times.

Notes
[1]From CRediT taxonomy; https://www.cell.com/pb/assets/raw/shared/guidelines/CRediT-taxonomy.pdf
