# Supplementary material for: A blood atlas of COVID-19 defines hallmarks of disease severity and specificity
Source: Cell. 2022 Mar 3;185(5):916–938.e58. doi: 10.1016/j.cell.2022.01.012 (PMC8776501; doi:10.1016/j.cell.2022.01.012)
Supplement: Data S2: Clinical phenotyping, related to STAR Methods and Table S1 [file mmc6.pdf]

Data S2: Clinical Phenotyping, Related to STAR Methods and Table S1

Related to **STAR Methods**: Clinical phenotyping and **Table S1: Demographics and clinical phenotyping for overall cohorts and hospitalized COVID-19 cohort**.

Table of Contents

1: Correlation matrix of clinical covariates and markers of response in hospitalized COVID-19 cases 2

2: Patient clustering by acute physiology. 3

3: Overview of hospitalized COVID-19 sampling by time from symptom onset and WHO severity (categorical) with maximal severity indicated 4

4: Admission samples for hospitalized COVID-19 (n=116) and sepsis (n=58) including total and differential cell count and clinically assayed biomarkers. 5

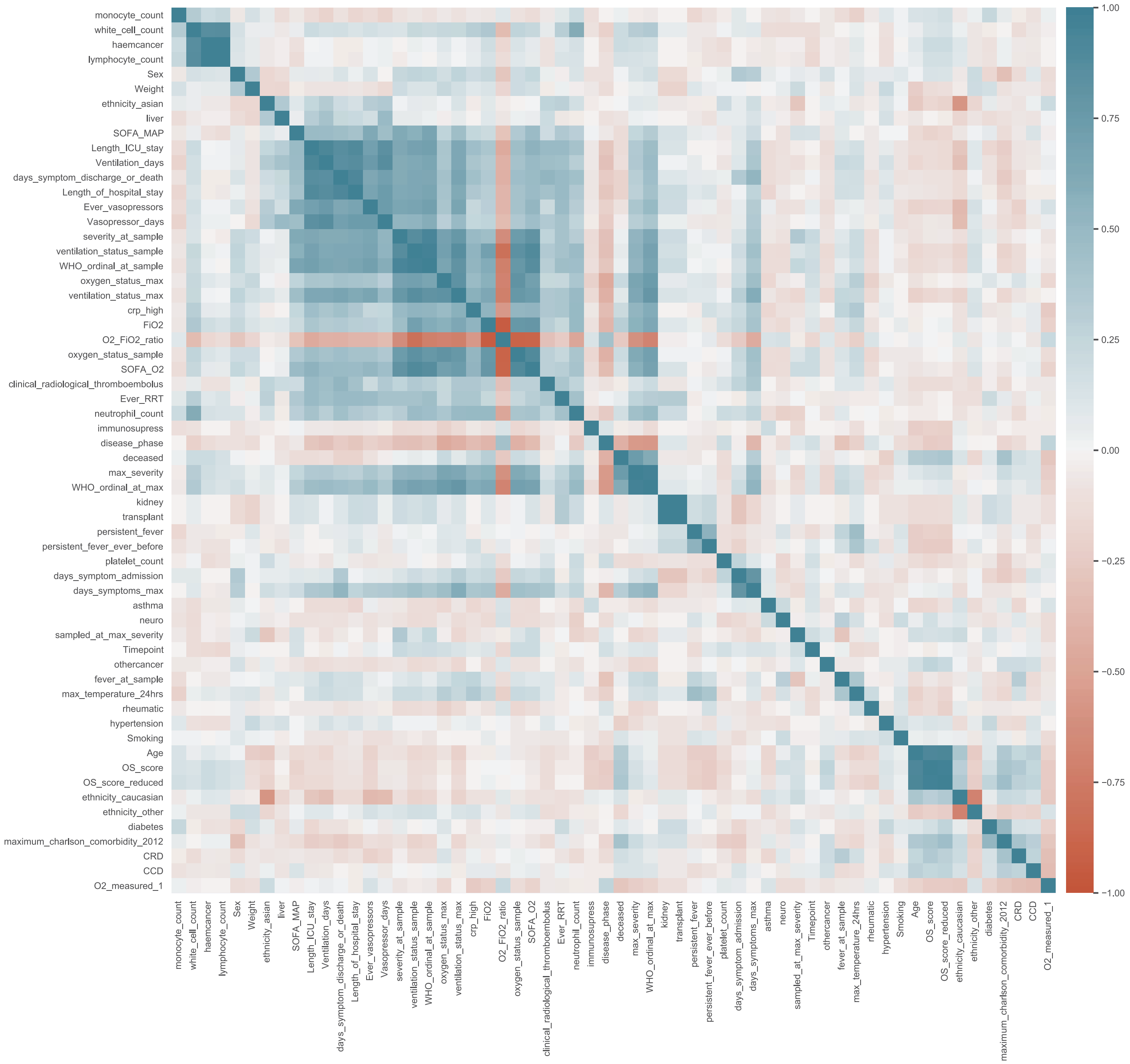

1: Correlation matrix of clinical covariates and markers of response in hospitalized COVID-19 cases. There were positive correlations (spearman's rho >0.5) between markers of severity based on WHO severity scales, oxygenation status, ventilation status, Sequential Organ Failure Assessment (SOFA) oxygenation score, vasopressor use, length of intensive care unit (ICU) stay, days on mechanical ventilation, C-reactive protein (CRP) and neutrophil count. Related to STAR Methods: Clinical phenotyping: admission and disease timescales.

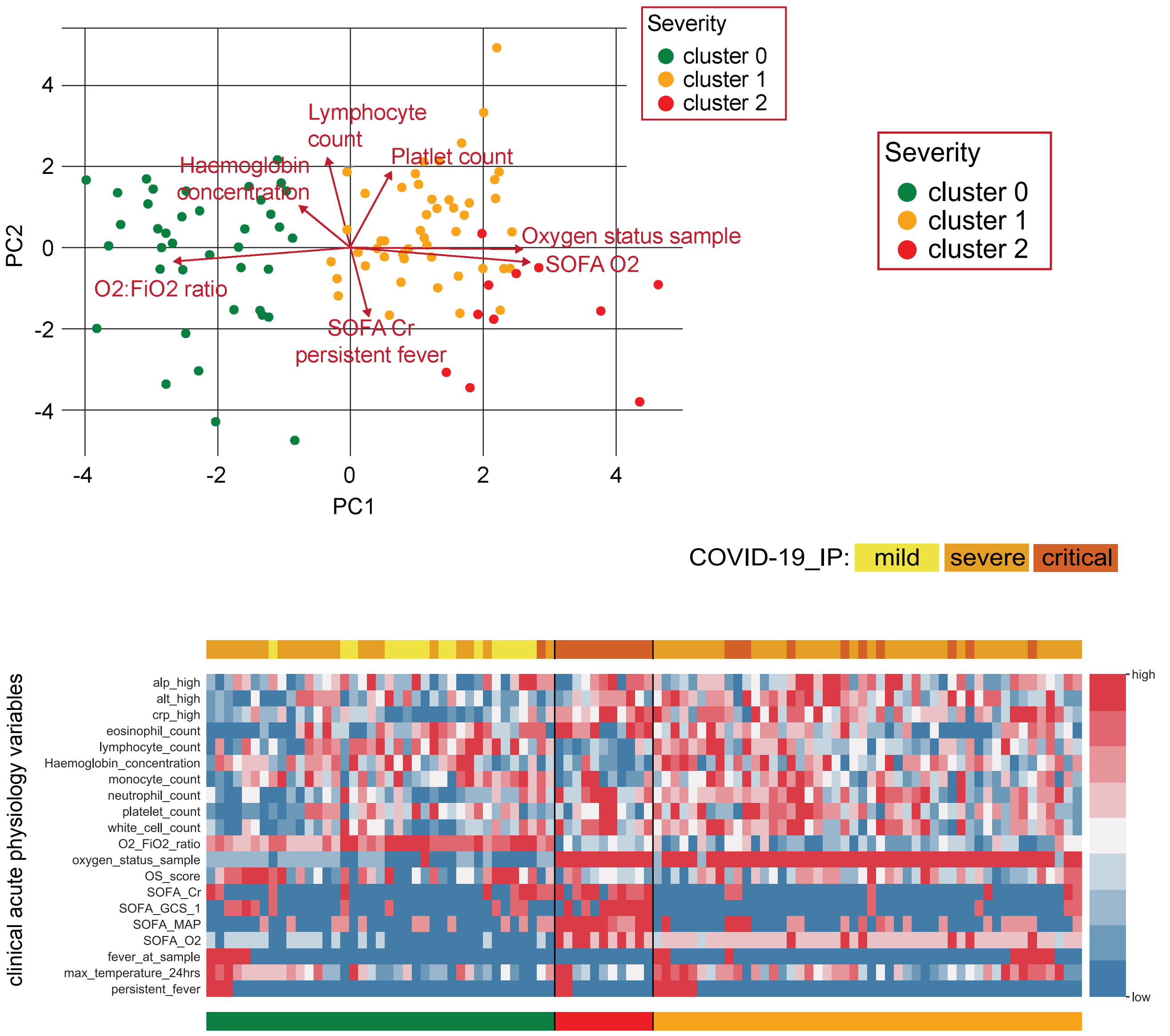

2: Patient clustering by acute physiology. Acute measures of physiology and clinical biomarkers of response without significant missingness (including measures of oxygenation requirements, blood cell counts, fever, ALT, CRP). Related to STAR Methods: Clinical phenotyping: admission and disease timescales.

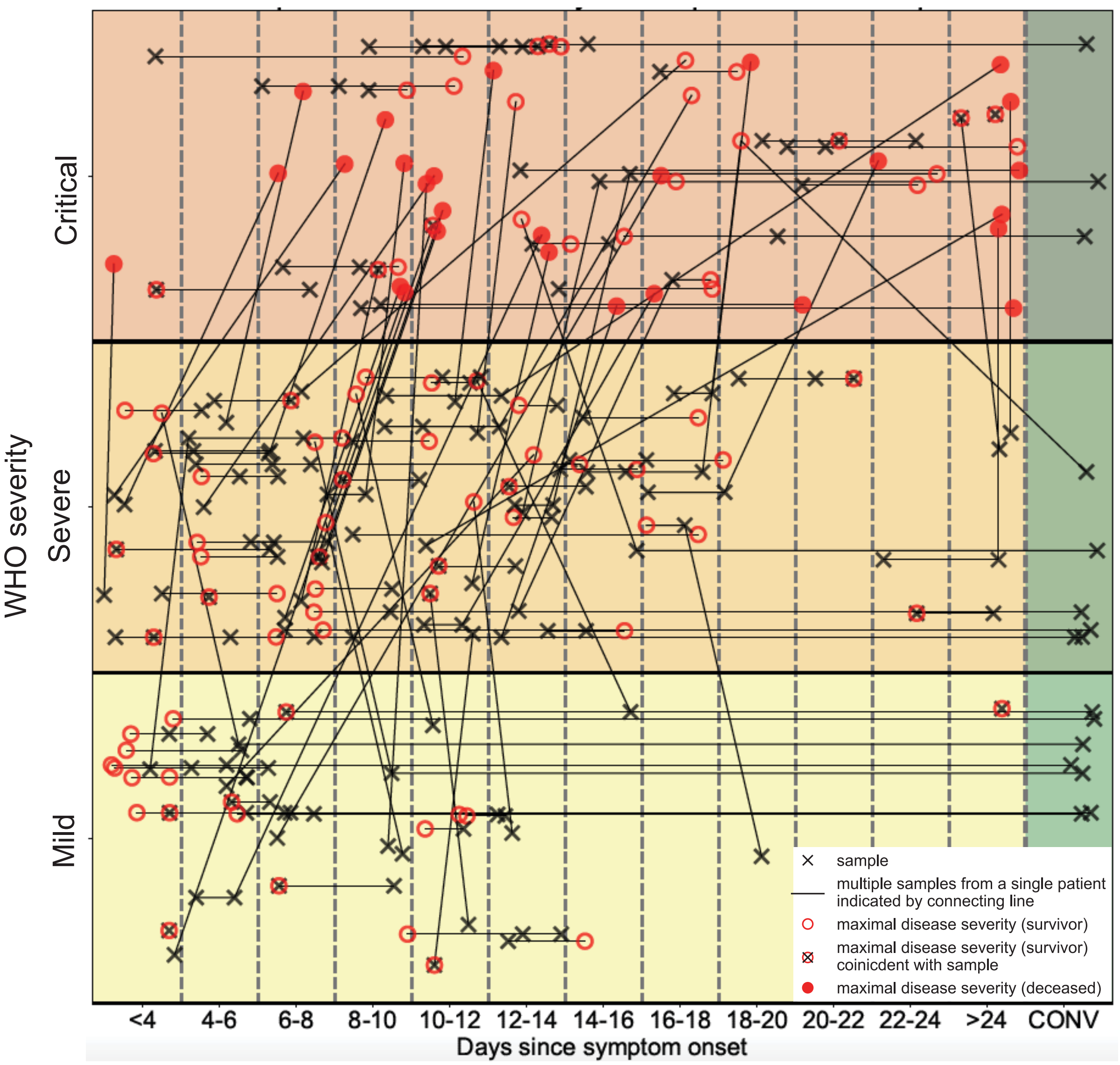

3: Overview of hospitalized COVID-19 sampling by time from symptom onset and WHO severity (categorical) with maximal severity indicated. Related to STAR Methods: Clinical phenotyping: admission and disease timescales.

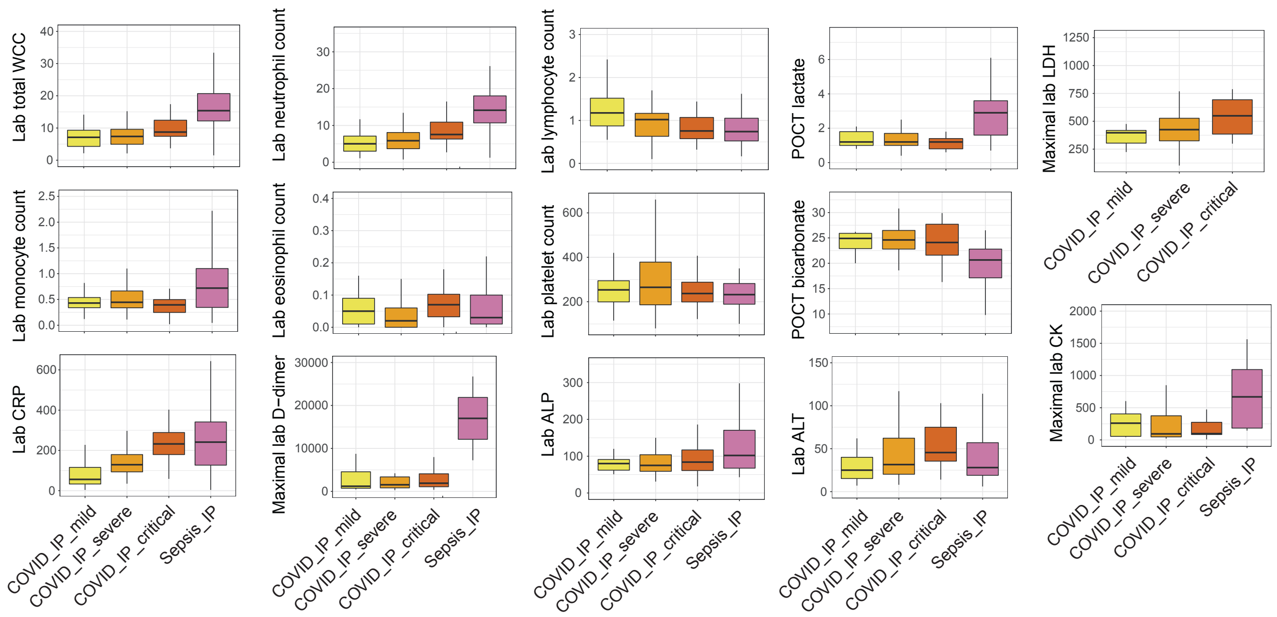

4: Admission samples for hospitalized COVID-19 (n=116) and sepsis (n=58) including total and differential cell count and clinically assayed biomarkers. C reactive protein (CRP), D-dimer, lactate dehydrogenase (LDH), creatine kinase (CK), alanine aminotransferase (ALT) and alkaline phosphatase (ALP). Related to STAR Methods: Clinical phenotyping: other clinical and therapeutic tests and interventions.
