# Supplementary material for: A blood atlas of COVID-19 defines hallmarks of disease severity and specificity
Source: Cell. 2022 Mar 3;185(5):916–938.e58. doi: 10.1016/j.cell.2022.01.012 (PMC8776501; doi:10.1016/j.cell.2022.01.012)
Supplement: Data S3: Analysis of mass and flow cytometry, repertoire, and proteomics, related to STAR Methods [file mmc7.pdf]

Data S3: Analysis of Mass and Flow Cytometry, Repertoire and Proteomics, Related to STAR Methods

Related to **STAR Methods**: Mass cytometry data analysis; Flow cytometry data analysis; Repertoire analysis; Pre-processing and analysis of surface protein (ADT) data; Luminex data analysis; and Tims-TOF mass spectrometry analysis.

Table of Contents

Mass cytometry *3*

1: Gating strategy for clean-up. **3**

2: Gating strategy for doublets exclusion. **4**

3: Flow-chart describing the analytical pipeline for the analysis of the mass cytometry data. **5**

4: Whole blood (non-depleted) assayed by single cell mass cytometry (CyTOF). **6**

5: Principal components analysis (PCA) of whole blood (non-depleted) assayed by single cell mass cytometry (CyTOF). **7**

6: Whole blood (depleted) assayed by single cell mass cytometry **9**

7: Myeloid cell populations from whole blood (depleted) assayed by single cell mass cytometry **10**

8: Single cell mass cytometry (CyTOF) compositional analysis showing UMAP, clustering and differential abundance of major B cell populations. **12**

9: Phenotype of the T/NK cell populations assayed by single cell mass cytometry. **13**

Multicolor flow cytometry analysis *14*

10: Phenotype and frequency of subsets of memory CD4 T cell subsets defined based on the expression of CCR4, CCR6 and CXCR3 .... **14**

Repertoire analysis *15*

11: UMAP embedding B cell repertoire analysis (A) UMAP embedding with cluster identities from CITE-seq (B) UMAP embedding of all B cells from single cell VDJ dataset. **15**

12: Illustration of the method used to identify CDR3 Kmers associated with COVID-19 compared to cells from healthy volunteers and patients with sepsis **16**

13: Cluster proportions **17**

14: Mean plasmablast diversity indices by comparator group; clonal expansion index, CEI, clonal diversification index, CDI. **18**

15: Correlation analysis of IGHV genes detected by single cell VDJ data and RNA VDJ data, within the (top) IGHD/M unmutated, (middle) IGHD/M mutated and (bottom) class-switched VDJ sequences. ... **19**

16: Volcano plots of log_2 fold change of IGHV gene usage of IGHD/M unmutated sequences (derived from single cell VDJ data), IGHD/M mutated sequences (derived from RNA VDJ data), class-switched sequences (derived from RNA VDJ data). **20**

17: Proportion of constant region genes across B cell clusters per study group **21**

18: Clonal overlap across comparator groups with total number of convergent clones per group shown with clonal network depicting distribution of convergent clusters (inset) and sequence logos of COVID-19 exclusive clusters (with IGHV/J usage and number of members). **22**

19: Cumulative bar chart of the frequencies of known SARS-CoV-2 binding BCRs per patient group. ... **23**

Proteomics *24*

20: Clustering of surface protein (ADT) data 24

21: Plasma protein abundance for specific proteins assayed by Luminex assay. 25

22: Heat map showing abundance of plasma and serum proteins by disease groups assayed using Luminex 26

23: UMAP of plasma proteins assayed by Luminex assay, colored by comparator group. 27

24: Unsupervised hierarchical clustering and supervised correlation analysis of plasma proteins assayed by HT-LC-MS/MS mass spectrometry. 28

25: Plasma protein abundance for specific proteins assayed by HT-LC-MS/MS mass spectrometry. 29

Mass cytometry

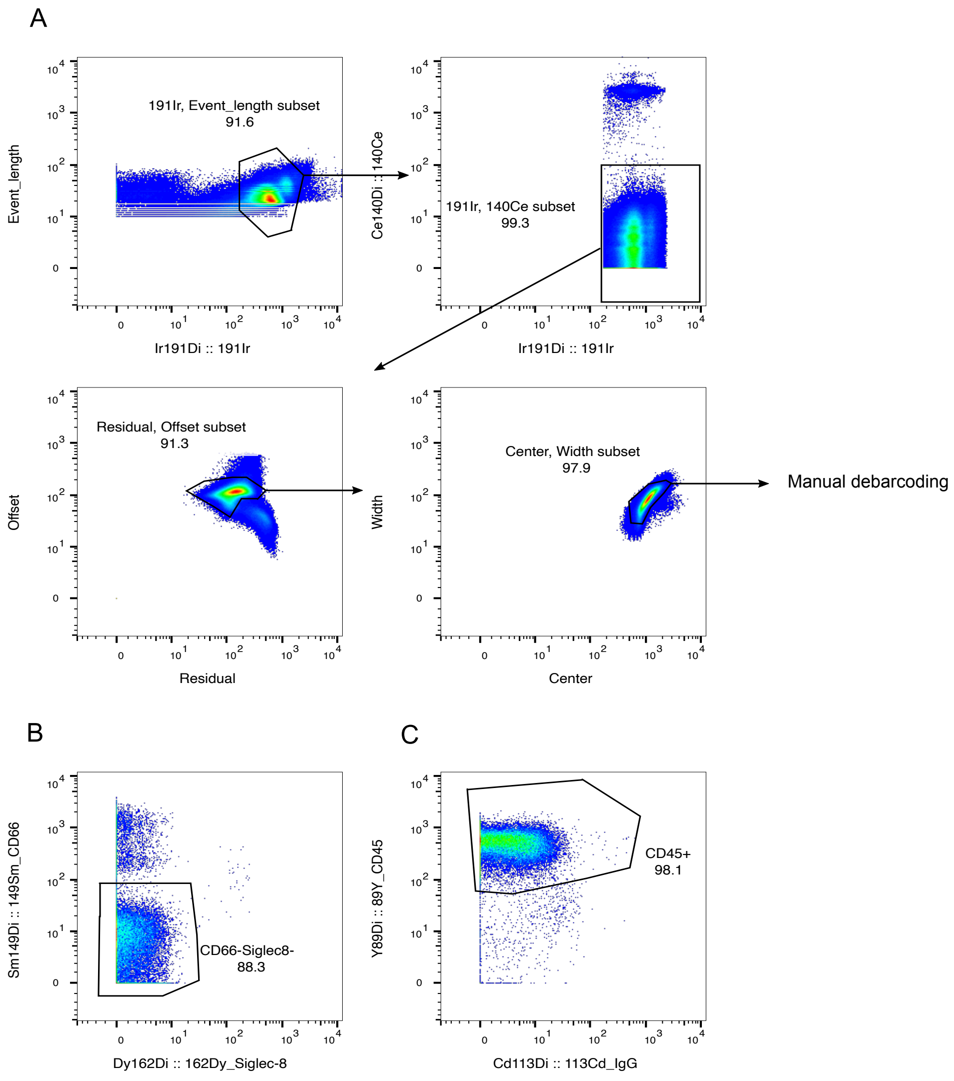

1: Gating strategy for clean-up. A Gating strategy for removal of beads, DNA negative events and for Gaussian parameters clean-up prior to Manual Debarcoding. **B** Clean up step to remove residual CD66+ granulocytes and Siglec-8 Eosinophils, in granulocytes depleted samples. This step was followed by gating of CD45+ cells. **C**. Gating of CD45+ cell for subsequent analysis. This step was performed for both depleted and non-depleted (original) samples. Related to STAR Methods: Mass cytometry data analysis: CyTOF data pre-processing.

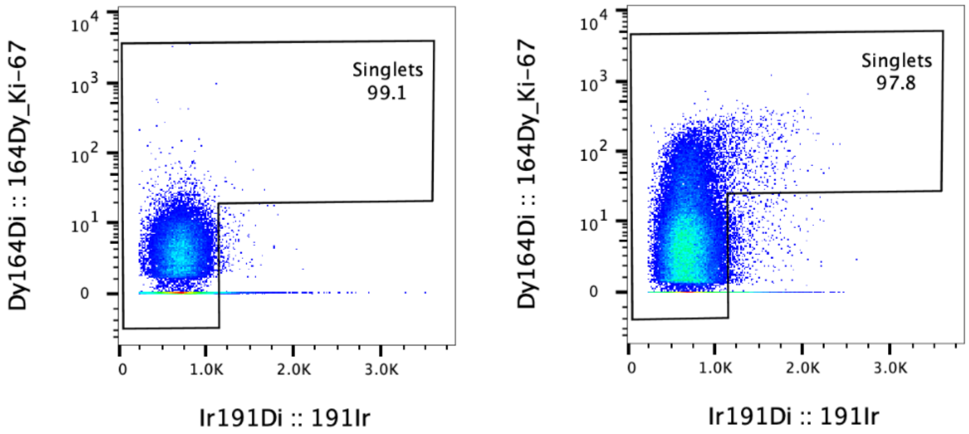

2: Gating strategy for doublets exclusion. Representative scatter plots showing DNA content on x-axis (191Ir - Iridium) versus the proliferation marker Ki67 on y-axis. The sample on the right shows a population of proliferating Ki67^{+} cells absent on the sample on the left. To not exclude proliferating cells from the analysis, the gating was made excluding only Iridium^{high}Ki67^{-} cells. Related to STAR Methods: Mass cytometry data analysis**:** CyTOF data pre-processing**.**

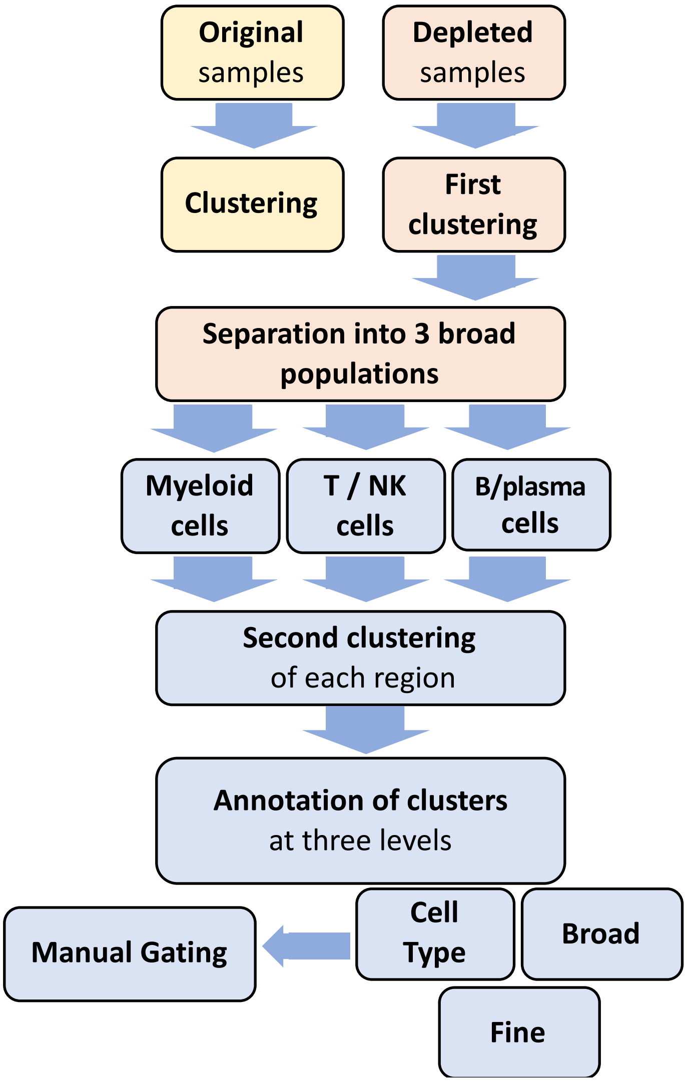

3: Flow-chart describing the analytical pipeline for the analysis of the mass cytometry data. Related to STAR Methods: Mass cytometry data analysis: clustering.

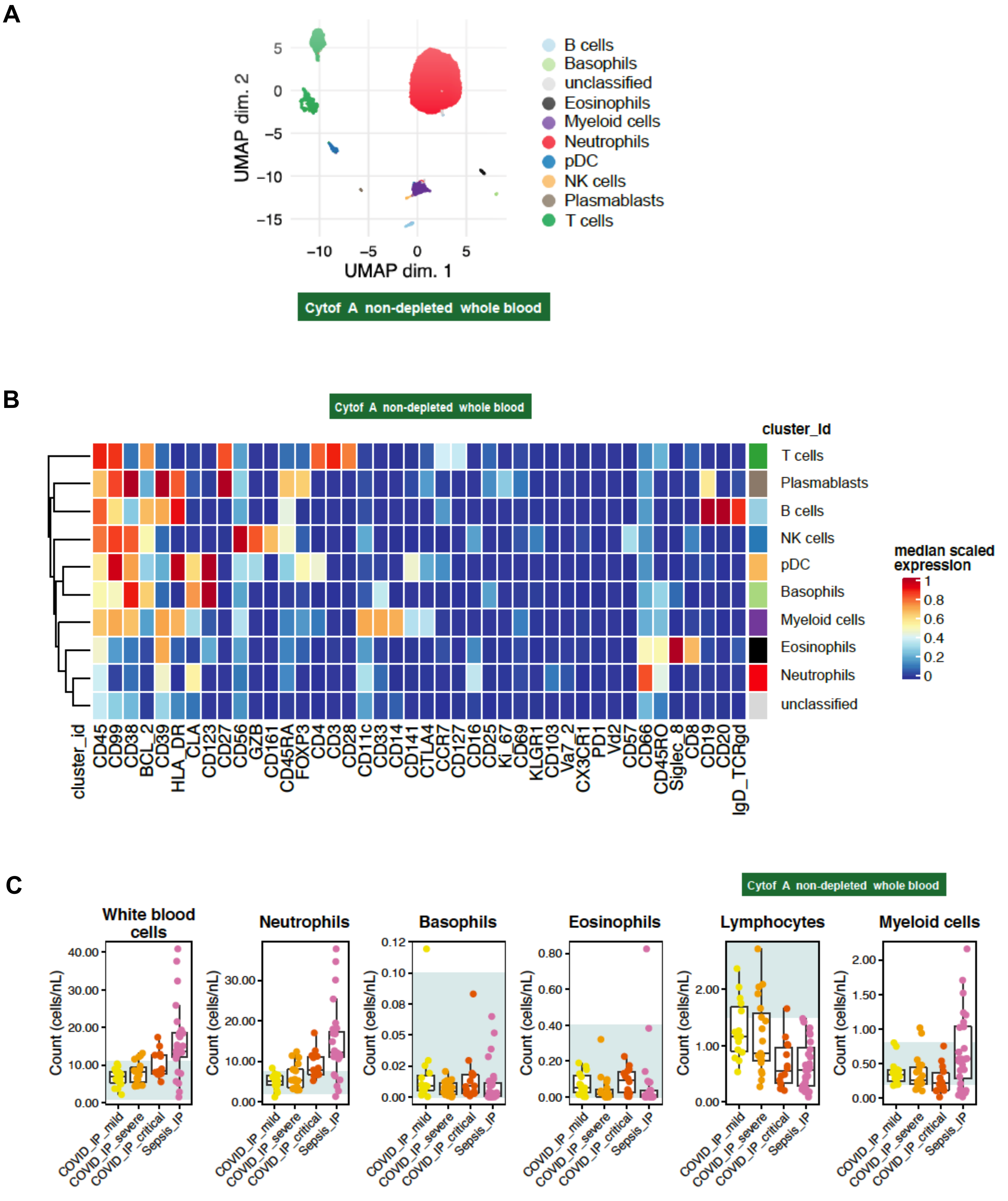

4: Whole blood (non-depleted) assayed by single cell mass cytometry (CyTOF). Non-granulocyte depleted samples. A self-organizing map algorithm (FlowSOM) resolved 25 clusters by consensus clustering for 3,893,390 cells after down sampling to a maximum of 40,000 cells. Clusters merged to identify broad immune cell populations (A) UMAP (B) clustering of major cell populations (y axis) by discriminating marker (x-axis) (C) cell counts by patient group. Related to STAR Methods: Mass cytometry data analysis: differential abundance analysis.

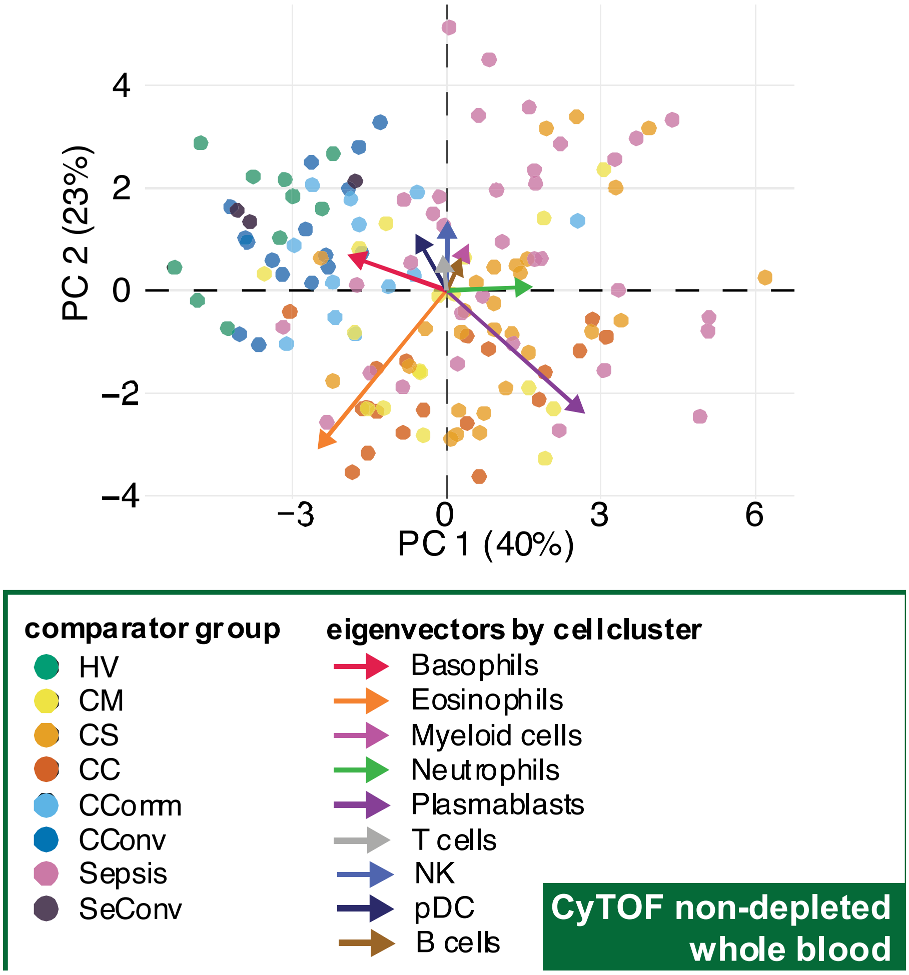

5: Principal components analysis (PCA) of whole blood (non-depleted) assayed by single cell mass cytometry (CyTOF). Major cell populations in whole blood for clinical comparator groups showing for non-granulocyte depleted samples PCA with arrows indicating drivers of variation by cell population. Related to STAR Methods: Mass cytometry data analysis: differential abundance analysis.

A

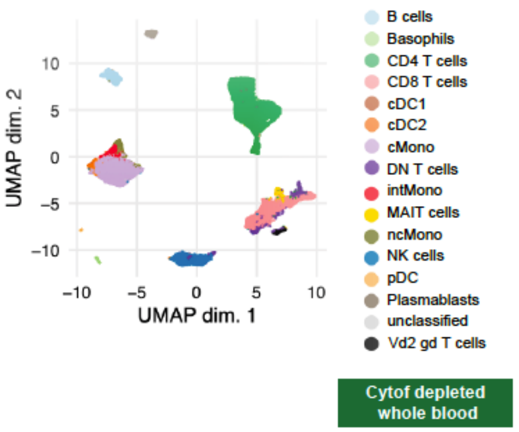

UMAP dim. 2
UMAP dim. 1
B cells
Basophils
CD4 T cells
CD8 T cells
cDC1
cDC2
cMono
DN T cells
intMono
MAIT cells
ncMono
NK cells
pDC
Plasmablasts
unclassified
Vd2 gd T cells
Cytof depleted whole blood

B

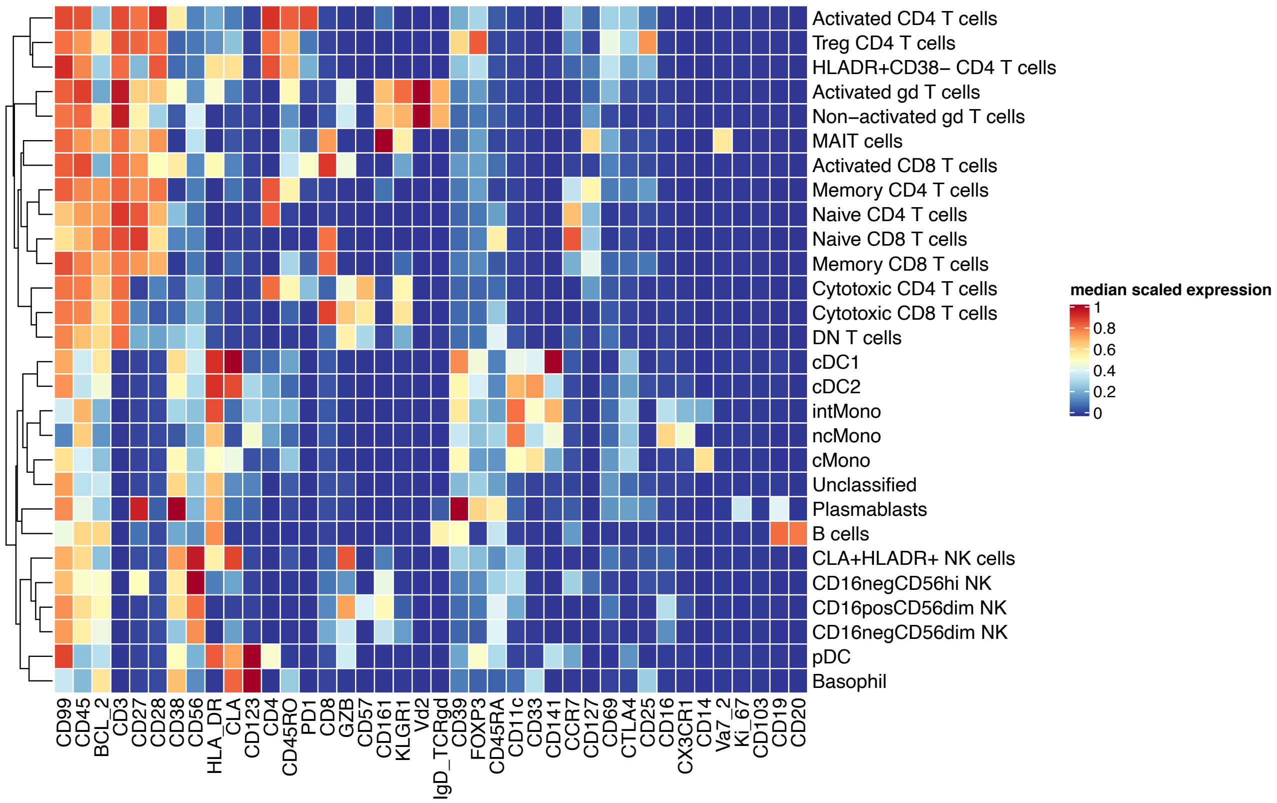

Activated CD4 T cells
Treg CD4 T cells
HLADR+CD38− CD4 T cells
Activated gd T cells
Non−activated gd T cells
MAIT cells
Activated CD8 T cells
Memory CD4 T cells
Naive CD4 T cells
Naive CD8 T cells
Memory CD8 T cells
Cytotoxic CD4 T cells
Cytotoxic CD8 T cells
DN T cells
cDC1
cDC2
intMono
ncMono
cMono
Unclassified
Plasmablasts
B cells
CLA+HLADR+ NK cells
CD16negCD56hi NK
CD16posCD56dim NK
CD16negCD56dim NK
pDC
Basophil
median scaled expression
CD99 CD45 BCL_2 CD3 CD27 CD28 CD38 CD56 HLA_DR CLA CD123 CD4 CD45RO PD1 CD8 GZB CD57 CD161 KLGR1 Vd2 IgD_TCRgd CD39 FOXP3 CD45RA CD11c CD33 CD141 CCR7 CD127 CD69 CTLA4 CD25 CD16 CX3CR1 CD14 Va7_2 Ki_67 CD103 CD19 CD20

C

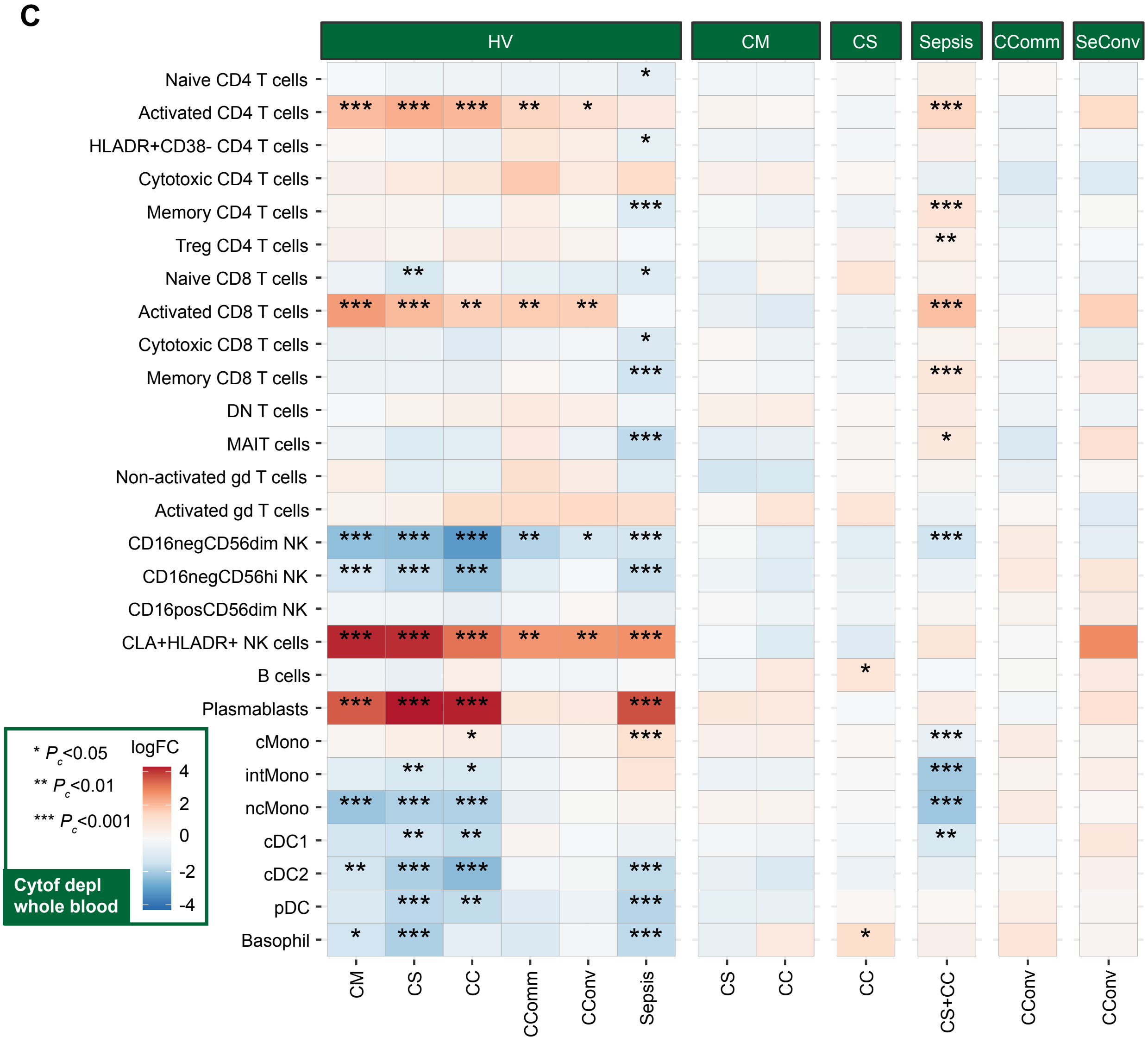

6: Whole blood (depleted) assayed by single cell mass cytometry. Heatmap showing the phenotype of broad cell populations for single cell mass cytometry (CyTOF) of granulocyte (CD66+) depleted whole blood with 7,118,158 cells assayed. (A) UMAP (B) clustering of major cell populations (y axis) by discriminating marker (x-axis) (C) differential abundance analysis. Related to STAR Methods: Mass cytometry data analysis: differential abundance analysis.

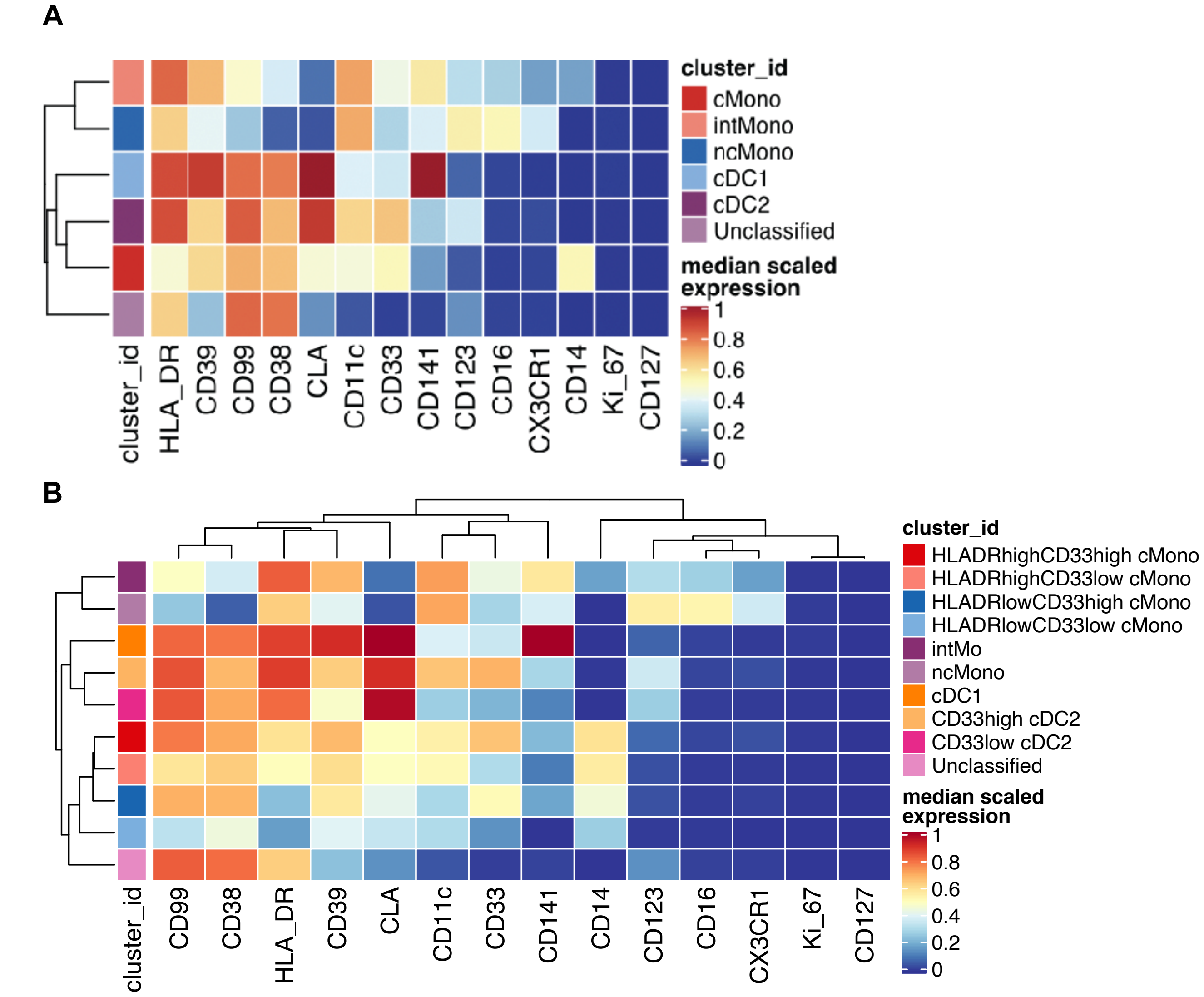

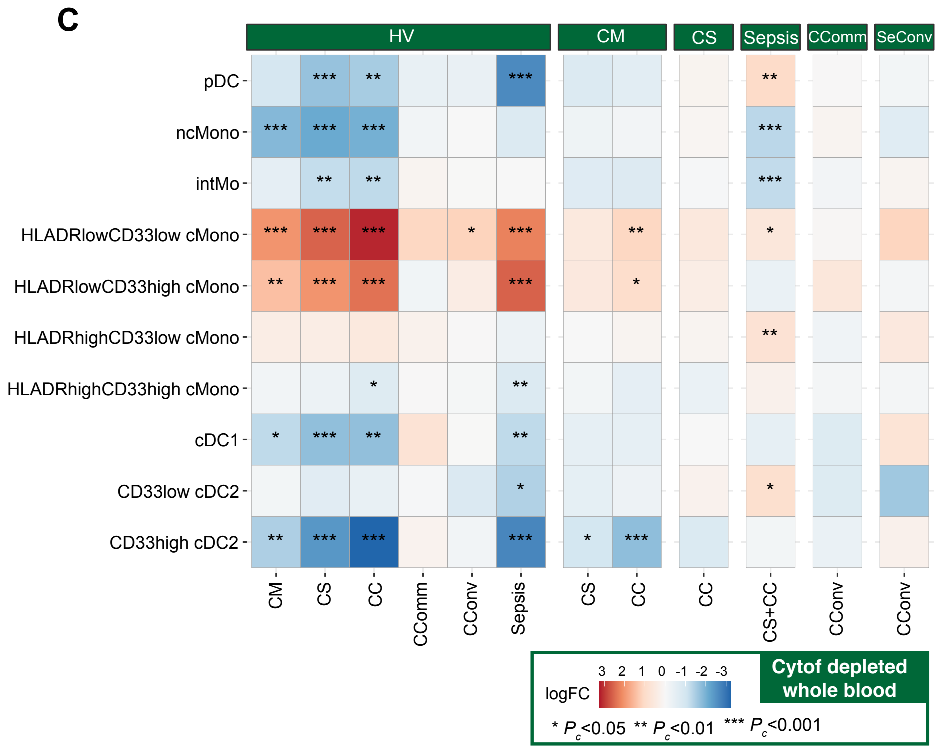

7: Myeloid cell populations from whole blood (depleted) assayed by single cell mass cytometry. (A) Overall populations. (B) Fine scale populations. (C) differential abundance analysis. Related to STAR Methods: Mass cytometry data analysis: differential abundance analysis.

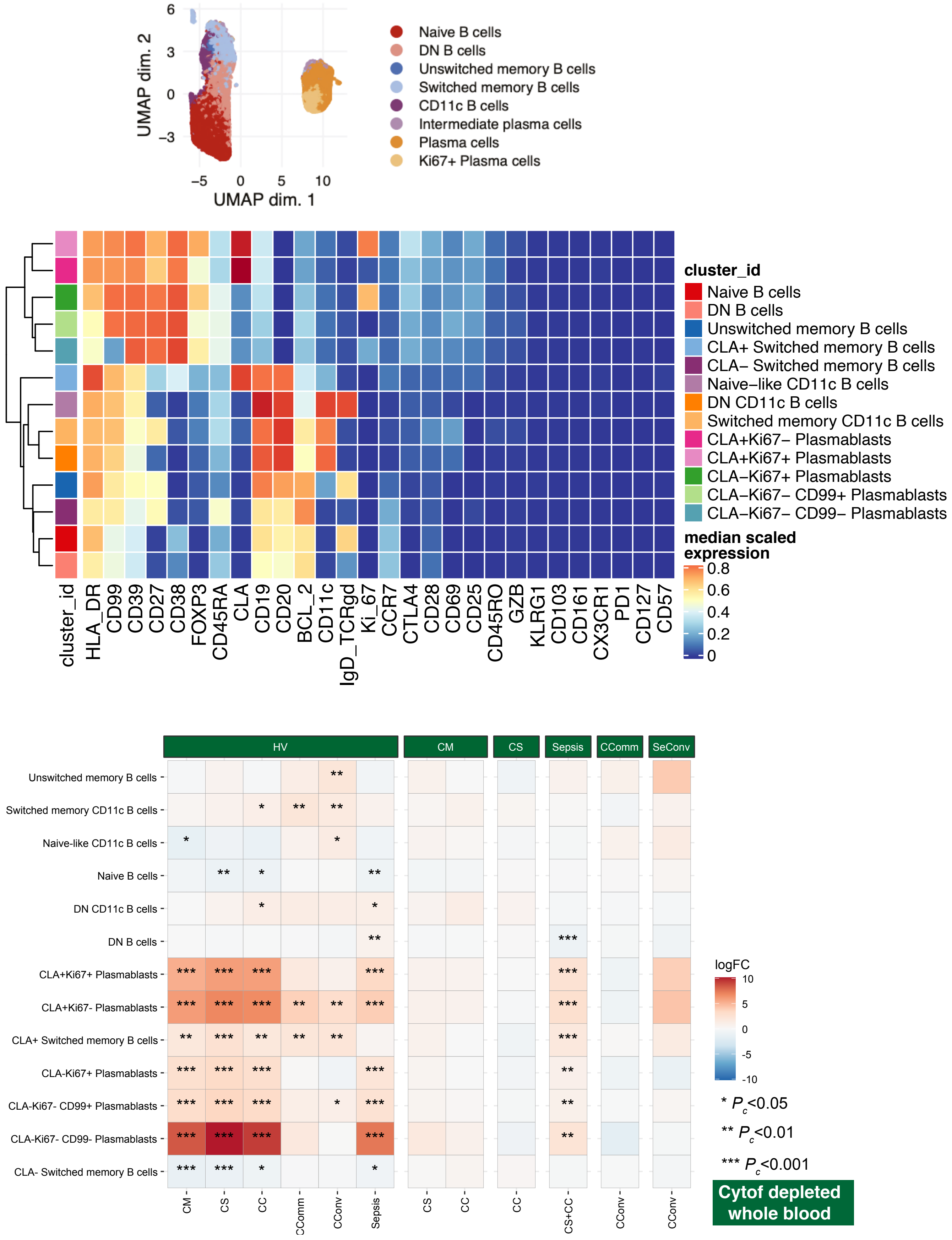
UMAP dim. 1
UMAP dim. 2
Naive B cells
DN B cells
Unswitched memory B cells
Switched memory B cells
CD11c B cells
Intermediate plasma cells
Plasma cells
Ki67+ Plasma cells
cluster_id
Naive B cells
DN B cells
Unswitched memory B cells
CLA+ Switched memory B cells
CLA– Switched memory B cells
Naive–like CD11c B cells
DN CD11c B cells
Switched memory CD11c B cells
CLA+Ki67– Plasmablasts
CLA+Ki67+ Plasmablasts
CLA–Ki67+ Plasmablasts
CLA–Ki67– CD99+ Plasmablasts
CLA–Ki67– CD99– Plasmablasts
median scaled expression
cluster_id HLA_DR CD99 CD39 CD27 CD38 FOXP3 CD45RA CLA CD19 CD20 BCL_2 CD11c IgD_TCRgd Ki_67 CCR7 CTLA4 CD28 CD69 CD25 CD45RO GZB KLRG1 CD103 CD161 CX3CR1 PD1 CD127 CD57
HV
CM
CS
Sepsis
CComm
SeConv
Unswitched memory B cells
Switched memory CD11c B cells
Naive-like CD11c B cells
Naive B cells
DN CD11c B cells
DN B cells
CLA+Ki67+ Plasmablasts
CLA+Ki67- Plasmablasts
CLA+ Switched memory B cells
CLA-Ki67+ Plasmablasts
CLA-Ki67- CD99+ Plasmablasts
CLA-Ki67- CD99- Plasmablasts
CLA- Switched memory B cells
CM CS CC CComm CConv Sepsis CS CC CC CS+CC CConv CConv
logFC
* P_c<0.05
** P_c<0.01
*** P_c<0.001
Cytof depleted whole blood

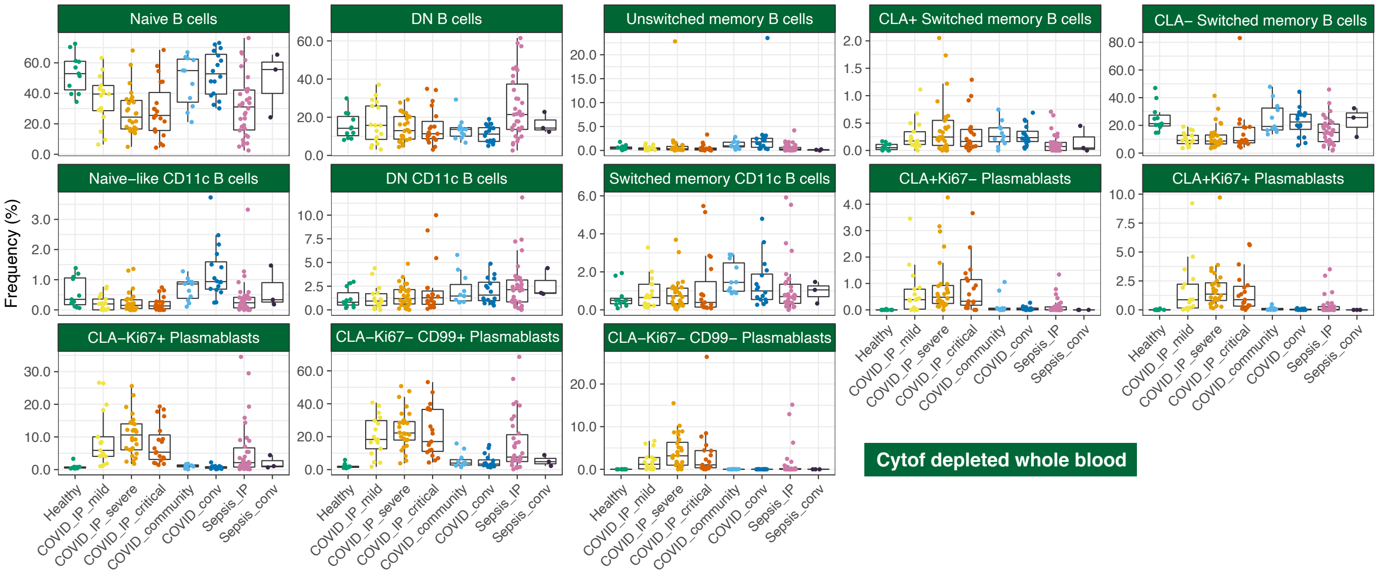

8: Single cell mass cytometry (CyTOF) compositional analysis showing UMAP, clustering and differential abundance of major B cell populations. Related to STAR Methods: Mass cytometry data analysis: differential abundance analysis.

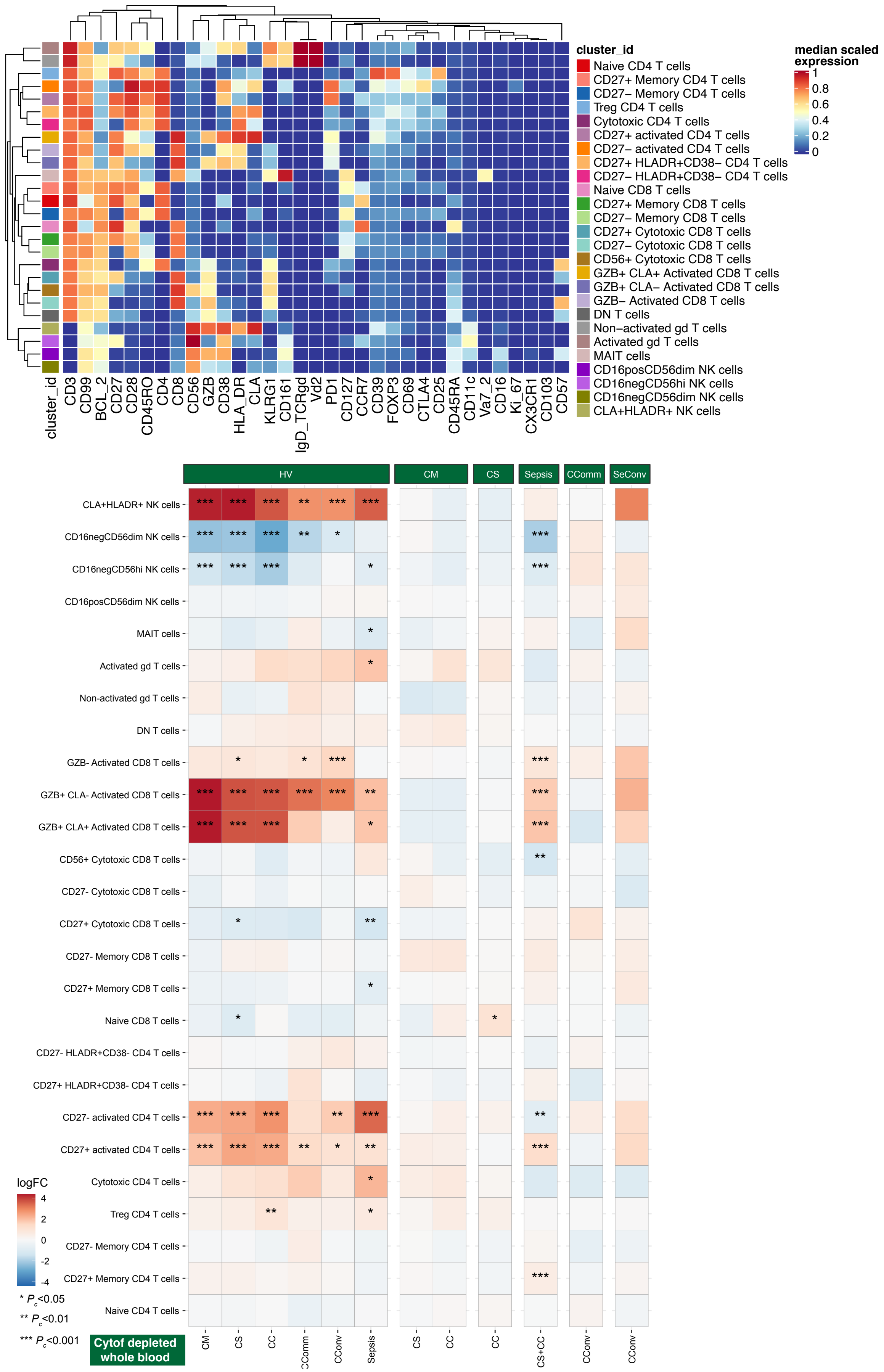

9: Phenotype of the T/NK cell populations assayed by single cell mass cytometry. (A) cell clustering (B) differential abundance analysis. Related to STAR Methods: Mass cytometry data analysis: differential abundance analysis.

Multicolor flow cytometry analysis

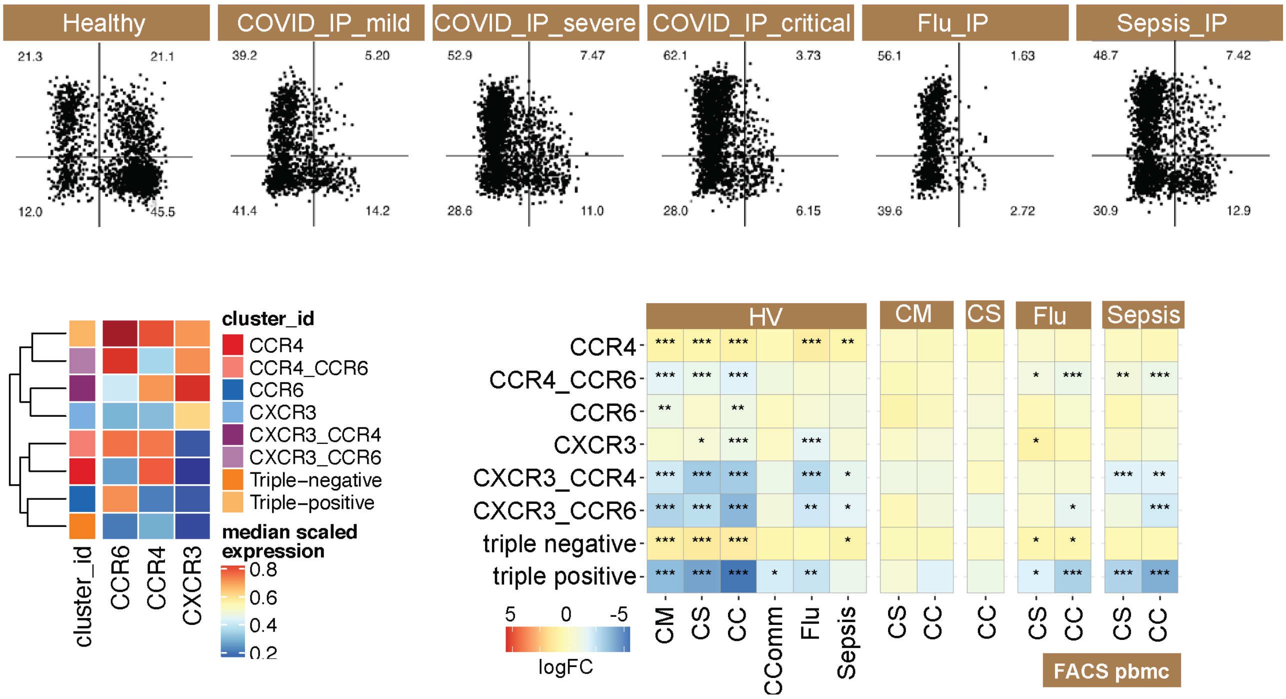

10: Phenotype and frequency of subsets of memory CD4 T cell subsets defined based on the expression of CCR4, CCR6 and CXCR3. PBMCs assayed using multicolor flow cytometry analysis. Dotplots, clustering and heatmap. Relates to Figure S4A-C. Related to STAR Method: Flow cytometry data analysis.

Repertoire analysis

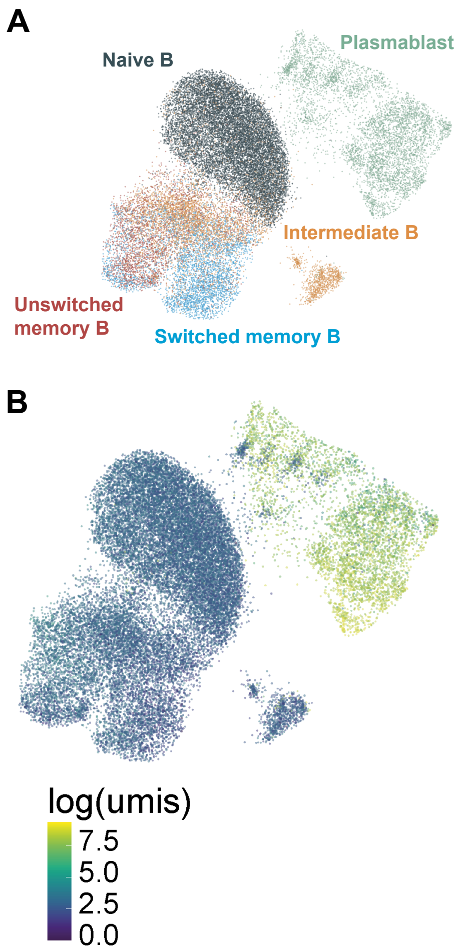

11: UMAP embedding B cell repertoire analysis (A) UMAP embedding with cluster identities from CITE-seq (B) UMAP embedding of all B cells from single cell VDJ dataset. As expected, PB showed the highest BCR expression in the CITEseq dataset. Color scale in panel B depicts log total UMIs per VDJ sequence. Related to STAR Methods: Repertoire analysis: additional pre-processing of scBCR-seq data.

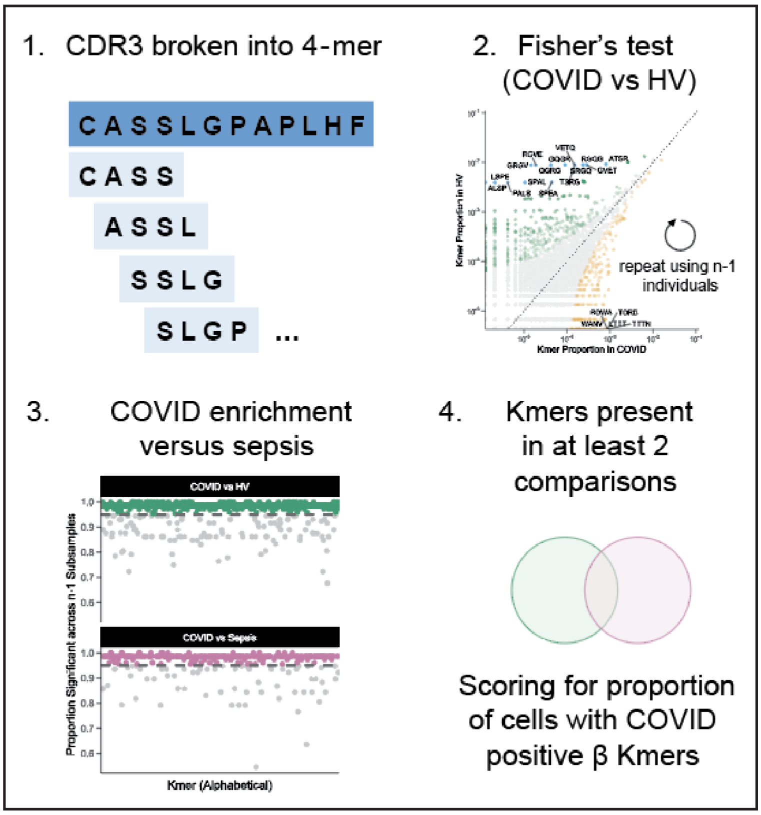

12: Illustration of the method used to identify CDR3 Kmers associated with COVID-19 compared to cells from healthy volunteers and patients with sepsis. Related to STAR Methods: Repertoire analysis: scTCR cytotoxicity and Kmer analysis.

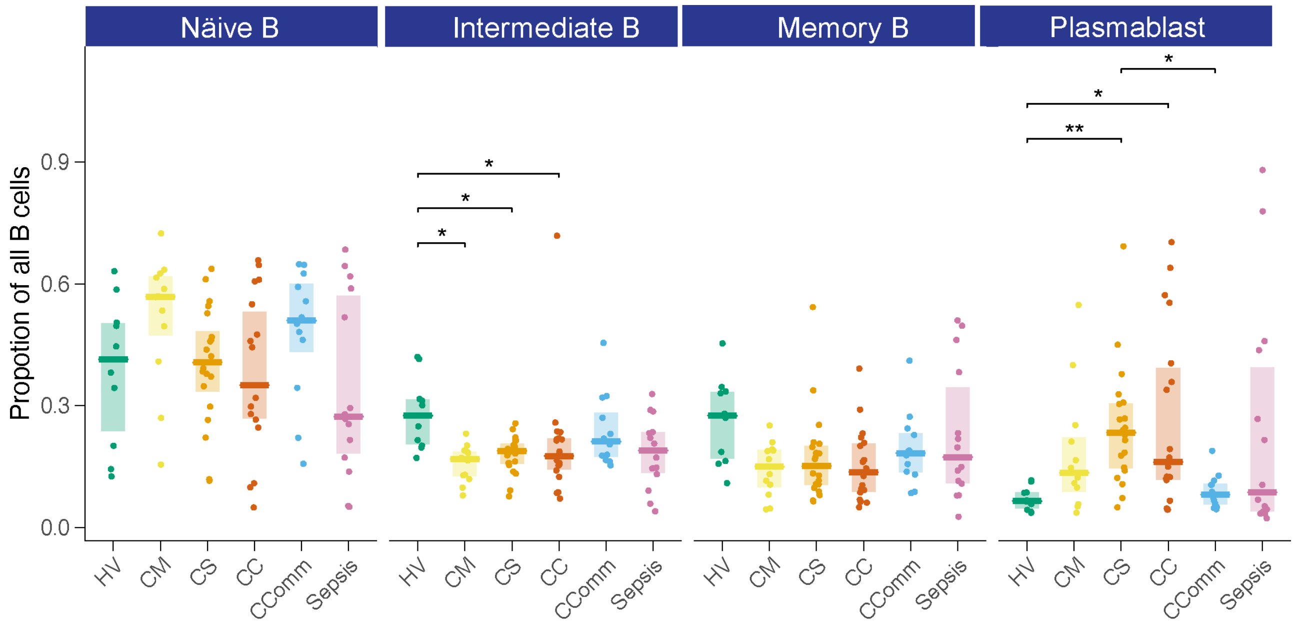

13: Cluster proportions. Related to STAR Methods: Repertoire analysis: RNA-velocity B cell analysis.

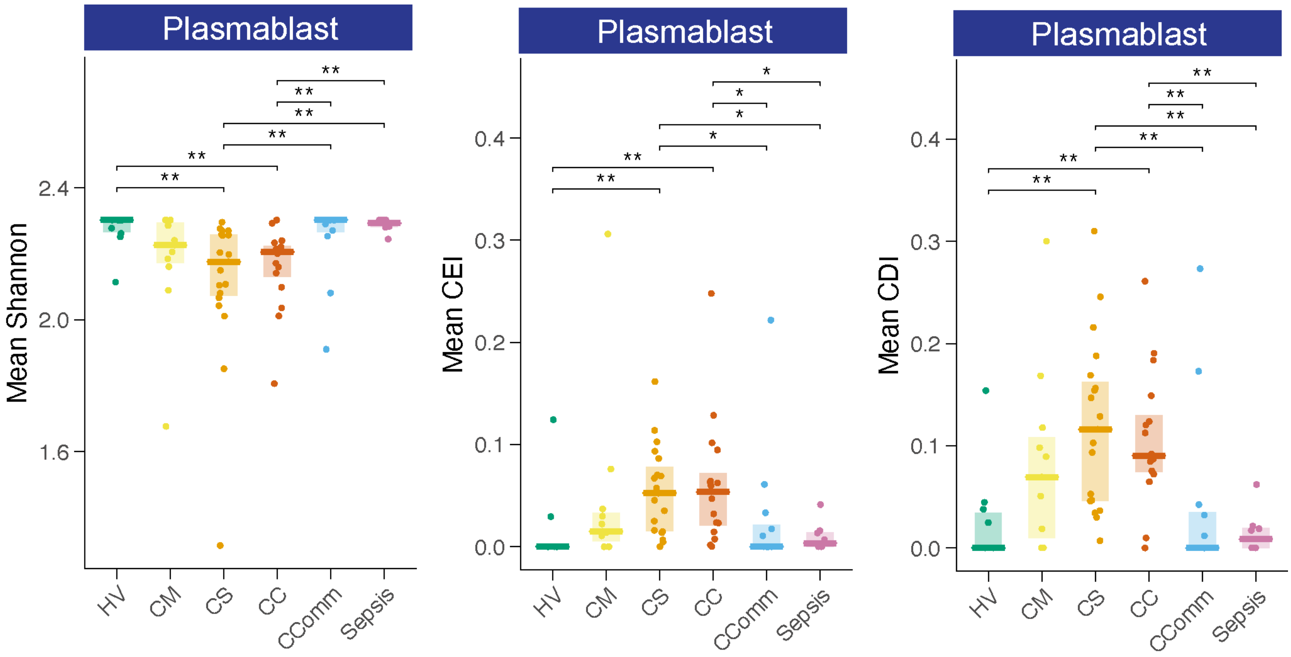

14: Mean plasmablast diversity indices by comparator group; clonal expansion index, CEI, clonal diversification index, CDI. Related to STAR Methods: Repertoire analysis: RNA-velocity B cell analysis.

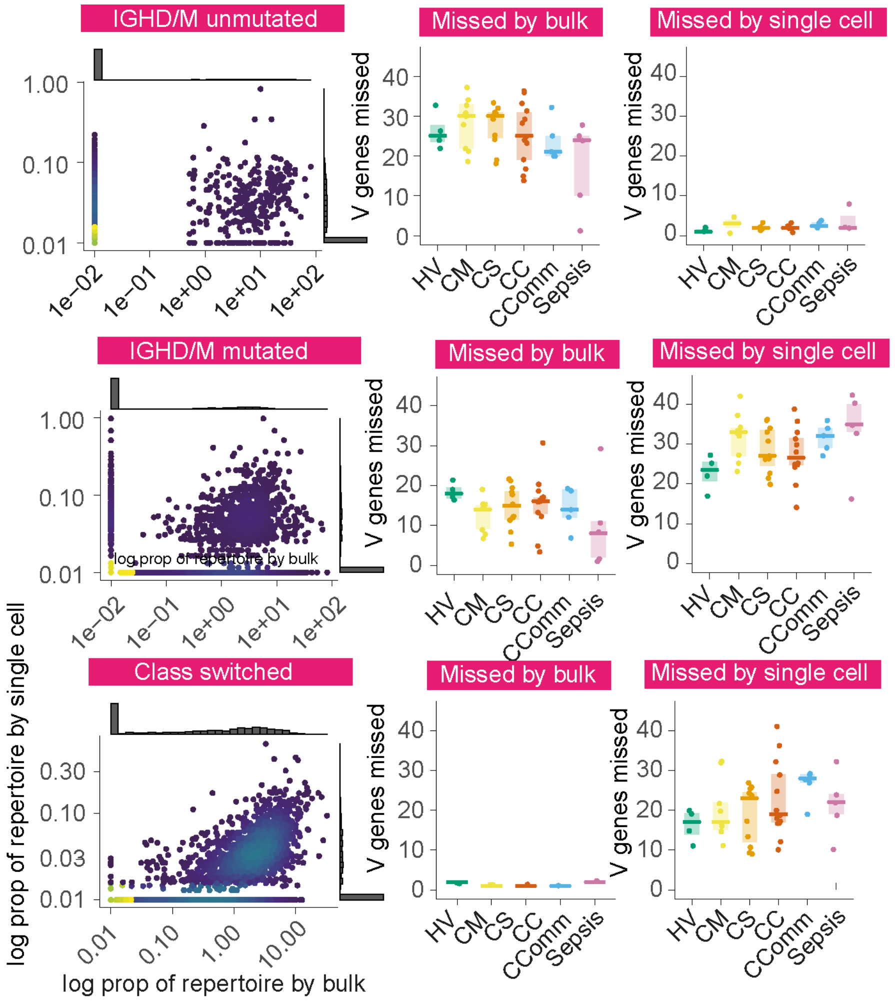

15: Correlation analysis of IGHV genes detected by single cell VDJ data and RNA VDJ data, within the (top) IGHD/M unmutated, (middle) IGHD/M mutated and (bottom) class-switched VDJ sequences. Scatter plots, with marginal histograms, depict log proportions of repertoires detected and missed by both datasets. Boxplots to the right depict number of IGHV genes missed by RNA (bulk) and single cell VDJ data. Related to STAR Methods: Repertoire analysis: RNA-velocity B cell analysis.

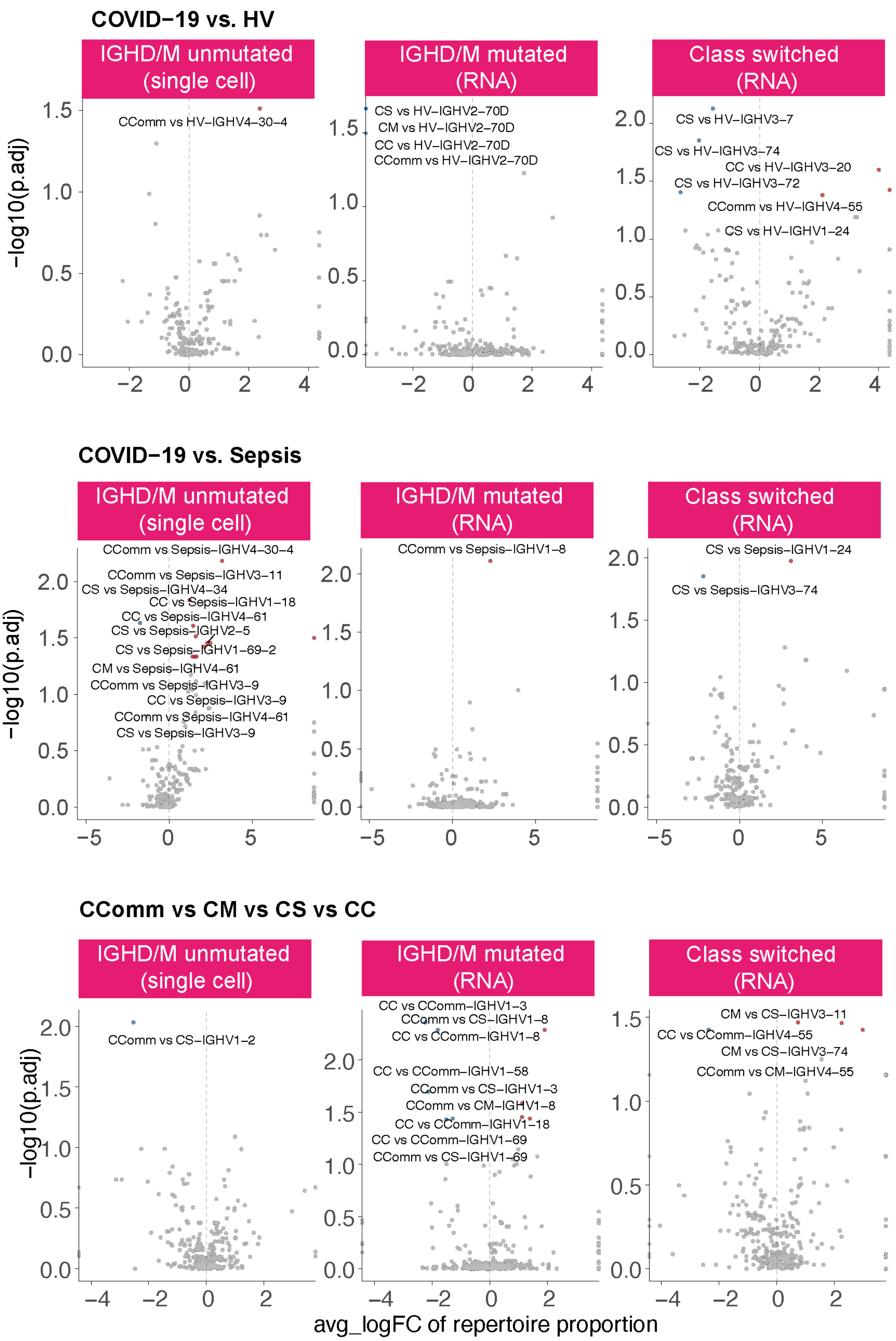

16: Volcano plots of $\log_2$ fold change of IGHV gene usage of IGHD/M unmutated sequences (derived from single cell VDJ data), IGHD/M mutated sequences (derived from RNA VDJ data), class-switched sequences (derived from RNA VDJ data). Top panel depicts significant genes across pairwise comparisons of COVID-19 groups vs. healthy volunteers. Middle panel depicts significant genes across COVID-19 vs sepsis groups. Bottom panel depicts significant genes across COVID-19 severity groups. All significant pairwise comparisons are derived from Dunn tests post Kruskal Wallis testing. Related to STAR Methods: Repertoire analysis: RNA-velocity B cell analysis.

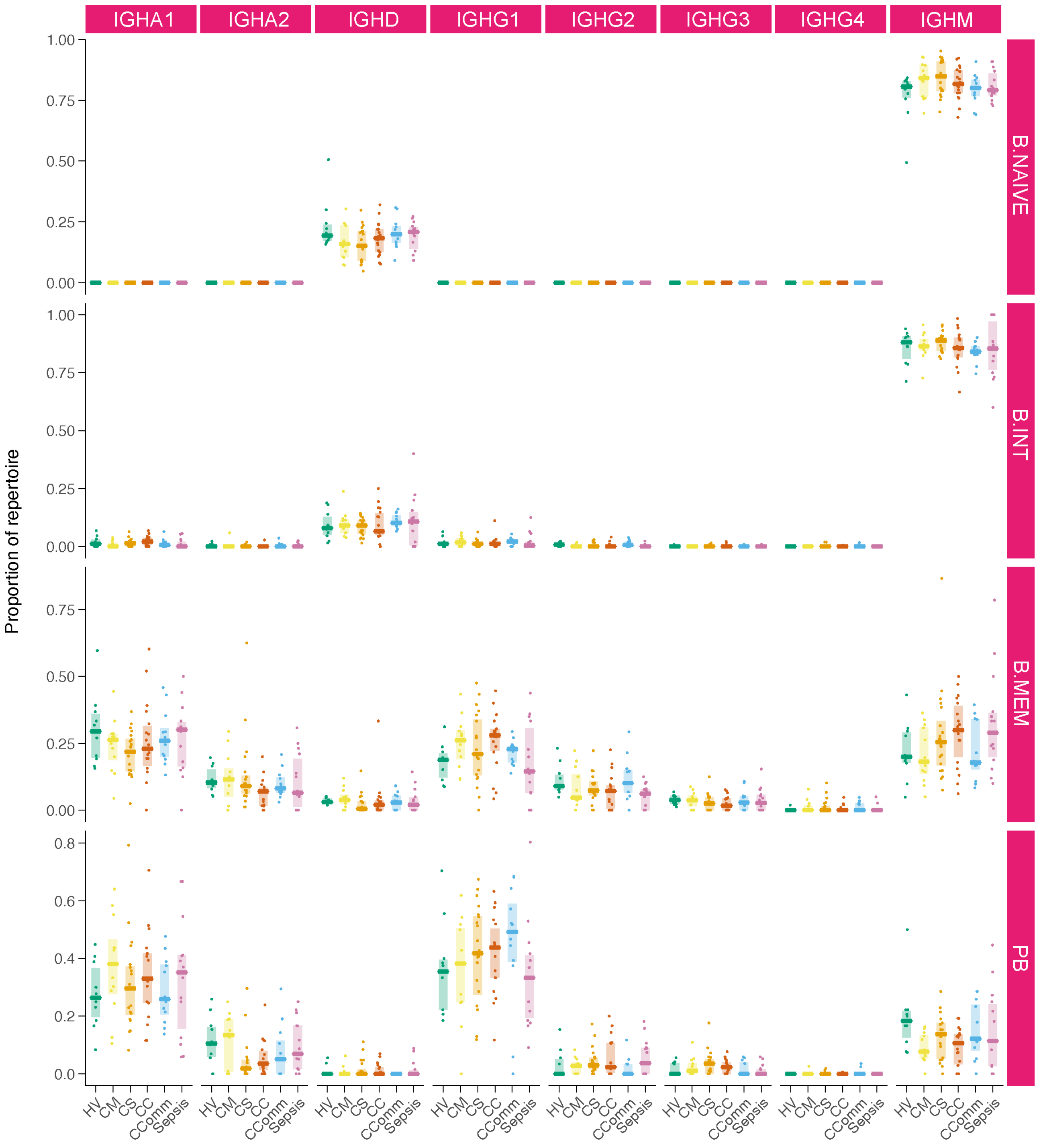

17: Proportion of constant region genes across B cell clusters per study group. Related to STAR Methods: Repertoire analysis: RNA-velocity B cell analysis.

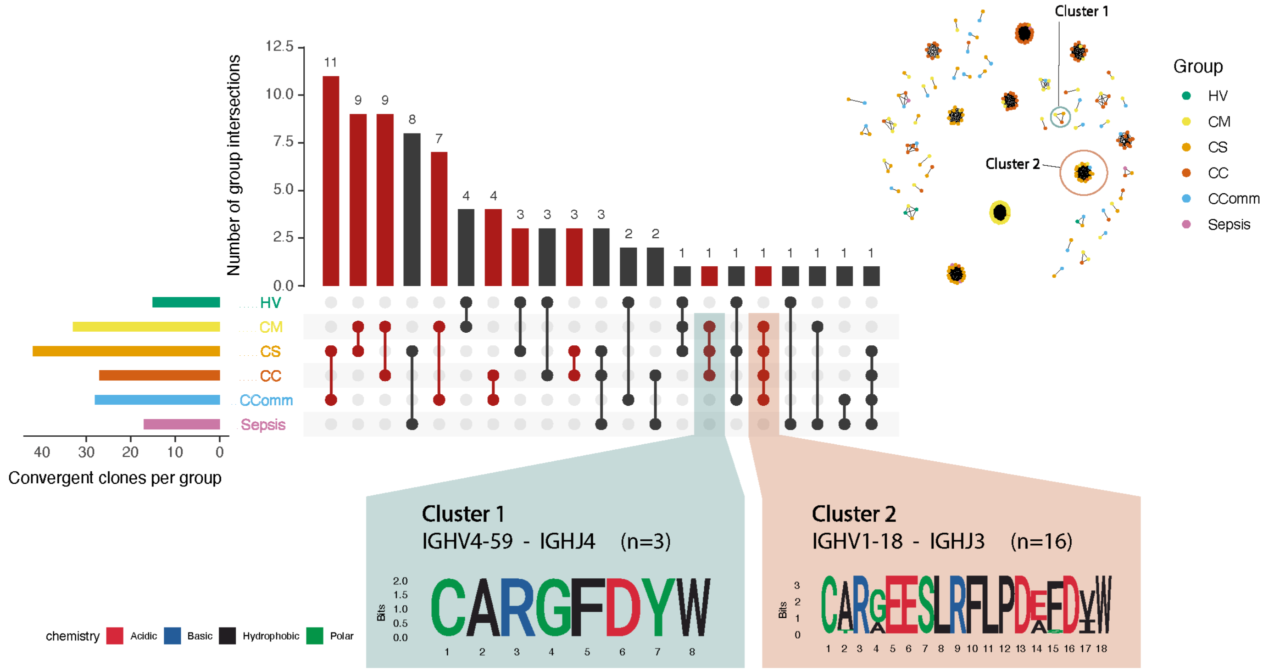

18: Clonal overlap across comparator groups with total number of convergent clones per group shown with clonal network depicting distribution of convergent clusters (inset) and sequence logos of COVID-19 exclusive clusters (with IGHV/J usage and number of members). Related to STAR Methods: Repertoire analysis: RNA-velocity B cell analysis.

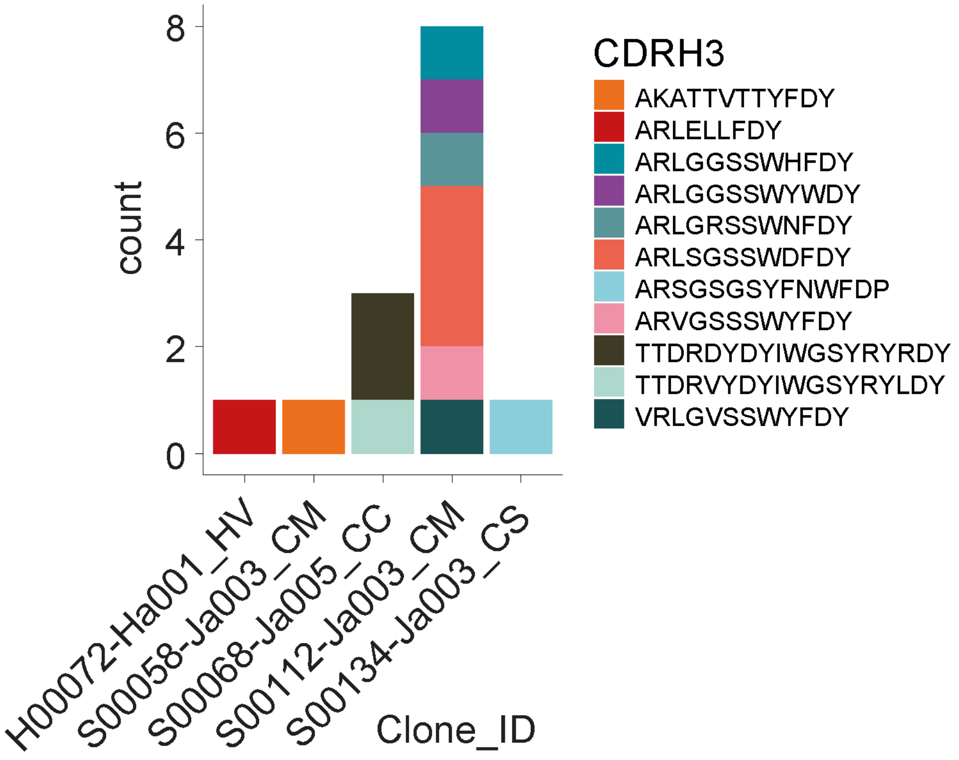

19: Cumulative bar chart of the frequencies of known SARS-CoV-2 binding BCRs per patient group. Each section represents an individual patient. Related to STAR Methods: Repertoire analysis: RNA-velocity B cell analysis.

Proteomics

Clustering of surface protein (ADT) data

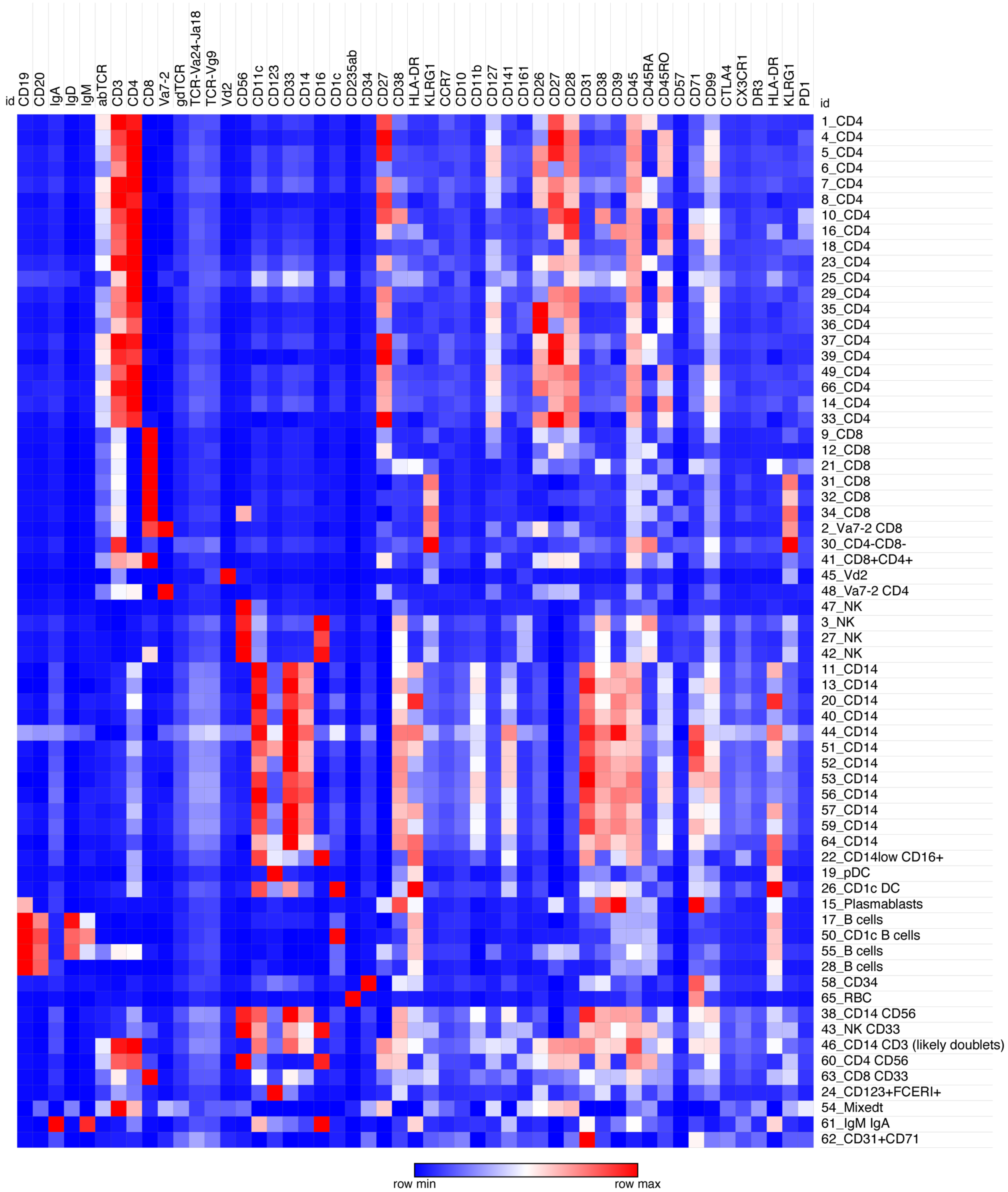

20: Clustering of surface protein (ADT) data. Initial cytosplore clustering of the CITE-seq datasaet by ADT surface phenotype based on n=192 ADT tags. Columns: a selection of informative surface proteins (top labels), rows: Cytosplore cluster numbers and assigned phenotypes (right labels). Related to Figures 1B,C; STAR Methods: CITE-seq: Pre-processing and analysis of the surface protein (ADT) data; and Data S5: Multimodal annotation of CITE-seq data.

Plasma and serum proteins assayed by Luminex

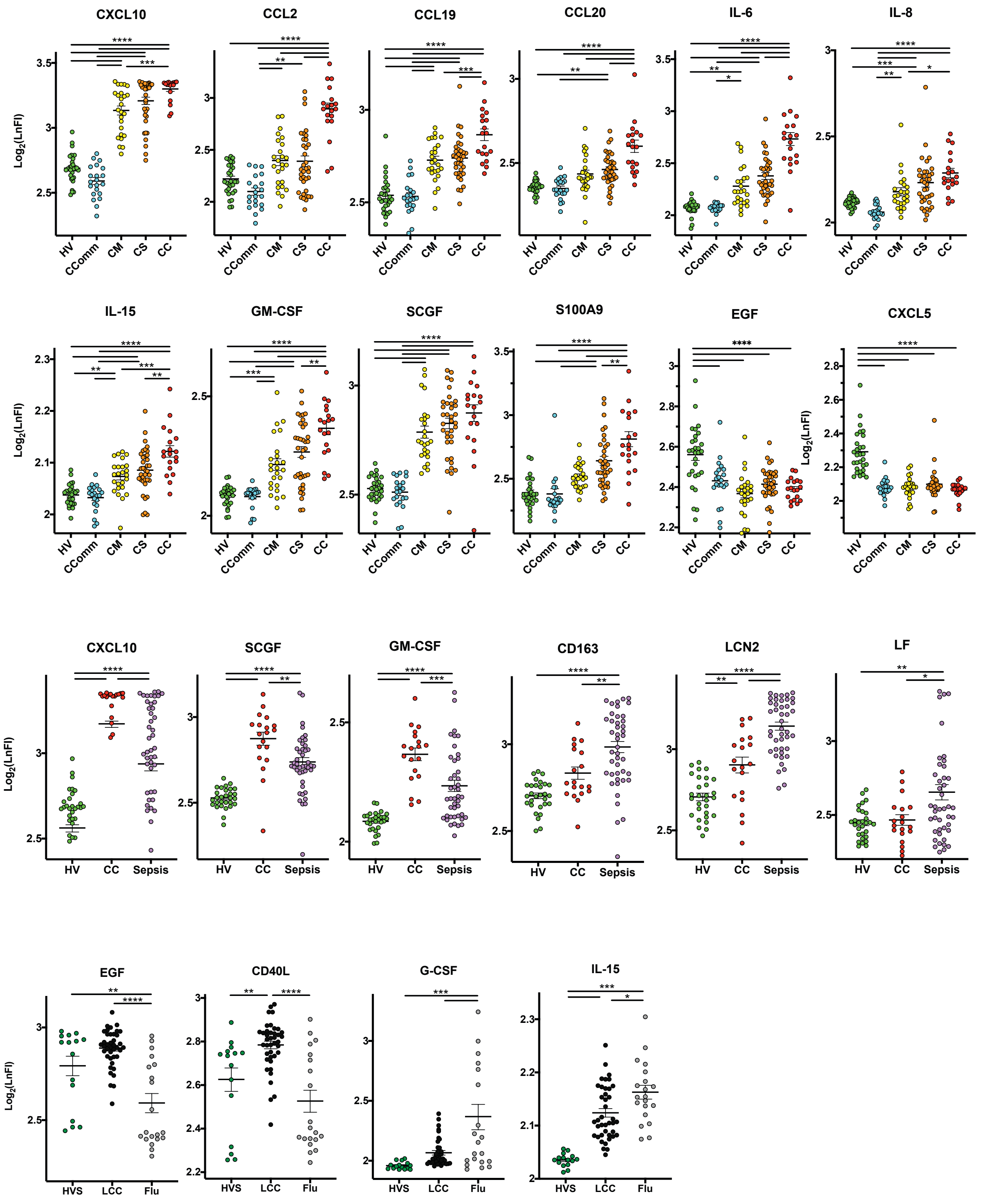

21: Plasma protein abundance for specific proteins assayed by Luminex assay. Individual protein abundance across the comparator groups shown. Related to STAR Methods: Luminex data analysis: Cytokine enrichment profile analysis.

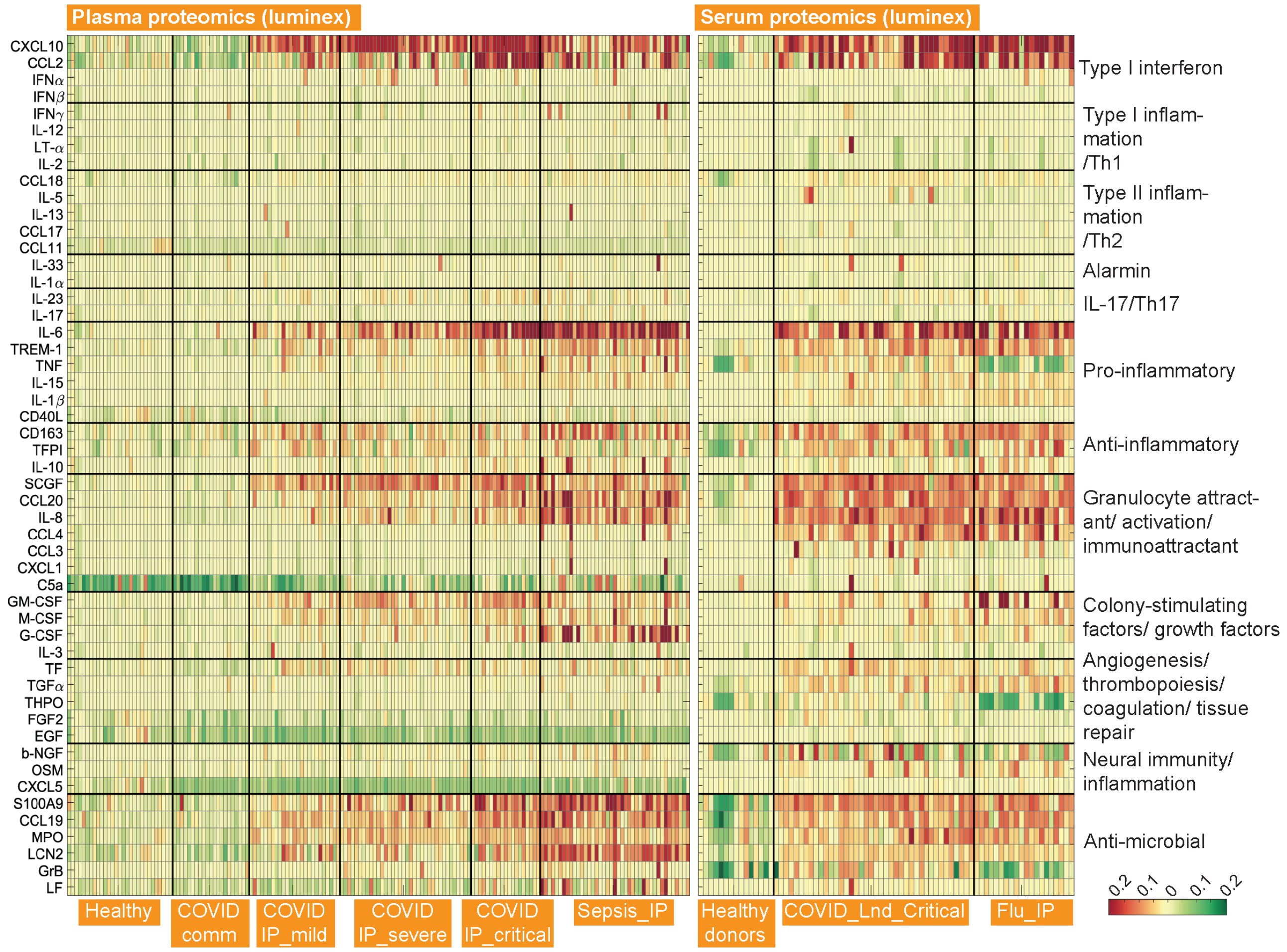

22: Heat map showing abundance of plasma and serum proteins by disease groups assayed using Luminex. Related to STAR Methods: Luminex data analysis: heatmap.

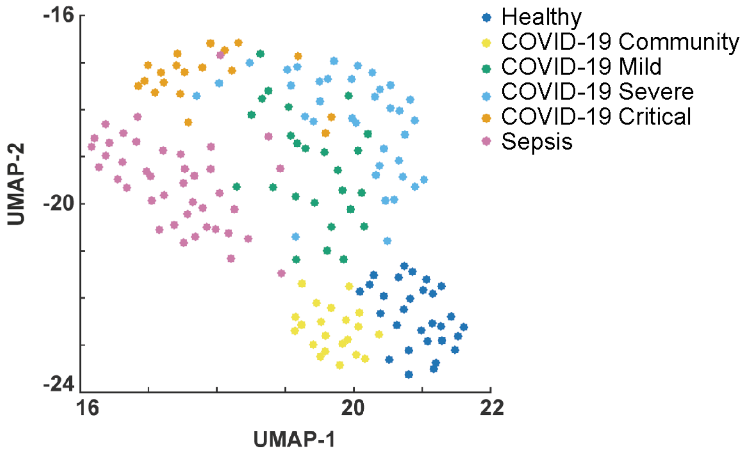

23: UMAP of plasma proteins assayed by Luminex assay, colored by comparator group. Related to STAR Methods: Luminex data analysis: uniform manifold projection.

Plasma proteins assayed by HT-LC-MS/MS mass spectrometry

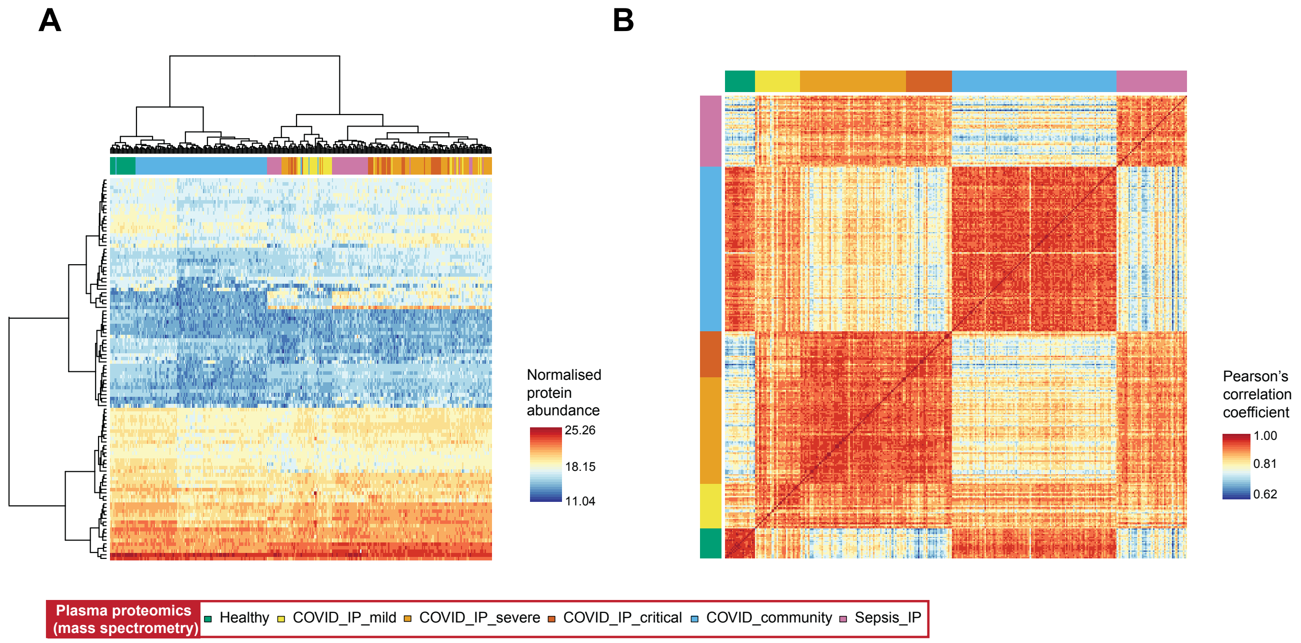

24: Unsupervised hierarchical clustering and supervised correlation analysis of plasma proteins assayed by HT-LC-MS/MS mass spectrometry. (A) Unsupervised clustering analysis all samples (B) supervised correlation analysis all samples. Related to STAR Methods: Tims-TOF mass spectrometry analysis: statistical analysis.

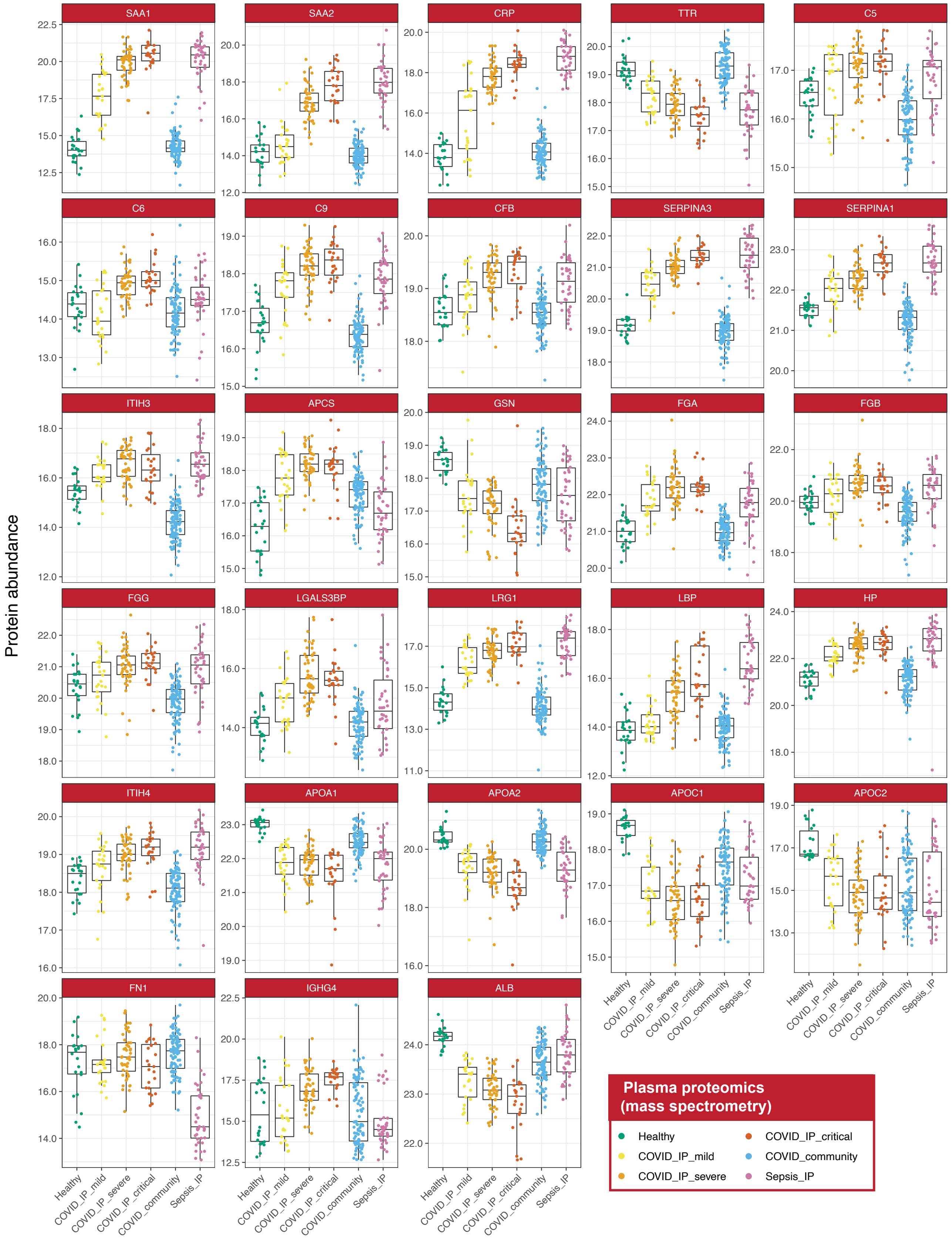

25: Plasma protein abundance for specific proteins assayed by HT-LC-MS/MS mass spectrometry. Individual protein abundance across the comparator groups shown. Only one sample per patient at the maximal severity are plotted. Related to STAR Methods: Tims-TOF mass spectrometry analysis.
