# Supplementary material for: A blood atlas of COVID-19 defines hallmarks of disease severity and specificity
Source: Cell. 2022 Mar 3;185(5):916–938.e58. doi: 10.1016/j.cell.2022.01.012 (PMC8776501; doi:10.1016/j.cell.2022.01.012)
Supplement: Data S4: Multimodal annotation of CITE-seq data, related to STAR Methods, Figure 1, and Table S2 [file mmc8.pdf]

Data S4: Multimodal Annotation of CITE-seq Data, Related to STAR Methods, Figure 1B,C and Table S2

Related to **Figure 1B,C** and the **STAR Methods**: CITE-seq: multimodal cluster annotation. Supporting GEX and ADT clustering analyses are shown in **Figure S1E** and **Data S3: Analysis of Mass and Flow Cytometry, Repertoire and Proteomics, Related to STAR Methods**. The multimodal definitions of the lymphoid cell populations are given in **Table S2: Definition of lymphoid clusters for CITE-seq data**.

Table of Contents

T and NK cell populations....2
- CD4 and CD8 T cell sub-populations 2
  - 1: Dot plot of CD4 T-cell sub-populations 2
  - 2: Dot plot of CD8 T-cell sub-populations 3
- Double positive and negative T cell sub-populations.... 4
  - 3: Dot plot of double positive (DP) T-cell populations 4
  - 4: Dot plot of double negative (DN) T-cell populations 5
- Mucosal associated invariant T (MAIT) cell sub-populations 6
  - 5: Dot plot of MAIT cell sub-populations 6
- Gamma delta T cell sub-populations 7
  - 6: Dot plot of Vδ2+ve γδ T-cell sub-populations. 7
  - 7: Dot plot of Vδ2-ve Vγ9-ve γδ T-cell sub-populations.... 8
  - 8: Dot plot of Vδ2-ve Vγ9+ve γδ T-cell sub-populations. 9
- Invariant natural killer T (iNKT) sub-populations 10
  - 9: Dot plot of iNKT cells.... 10
- Natural Killer (NK) cell sub-populations 11
  - 10: Dot plot of NK cell sub-populations. 11

B cell and plasmablast sub-population phenotypes....12
  - 11: Dot plot of B cell and plasmablast sub-populations.... 12

Mononuclear phagocyte (MNP) sub-populations 13
  - 12: Dot plot of MNP cell sub-populations 13

Hematopoietic stem (and progenitor) cell sub-populations....14
  - 13: Dot plot of hematopoietic stem (and progenitor) cell sub-populations 14

Platelet and erythrocyte sub-populations....15
  - 14: Dot plot of platelet and erythrocyte cell sub-populations. 15

T and NK cell populations

The multimodal definition of the major T and NK cell populations is given in **Table S2: Definition of lymphoid clusters for CITE-seq data**.

CD4 and CD8 T cell sub-populations

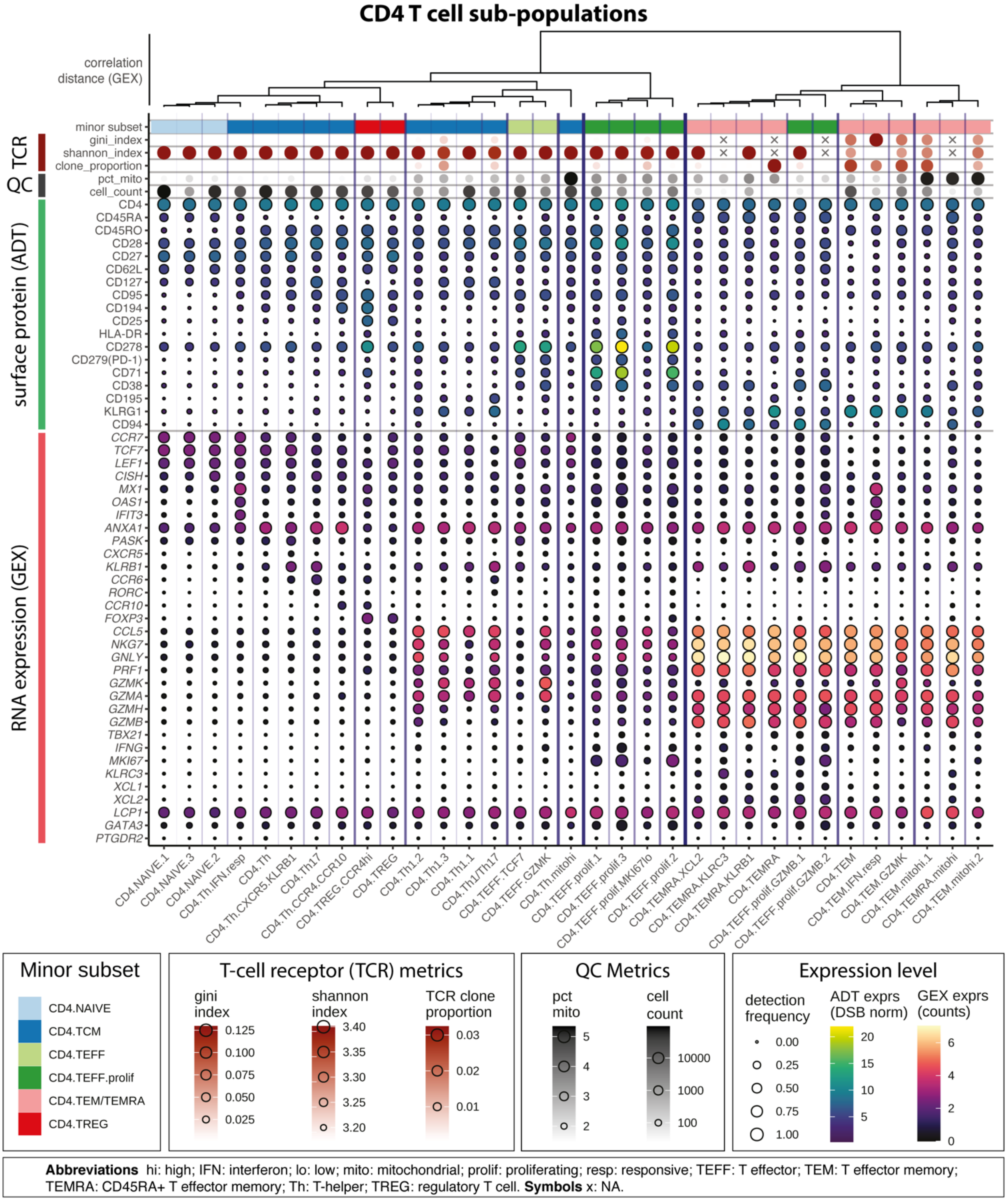

1: Dot plot of CD4 T-cell sub-populations.

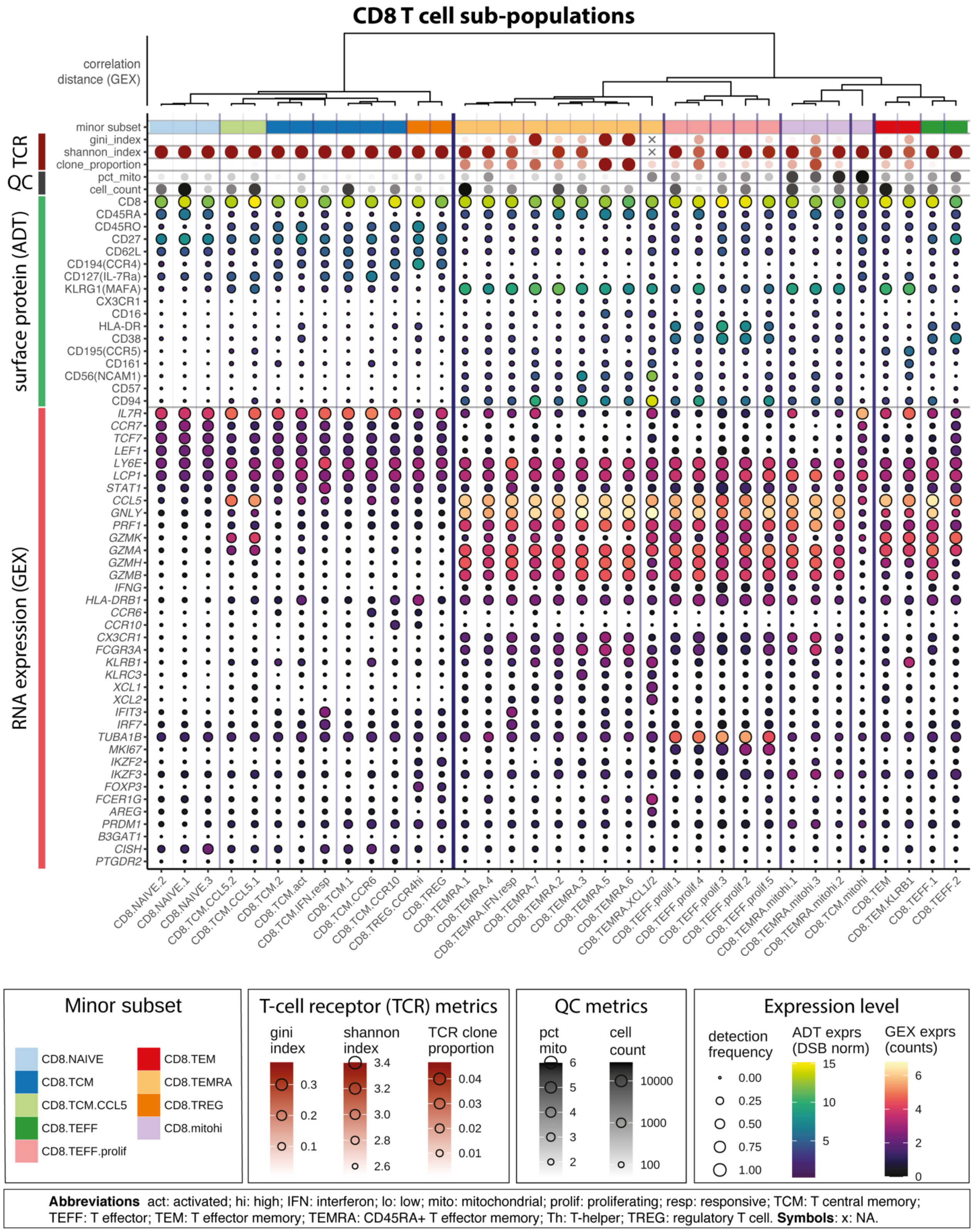

2: Dot plot of CD8 T-cell sub-populations.

Double positive and negative T cell sub-populations

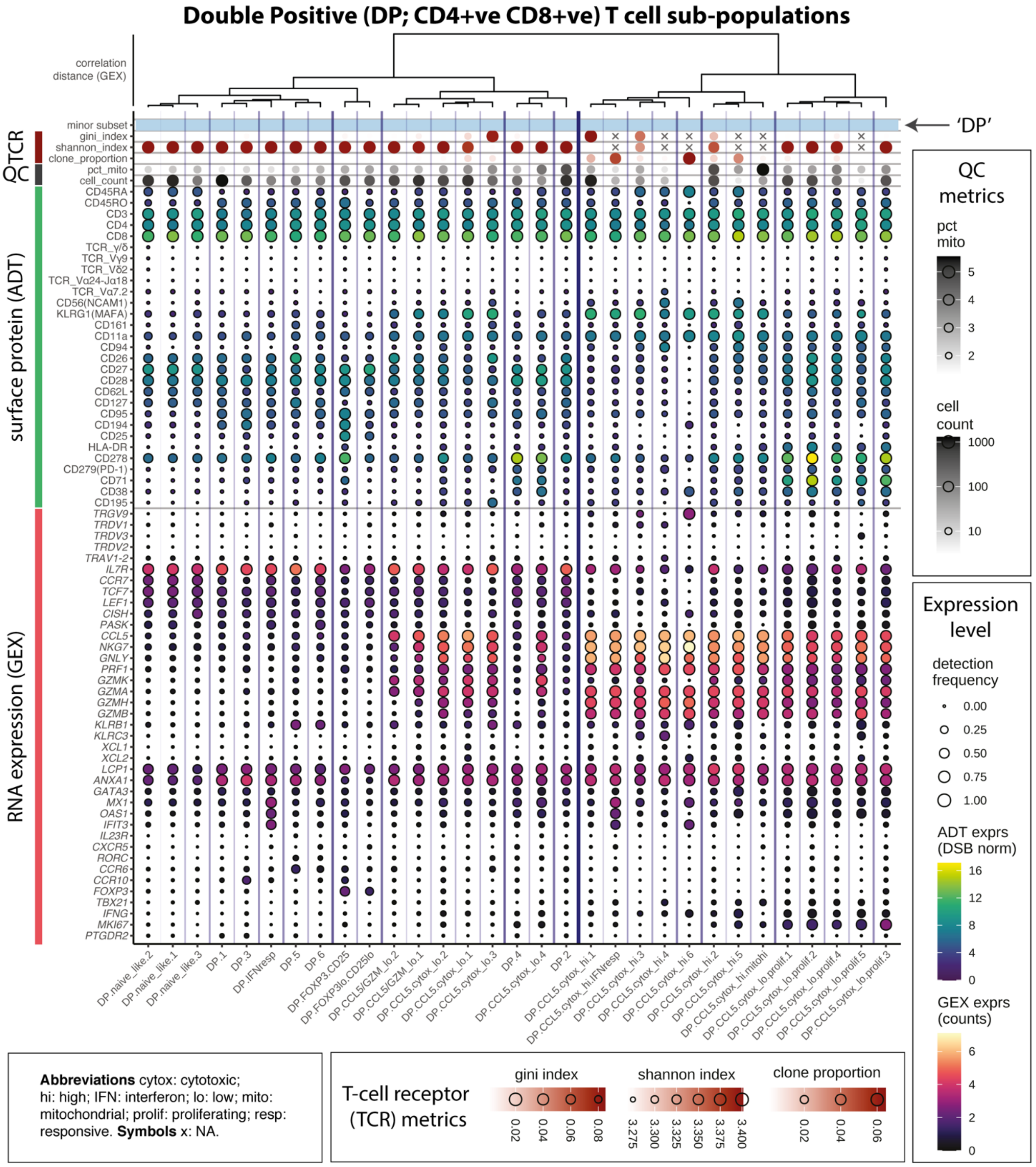

3: Dot plot of double positive (DP) T-cell populations.

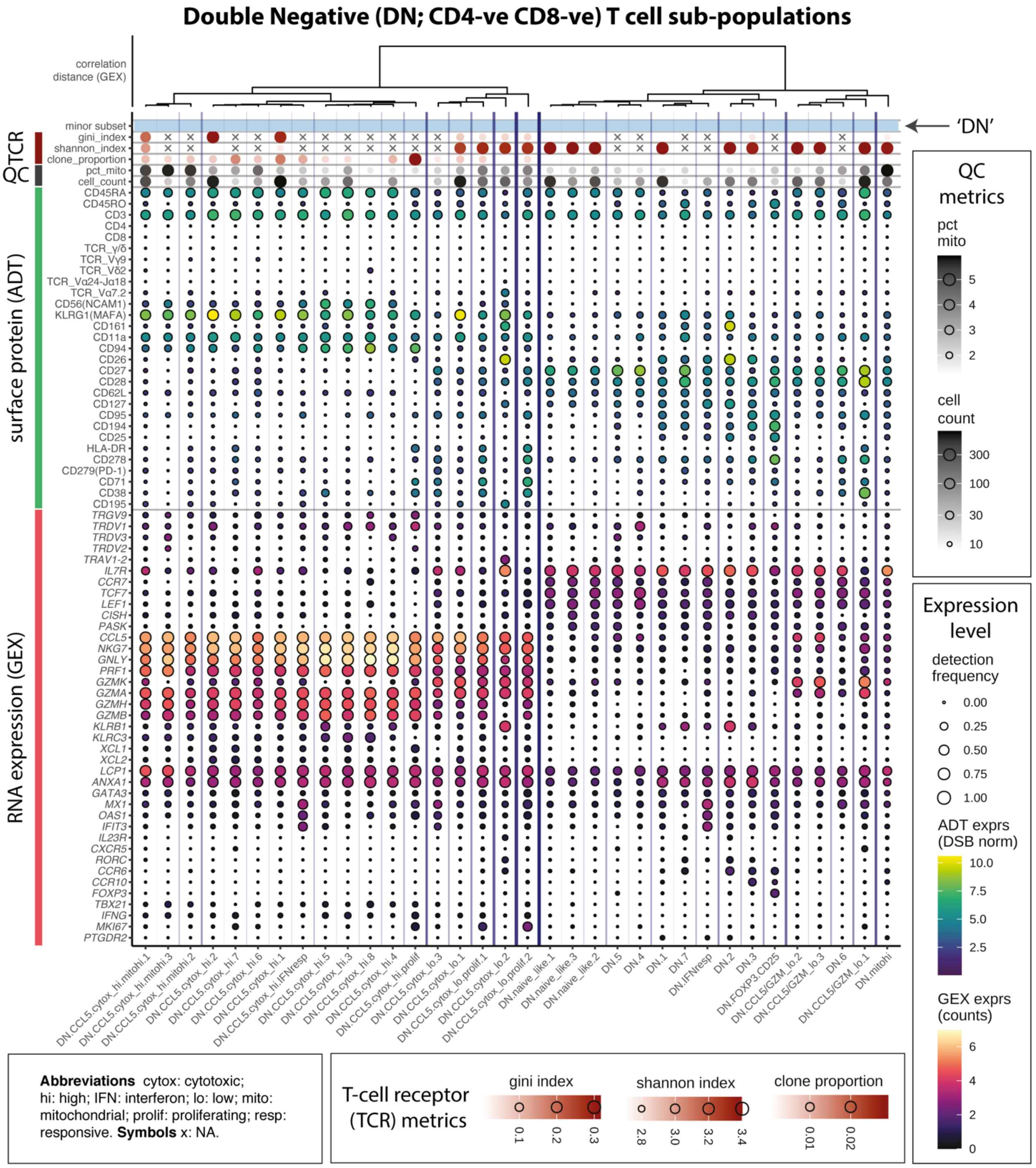

4: Dot plot of double negative (DN) T-cell populations.

Mucosal associated invariant T (MAIT) cell sub-populations

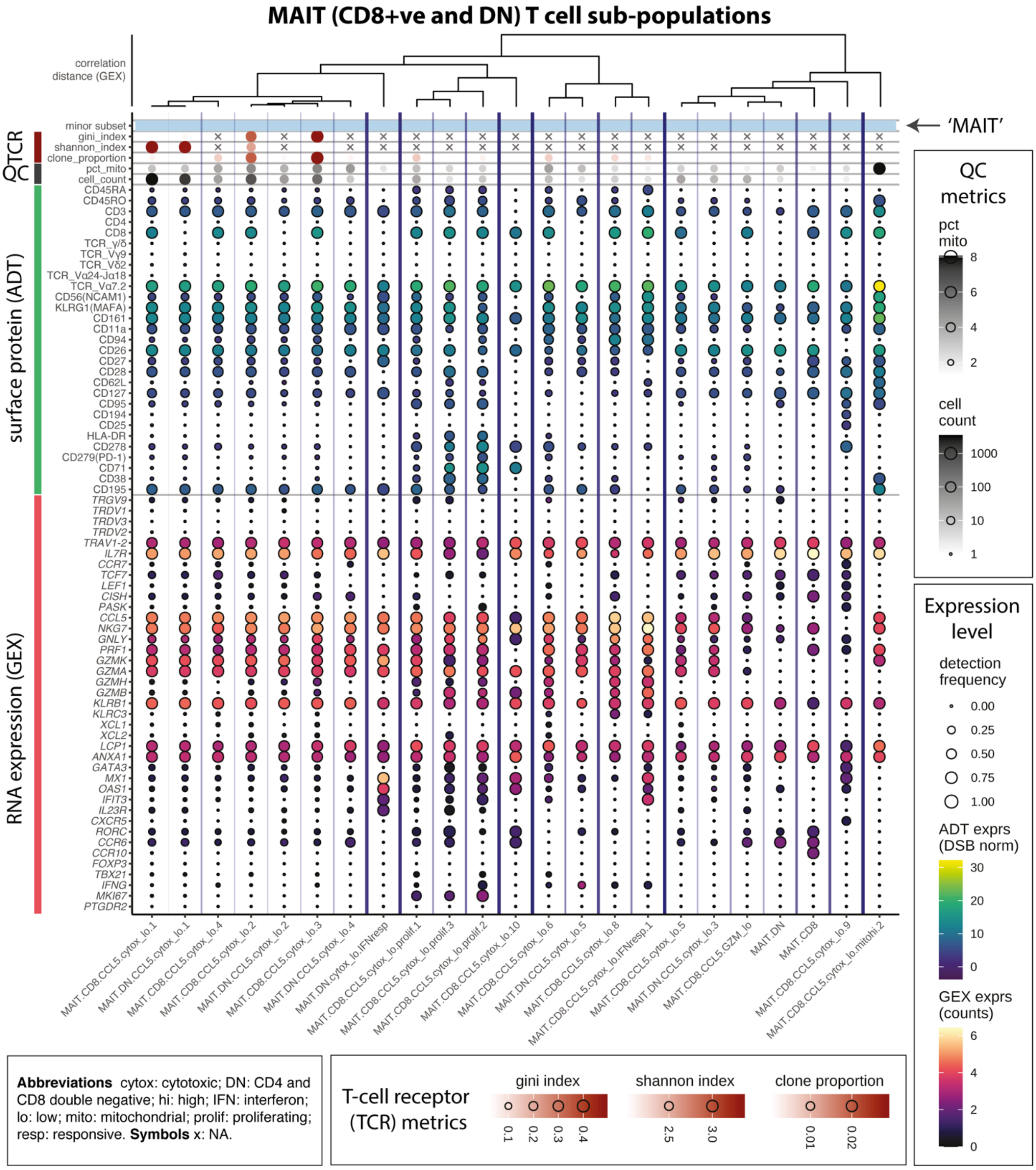

5: Dot plot of MAIT cell sub-populations.

Gamma delta T cell sub-populations

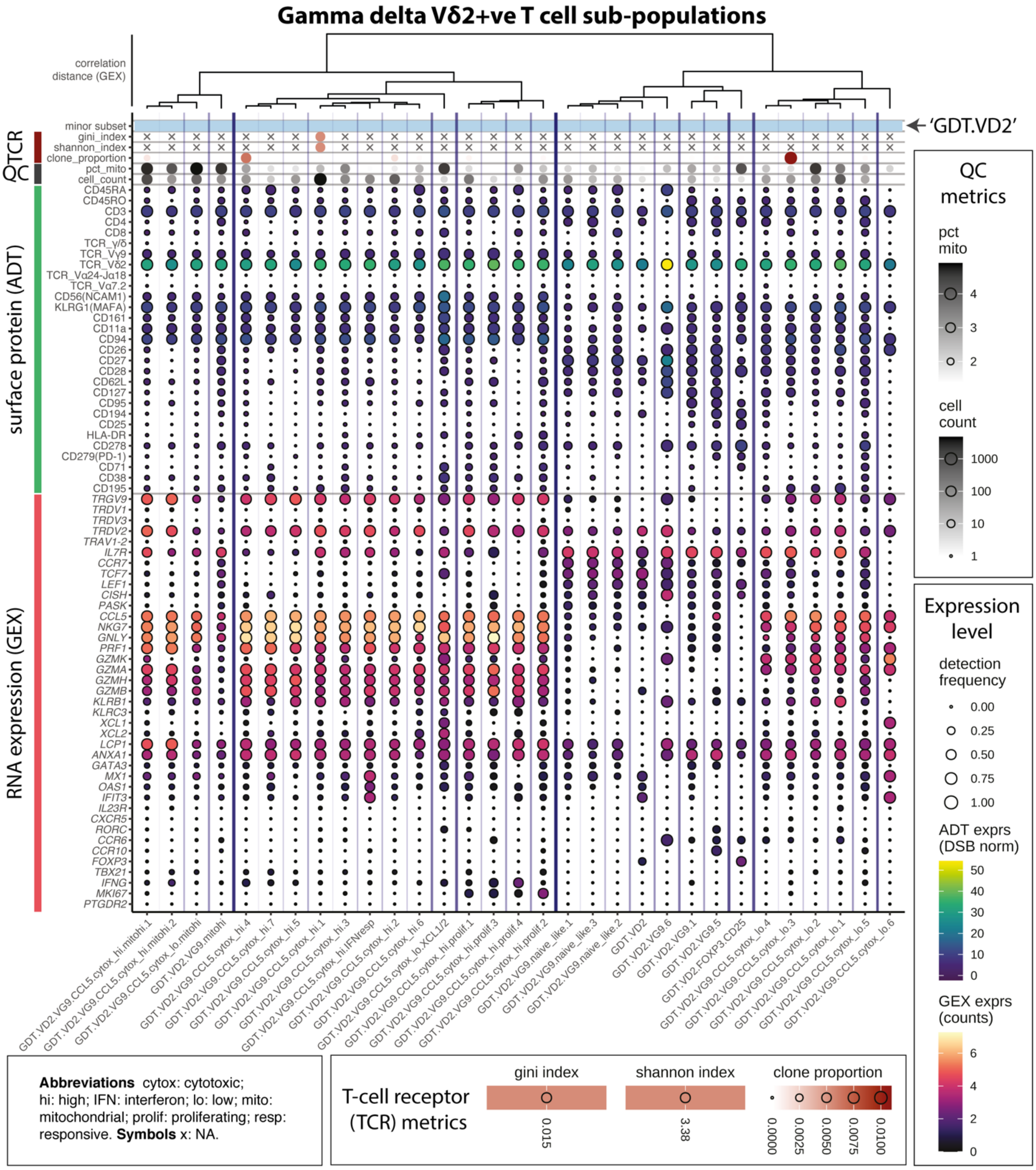

6: Dot plot of Vδ2+ve γδ T-cell sub-populations.

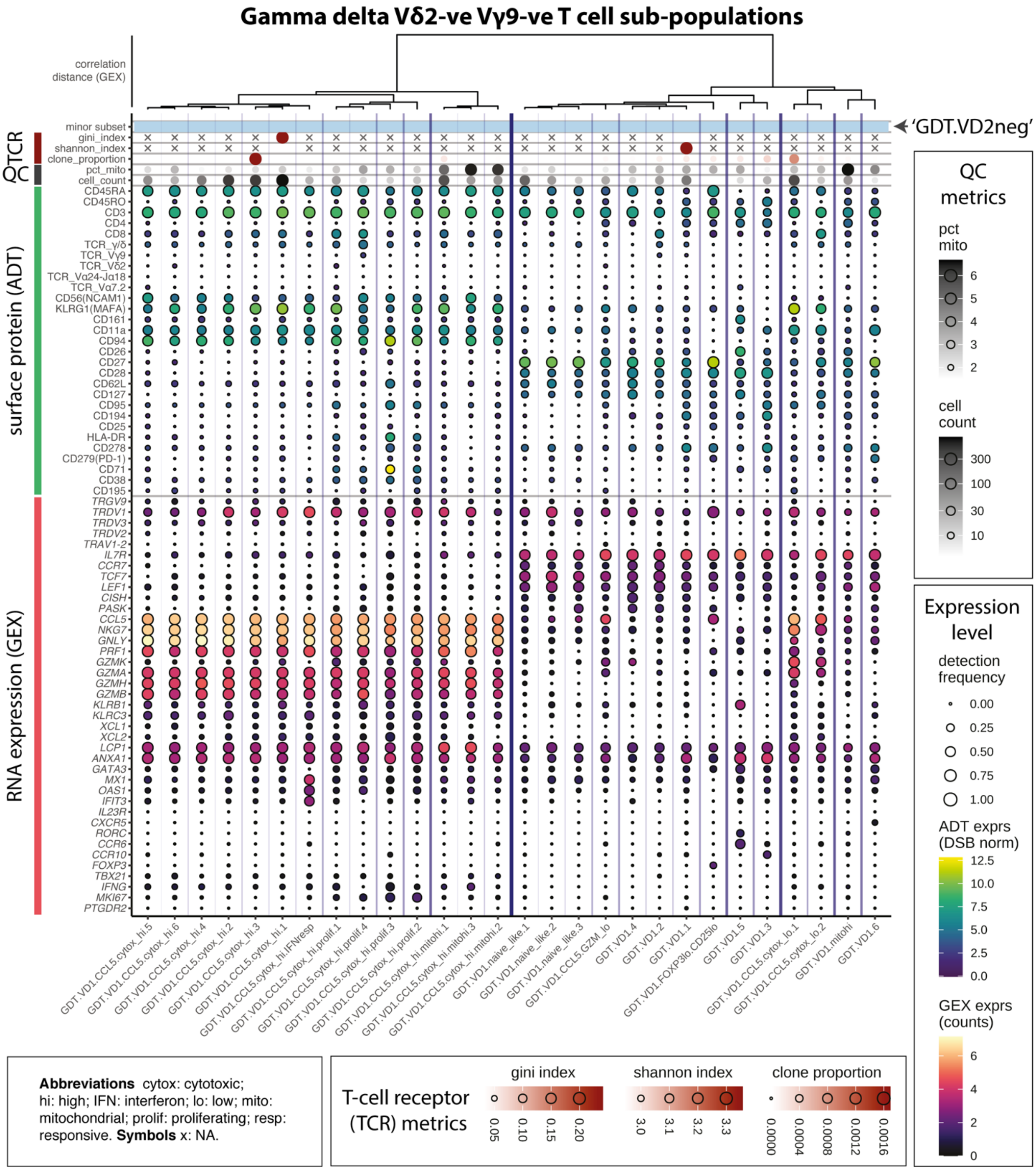

7: Dot plot of Vδ2-ve Vγ9-ve γδ T-cell sub-populations.

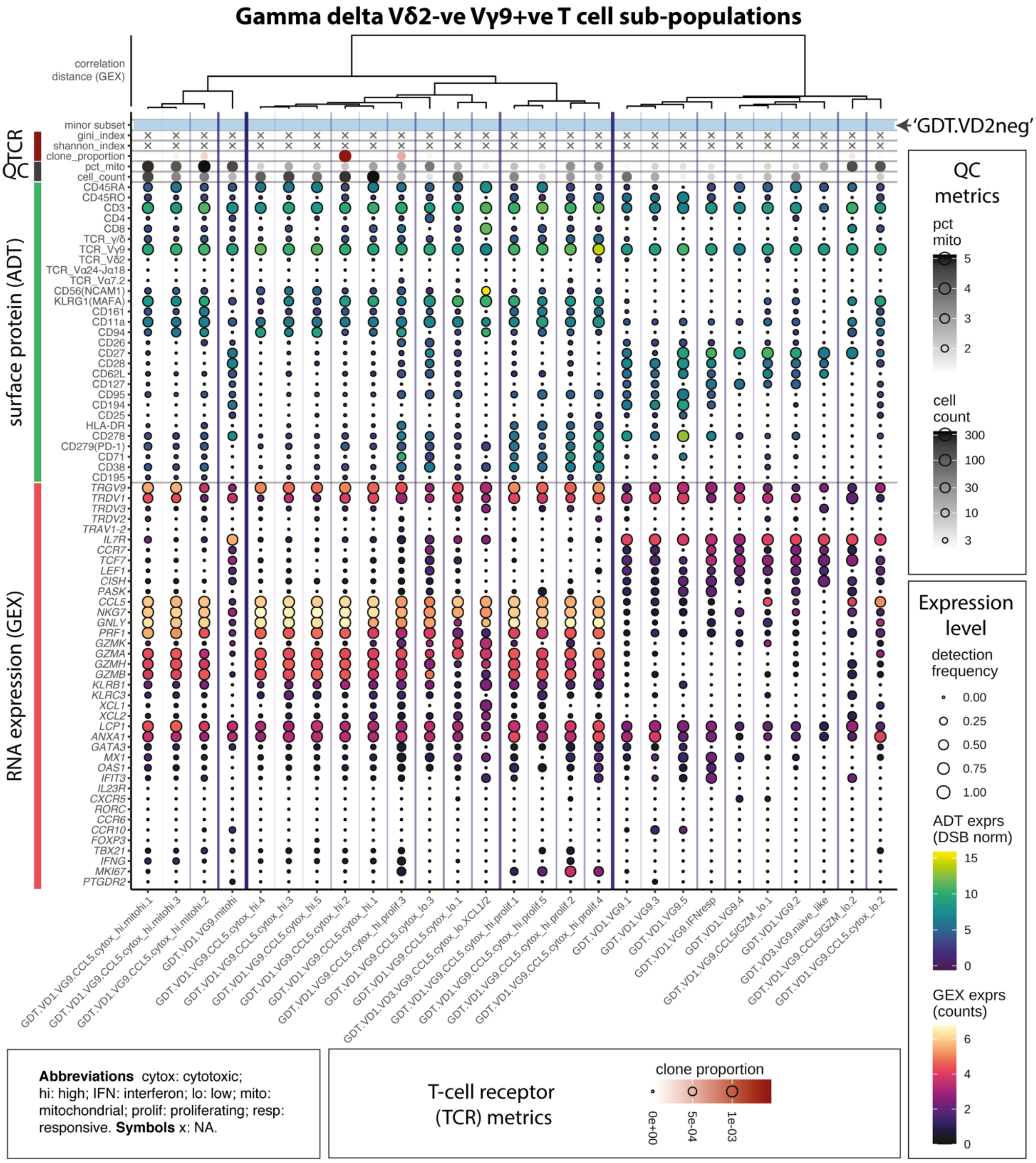

8: Dot plot of Vδ2-ve Vγ9+ve γδ T-cell sub-populations.

Invariant natural killer T (iNKT) sub-populations

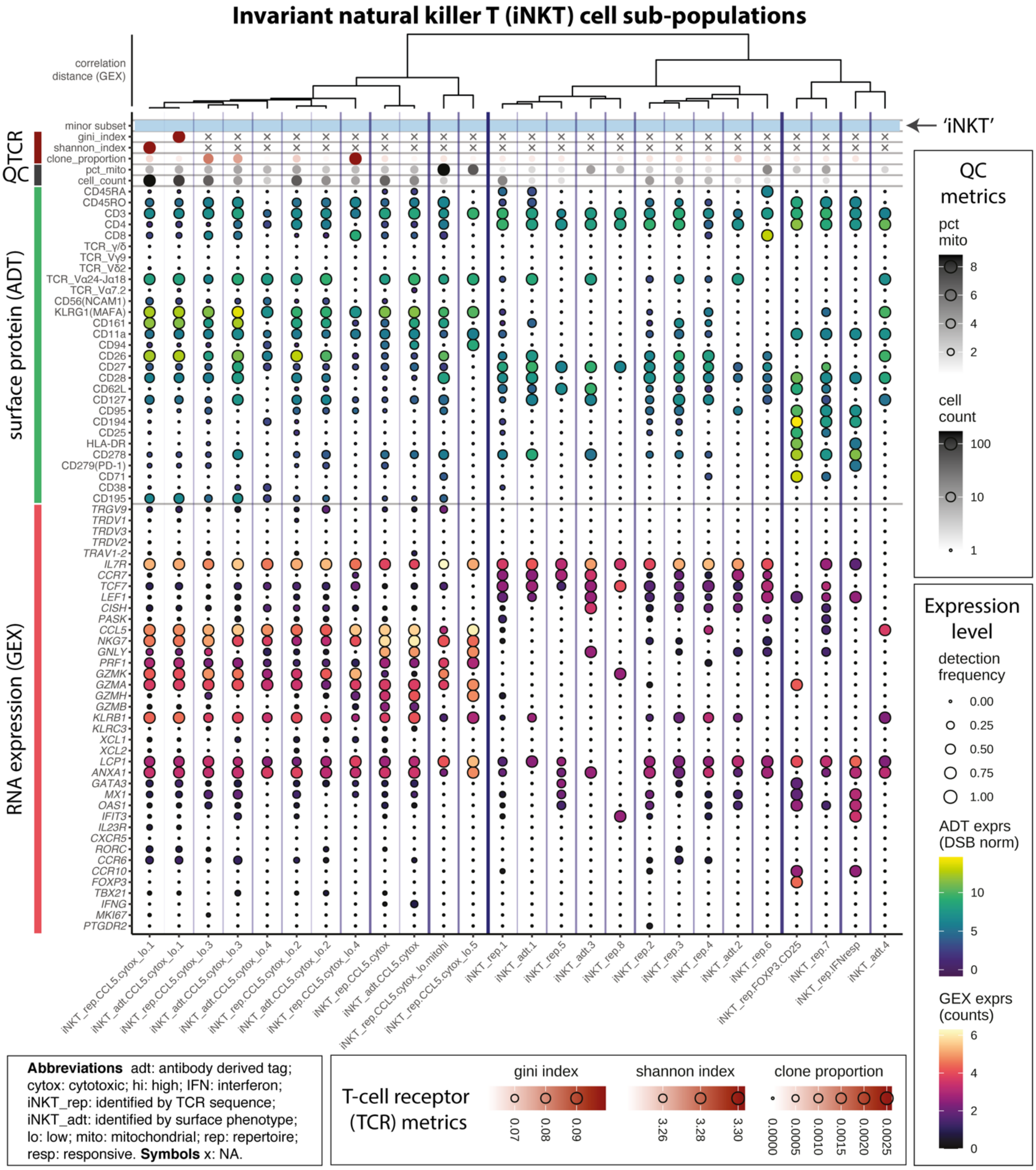

9: Dot plot of iNKT cells.

Natural Killer (NK) cell sub-populations

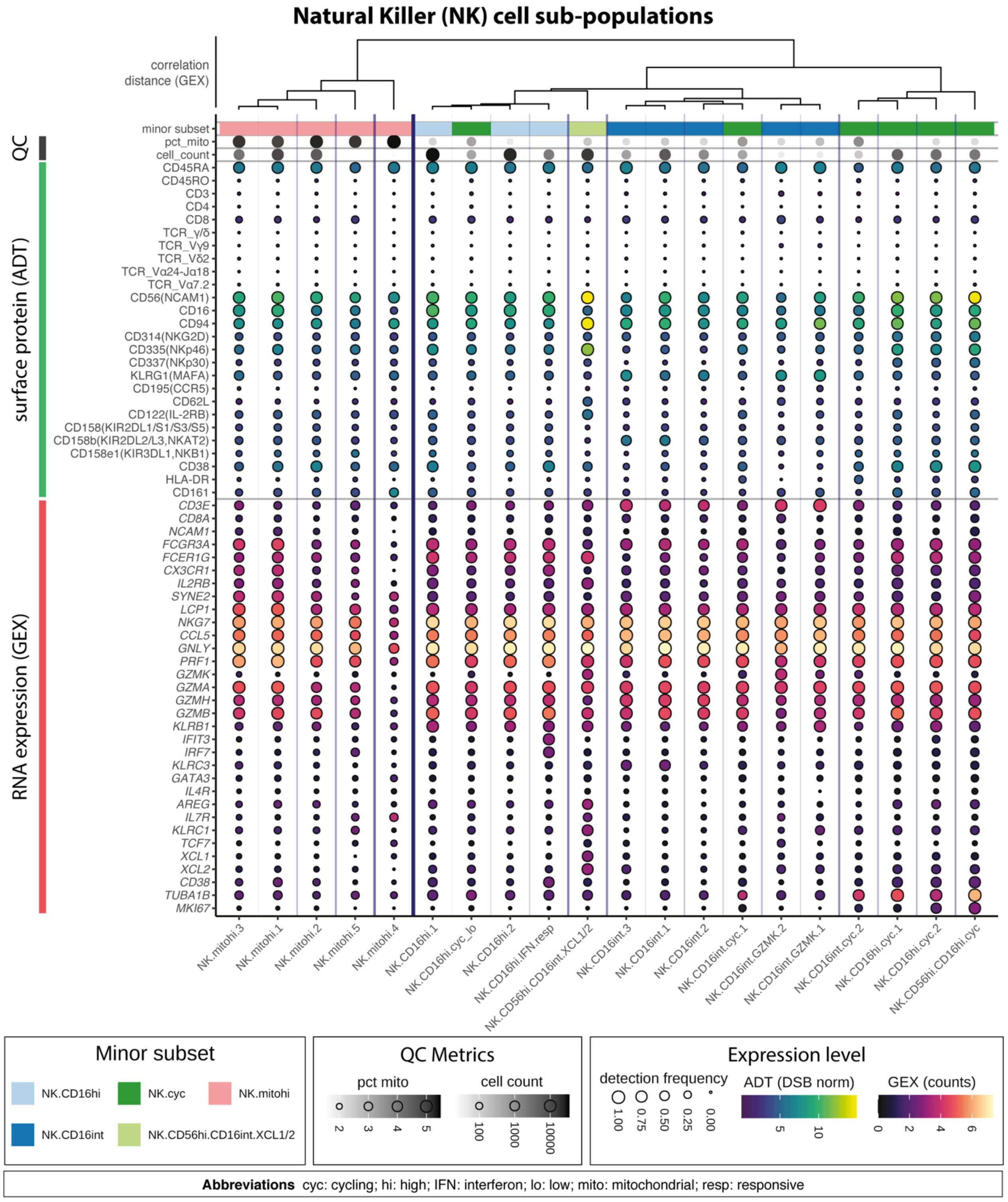

10: Dot plot of NK cell sub-populations.

B cell and plasmablast sub-population phenotypes

The multimodal definition of the B cell and plasmablast populations are given in **Table S2: Definition of lymphoid clusters for CITE-seq data**.

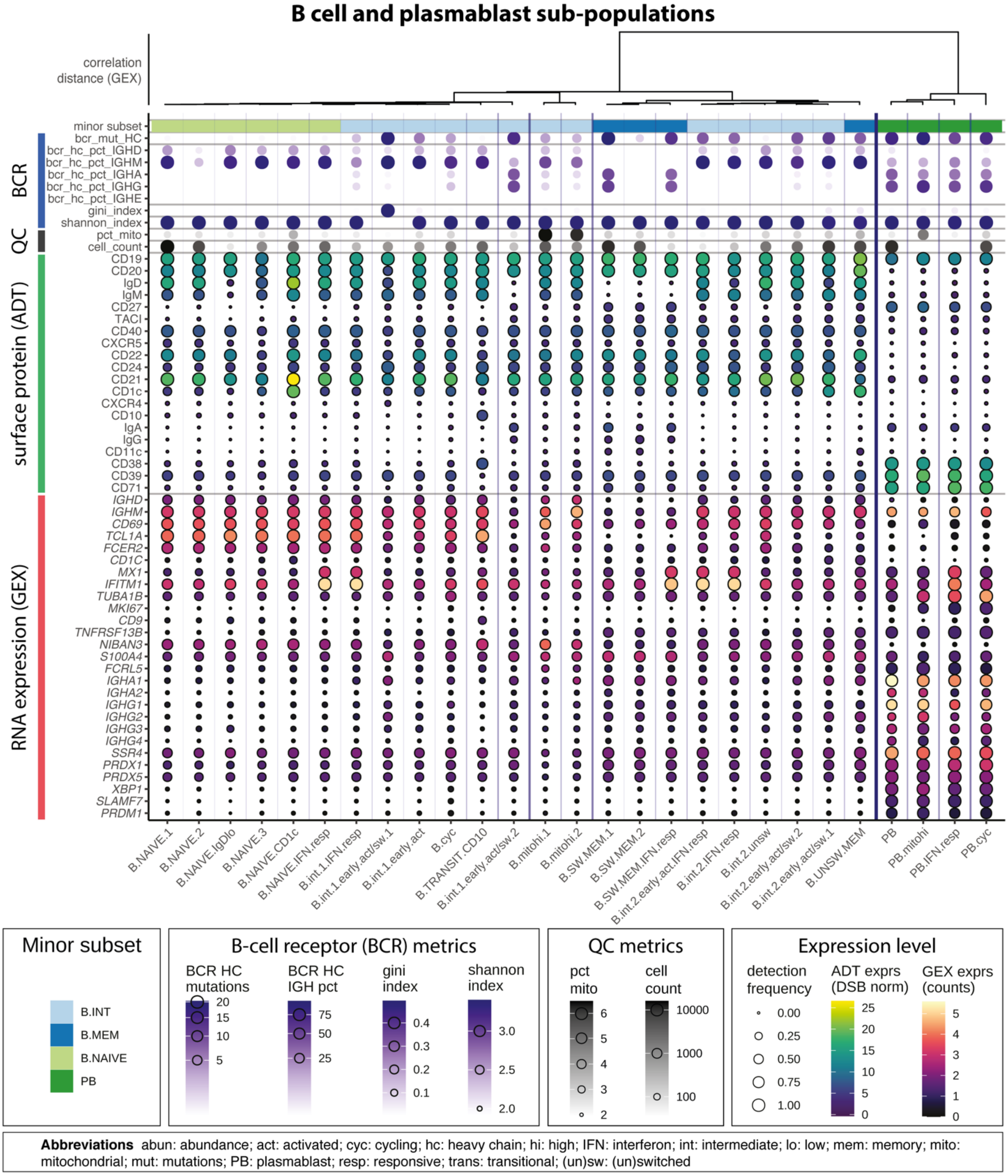

11: Dot plot of B cell and plasmablast sub-populations.

Mononuclear phagocyte (MNP) sub-populations

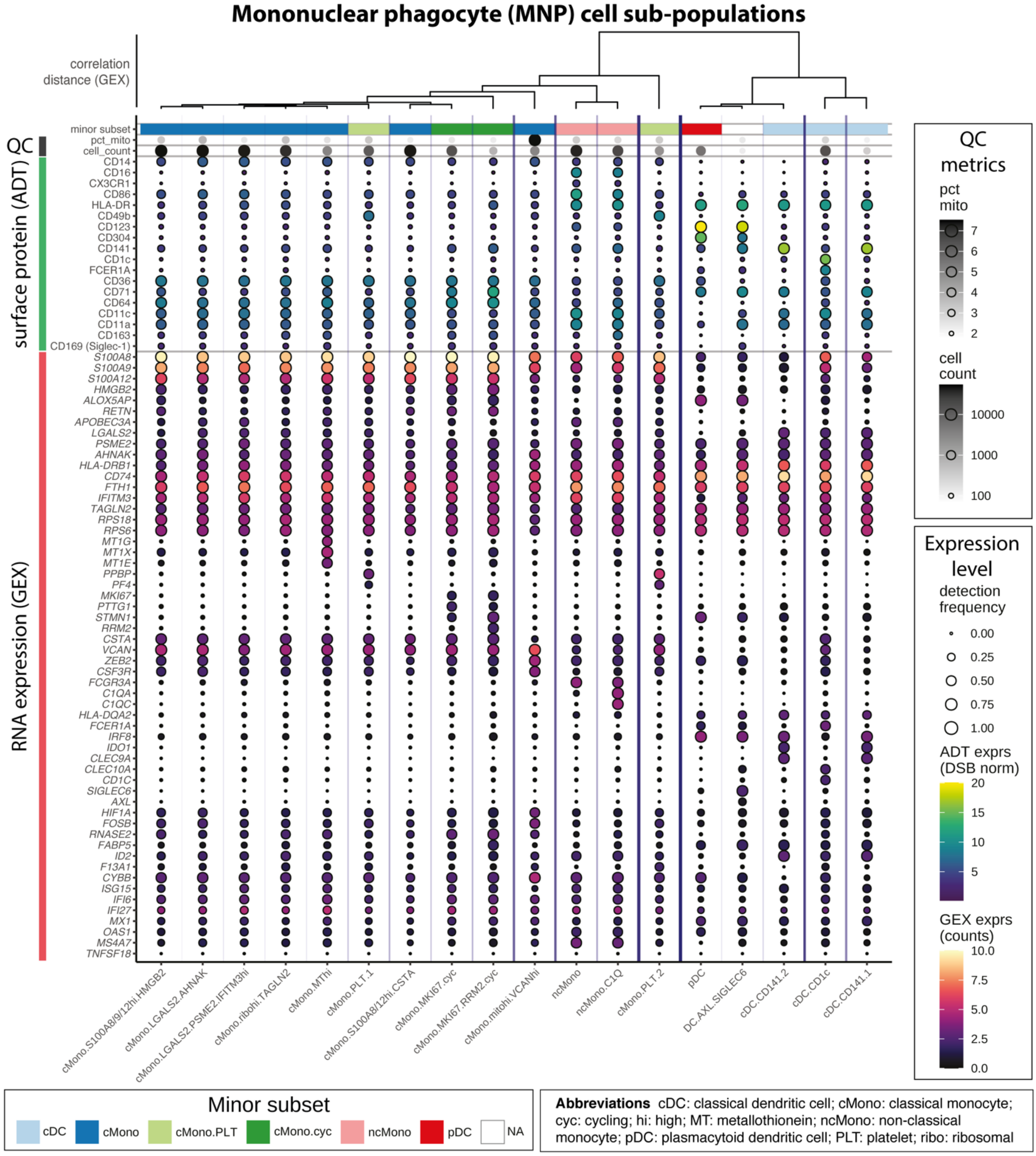

12: Dot plot of MNP cell sub-populations.

Hematopoietic stem (and progenitor) cell sub-populations

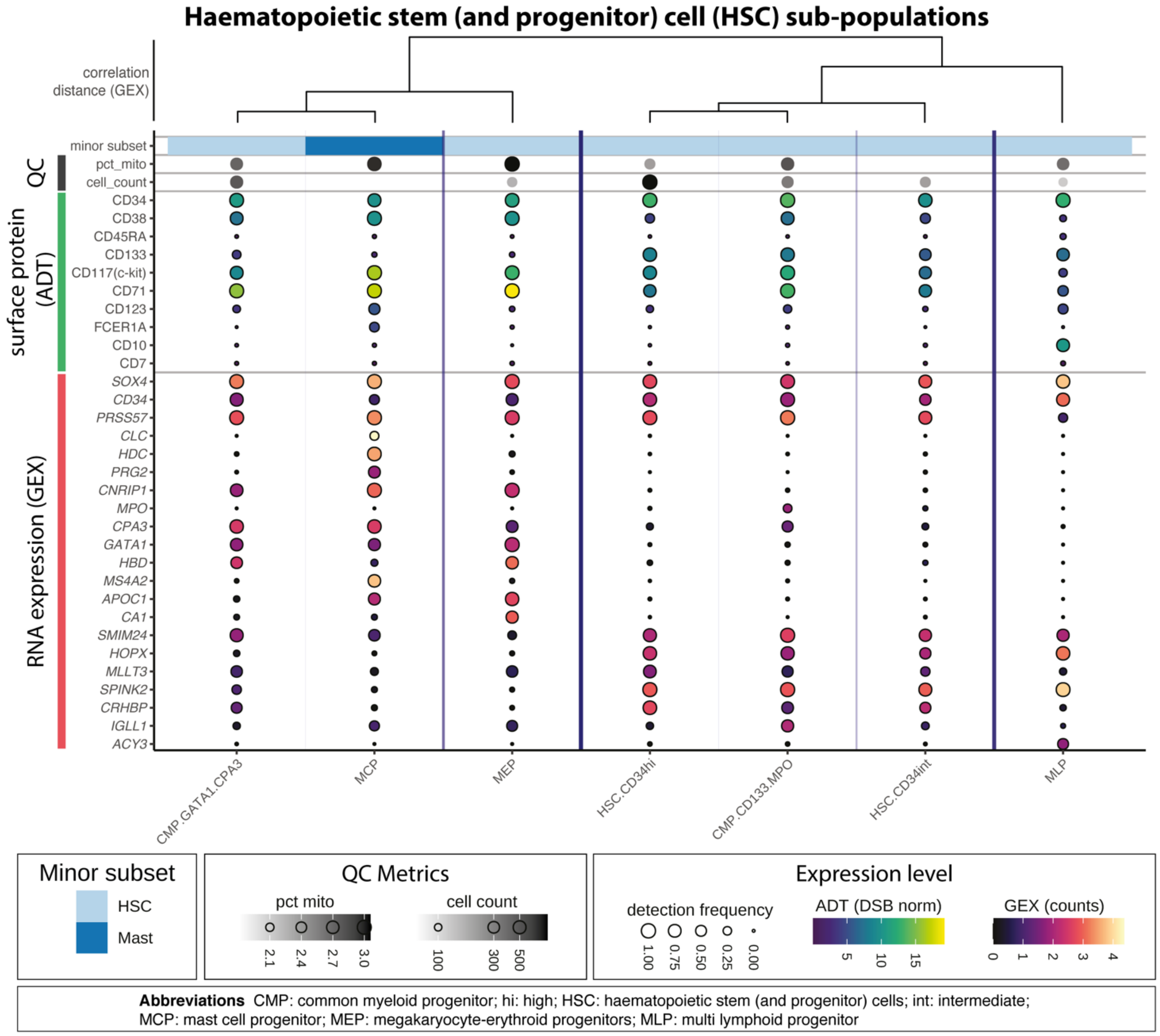

13: Dot plot of hematopoietic stem (and progenitor) cell sub-populations.

Platelet and erythrocyte sub-populations

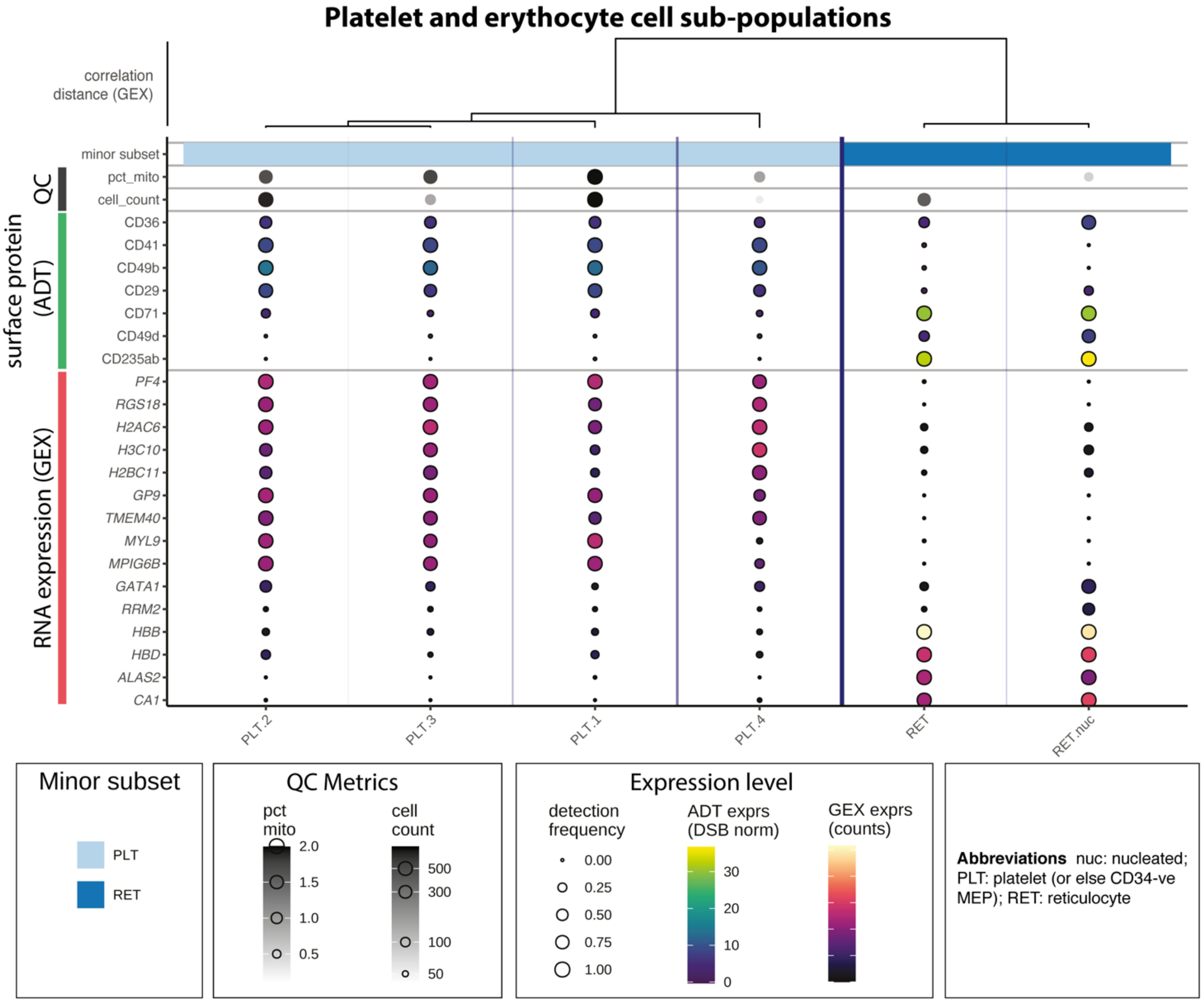

14: Dot plot of platelet and erythrocyte cell sub-populations.
