# Supplementary material for: A blood atlas of COVID-19 defines hallmarks of disease severity and specificity
Source: Cell. 2022 Mar 3;185(5):916–938.e58. doi: 10.1016/j.cell.2022.01.012 (PMC8776501; doi:10.1016/j.cell.2022.01.012)
Supplement: Data S5: Compositional analysis of CITE-seq data, related to STAR Methods [file mmc9.pdf]

Data S5: Compositional Analysis of CITE-seq Data, Related to STAR Methods

Related to **STAR Methods**: CITE-seq: composition analysis.

Table of Contents

Composition analysis **2**
Composition analysis of cell types 2
1: Cell type frequency by clinical category 2
2: Cell type PCA and association tests 3
3: Cell type differential abundance analysis 4
Composition analysis of major cell subsets 5
4: Major cell subset frequency by clinical category 5
5: Major cell subset PCA and association tests 6
6: Major cell subset differential abundance analysis 7
Composition analysis of minor cell subsets 8
7: Minor cell subset frequency by clinical category 8
8: Minor cell subset PCA and association tests 9
9: Minor cell subset differential abundance analysis 10
Composition analysis of T and natural killer cell clusters 11
10: T and NK cluster frequency by clinical category 16
11: T and NK cluster PCA and association tests 17
12: T and NK cluster differential abundance analysis 18
Composition analysis of B and plasmablast cell clusters 19
13: B and PB cluster frequency by clinical category 19
14: B and PB PCA and association tests 20
15: B and PB cluster differential abundance analysis 21
Composition analysis of mononuclear phagocyte clusters 22
16: MNP cluster frequency by clinical category 22
17: MNP cluster PCA and association tests 23
18: MNP cluster differential abundance analysis 24

Composition analysis

Composition analysis of cell types

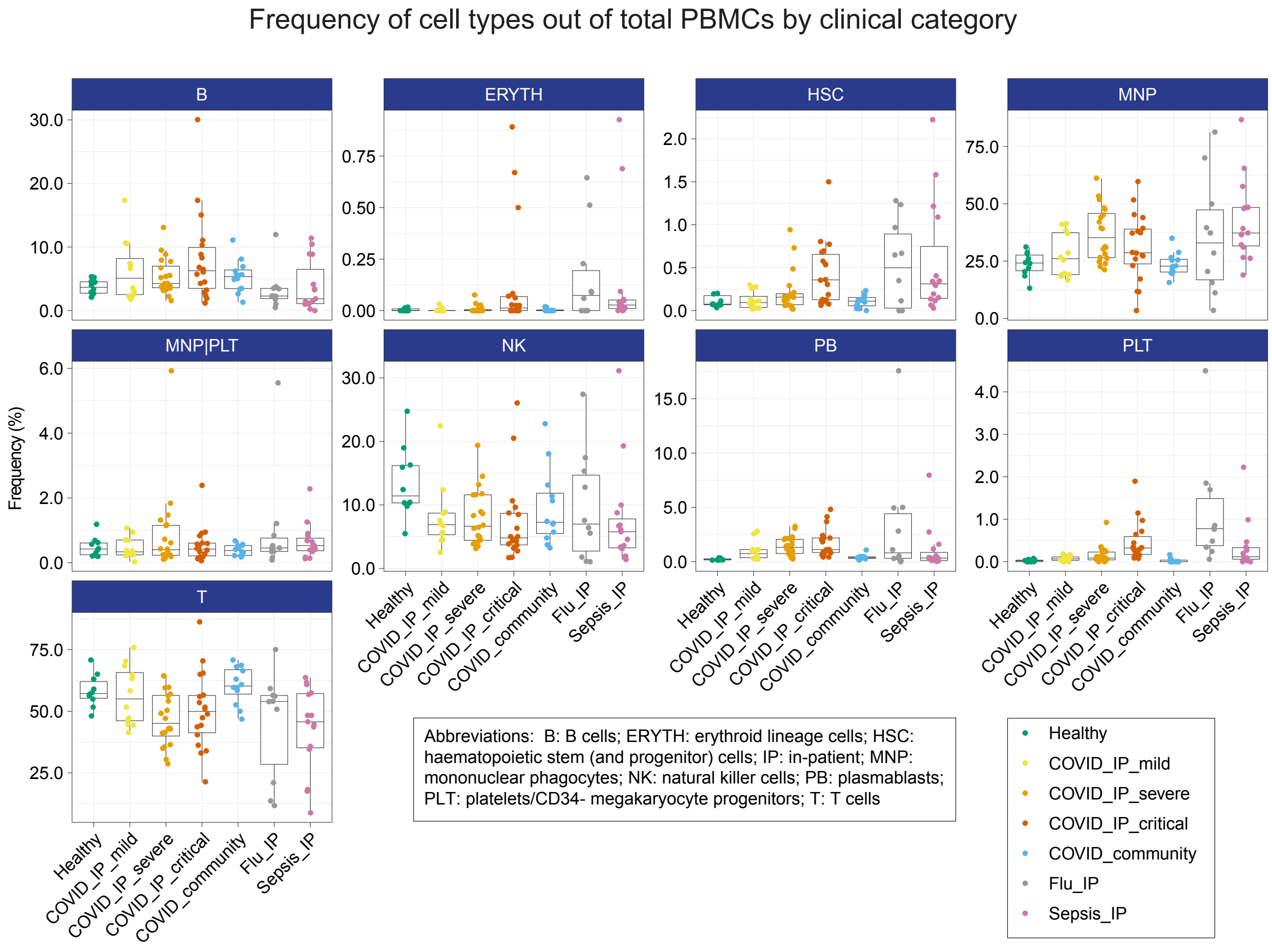

1: Cell type frequency by clinical category. The box plots show the median and the first and third quartiles; whiskers show 1.5 × the interquartile range above and below the box. Cell types are ordered alphabetically. Related to STAR Methods: CITE-seq: composition analysis.

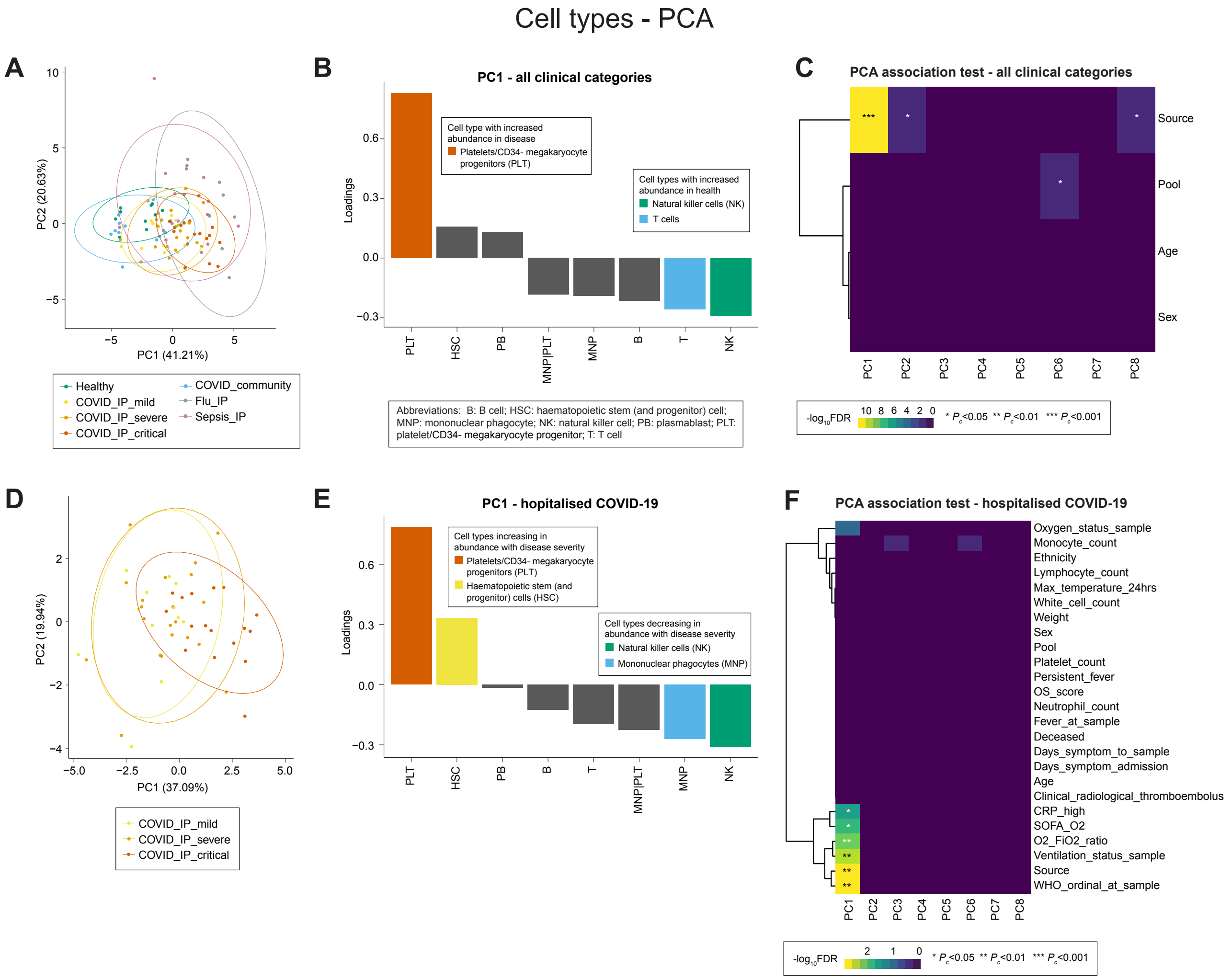

2: Cell type PCA and association tests. (A) PCA plot for all clinical categories. Percentage of variance explained by each PC is shown in the brackets. (B) Loadings of cell types on PC1 from analysis including all clinical categories. (C) Association test between top PCs and source (clinical category), age, sex, and sample pool for all clinical categories. (D) PCA plot for hospitalized COVID-19. Percentage of variance explained by each PC is shown in the brackets. (E) Loadings of cell types on PC1 from analysis of hospitalized COVID-19 cases. (F) Association test between top PCs and clinical, demographic and experimental variables for hospitalized COVID-19 cases. Significance tested using Benjamini-Hochberg-corrected ANOVA. Related to STAR Methods: CITE-seq: composition analysis.

Cell types - edgeR differential abundance analysis

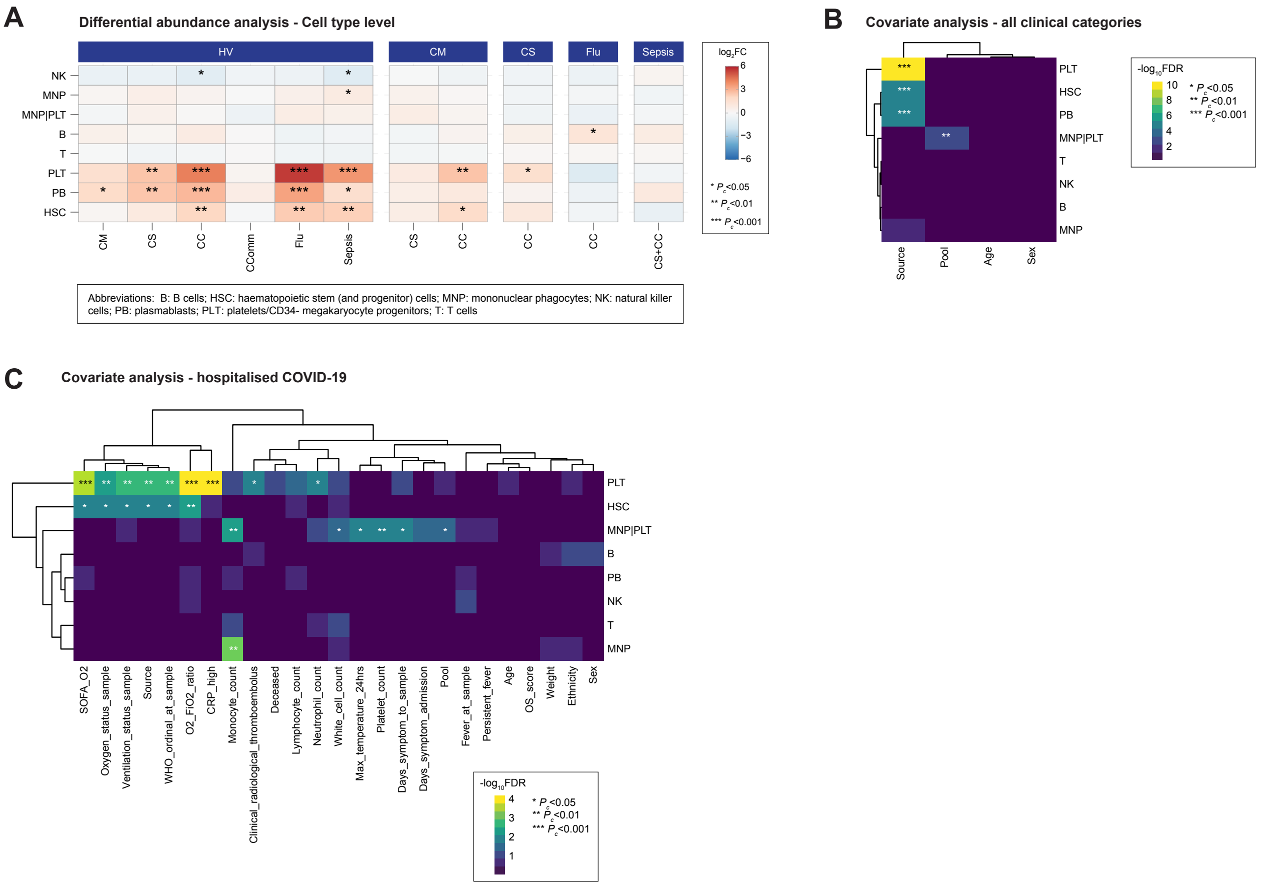

3: Cell type differential abundance analysis. (A) Cell type differential abundance analysis between clinical categories, controlling for age, sex and sample pool effects. (B) Cell type covariate analysis for source (clinical category), age, sex and sample pool for all clinical categories. (C) Cell type covariate analysis for clinical, demographic and experimental variables for hospitalized COVID-19 cases. Significance tested using Benjamini-Hochberg-corrected ANOVA. Related to STAR Methods: CITE-seq: composition analysis.

Composition analysis of major cell subsets

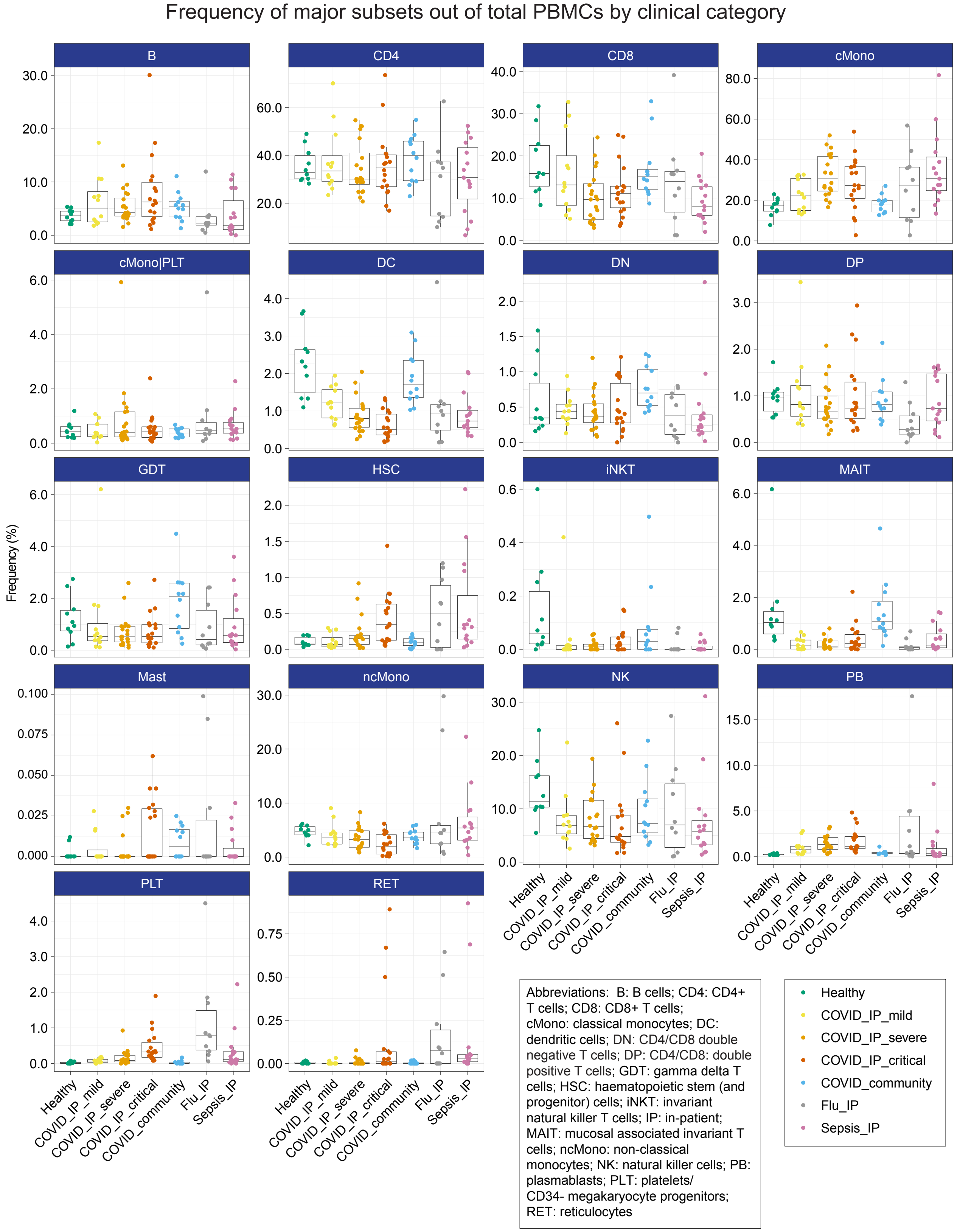

4: Major cell subset frequency by clinical category. Box plots show the median and the first and third quartiles; whiskers show 1.5 × the interquartile range above and below the box. Major subsets are ordered alphabetically. Related to STAR Methods: CITE-seq: composition analysis.

Major subsets - PCA

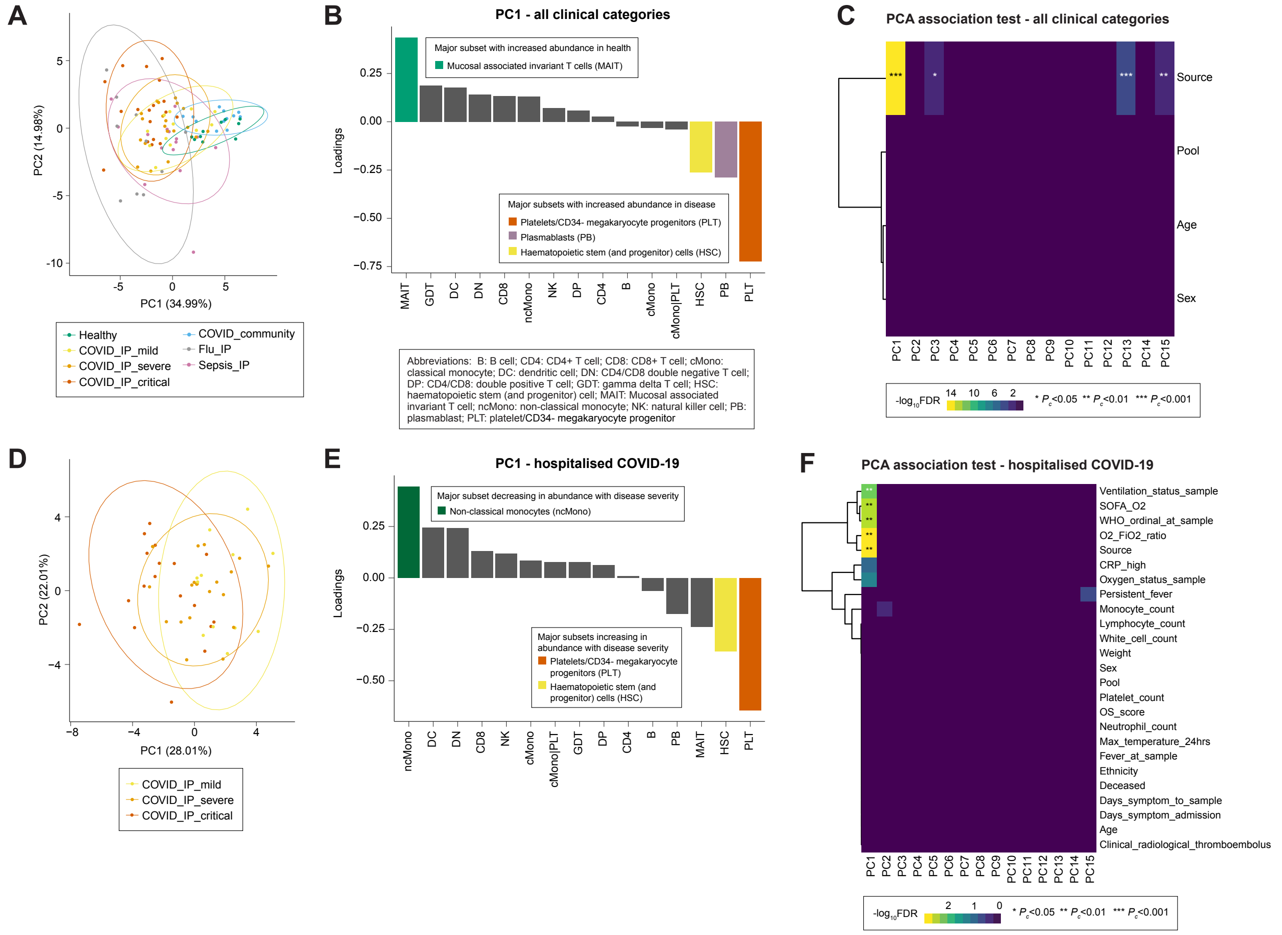

5: Major cell subset PCA and association tests. (A) PCA plot for all clinical categories. Percentage of variance explained by each PC is shown in the brackets. (B) Loadings of major subsets on PC1 from analysis including all clinical categories. (C) Association test between top PCs and source (clinical category), age, sex, and sample pool for all clinical categories. (D) PCA plot for hospitalized COVID-19. Percentage of variance explained by each PC is shown in the brackets. (E) Loadings of major subsets on PC1 from analysis of hospitalized COVID-19 cases. (F) Association test between top PCs and clinical, demographic and experimental variables for hospitalized COVID-19 cases. Significance tested using Benjamini-Hochberg-corrected ANOVA. Related to STAR Methods: CITE-seq: composition analysis.

Major subsets - edgeR differential abundance analysis

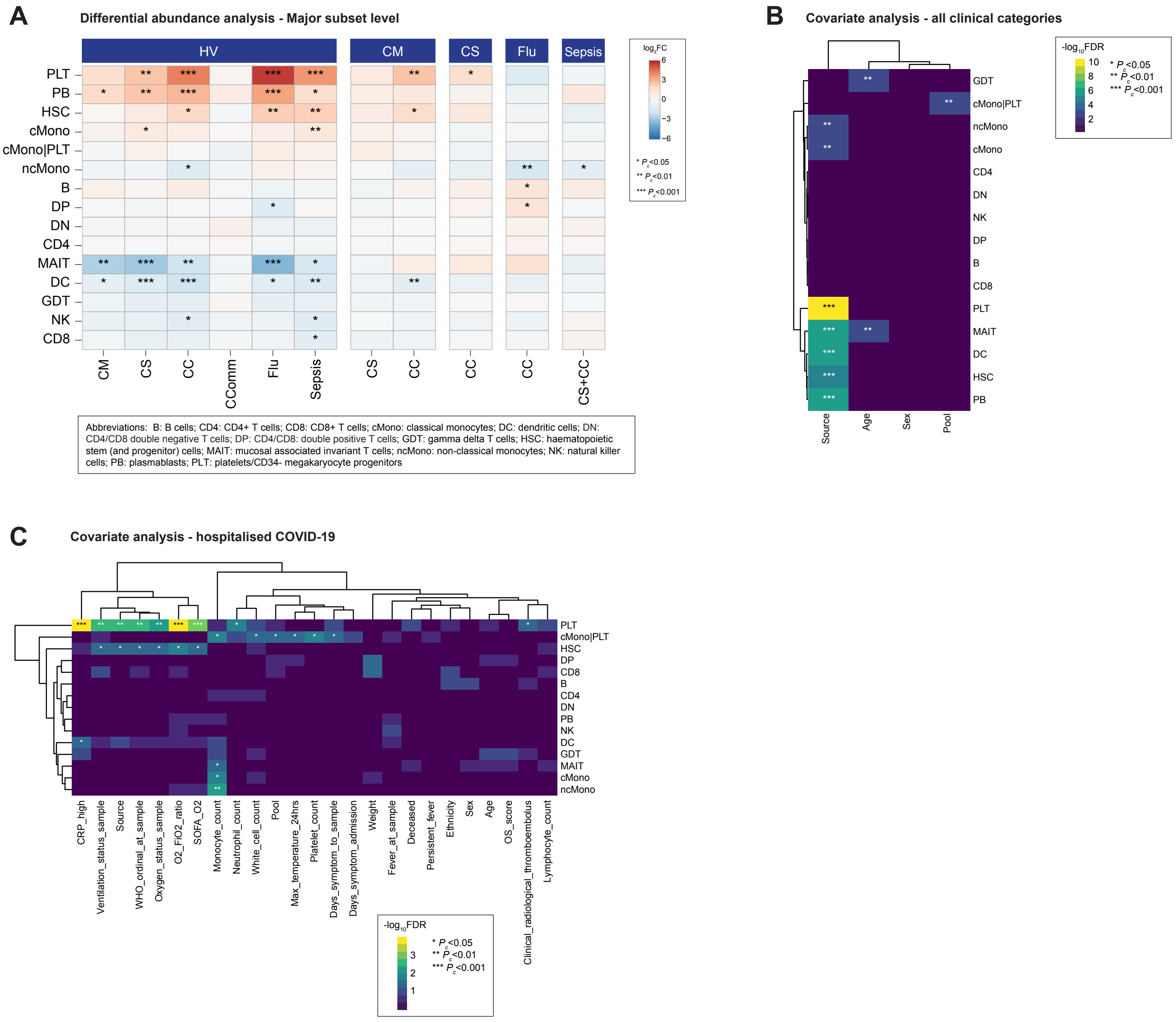

6: Major cell subset differential abundance analysis. (A) Major subset differential abundance analysis between clinical categories, controlling for age, sex and sample pool. (B) Major subset covariate analysis for source (clinical category), age, sex and pool for all clinical categories. (C) Major subset covariate analysis for clinical, demographic and experimental variables for hospitalized COVID-19 cases. Significance tested using Benjamini-Hochberg-corrected ANOVA. Related to STAR Methods: CITE-seq: composition analysis.

Composition analysis of minor cell subsets

Frequency of minor subsets out of total PBMCs by clinical category

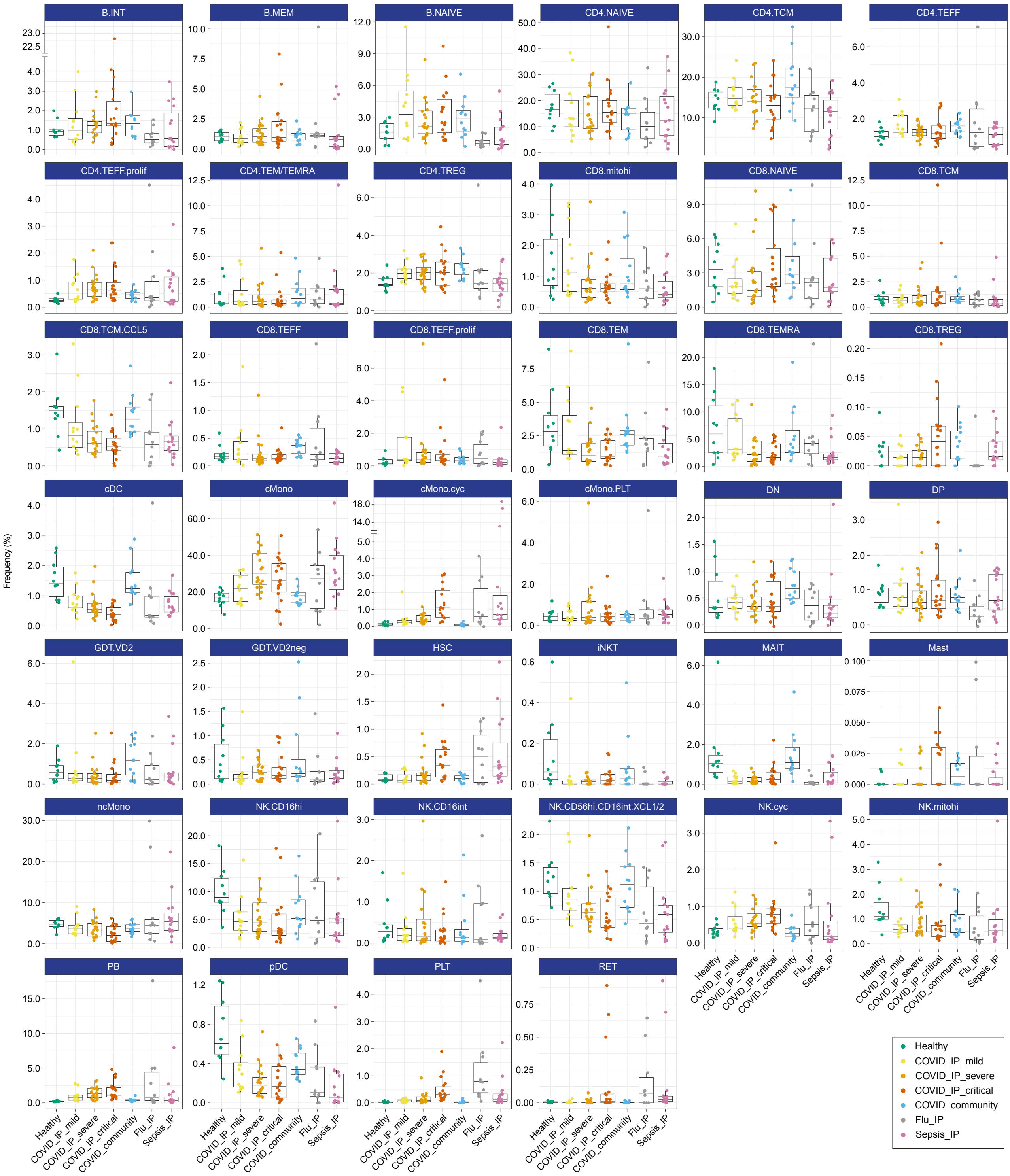

Abbreviations: B: B cell; cDC: classical dendritic cell; cMono: classical monocyte; cyc: cycling; DN: CD4/CD8 double negative T cell; DP: CD4/CD8: double positive T cell; GDT: gamma delta T cell; hi: high; HSC: haematopoietic stem (and progenitor) cell; iNKT: invariant NKT cell; INT/int: intermediate; IP: in-patient; MAIT: mucosal associated invariant T cell; MEM: memory; mito: mitochondrial; ncMono: non-classical monocyte; neg: negative; NK: natural killer cell; PB: plasmablast; pDC: plasmacytoid dendritic cell; PLT: platelet/CD34- megakaryocyte progenitor; prolif: proliferating; TCM: T central memory; TEFF: T effector; TEM(RA): T effector memory (CD45RA re-expressing); TREG: T regulatory cell; RET: reticulocytes

7: Minor cell subset frequency by clinical category. Box plots show the median and the first and third quartiles; whiskers show 1.5 × the interquartile range above and below the box. Minor subsets are ordered alphabetically. Related to STAR Methods: CITE-seq: composition analysis.

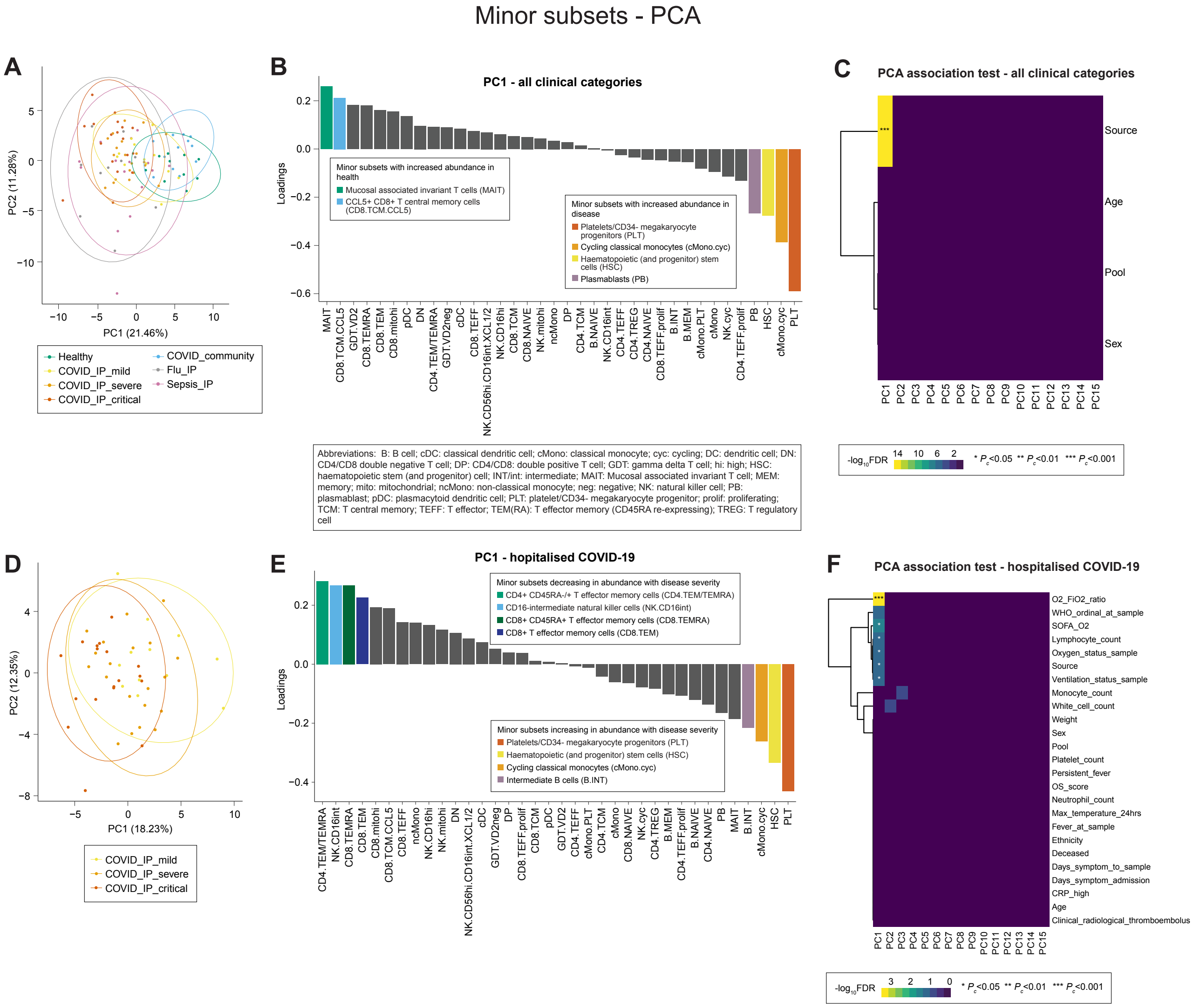

8: Minor cell subset PCA and association tests. (A) PCA plot for all clinical categories. Percentage of variance explained by each PC is shown in the brackets. (B) Loadings of minor subsets on PC1 from analysis including all clinical categories. (C) Association test between top PCs and source (clinical category), age, sex, and sample pool for all clinical categories. (D) PCA plot for hospitalized COVID-19. Percentage of variance explained by each PC is shown in the brackets. (E) Loadings of minor subsets on PC1 from analysis of hospitalized COVID-19 cases. (F) Association test between top PCs and clinical, demographic and experimental variables for hospitalized COVID-19 cases. Significance tested using Benjamini-Hochberg-corrected ANOVA. Related to STAR Methods: CITE-seq: composition analysis.

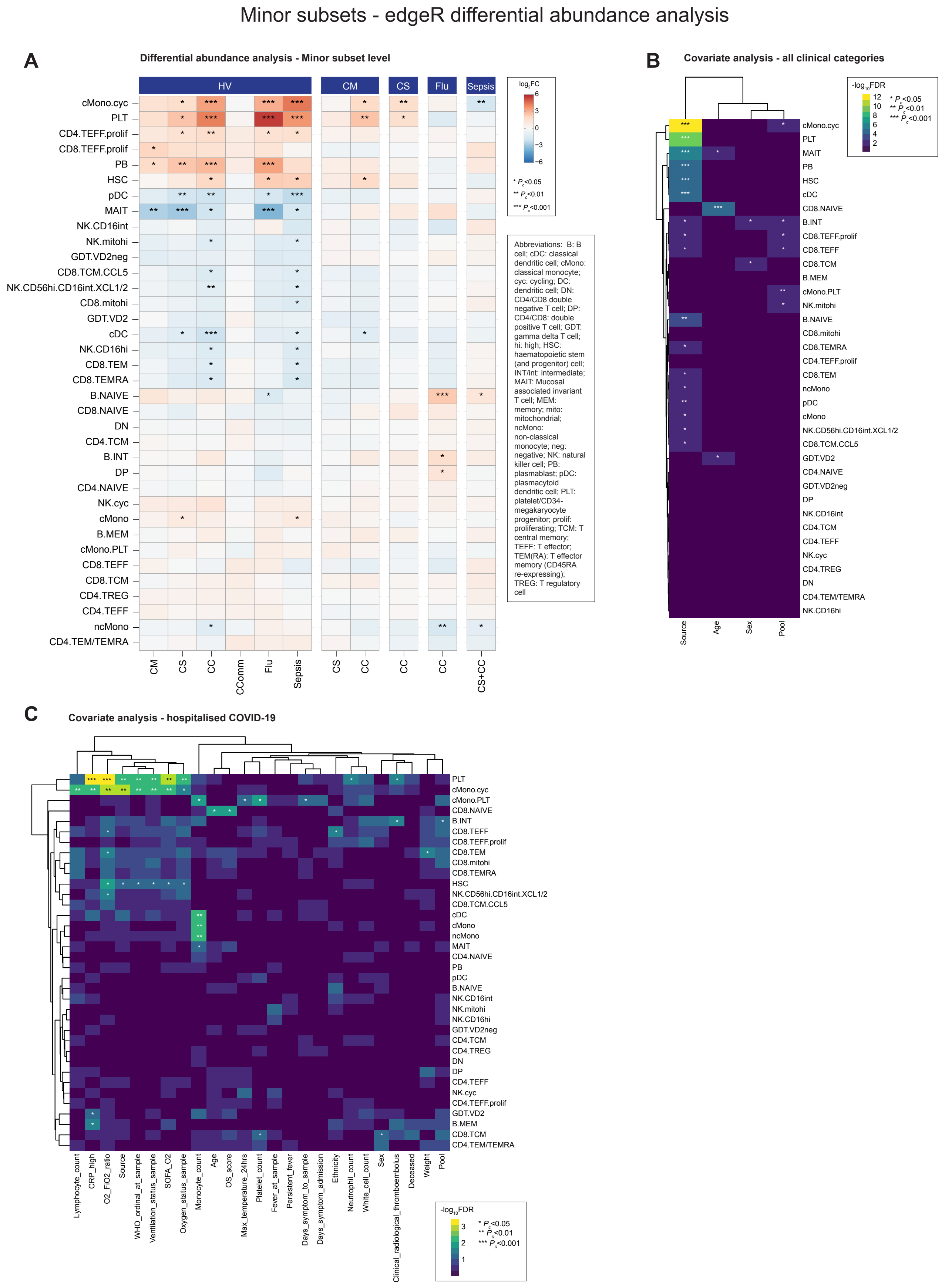

9: Minor cell subset differential abundance analysis. (A) Minor subset differential abundance analysis between clinical categories, controlling for age, sex and sample pool. (B) Minor subset covariate analysis for source (clinical category), age, sex and pool for all clinical categories. (C) Minor subset covariate analysis for clinical, demographic and experimental variables for hospitalized COVID-19 cases. Significance tested using Benjamini-Hochberg-corrected ANOVA. Related to STAR Methods: CITE-seq: composition analysis.

Composition analysis of T and natural killer cell clusters

Frequency of clusters out of total T and natural killer cells by clinical category (1)

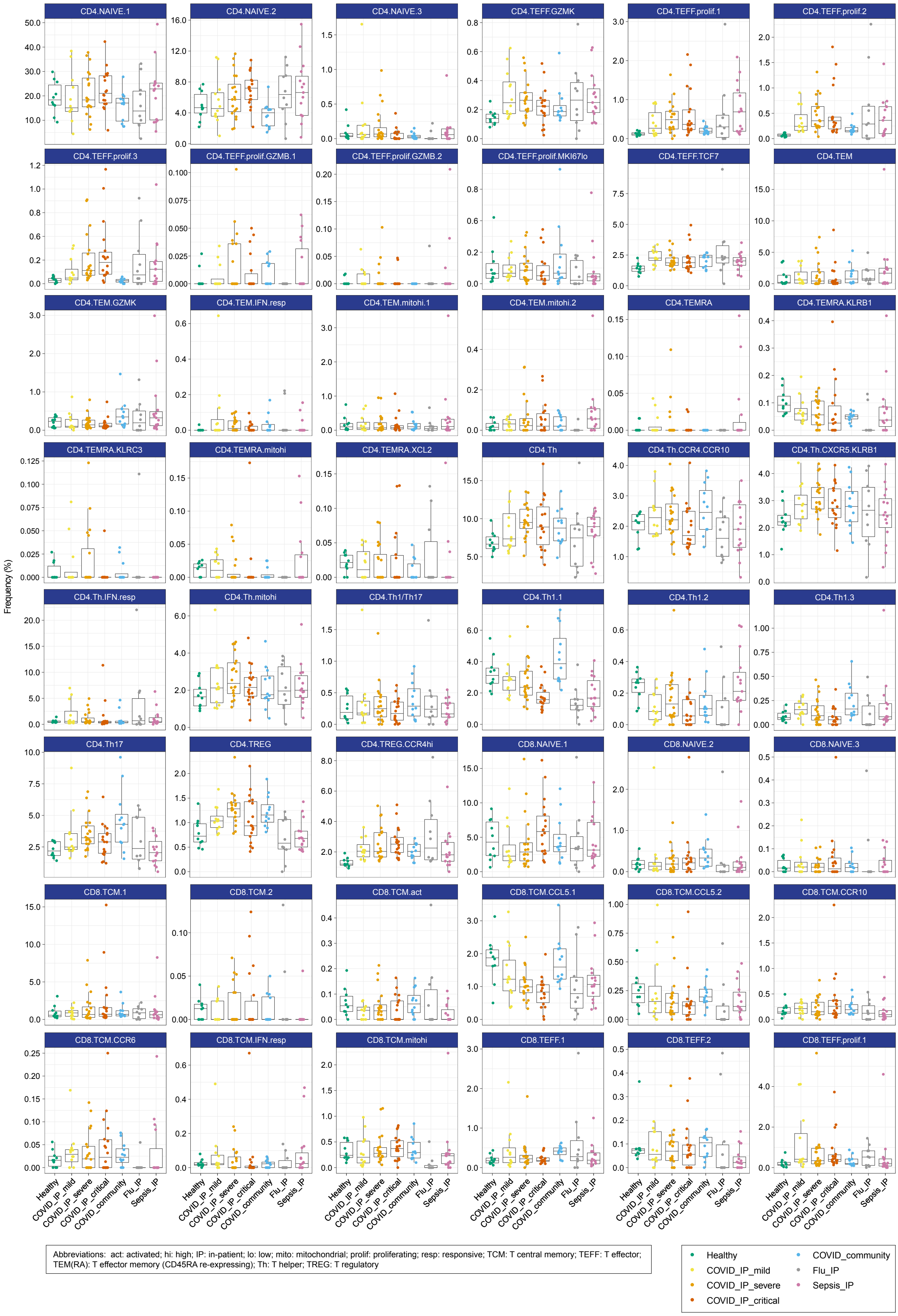

Abbreviations: act: activated; hi: high; IP: in-patient; lo: low; mito: mitochondrial; prolif: proliferating; resp: responsive; TCM: T central memory; TEFF: T effector; TEM(RA): T effector memory (CD45RA re-expressing); Th: T helper; TREG: T regulatory

Frequency of clusters out of total T and natural killer cells by clinical category (2)

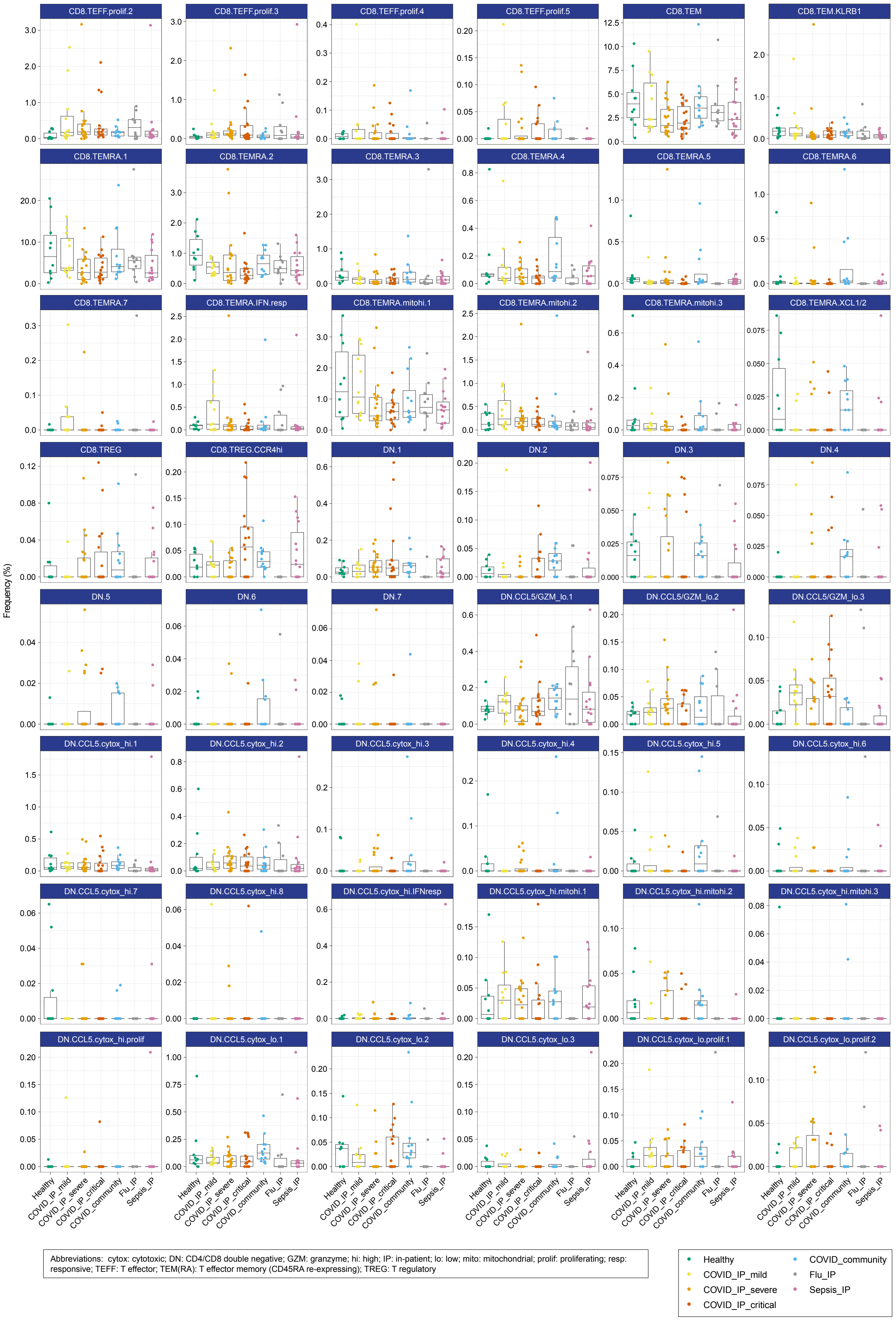

Abbreviations: cytox: cytotoxic; DN: CD4/CD8 double negative; GZM: granzyme; hi: high; IP: in-patient; lo: low; mito: mitochondrial; prolif: proliferating; resp: responsive; TEFF: T effector; TEM(RA): T effector memory (CD45RA re-expressing); TREG: T regulatory

Frequency of clusters out of total T and natural killer cells by clinical category (3)

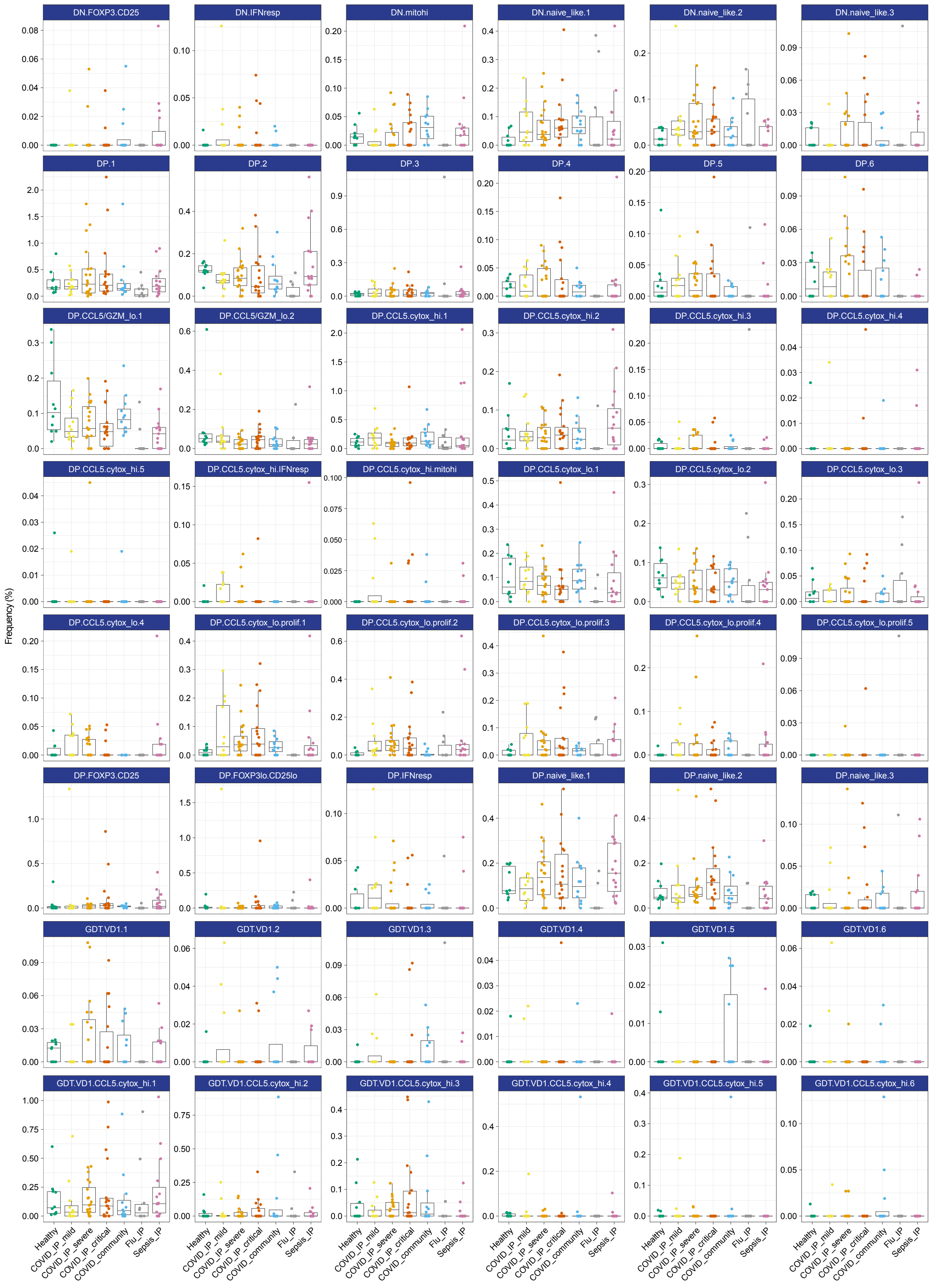

Abbreviations: cytox: cytotoxic; DN: CD4/CD8 double negative; DP: CD4/CD8: double positive; GDT: gamma delta T; GZM: granzyme; hi: high; IP: in-patient; lo: low; mito: mitochondrial; prolif: proliferating; resp: responsive

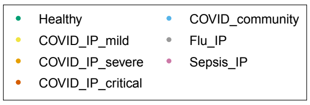

Frequency of clusters out of total T and natural killer cells by clinical category (4)

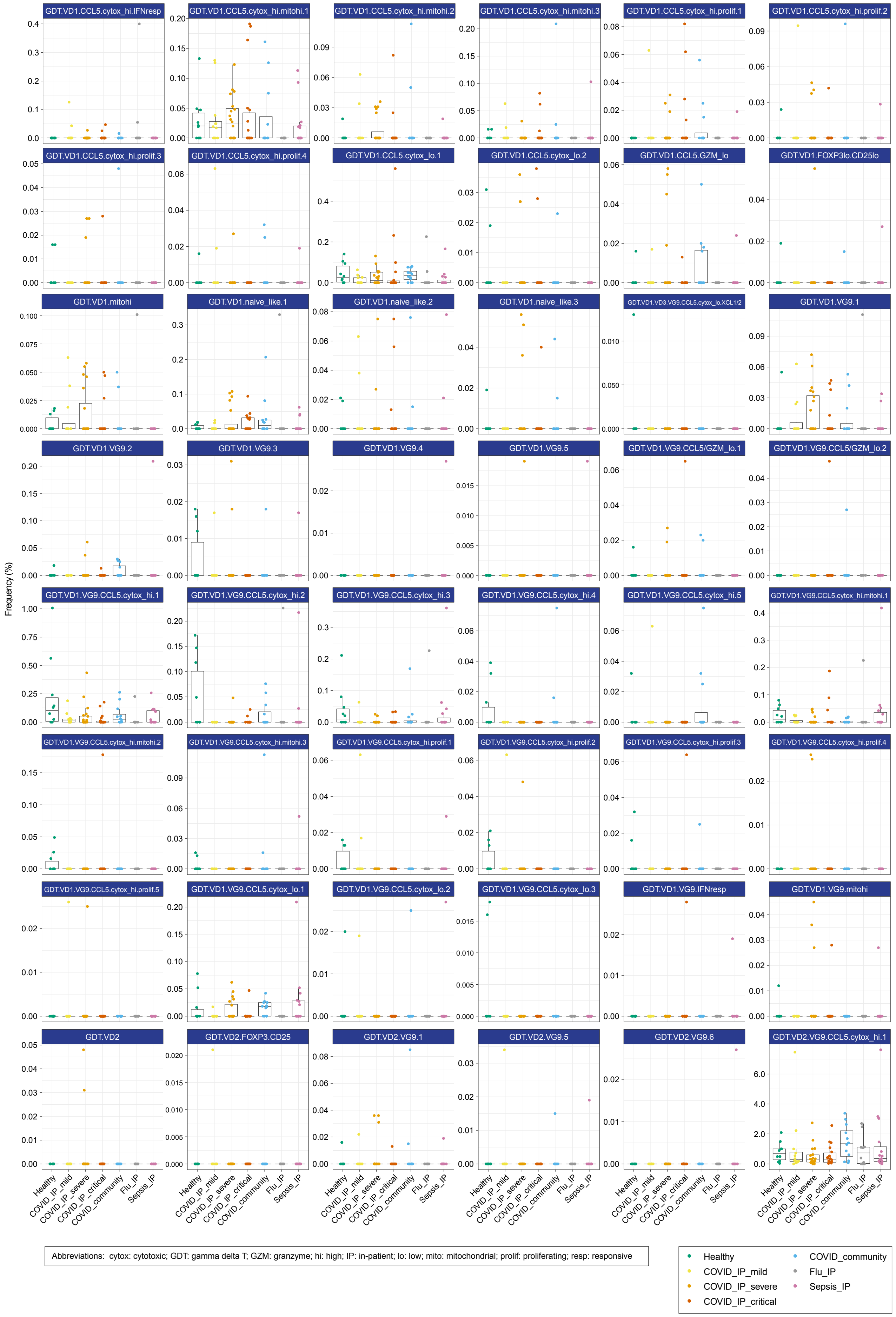

Abbreviations: cytox: cytotoxic; GDT: gamma delta T; GZM: granzyme; hi: high; IP: in-patient; lo: low; mito: mitochondrial; prolif: proliferating; resp: responsive

Frequency of clusters out of total T and natural killer cells by clinical category (5)

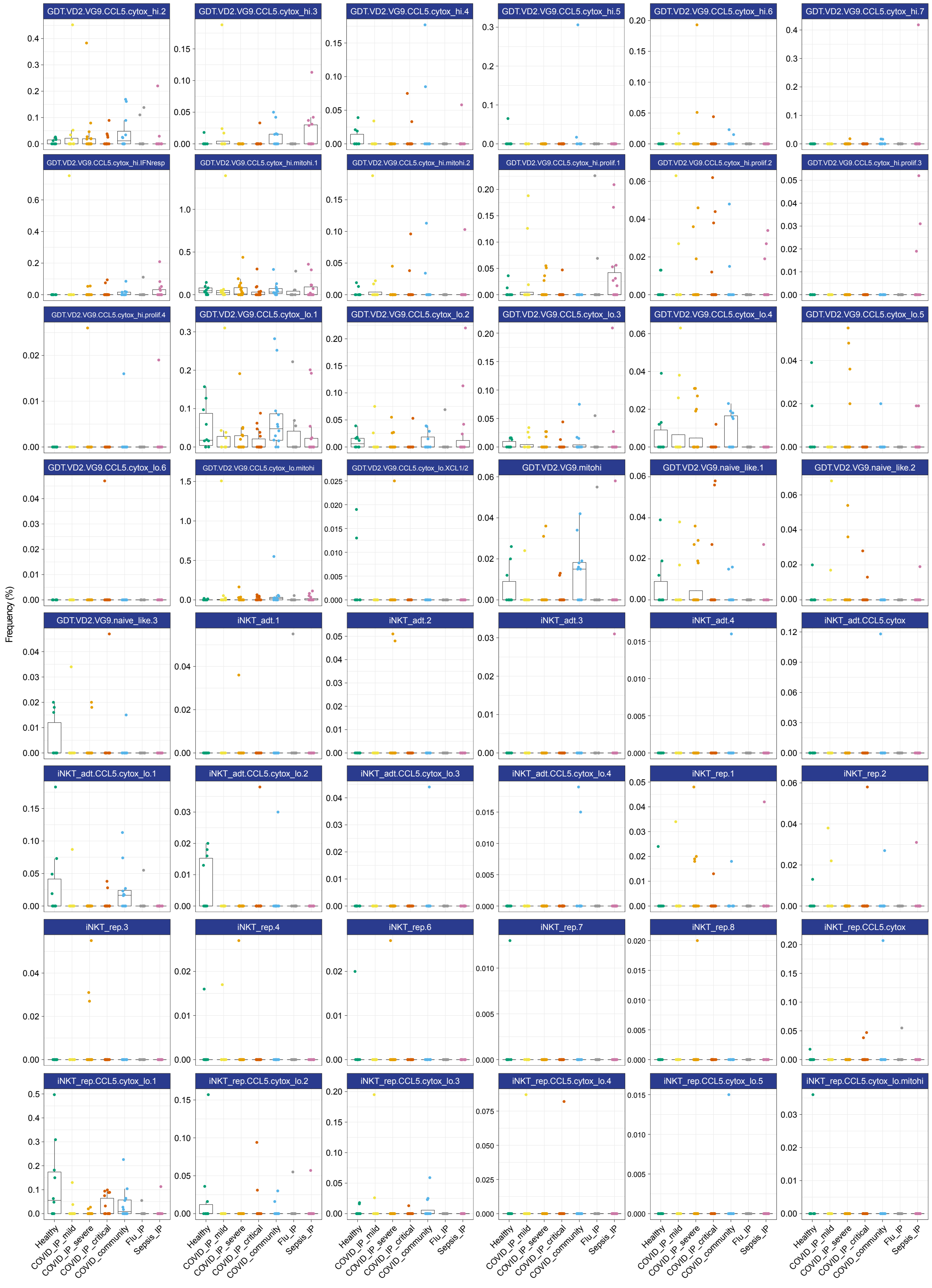

Abbreviations: adt: antibody-dependent tag-based annotation; cytox: cytotoxic; GDT: gamma delta T; hi: high; iNKT: invariant natural killer T cell; IP: in-patient; lo: low; mito: mitochondrial; prolif: proliferating; rep: repertoire-based annotation; resp: responsive

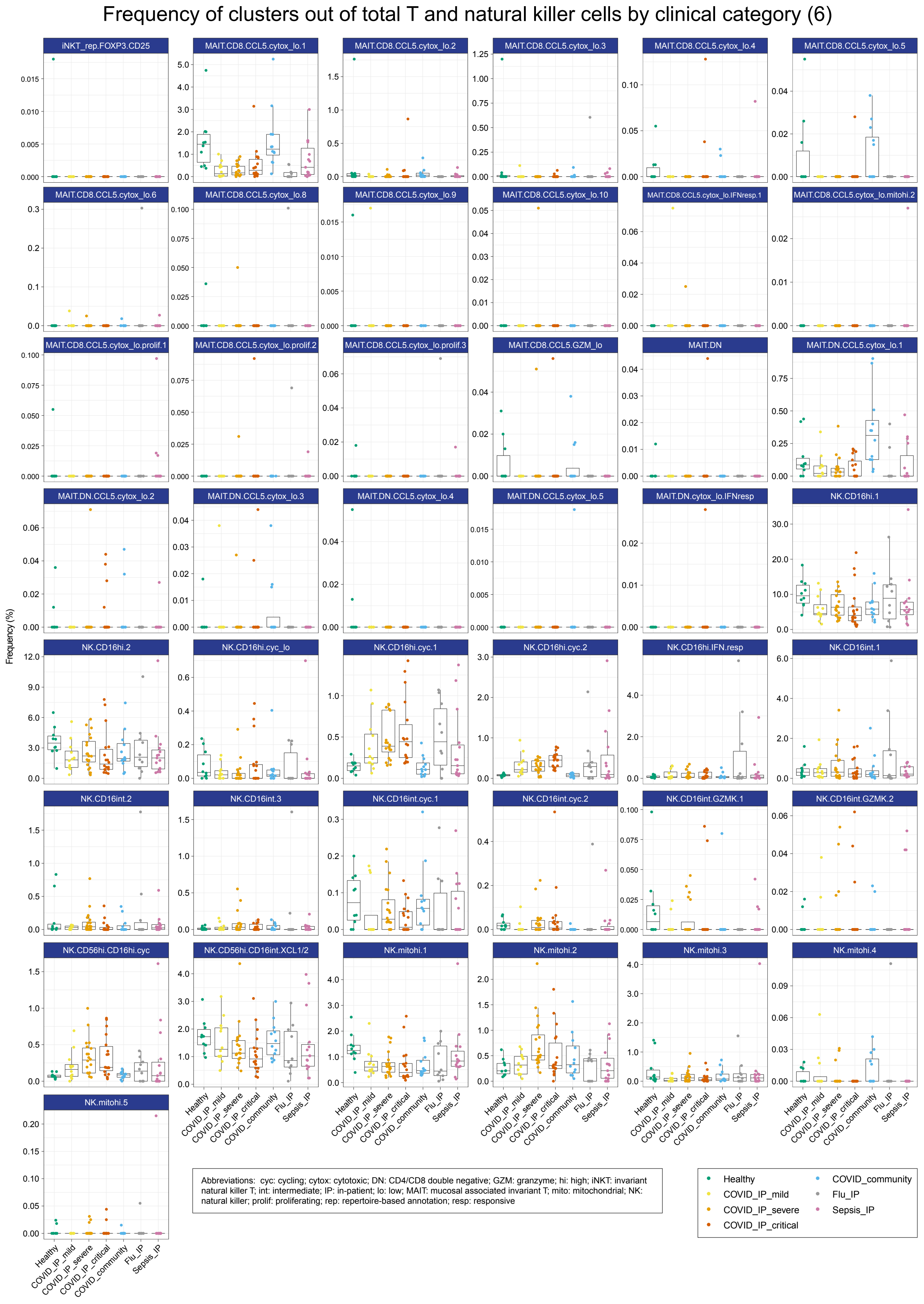

10: T and NK cluster frequency by clinical category. Box plots show the median and the first and third quartiles; whiskers show 1.5 × the interquartile range above and below the box. Clusters are ordered alphabetically. Related to STAR Methods: CITE-seq: composition analysis.

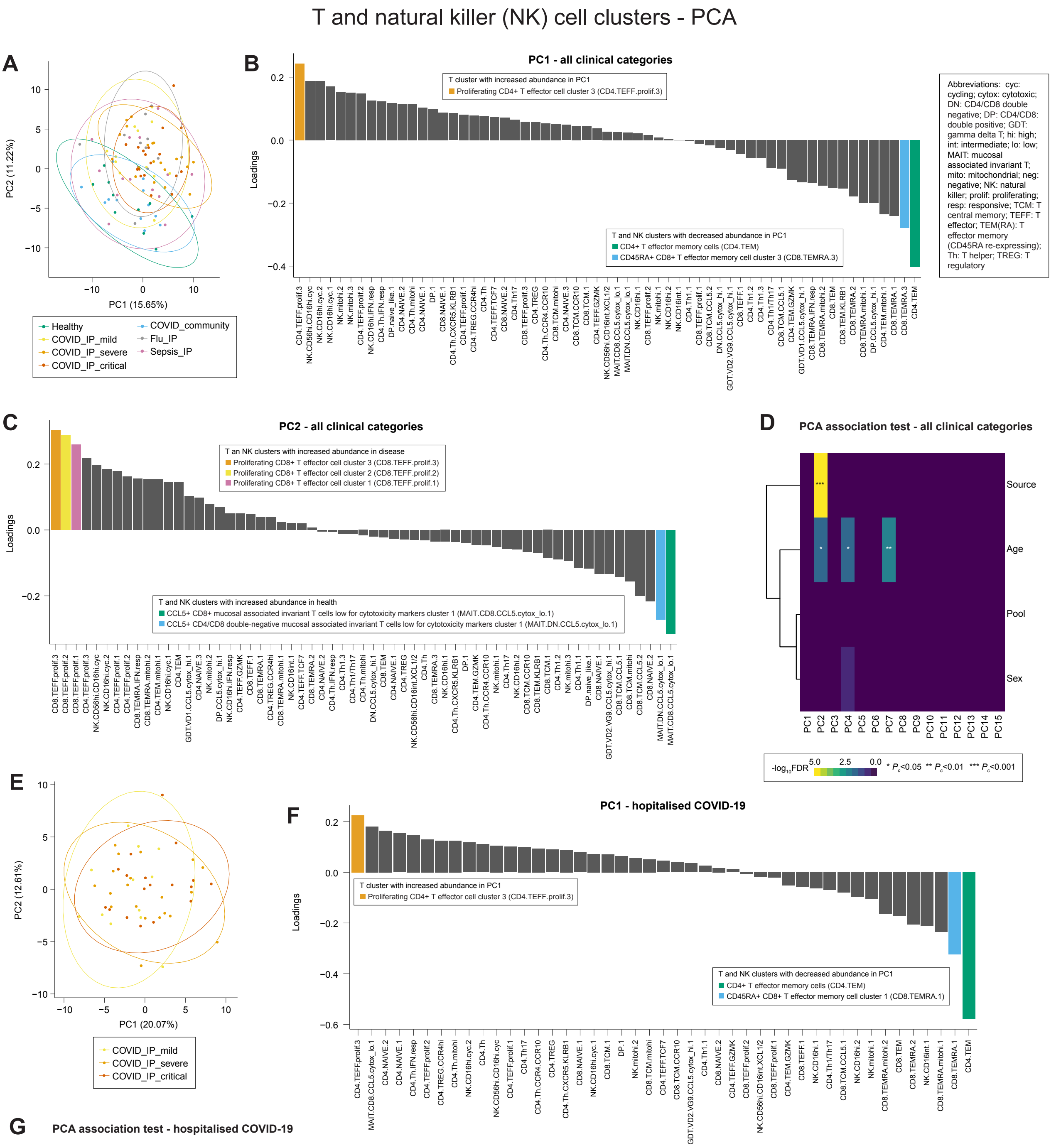

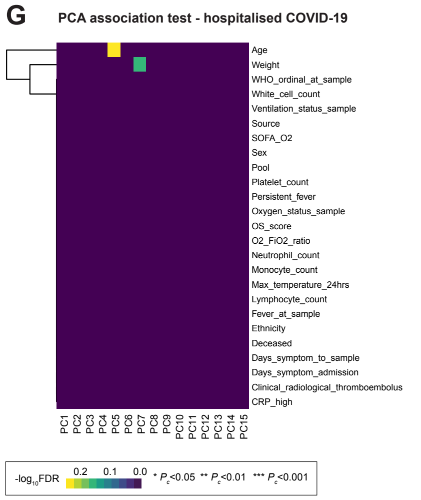

11: T and NK cluster PCA and association tests.

(A) PCA plot for all clinical categories. Percentage of variance explained by each PC is shown in the brackets. (B) Loadings of T and NK clusters on PC1 from analysis including all clinical categories. (C) Loadings of T and NK clusters on PC2 (associated with source as shown in (D)), from analysis including all clinical categories. (D) Association test between top PCs and source (clinical category), age, sex, and sample pool for all clinical categories. (E) PCA plot for hospitalised COVID-19. Percentage of variance explained by each PC is shown in the brackets. (F) Loadings of T and NK clusters on PC1 from analysis of hospitalised COVID-19 cases. (G) Association test between top PCs and clinical, demographic and experimental variables for hospitalised COVID-19 cases. Significance tested using Benjamini-Hochberg-corrected ANOVA. Related to STAR Methods: CITE-seq: composition analysis.

T and natural killer (NK) cell clusters - edgeR differential abundance analysis

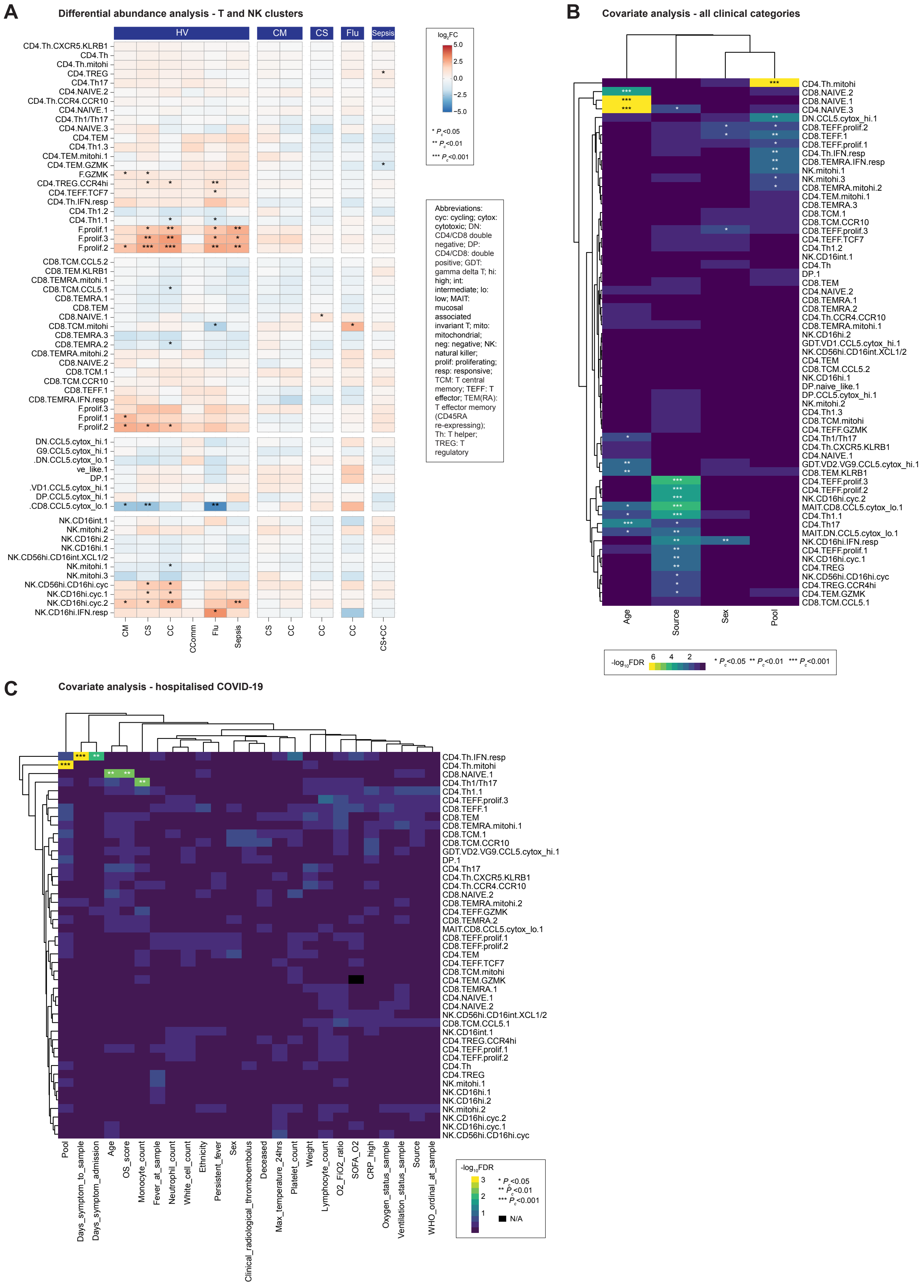

12: T and NK cluster differential abundance analysis. (A) T and NK cluster differential abundance analysis between clinical categories, controlling for age, sex, and sample pool. (B) T and NK cluster covariate analysis for source (clinical category), age, sex and pool for all clinical categories. (C) T and NK cluster covariate analysis for clinical, demographic and experimental variables for hospitalized COVID-19 cases. Significance tested using Benjamini-Hochberg-corrected ANOVA. Related to STAR Methods: CITE-seq: composition analysis.

Composition analysis of B and plasmablast cell clusters

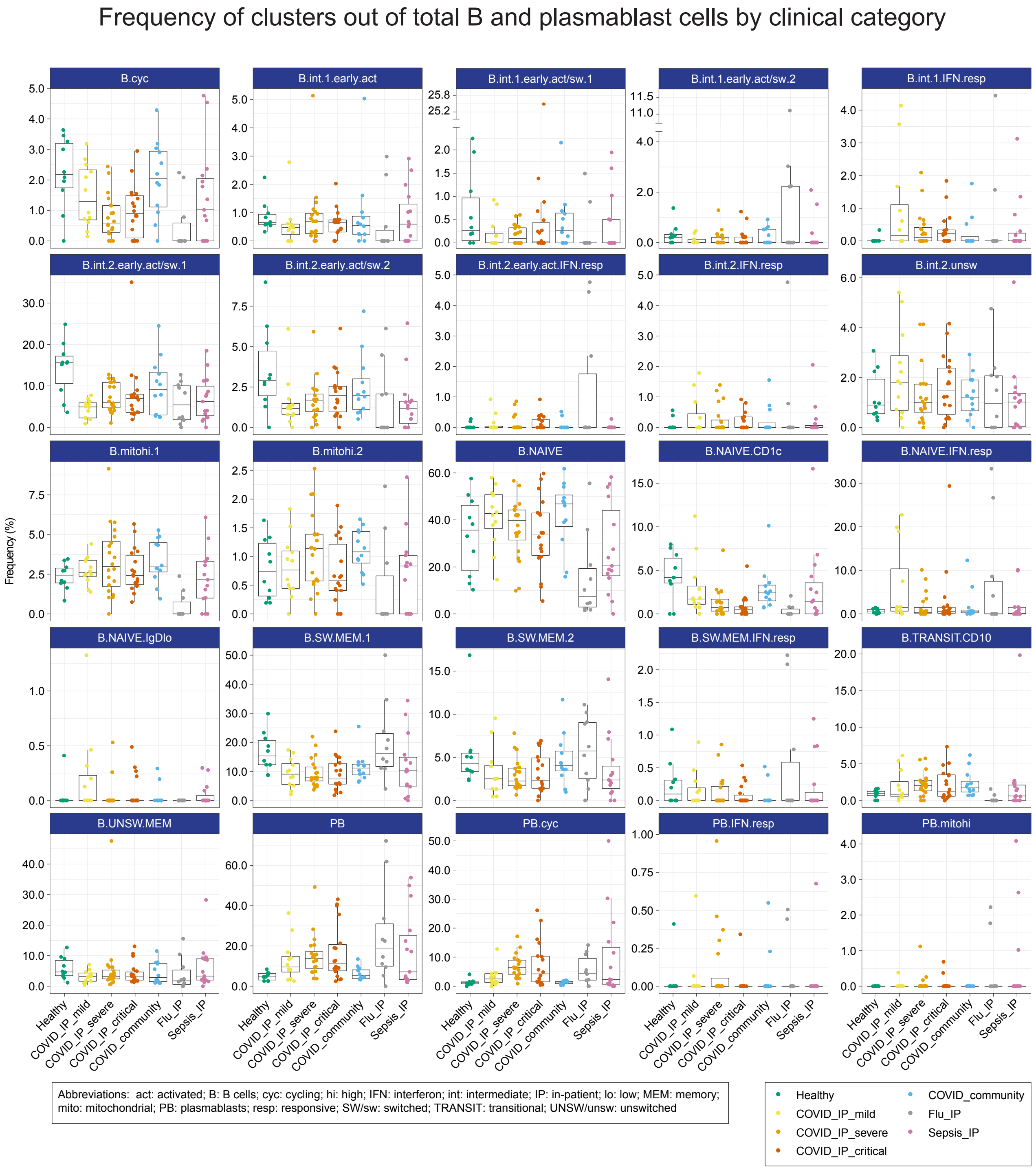

13: B and PB cluster frequency by clinical category. Box plots show the median and the first and third quartiles; whiskers show 1.5 × the interquartile range above and below the box. Clusters are ordered alphabetically. Related to STAR Methods: CITE-seq: composition analysis.

B and plasmablast (PB) cell clusters - PCA

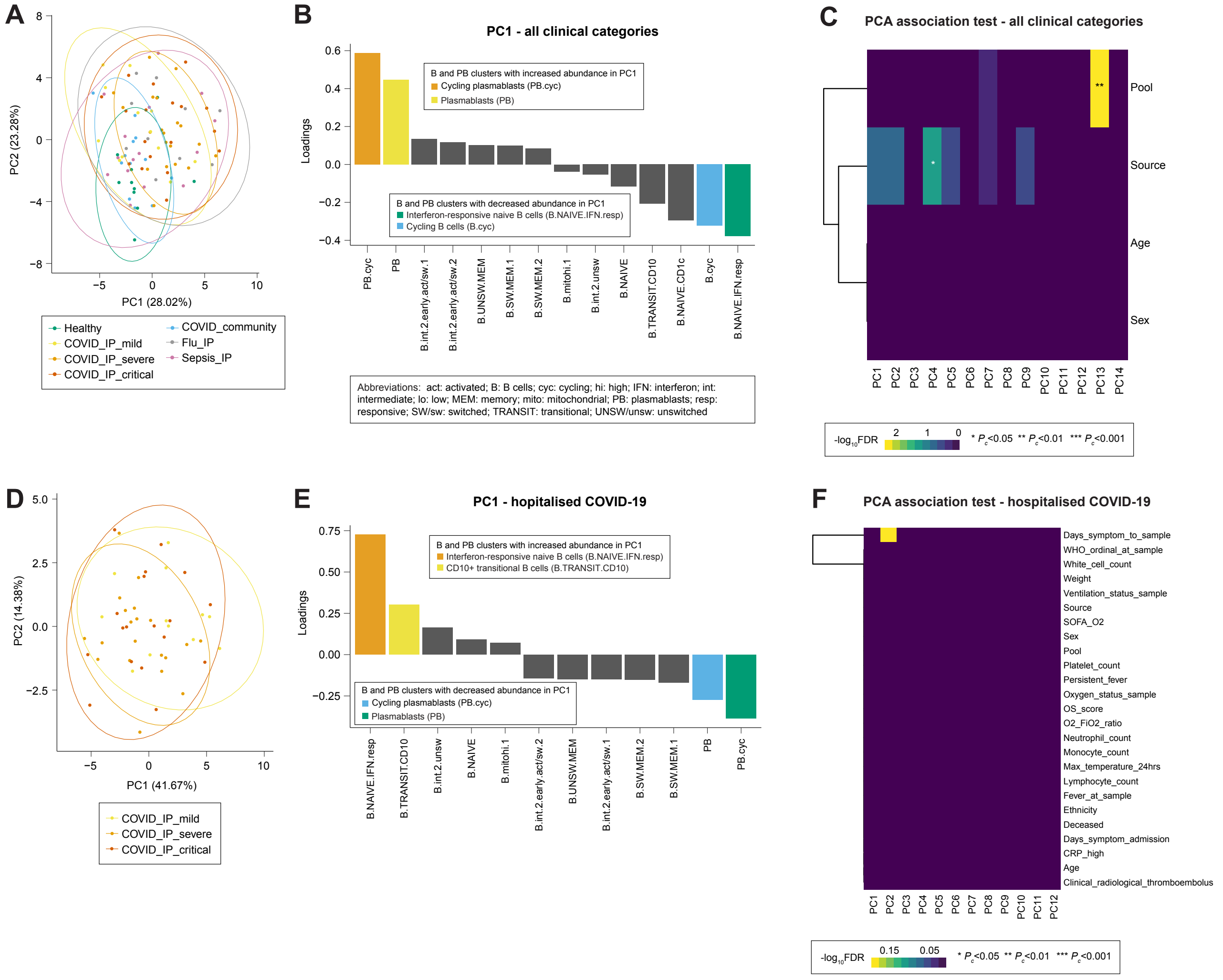

14: B and PB PCA and association tests. (A) PCA plot for all clinical categories. Percentage of variance explained by each PC is shown in the brackets. (B) Loadings of B and PB clusters on PC1 from analysis including all clinical categories. (C) Association test between top PCs and source (clinical category), age, sex, and sample pool for all clinical categories. (D) PCA plot for hospitalized COVID-19. Percentage of variance explained by each PC is shown in the brackets. (E) Loadings of B and PB clusters on PC1 from analysis of hospitalized COVID-19 cases. (F) Association test between top PCs and clinical, demographic and experimental variables for hospitalized COVID-19 cases. Significance tested using Benjamini-Hochberg-corrected ANOVA. Related to STAR Methods: CITE-seq: composition analysis.

B and plasmablast (PB) cell clusters - edgeR differential abundance analysis

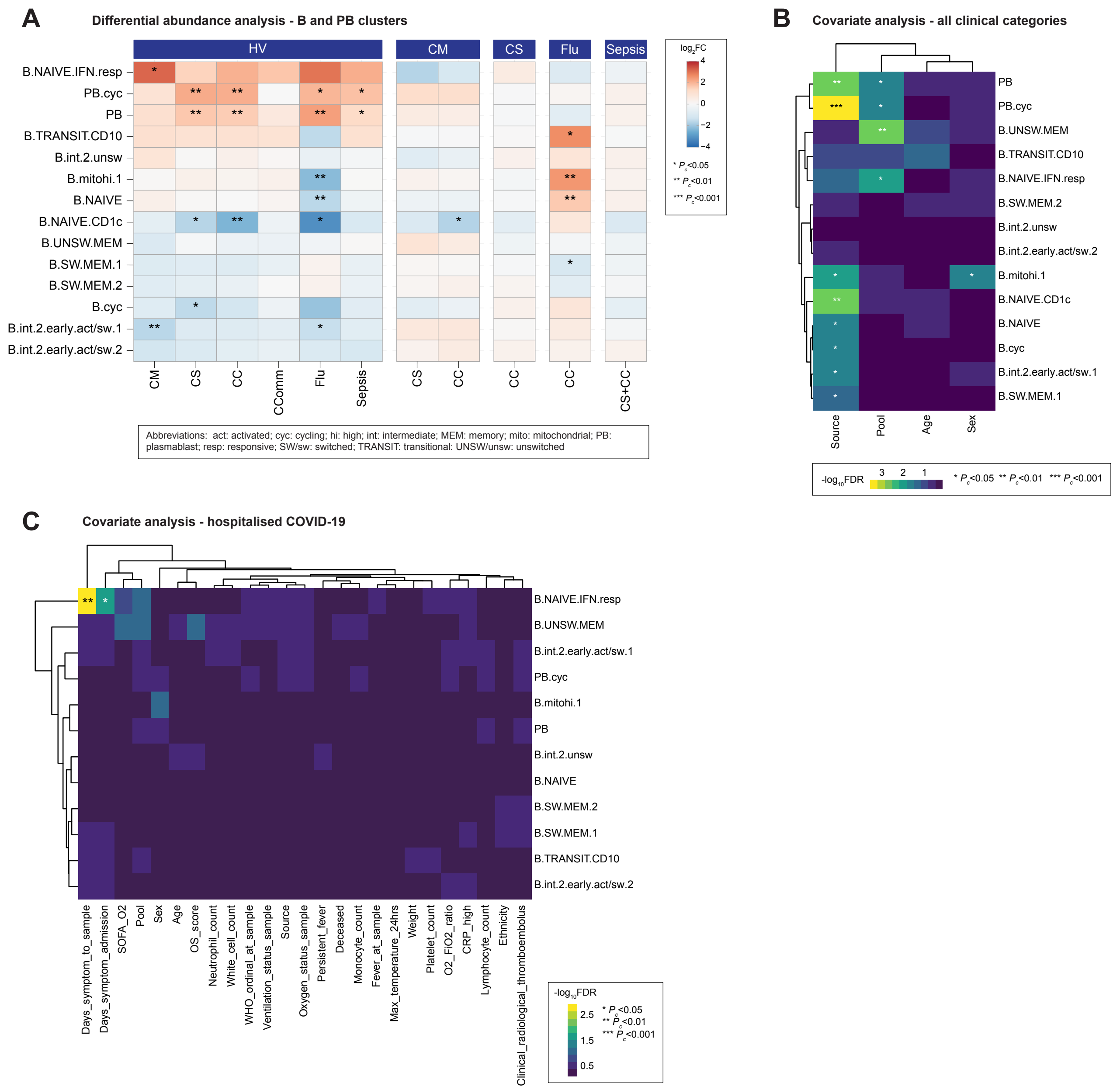

15: B and PB cluster differential abundance analysis. (A) B and PB cluster differential abundance analysis between clinical categories, controlling for age, sex and sample pool. (B) B and PB cluster covariate analysis for source (clinical category), age, sex and pool for all clinical categories. (C) B and PB cluster covariate analysis for clinical, demographic and experimental variables for hospitalized COVID-19 cases. Significance tested using Benjamini-Hochberg-corrected ANOVA. Related to STAR Methods: CITE-seq: composition analysis.

Composition analysis of mononuclear phagocyte clusters

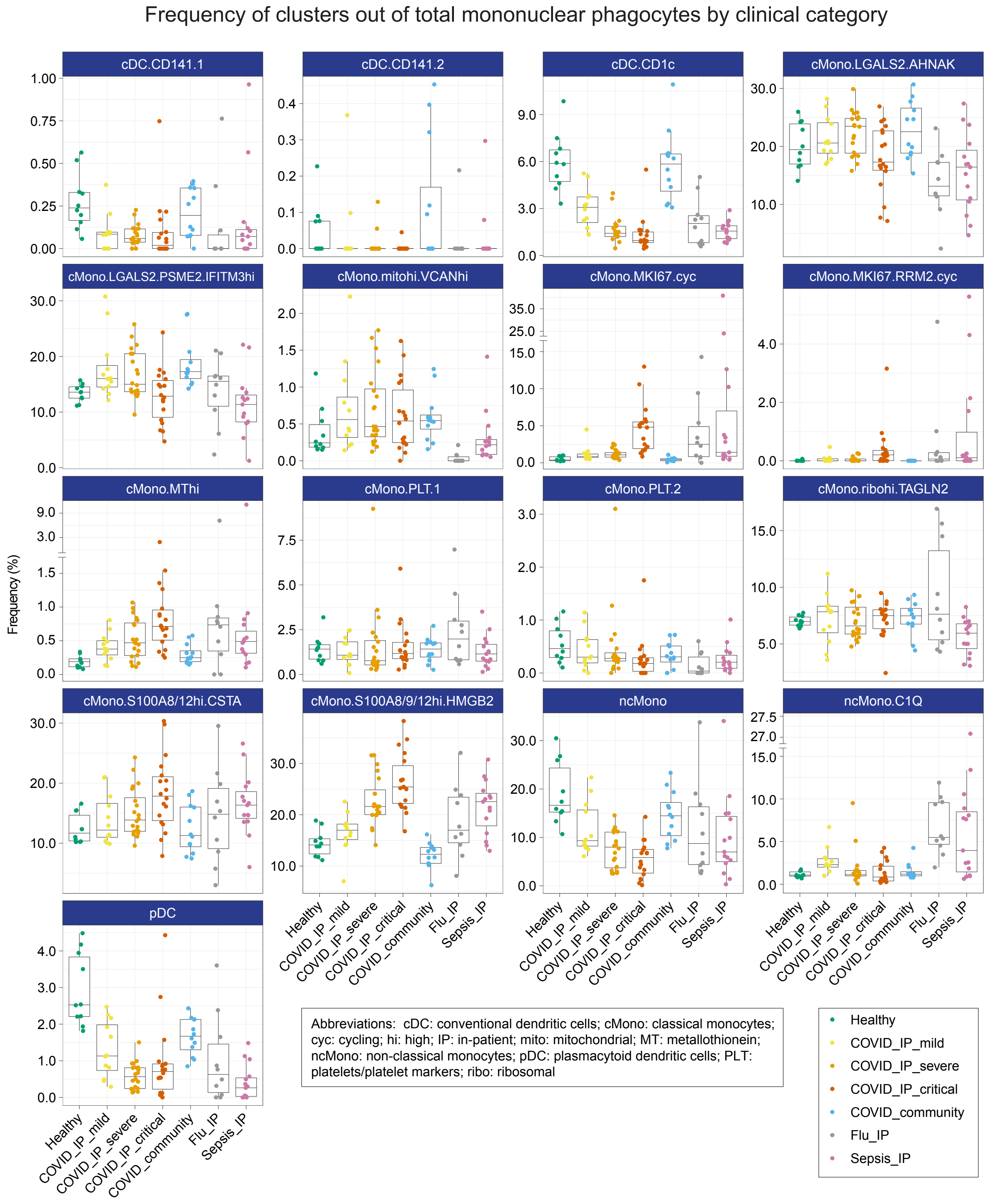

16: MNP cluster frequency by clinical category. Box plots show the median and the first and third quartiles; whiskers show 1.5 × the interquartile range above and below the box. Clusters are ordered alphabetically. Related to STAR Methods: CITE-seq: composition analysis.

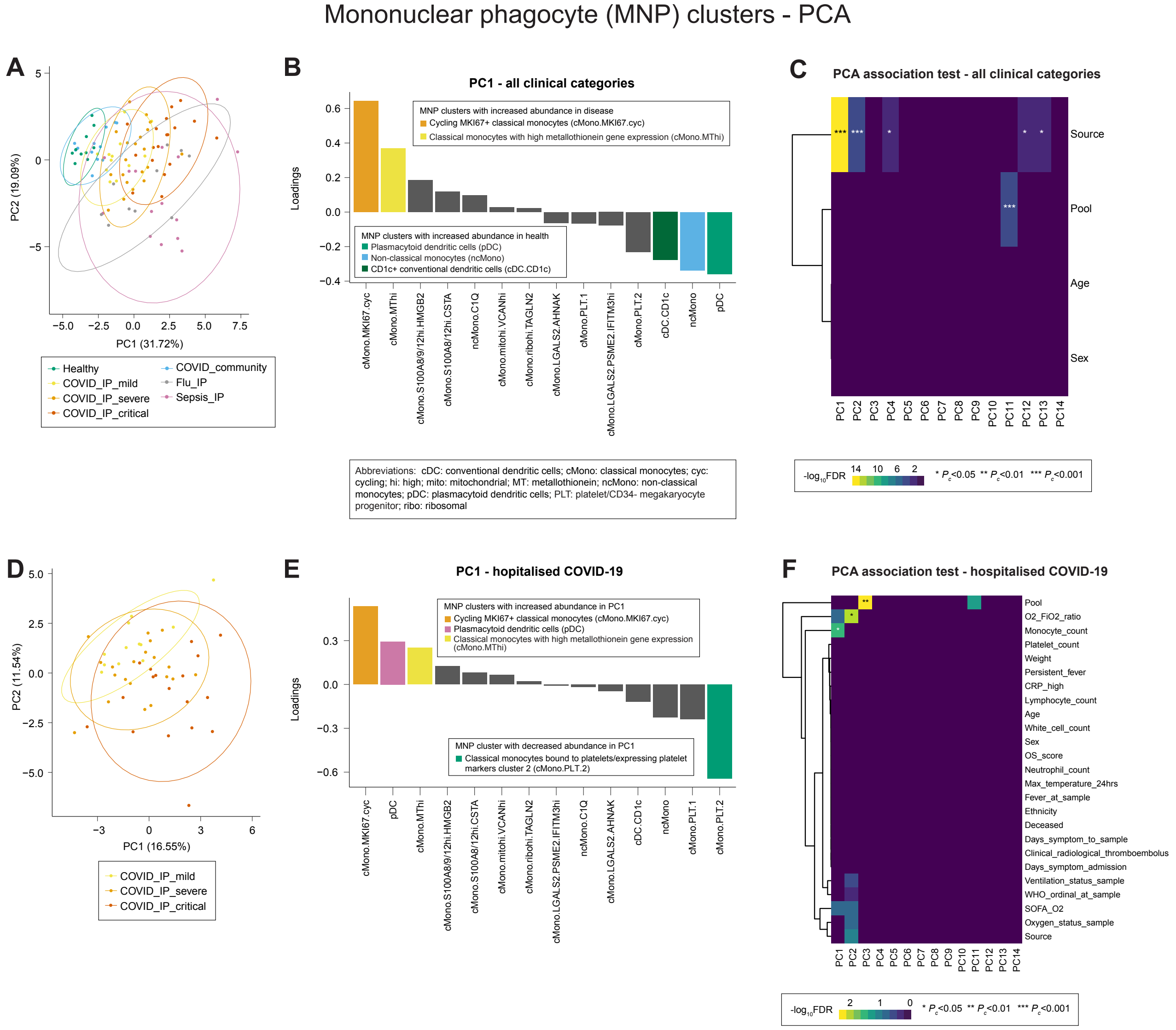

17: MNP cluster PCA and association tests. (A) PCA plot for all clinical categories. Percentage of variance explained by each PC is shown in the brackets. (B) Loadings of MNP clusters on PC1 from analysis including all clinical categories. (C) Association test between top PCs and source (clinical category), age, sex, and sample pool for all clinical categories. (D) PCA plot for hospitalized COVID-19. Percentage of variance explained by each PC is shown in the brackets. (E) Loadings of MNP clusters on PC1 from analysis of hospitalized COVID-19 cases. (F) Association test between top PCs and clinical, demographic and experimental variables for hospitalized COVID-19 cases. Significance tested using Benjamini-Hochberg-corrected ANOVA. Related to STAR Methods: CITE-seq: composition analysis.

Mononuclear phagocyte (MNP) clusters - edgeR differential abundance analysis

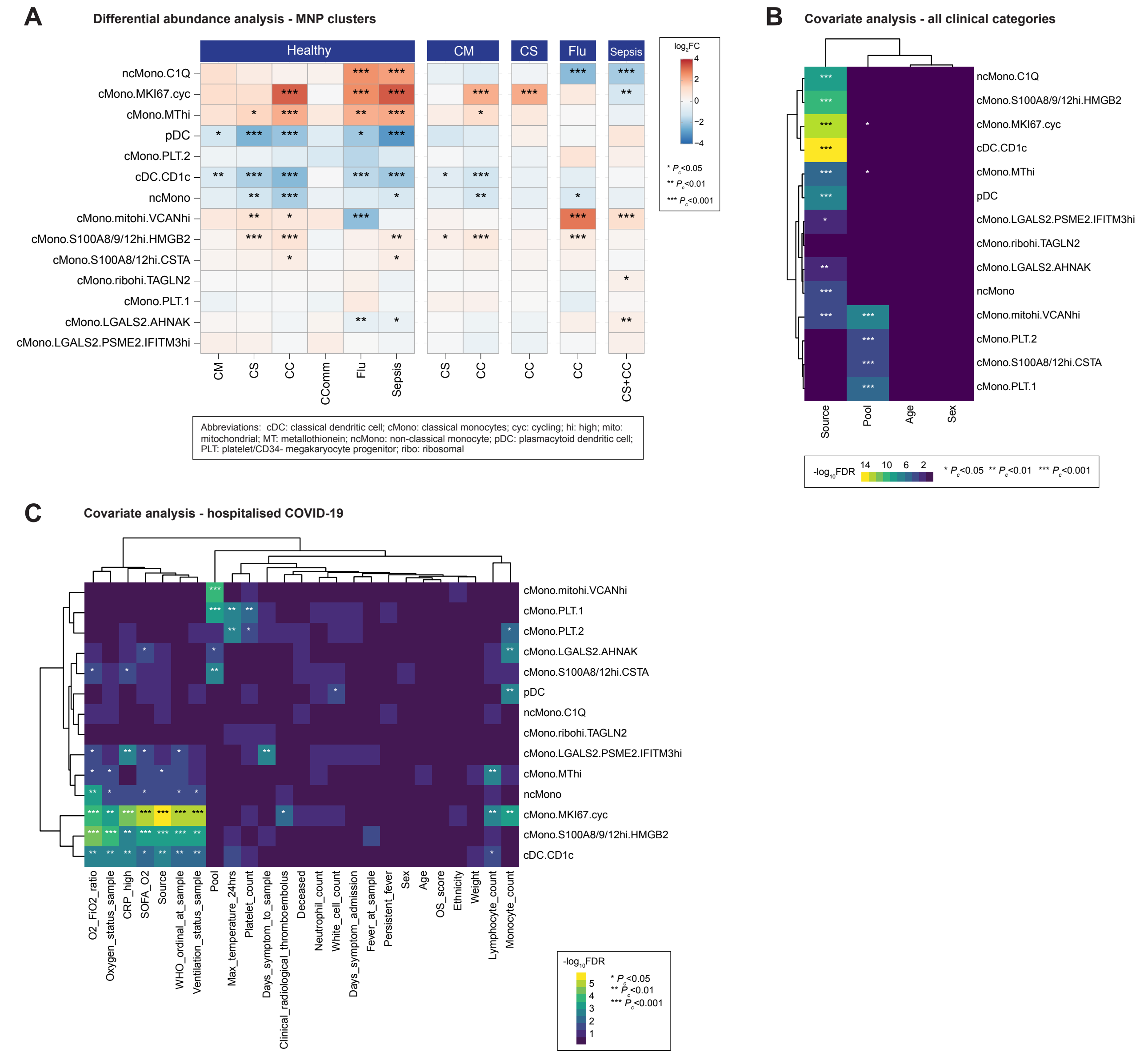

18: MNP cluster differential abundance analysis. (A) MNP cluster differential abundance analysis between clinical categories, controlling for age, sex and sample pool. (B) MNP cluster covariate analysis for source (clinical category), age, sex and pool for all clinical categories. (C) MNP cluster covariate analysis for clinical, demographic and experimental variables for hospitalized COVID-19 cases. Significance tested using Benjamini-Hochberg-corrected ANOVA. Related to STAR Methods: CITE-seq: composition analysis.
