# Supplementary material for: A blood atlas of COVID-19 defines hallmarks of disease severity and specificity
Source: Cell. 2022 Mar 3;185(5):916–938.e58. doi: 10.1016/j.cell.2022.01.012 (PMC8776501; doi:10.1016/j.cell.2022.01.012)
Supplement: Data S6: Gene expression analysis of CITE-seq data (principal component analysis, differential expression, and WGCNA parameters and modules), related to STAR Methods, Figure 3, and Table S3 [file mmc10.pdf]

Data S6: Gene Expression Analysis of CITE-seq Data (Principal Component Analysis, Differential Expression and WGCNA Parameters and Modules), Related to STAR Methods, Figure 3 and Table S3

Related to **Figure 3**; the **STAR Methods**: CITE-seq: PCA analysis, differential expression analysis and WGCNA analysis; **Table S3**: Differential Expression and Pathway Enrichment for CITE-seq Data and **Key Resource**: CBD-KEY-CITESEQ-GEX-WGCNA.

Table of Contents

Principal Components Analysis **2**
- 1: The first two principal components of pseudobulk gene expression for each of the major subsets........ 2
- 2: Association with source and COVID-19 severity for each of the first 10 PCs in each cluster, grouped by minor subset, major subset and cell type 3
- 3: Association of principle components with clinical within hospitalized patients........ 4
- 4: UMAP plots of gene expression, based on combining PCs across all clusters at the minor subset, major subset or cell type levels........ 5

Differential Expression Analysis **6**
- 5: Numbers of differentially expressed genes for different contrasts by cell cluster, colored by cell type 6
- 6: Volcano plots of differential expression for critical COVID-19 vs heathy controls for each of the major subsets 7
- 7: Relationship between mean abundance of minor subsets (i.e. the proportion of all cells that belong to this subset across all samples) and number of differentially expressed genes for a range of contrasts........ 8
- 8: Relationship between differential abundance (measured by log-fold change in cell frequency between the two contrasts) and number of differentially expressed genes for each minor subset 9

WGCNA Parameters and Modules **10**
- 9: Parameter settings for CITE-seq WGCNA analysis 10
- 10: WGCNA modules identified for each “major cell type” analysed 11

Principal Components Analysis

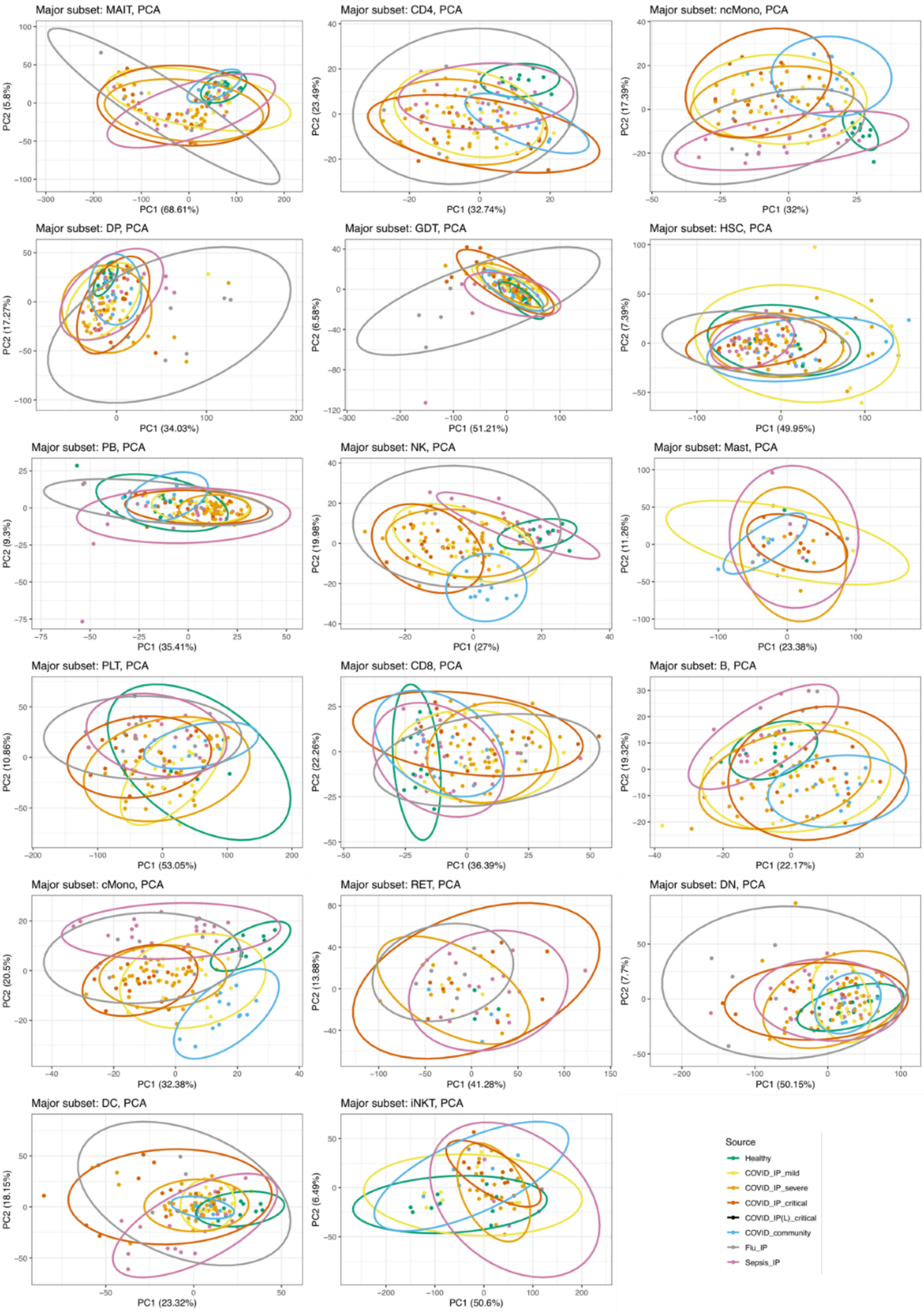

1: The first two principal components of pseudobulk gene expression for each of the major subsets, colored by sample source. Related to STAR Methods: CITE-seq: PCA analysis.

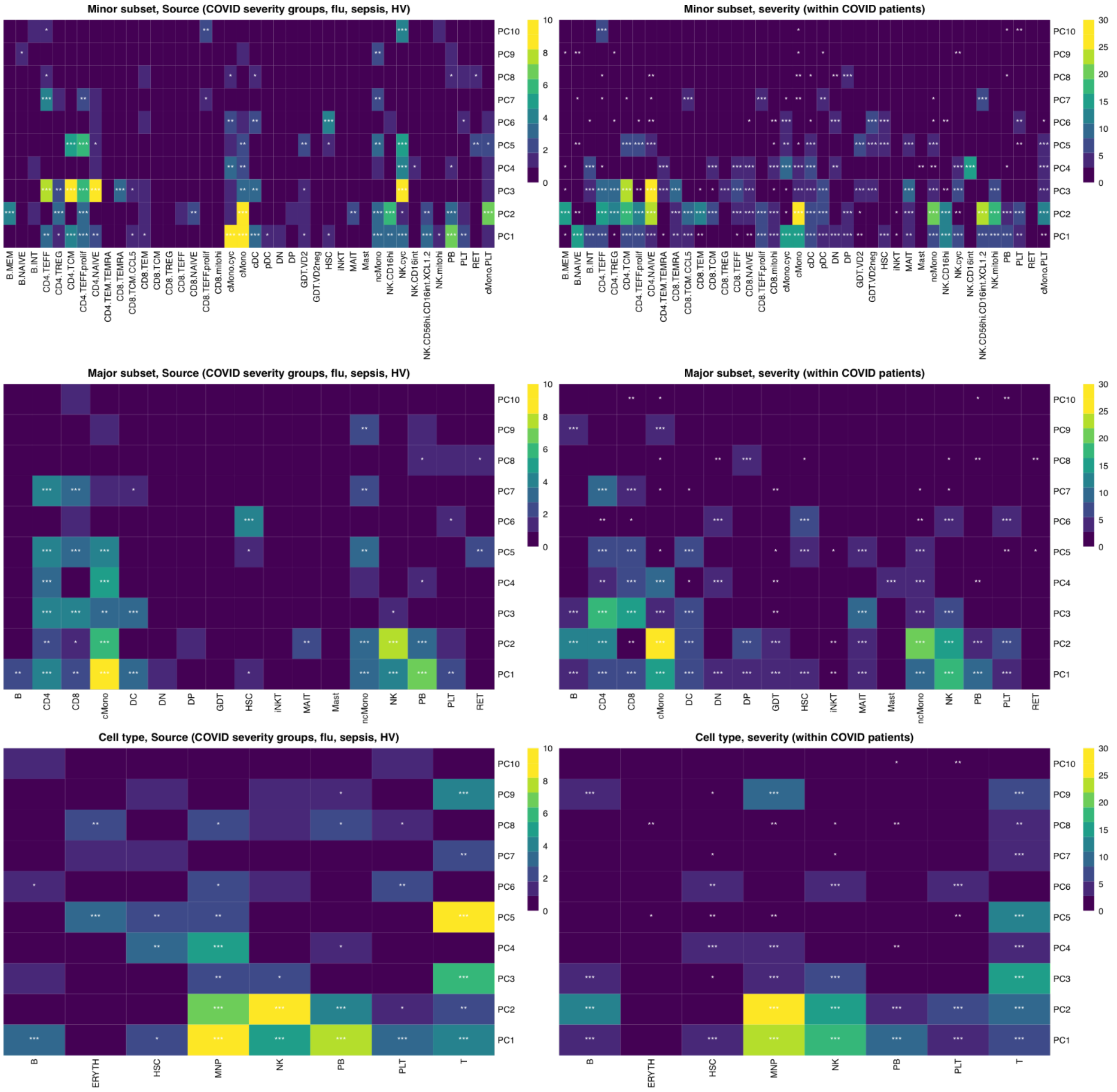

2: Association with source and COVID-19 severity for each of the first 10 PCs in each cluster, grouped by minor subset, major subset and cell type. The color shows the -log$_{10}$ transformation of the Benjamini-Hochberg corrected omnibus p-value, capped at 10 (for Source) and 30 (for severity). *** $p < 0.001$, ** $p < 0.01$, * $p < 0.05$. Related to STAR Methods: CITE-seq: PCA analysis.

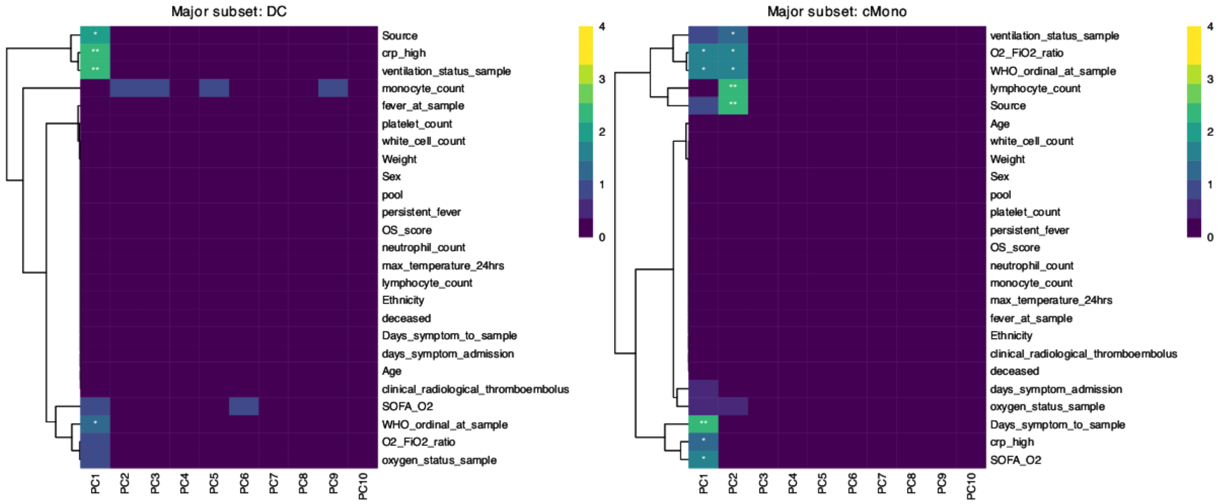

3: Association of principle components with clinical within hospitalized patients. The color shows the $-\log_{10}$ transformation of the Benjamini-Hochberg corrected omnibus p-value. *** $p < 0.001$, ** $p < 0.01$, * $p < 0.05$. Related to STAR Methods: CITE-seq: PCA analysis.

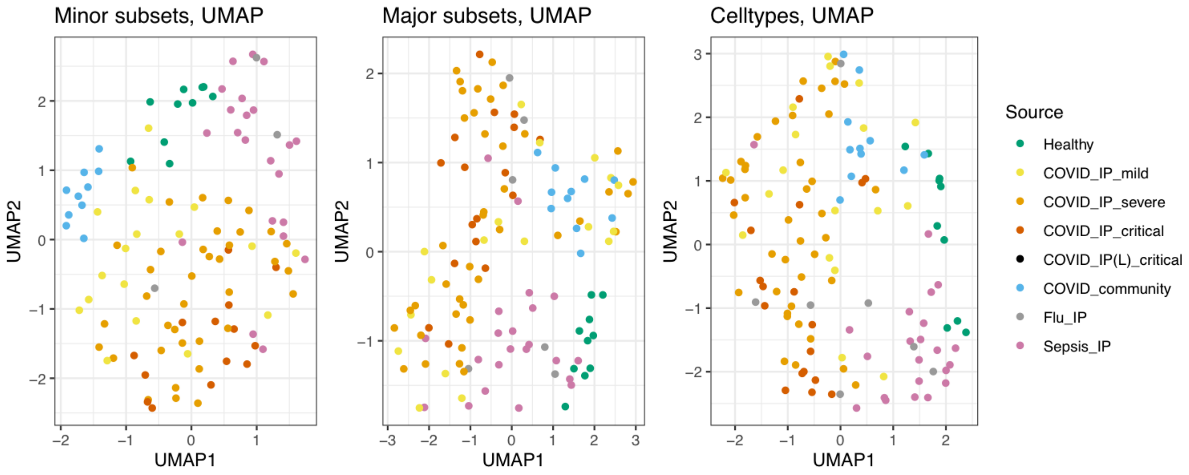

4: UMAP plots of gene expression, based on combining PCs across all clusters at the minor subset, major subset or cell type levels. Related to STAR Methods: CITE-seq: PCA analysis.

Differential Expression Analysis

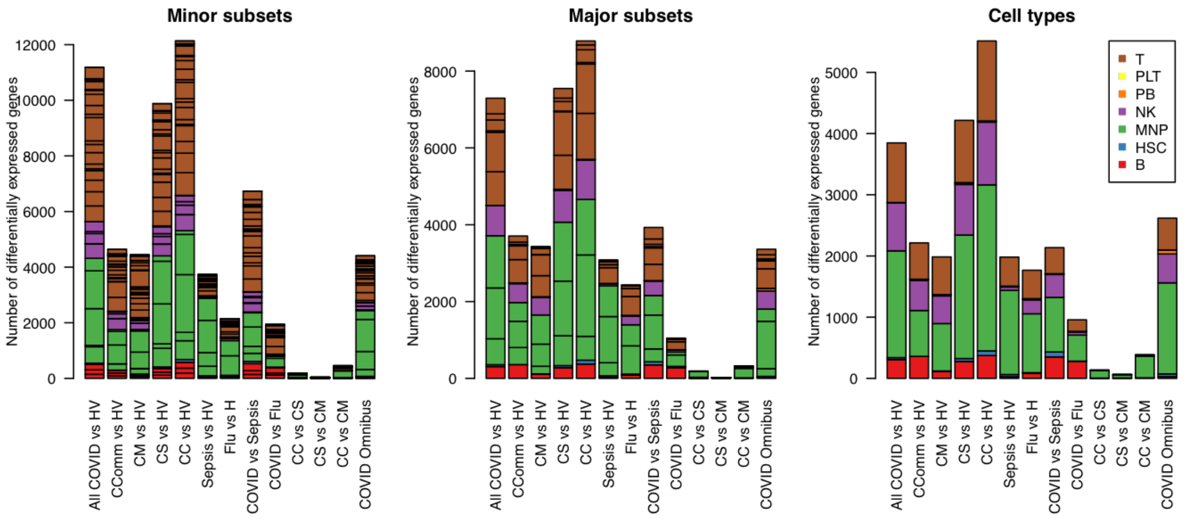

5: Numbers of differentially expressed genes for different contrasts by cell cluster, colored by cell type. The size of each individual rectangle gives the number of differentially expressed genes (FDR < 0.01, FC > 2) for a particular cell type for a given contrast, with cell types grouped and colored by cell lineage. Related to STAR Methods: CITE-seq: differential expression analysis.

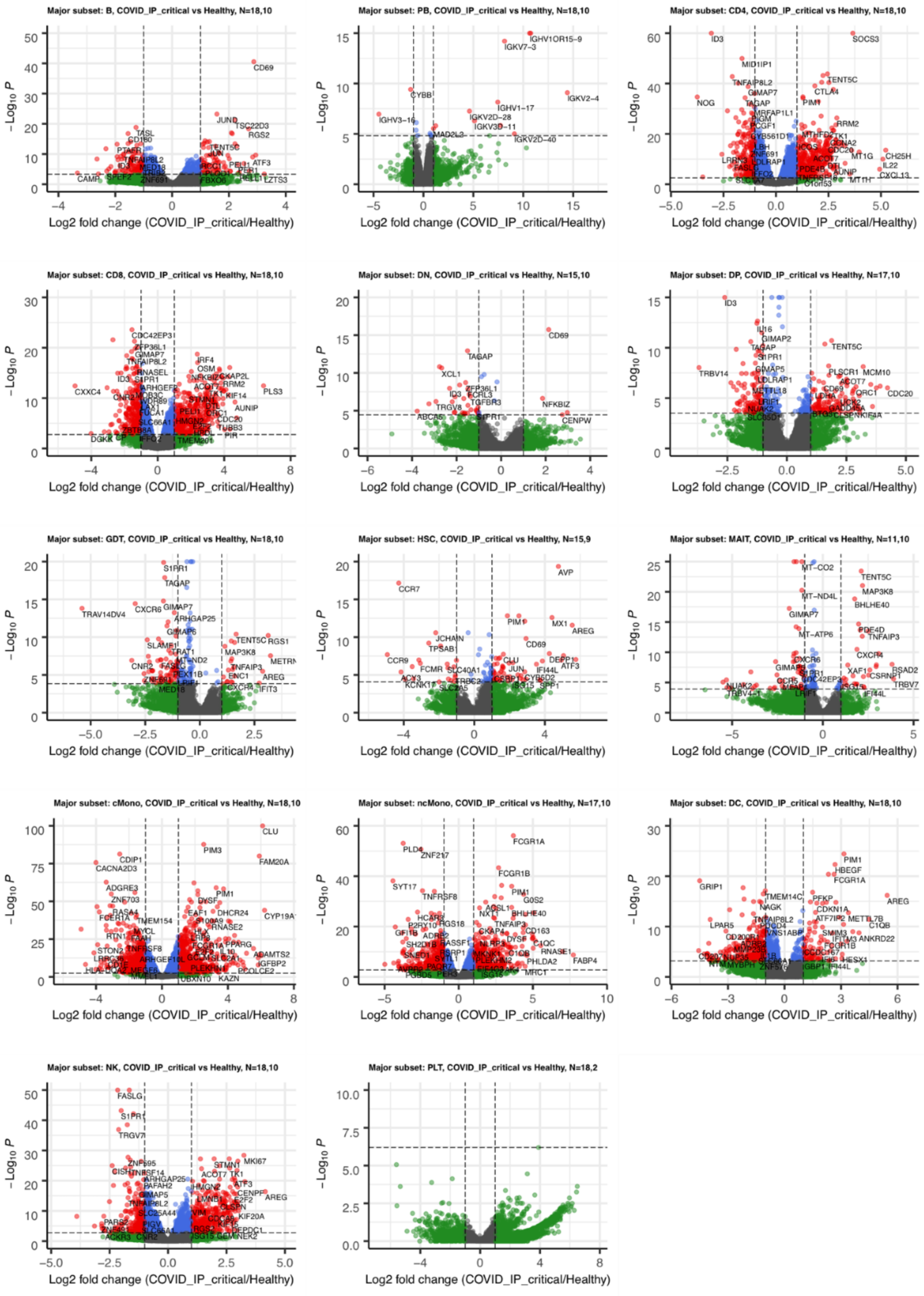

6: Volcano plots of differential expression for critical COVID-19 vs heathy controls for each of the major subsets. The N given in each title refers to the number COVID-19 patients and number of controls with a cell count high enough to be included in each analysis. Related to STAR Methods: CITE-seq: differential expression analysis.

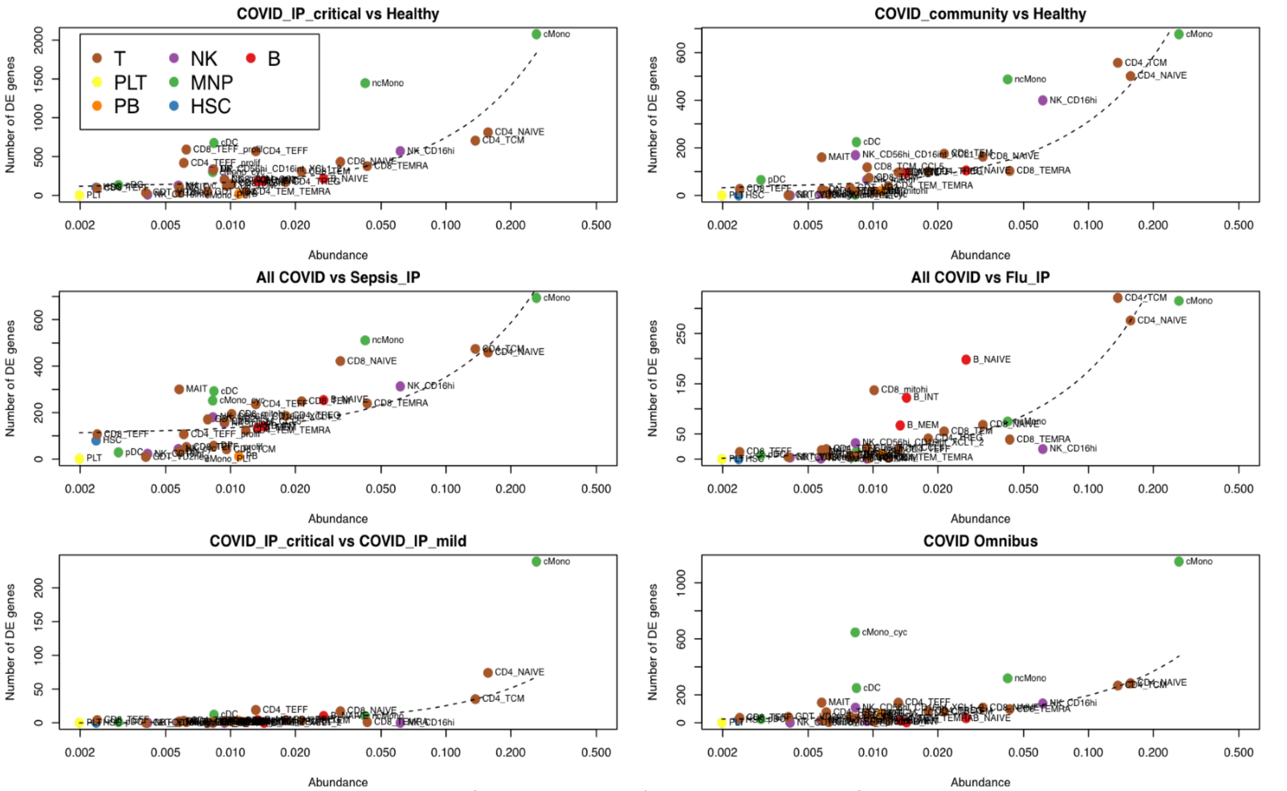

7: Relationship between mean abundance of minor subsets (i.e. the proportion of all cells that belong to this subset across all samples) and number of differentially expressed genes for a range of contrasts. Points are colored by cell type and labelled with the minor subset name. Related to STAR Methods: CITE-seq: differential expression analysis.

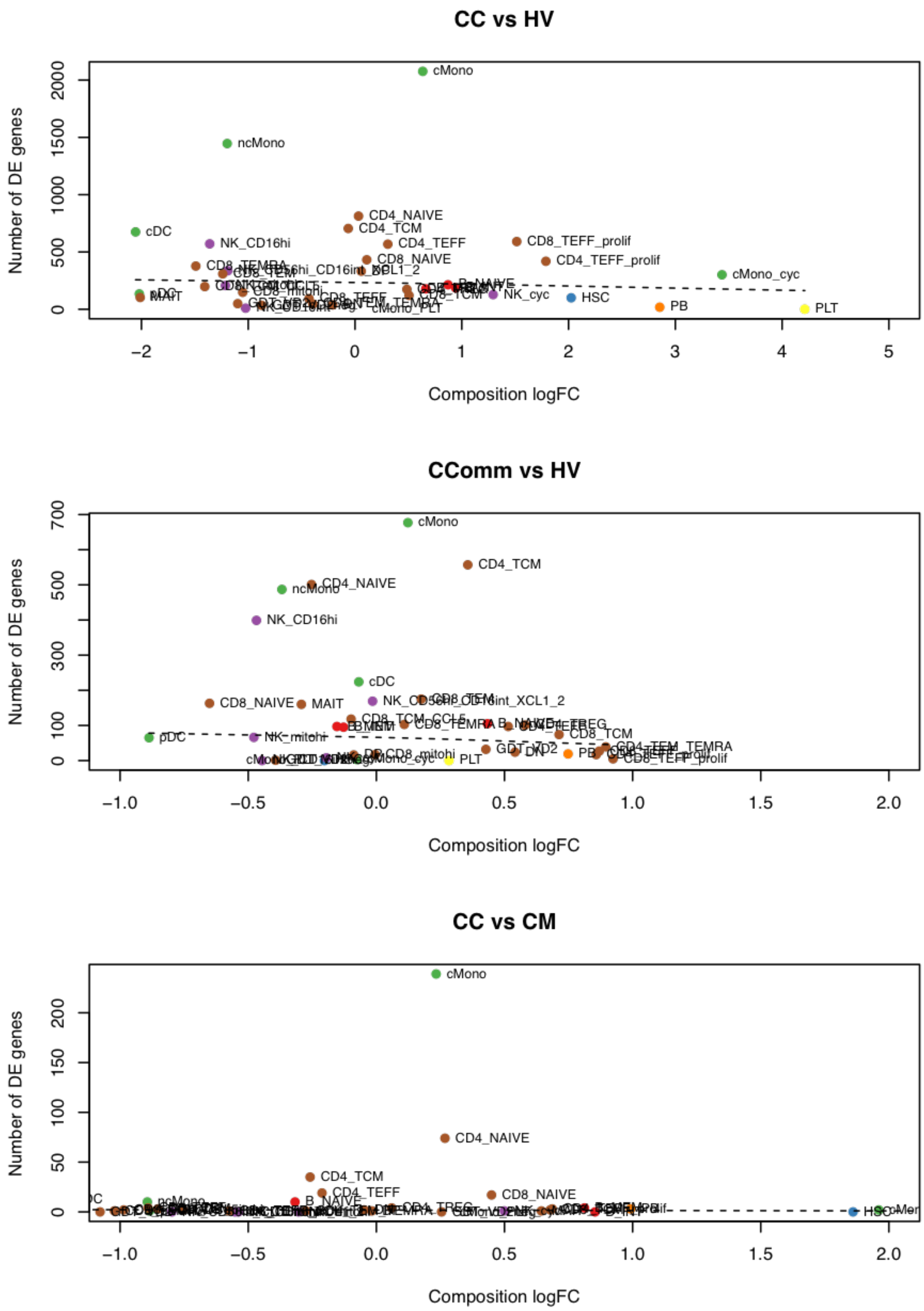

8: Relationship between differential abundance (measured by log-fold change in cell frequency between the two contrasts) and number of differentially expressed genes for each minor subset. Points are colored by cell type (using the same color scheme as Figure 42) and labelled with the minor subset name. Related to STAR Methods: CITE-seq: differential expression analysis.

WGCNA Parameters and Modules

WGCNA parameter	NK cells	cMono	ncMono	PB	B cells	CD4 T cells	CD8 T cells
network type	signed hybrid	signed hybrid	signed hybrid	signed hybrid	signed hybrid	signed hybrid	signed hybrid
adjacency correlation function	bicor	bicor	bicor	bicor	bicor	bicor	bicor
adjacency distance function	dist	dist	dist	dist	dist	dist	dist
TOM type	signed	signed	signed	signed	signed	signed	signed
minFraction (goodSamplesGenes)	0.5	0.5	0.5	0.5	0.5	0.5	0.5
minNSamples (goodSamplesGenes)	4	4	4	4	4	4	4
minNGenes (goodSamplesGenes)	4	4	4	4	4	4	4
minRelativeWeight (goodSamplesGenes)	0.1	0.1	0.1	0.1	0.1	0.1	0.1
minSize (cutreeStatic)	10	10	10	10	10	10	10
cutHeight (cutreeStatic)	125	100	200	156	200	100	120
Soft power (adjacency)	3	6	4	5	4	6	6
minimum module size (cutreeDynamic)	50	50	50	50	50	50	50

9: Parameter settings for CITE-seq WGCNA analysis

major cell type	module color	assigned name
B cell	cyan	cycling
B cell	greenyellow	ER_stress
B cell	purple	IFN_resp
B cell	pink	integrin_sig
B cell	turquoise	IRF2.ZF-C2H2
B cell	green	mature.B.cell
B cell	midnightblue	mito_translation
B cell	black	OxPhos
B cell	blue	p38MAPK.AP1
B cell	magenta	ribosomal
B cell	grey	unassigned_genes
B cell	brown	wnt.notch
CD4 T cell	lightgreen	ambient_rna
CD4 T cell	black	cycling
CD4 T cell	pink	IFN_resp
CD4 T cell	cyan	integrin_sig
CD4 T cell	turquoise	IRF2.ZF-C2H2
CD4 T cell	lightyellow	JAK_STAT.IL_sig
CD4 T cell	green	p38MAPK.AP1
CD4 T cell	midnightblue	ribosomal
CD4 T cell	grey	unassigned_genes
CD8 T cell	lightcyan	ambient_rna
CD8 T cell	blue	cycling
CD8 T cell	midnightblue	IFN_resp
CD8 T cell	yellow	IRF2.ZF-C2H2
CD8 T cell	purple	mito_translation
CD8 T cell	pink	NK_activation
CD8 T cell	black	p38MAPK.AP1
CD8 T cell	magenta	ribosomal
CD8 T cell	grey	unassigned_genes
CD8 T cell	cyan	vesicle_loc
cMono	grey60	cycling
cMono	lightgreen	FKBP5.CD163
cMono	black	IFN_resp
cMono	turquoise	IRF2.ZF-C2H2
cMono	tan	OxPhos
cMono	lightcyan	p38MAPK.AP1
cMono	green	ribosomal
cMono	grey	unassigned_genes
ncMono	yellow	cell_adhesion
ncMono	greenyellow	cell_growth
ncMono	blue	Cilium.PECAM1
ncMono	black	IC_Sig
ncMono	green	IFN_resp
ncMono	pink	IL-1_pathway
ncMono	purple	IRF2.ZF-C2H2
ncMono	salmon	OxPhos
ncMono	magenta	p38MAPK.AP1
ncMono	brown	ribosomal
ncMono	grey	unassigned_genes
NK cell	salmon	ambient_rna
NK cell	blue	cycling
NK cell	red	ECM_organisation
NK cell	purple	IFN_resp
NK cell	turquoise	IRF2.ZF-C2H2
NK cell	green	ITK.STAT4
NK cell	tan	leuk_prolif
NK cell	black	mito_translation
NK cell	brown	p38MAPK.AP1
NK cell	greenyellow	ribosomal
PB	turquoise	cycling
PB	brown	IFN_resp
PB	blue	ribosomal
PB	grey	unassigned_genes

10: WGCNA modules identified for each “major cell type” analysed
